# Sialotranscriptome of *Simulium pertinax* and functional characterization of SpVaP: a novel recombinant simuliid-specific vasodilator

**DOI:** 10.1007/s11010-026-05718-4

**Published:** 2026-09-03

**Authors:** Camila Amaro Caldeira, Lucyana Keity Santana Silva, Thays Duarte de Oliveira, Andrea de Barros Pinto Viviani Cunha, Vincent Louis Viala, Bianca de Carvalho Lins Fernandes Távora, Bianca Cestari Zychar, Bruno Miranda de Almeida, Enéas de Carvalho, Emídio Beraldo Neto, Paula Andreia Lucas Calabria, Sarah Natalie Cirilo Gimenes, Eliana Faquim de Lima Mauro, Geraldo Santana Magalhaes

**Affiliations:** 1 Immunopathology Laboratory, Butantan Institute, São Paulo, SP Brazil; 2 Superintendence for Endemic Disease Control (SUCEN), Caraguatatuba, SP Brazil; 3 Biochemistry Laboratory, Butantan Institute, São Paulo, SP Brazil; 4 Multiuser Intravital Microscopy Platform, Butantan Institute, São Paulo, SP Brazil; 5 Bacteriology Laboratory, Butantan Institute, São Paulo, SP Brazil

**Keywords:** *Simulium pertinax*, Sialotranscriptome, Recombinant proteins, SVEP, Hemostatic system

## Abstract

The salivary glands of hematophagous arthropods possess a remarkable molecular diversity, reflecting evolutionary adaptations to blood feeding lifestyle and vector-host interactions. In this study, we characterized the salivary-gland transcriptome of adult female *Simulium pertinax*, a medically important black fly species widely distributed in South America. Through RNA sequencing and bioinformatics analyses, we identified 15,708 high-quality transcripts, of which 1947 clusters were related to secreted proteins, distributed across 23 distinct functional families, including digestive enzymes, protease inhibitors, antimicrobial peptides, and hemostasis modulators. Phylogenetic analyses of selected hematophagy-associated families, including Kunitz-domain inhibitors, Apyrases, Kazal-type inhibitors, Serpins, and SVEP homologs, revealed a combination of conserved evolutionary relationships and lineage-specific diversification among simuliids. Notably, the SVEP (*Simulium* Vasodilator Erythema Protein) family was highly represented and appears to be restricted to black flies. Given that its biological role remains largely uncharacterized, we selected a novel SVEP variant for heterologous expression and functional characterization, which was designated as recombinant *Simulium pertinax* Vasoactive Protein (rSpVaP). This recombinant protein elicited vasodilator activity in the arterioles of the mouse cremaster muscle, as well as significant paw edema. These findings support rSpVaP as a biologically active SVEP-related salivary vasoactive protein in simuliids, underscoring its contribution to facilitating blood-feeding. Taken together, these results highlight the value of combining transcriptomic profiling with functional characterization to uncover the molecular adaptations underlying hematophagy in black flies, enhancing our understanding of vector-host interactions and the evolutionary pressures shaping the sialotranscriptome.

**Supplementary Information:**

The online version contains supplementary material available at https://doi.org/10.1007/s11010-026-05718-4.

## Introduction

Species of the genus *Simulium* (Diptera: Simuliidae) are recognized as vectors of pathogens affecting humans and animals, including the causative agent of onchocerciasis (“river blindness”) [1]. In Brazil, *Simulium guianense* is the main vector of onchocerciasis, while *Simulium vittatum* has been extensively studied due to its medical and veterinary relevance [2, 3]. Among South American black flies, *Simulium pertinax* is widely distributed and is of ecological and public health importance due to its aggressiveness and abundance in rural and urban areas, as well as its potential role in pathogen transmission [4]. However, the molecular biology of *S. pertinax*, particularly its salivary transcriptome (sialotranscriptome), remains poorly understood.

The saliva of hematophagous arthropods contains bioactive molecules that modulate host immune and hemostatic responses, facilitating blood feeding [4, 5]. Sialotranscriptomic studies in mosquitoes (*Anopheles gambiae*, *Aedes aegypti*), ticks (*Ixodes scapularis*), and sandflies (*Lutzomyia longipalpis*) have revealed diverse immunomodulatory, anticoagulant, and antiplatelet proteins [6–9]. In simuliids, sialotranscriptomes of *S. guianense* and *S. vittatum* identified salivary proteins associated with anticoagulant and immunomodulatory functions [10, 11], underscoring the relevance of salivary components in vector-host interactions.

In this context, characterizing the salivary repertoire of *S. pertinax* can provide valuable insights into the molecular mechanisms underlying hematophagy, host interaction, and the diversification of salivary proteins in black flies. Given its wide geographic distribution and host-feeding plasticity [4], this species represents a relevant model for expanding current knowledge of simuliid salivary biology.

In this study, we characterized the salivary-gland transcriptome of adult female *S. pertinax* through de novo transcriptome assembly and functional annotation, followed by phylogenetic comparisons of selected salivary protein families with homologs from other hematophagous arthropods. Female salivary glands were selected because females are responsible for blood feeding, making this tissue directly relevant for identifying salivary molecules potentially involved in the modulation of host hemostasis and vascular responses. Several transcripts encoding putative immunomodulatory, anticoagulant, and antiplatelet proteins were identified and phylogenetic comparisons allowed the inference of evolutionary diversification patterns. Among these, sequences homologous to the *Simulium* Vasodilator Erythema Protein (SVEP) family were particularly noteworthy due to their apparent restriction to black flies based on currently available sequences and their limited functional characterization [3]. Therefore, a representative variant was expressed in *E. coli* and designated as rSpVaP (recombinant *S. pertinax* Vasoactive Protein). The purified rSpVaP was soluble and exhibited marked vasodilatory and edematogenic activities *in vivo*, as demonstrated by intravital microscopy and paw edema assays.

This integrative approach demonstrates the feasibility of combining transcriptomic screening with functional validation, providing a framework for future investigation of additional salivary proteins in *S. pertinax* and their role in modulating host hemostasis during blood feeding.

## Material and methods

### Sample collection and processing

The specimens of *S. pertinax* were collected in the region of Ilhabela, on the north coast of the state of São Paulo, Brazil, in collaboration with the Superintendence of Endemic Diseases (SUCEN). Arthropod collections were carried out using an entomological net at locations and times of peak arthropod activity, aiming to maximize the number of specimens captured.

The captured arthropods were anaesthetized with cotton wool moistened in ethyl acetate and packed in entomological boxes for transport to the SUCEN laboratory in Caraguatatuba (São Paulo, Brazil). There, they were sorted and identified, and their salivary glands were dissected and immediately preserved in TRIzol reagent (Thermo Fisher Scientific) for downstream molecular analysis.

### RNA extraction and cDNA library preparation and sequencing

Total RNA was extracted from a single pooled sample comprising salivary glands dissected from approximately 600 adult female specimens, using TRIzol reagent (Thermo Fisher Scientific) following the manufacturer’s protocol. The mRNA was isolated using the Dynabeads^®^ mRNA DIRECT kit (Invitrogen, Life Technologies Corp.) based on oligo (dT)-conjugated magnetic beads. The mRNA was quantified using the Quant-iT™ RiboGreen^®^ RNA kit (Invitrogen, Life Technologies Corp.), and the integrity of the samples was checked on a Bioanalyzer 2100 with picochip (Agilent Technologies Inc.). A single pooled cDNA library was constructed from 15 ng of mRNA, previously fragmented in a ZnCl_2_ solution at 70 °C for 30 s. The first strand of cDNA was synthesized using a standard system (Roche Diagnostics) with random primers. The fragments were then subjected to terminal repair, followed by adapter ligation using a rapid cDNA library preparation kit (Roche Diagnostics). Purification was carried out using Agencourt AMPure XP beads (Beckman Coulter Inc.). The cDNA library was diluted to 10 nM, denatured with 0.1 N NaOH, and adjusted to a final concentration of 12 pM, spiked with 5% PhiX control (12 pM). Sequencing was carried out on Illumina HiSeq 1500 equipment, using the TruSeq Rapid PE Cluster v2 and TruSeq SBS v2 500 cycles kits (Illumina), resulting in paired reads of 251 base pairs (2 × 251 bp paired-end).

### Bioinformatics and phylogenetic analysis

The sequencing data were processed as previously described in De Lucca Caetano *et al.* [12]. Briefly, raw reads were demultiplexed using CASAVA, and only reads with Phred quality scores ≥ Q30 were retained for downstream analyses. An in-house pipeline was used for read preprocessing, including removal of homopolymers, low-complexity regions, adapters, and low-quality bases, using FASTQ-mcf; contaminant reads were removed by mapping with Bowtie2. Reads shorter than 40 bp were excluded. The transcriptome was assembled with rnaSPAdes (k-mer 55) [13]. To reduce redundancy, assembled transcripts were hierarchically clustered using Corset, and representative transcripts from each cluster were retained for subsequent analyses.

Mitochondrial sequences were removed from both the assembly and the read set to avoid bias in the estimation of nuclear transcript abundance. Because no mitochondrial reference sequence was available for *S. pertinax*, reads were first mapped against the complete mitochondrial genome of *S. guianense* (NCBI accession NC_088444) using the Geneious mapper (low sensitivity, two iterations), and the resulting consensus was used as a species-specific reference for a second mapping round. Reads that did not map to this reference were retained as the nuclear-enriched dataset, and mitochondrial transcripts identified by BLAST were excluded from the assembly.

The non-mitochondrial reads were then mapped onto the predicted coding sequences (CDS; protein-coding regions) of the transcriptome (unigene.blast.cds), rather than to the full transcript sequences, to avoid potential interference from untranslated regions (UTRs) in transcript abundance estimates. Based on CDS length and the number of mapped reads, transcript abundance was estimated using FPKM (Fragments Per Kilobase of transcript per Million mapped reads), which normalizes read counts by both transcript length and sequencing depth. FPKM was used as the expression metric for the family-level abundance analysis because it was consistently available for the classified clusters in the complete annotation and expression dataset.

For functional annotation of the transcripts, we used the BlastX tool to compare nucleotide sequences with the non-redundant (NR) protein database from the National Center for Biotechnology Information (NCBI, National Library of Medicine, NIH) and with additional databases, including SwissProt, Gene Ontology (GO), Eukaryotic Orthologous Groups (KOG), Kyoto Encyclopedia of Genes and Genomes Orthology (KEGG Orthology or KO), Molecular Function (MF), and Protein Families Database (PFAM). Similarity identification was conducted using the BLAST tool [14]. Open reading frames (ORFs) longer than 60 amino acids were predicted with TransDecoder, and the resulting protein sequences were screened for secretory signal peptides with SignalP 5.0. Clusters were subsequently partitioned into a predicted secretory fraction. For the analysis of predicted secreted protein families, only transcripts encoding proteins with signal peptides were considered. These transcripts were assigned to functional families based on BLAST similarity, domain annotation, and correspondence with known salivary protein groups. When multiple annotation hits were available for the same cluster, the family assignment was based on the hit most consistent with the predicted secreted salivary protein repertoire. Clusters included in the curated “Other putative secreted peptides” group were retained in this category only when no clear annotation to a specific secreted protein family was detected.

To estimate the family-level contribution to the predicted secreted-protein repertoire, FPKM values of all clusters assigned to the same functional family were summed. The relative abundance of each family was then calculated as:

Relative abundance of family (%) = [Σ FPKM of clusters assigned to that family/Σ FPKM of all classified predicted secreted-protein clusters included in the analysis] × 100.

This FPKM-based calculation was used to describe the relative contribution of each predicted secreted protein family within the *S. pertinax* salivary-gland transcriptome. Thus, the resulting percentages represent family-level relative transcript abundance within the classified predicted secreted-protein dataset, not the number of sequences per family.

For the phylogenetic analysis, the main proteins of interest were chosen, their characteristic domain architecture was further confirmed using HMMER and each group was aligned with sequences from other simuliids and culicids available in the public GenBank database. Multiple sequence alignment was carried out using ClustalW [15] implemented in the MEGA X software [16]. The trees were generated using the neighbor-joining (NJ) method implemented in the MEGA X software and using bootstrap statistical tests for evolutionary inference.

### rSpVaP expression

Based on our transcriptomic analysis, a highly represented and complete sequence from the SVEP family (transcript SIMPER PX899338) was selected for functional characterization and designated as *S. pertinax* Vasoactive Protein (SpVaP). This transcript encodes a 152-residue precursor in which SignalP 5.0 predicts a 20-residue signal peptide (MSFLQIVVVLTVAIFGTATA); the mature protein therefore comprises 132 residues, beginning at Asp1 (DLIANGNCIT…) and ending at Thr132. Only this mature sequence was included in the expression construct. A nucleotide sequence codon-optimized for *E. coli* (by GenOne Biotechnologies) encoding the SVEP variant identified in the *S. pertinax* sialotranscriptome was synthesized and cloned into the pET-28a(+) expression vector. The sequence corresponding to the mature protein was cloned into pET-28a(+), introducing a C-terminal six-histidine tag to enable affinity purification.

Chemically competent *E. coli* BL21 Star™ (DE3) cells were transformed with the pET28a (+)-SpVaP plasmid. A single colony was selected and cultured overnight at 30 °C in LB medium supplemented with kanamycin (50 µg/mL). After this period, the culture was diluted 1:50 in fresh liquid LB medium containing the same antibiotic concentration and incubated until mid-log phase (OD_600_ ≈ 0.6). Recombinant protein expression was induced with 0.1 mM IPTG for 4 h at 30 °C. Cells were harvested by centrifugation (10,000×g, 15 min, 4 °C) and stored at − 20 °C until purification.

### rSpVaP purification

The rSpVaP purification was performed according to De Lucca Caetano *et al*. [12]. Bacterial pellets were resuspended in binding buffer (20 mM sodium phosphate, pH 7.4, 500 mM NaCl, 20 mM imidazole) and lysed by ultrasonication on ice. Sonication was performed at 20% amplitude using 3-second pulses followed by 4-second intervals, for a total duration of 90 s per cycle, with 4-minute pauses between cycles for cooling. This procedure was repeated five times. Cell lysates were clarified by centrifugation (10,000×g, 15 min, 4 °C), and the soluble fraction was subjected to immobilized metal affinity chromatography (IMAC) in a ÄKTA start™ closed system.

The purification was performed using Ni-Sepharose^®^ 6 Fast Flow resin (GE Healthcare). After washing, rSpVaP was eluted with 0% to 100% linear gradient of elution buffer (20 mM sodium phosphate, 500 mM NaCl, and 1M imidazole) by gradient. Eluted fractions were dialyzed against Tris-buffered saline (20 mM Tris, 150 mM NaCl, pH 7.4). Protein purity and integrity were assessed by 13.5% SDS-PAGE under reducing conditions and stained with Coomassie Brilliant Blue. Endotoxin was removed from the protein samples using the Pierce™ High-Capacity Endotoxin Removal Resin kit.

The concentration of the rSpVaP was determined in duplicate by the bicinchoninic acid method [17] using the Pierce™ BCA Protein Assay Kit (Thermo Fisher Scientific, Waltham, MA, USA) and BSA (Sigma Chemicals, St. Louis, MO, USA) to generate the standard curve following the manufacturer’s protocol. A 96-well flat-bottom plate was used, and the proteins were incubated with the reagent at 37°C for 1 hour, followed by reading on the SpectraMax 340 spectrophotometer (Molecular Devices, Sunnyvale, CA).

### Western blot analysis

For immunodetection, proteins were transferred to nitrocellulose membrane, using the Trans-Blot^®^ SD Semi-Dry Transfer Cell (Bio-Rad^®^ Laboratories, Hercules, CA, USA) following the manufacturer’s instructions with modifications according to Calabria *et al*. [18]. The antibodies used were a mouse monoclonal anti-polyhistidine antibody (Sigma Life Science, Merck Corporation, Darmstadt, Germany) at a 1:1,000 dilution, and peroxidase conjugated secondary antibody anti-mouse IgG (Sigma Life Science, Merck Corporation, Darmstadt, Germany) at a 1:1000 dilution. The antigenic components were revealed with 0.05% (w/v) 4-chloro-1α-naphthol in 15% (v/v) methanol in the presence of 0.03% H2O2 (v/v).

### Mass spectrometry

Mass spectrometry was performed according to Ferraz *et al*. [19]. The rSpVaP samples were digested in solution under the following conditions: (1) 5 μL of DTT (100 mM dithiothreitol) was added, and the mixture was kept at 60 °C for 30 min; (2) 2.5 μL of iodoacetamide (200 mM) was added and the samples were incubated for 30 min at room temperature, protected from light; (3) the sample was incubated for at least 12 h at room temperature with 10 μL of trypsin (40 ng/μL in 100 mM ammonium bicarbonate). The reaction was stopped by adding 50% ACN/5% TFA.

The samples were analyzed by liquid chromatography coupled with mass spectrometry in an ESI-IT-TOF system coupled to a UFLC (20A Prominence, Shimadzu). Each sample was injected into a C18 column (Kinetex C18, 5 μm; 50 × 2.1 mm) in a binary solvent system: (A) water:DMSO:acid (949:50:1) and (B) ACN:DMSO:water:acid (850:50:99:1). Gradient elution from 0 to 40% of solvent B was used for 35 min at a constant flow rate of 0.2 mL min⁻^1^. The samples were monitored by a Shimadzu SPD-M20A PDA detector before injection into the mass spectrometer. The interface voltage was 4.5 kV; the capillary voltage was 1.95 kV at 200 °C; and argon collision-induced fragmentation was set to 55% “energy.” MS spectra were acquired in positive mode, in the mass range of 350–1400 per charge (m/z). MS/MS spectra were collected in the range of 50 to 1950 m/z.

The raw data obtained in Shimadzu LCD software were converted (LCMS Protein Post run, Shimadzu) to mzXML format files prior to analysis. Peaks Studio V7.0 software (BSI, Toronto, ON, Canada) was used for processing (de novo peptide sequencing and proteomic identification). Proteomic identification was performed according to the following parameters: mass error (MS and MS/MS) set to 0.1 Da; methionine oxidation and carbamoylmethylation as variable and fixed modifications, respectively; trypsin method; maximum of 3 missed cleavages, maximum of 3 variable post-translational modifications (PTMs) per peptide, and 1 non-specific cleavage. Samples were analyzed against a customized protein database generated from the in-silico translation of the transcriptome generated in-house database.

### Circular dichroism and structural modelling

Circular dichroism (CD) spectra were recorded on a Jasco J-810 spectropolarimeter using a quartz cuvette with a 1 mm path length. rSpVaP was prepared in 10 mM sodium phosphate buffer, pH 7.4, at 0.1 mg/mL. Spectra were acquired from 185 to 260 nm at a scanning speed of 50 nm/min with the temperature controlled at 25 °C, and each spectrum corresponds to the average of ten accumulations; the buffer baseline was subtracted from all spectra. Ellipticity values were converted to mean residue ellipticity (MRE, deg·cm^2^·dmol⁻^1^) according to MRE = (θ × MRW) / (10 × c × l), where θ is the observed ellipticity in millidegrees, MRW is the mean residue weight (molecular mass in Da divided by the number of residues minus one), c is the protein concentration in mg/mL and l is the path length in cm. Secondary structure content was estimated with DichroWeb using the CDSSTR algorithm and a reference protein dataset.

In parallel, the three-dimensional structure of SpVaP was predicted with AlphaFold 3 using the mature sequence only, after removal of the signal peptide predicted by SignalP 5.0. The top-ranked of five models (pTM = 0.92; mean pLDDT = 94.7; pairwise Cα RMSD between models ≤ 0.15 Å) was retained. Secondary structure was assigned with DSSP 4.2.2 (Kabsch and Sander, 1983) and compared with the CD-derived estimates. Molecular graphics were prepared with PyMOL 3.2 (Schrödinger, LLC), and the diagrams in Fig. 7b, d were generated with Matplotlib 3.10 (Hunter, 2007). The model coordinates, the DSSP assignment and the scripts used to generate Fig. 7 are provided as Supplementary Data. S1.

### Animals

Male BALB/c mice weighing 20–25 g supplied by the housing facility of the Instituto Butantan were used. The animals were maintained for two days in a 12:12 h light: dark cycle and received water and food ad libitum, before experiments. All experimental procedures were conducted according to the ethical parameters proposed by the International Society of Toxinology and the Brazilian College of Experimental Animals and were approved by the Ethical Committee for the Use of Animals of Butantan Institute (n^o^ 8410020523).

### Intravital microscopy analysis

Intravital microscopy was performed to evaluate the immediate effects of rSpVaP on the microcirculation in the mouse cremaster muscle. For this purpose, 30 μL of rSpVaP (8.6 μM), recombinant glutathione S-transferase (rGST;8.6 μM), used as an unrelated protein control, or Tris-HCl buffer (0.1 M, pH 8.0) were topically applied to the cremaster muscle using a micropipette. The concentration applied corresponds to approximately 3.9 µg of rSpVaP per application, and was defined with reference to the concentration range over which recombinant SVEP from *S. vittatum* was shown to relax rabbit aortic rings [20], adjusted upwards to account for the topical route of administration and for the absence of potency data for SpVaP. Prior to use, endotoxins were removed from recombinant protein preparations using the Pierce™ High-Capacity Endotoxin Removal Resin Kit.

Mice were anesthetized with ketamine hydrochloride (100 mg/kg), xylazine (5 mg/kg), and midazolam (5 mg/kg), administered subcutaneously. The administration volume was standardized to 5 mL/kg. The cremaster muscle was exteriorized and mounted on a temperature-controlled transparent stage maintained at 37 °C, allowing direct visualization of the microvascular bed [21]. Microcirculatory observations were performed using an Olympus BX51WIF light microscope equipped with an Olympus DP74 digital color camera. Images were captured and analyzed using CellSens Dimension software.

Arterioles were randomly selected in each animal (20–50 μm in diameter and at least 100 μm in length). Following a 10 min stabilization period, rSpVaP, rGST, or Tris-HCl buffer were topically applied to the cremaster muscle. Arteriolar diameter was measured at 5, 10, and 15 min after treatment to assess vasodilatory responses. Seven animals were used per experimental group, and two independent experiments were performed.

### Edematogenic activity

To assess the local inflammatory response, paw edema was induced by intraplantar injection of recombinant SpVaP (12 µM, 30 µL) into the right hind paw. Recombinant glutathione S-transferase (rGST; 12 µM, 30 µL) and Tris-HCl buffer (0.1 M, pH 8.0, 30 µL) served as controls. The concentration applied corresponds to approximately 5.4 µg of rSpVaP per injection, defined on the same basis as described for the intravital microscopy assay. Both assays were conducted as single-concentration functional screens, and no concentration–response series was performed. Before use, endotoxin was removed from protein samples using the Pierce™ High-Capacity Endotoxin Removal Resin kit. Paw thickness was measured using a digital caliper (Starrett^®^) before injection and at 0.5, 1, 3, 6, and 24 h post-injection. Results were expressed as the difference from baseline measurements. Five animals per group were analyzed in two independent experiments.

### Statistical analysis

Evaluation of paw edema and intravital microscopy time-course data was performed using GraphPad Prism 10. Both datasets were analyzed by two-way repeated-measures ANOVA, with treatment and time as factors, followed by Šídák’s multiple-comparisons test with adjusted p-values. For both assays, comparisons were performed between rSpVaP and each control group, Tris–HCl and rGST, at the corresponding time points. Results are expressed as mean ± SEM unless otherwise indicated, and p < 0.05 was considered statistically significant.

## Results

### General aspects of the assembly

The RNA-seq run generated 22,490,995 paired-end reads, corresponding to 44,981,990 total reads. After mRNA enrichment, adapter and quality filtering, de novo assembly, hierarchical clustering, and redundancy reduction, the initial transcriptome output comprised 28,544 clusters. To minimize interference from mitochondrial-derived sequences in nuclear gene expression analyses, clusters with mitochondrial annotation or mapping evidence were excluded from this initial dataset. After this curation step, 15,708 nuclear-enriched clusters were retained for downstream annotation and expression analyses.

Functional annotation of these transcripts using the GO database revealed a broad distribution across Biological Process, Cellular Component, and Molecular Function domains (Fig. S2). This general annotation profile was dominated by terms related to cellular and metabolic processes, cellular and intracellular components, and molecular functions associated mainly with catalytic activity and binding, indicating that the assembled dataset captured a transcriptionally diverse salivary gland repertoire.

Among the 15,708 nuclear-enriched clusters, 1947 clusters were predicted to encode proteins with signal peptides, representing approximately 12.4% of the sialotranscriptome. Because secreted salivary proteins are directly relevant to blood feeding and vector–host interactions, subsequent analyses focused on this predicted secretory subset. These putative secreted proteins were classified into functional families/categories through automated annotation using SwissProt, GO, KOG, KO, MF, and PFAM databases. This secretory repertoire included Antigen-5, Kunitz-domain inhibitors, trypsins, amylases, Apyrases, SVEP, defensins, and other protein families commonly associated with digestion, immune defense, protease inhibition, and host interaction [10, 11, 22]. Similar families have also been reported in other *Simulium* sialotranscriptomes (*S. vittatum, S. guianense, S. nigrimanum*) [10, 11, 22], as well as in various *Culicidae* species, including *A. aegypti, Anopheles braziliensis, A. gambiae, and Anopheles funestus* [6, 8, 23, 24].

**Fig. 1:**
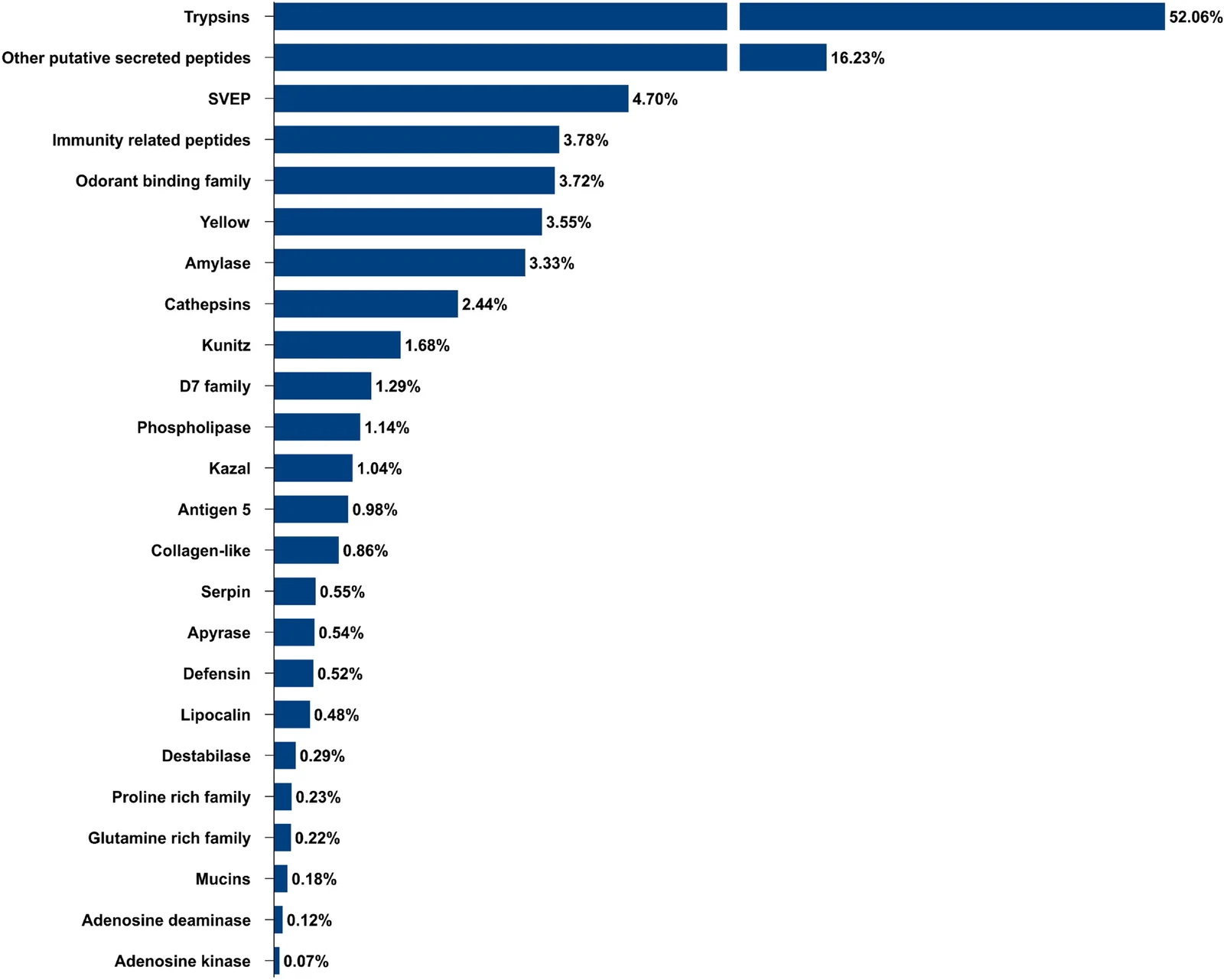
FPKM-based relative abundance of predicted secreted protein families in the S. pertinax salivary-gland transcriptome. Predicted secreted clusters were grouped by functional family, and FPKM values were summed within each group. Percentages represent the contribution of each family to the total FPKM of the predicted secreted-protein dataset. Thus, the graph reflects relative transcript abundance, not the number of sequences per family. A visual break was introduced in the bars corresponding to Trypsins and Other putative secreted peptides to improve visualization of the lower-abundance protein families. The percentage values shown next to each bar represent the actual calculated relative abundance

Transcript abundance analysis of the 1,947 SignalP-positive clusters encoding predicted secreted-proteins (Fig. 1) was performed by summing FPKM values for all clusters assigned to each functional family and expressing each family as a percentage of the total FPKM of the predicted secreted-protein dataset. This analysis revealed that trypsin-related sequences showed the highest relative abundance (52.06%), followed by other putative secreted peptides (16.23%), SVEPs (4.70%), immunity-related peptides (3.78%), odorant-binding proteins (3.72%), Yellow-related proteins (3.55%), amylases (3.33%), and cathepsins (2.44%).

As shown in Fig. 1, transcript abundance varied widely across the identified secretory families. SVEP proteins are known vasodilators in black flies, previously described in *S. vittatum* and *S. guianense* as salivary components associated with blood-feeding. In the *S. pertinax* sialotranscriptome, SVEP transcripts were among the most represented functionally defined families, accounting for 4.70% of the total FPKM-based relative abundance of the classified predicted secreted-protein dataset. In addition to these classical hematophagy-related families, 16.23% of the FPKM-based relative abundance corresponded to other putative proteins not directly associated with vascular or hemostatic modulation (grouped as “Other putative secreted peptides”). This heterogeneous category mainly comprised predicted or hypothetical secreted proteins, including several sequences related to hypothetical secreted proteins or secreted peptide precursors from *S. vittatum* and other putative salivary secreted peptides from hematophagous dipterans. Because many of these sequences lacked specific functional annotation, they were retained as a separate group rather than assigned to defined salivary protein families.

### Functional classification and phylogenetic analysis of selected *S. pertinax* families

To explore the evolutionary relationships of the salivary proteins identified in *S. pertinax*, phylogenetic analyses were performed using homologous sequences from other simuliids and selected non-simuliid taxa. While the sialotranscriptome comprises a wide array of functional groups, our phylogenetic reconstructions specifically prioritized selected protein families associated with blood-feeding adaptations or direct modulation of host hemostasis. This targeted comparison allowed for the identification of lineage-associated diversification as well conserved evolutionary relationships, providing a comparative evolutionary framework for interpreting the molecular adaptations underlying hematophagy in this species. The main functional families selected for detailed investigation are described below.

### Kunitz-domain protease inhibitors

Among the protease inhibitor families identified in the predicted secreted repertoire of *S. pertinax*, Kunitz-domain proteins are particularly relevant because several members have been functionally associated with hematophagy in black flies. These small proteins contain a Kunitz domain typically stabilized by three disulfide bonds and act mainly as reversible serine protease inhibitors through an exposed reactive-site loop. In black fly saliva, Kunitz proteins can interfere with host coagulation and inflammatory responses, as demonstrated for Simukunin from *S. vittatum* and *Guianensin* from *S. guianense* [25, 26].

**Fig. 2:**
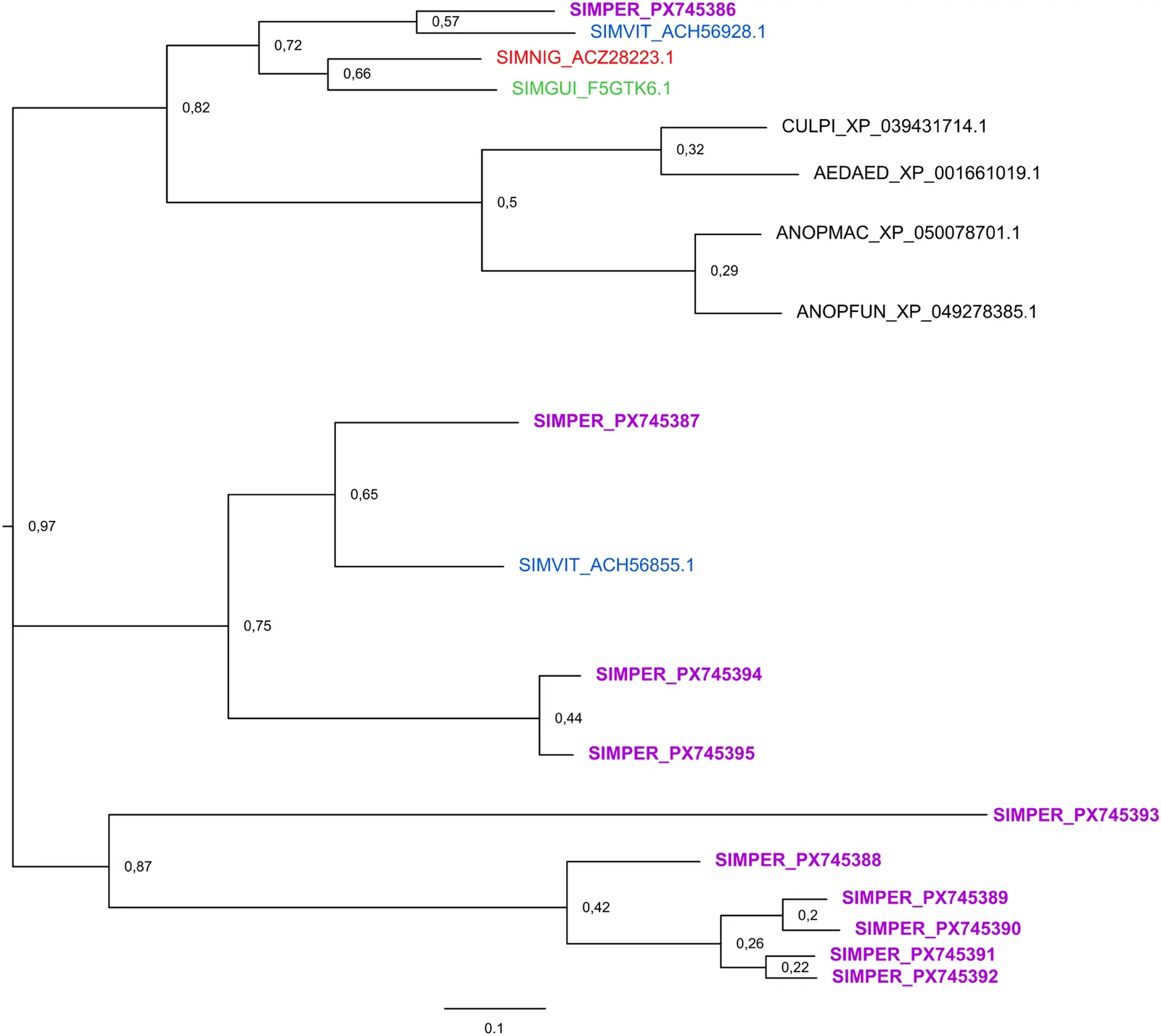
Phylogenetic analysis of sequences from the Kunitz family. The phylogeny was inferred from an amino acid sequence alignment using the Neighbor-Joining method implemented in MEGA X. The robustness of the inferred topology was evaluated using 10,000 bootstrap replicates. Sequences are identified by the first three letters of the genus and species names, followed by their respective NCBI accession numbers. Sequences from *S. pertinax* are highlighted in purple; homologs from *S. vittatum*, *S. guianense*, and *S. nigrimanum* are shown in blue, green, and red, respectively, whereas Culicidae sequences are shown in black. Values at the internal nodes represent bootstrap support expressed as proportions. The scale bar indicates the number of amino acid substitutions per site

Ten S*. pertinax* sequences encoding predicted secreted Kunitz-domain proteins were included in a phylogenetic analysis with homologs from other *Simulium* species and selected Culicidae representatives (Fig. 2). The *S. pertinax* sequences were distributed across three principal regions of the tree rather than forming a single compact species-specific cluster.

SIMPER_PX745386 grouped with the functionally characterized *S. vittatum* Kunitz inhibitor Simukunin (SIMVIT_ACH56928.1), although support for this pair was moderate (0.57). These sequences occurred within a broader simuliid assemblage that also contained the *S. nigrimanum* homolog SIMNIG_ACZ28223.1 and Guianensin from *S. guianense* (SIMGUI_F5GTK6.1). A second *S. pertinax* sequence, SIMPER_PX745387, was associated with the *S. vittatum* protein SIMVIT_ACH56855.1 (SV-170; support 0.65), within a lineage that also included the closely related SIMPER_PX745394 and SIMPER_PX745395 sequences. In contrast, SIMPER_PX745393 and SIMPER_PX745388–SIMPER_PX745392 constituted a *S. pertinax*-restricted lineage supported by a value of 0.87. Within this lineage, SIMPER_PX745393 displayed a comparatively long terminal branch, whereas SIMPER_PX745388–SIMPER_PX745392 formed a more compact subgroup whose internal relationships were weakly resolved.

Overall, the topology reveals both evolutionary relationships with Kunitz homologs from other simuliids and species-associated diversification within the *S. pertinax* repertoire. The association of some sequences with Kunitz proteins from other black fly species is consistent with shared ancestry within the family, whereas the *S. pertinax*-enriched lineages are compatible with lineage-specific duplication followed by sequence divergence. Nevertheless, the low to moderate support of several internal nodes limits conclusions regarding the precise order of these diversification events.

### Kazal-type protease inhibitors

Sequences homologous to the Kazal family of serine protease inhibitors were identified in *S. pertinax* (designated here by the prefix SIMPER). Kazal inhibitors are widely distributed in vertebrates and invertebrates [27–29] and may act as inhibitors of thrombin and other coagulation-related serine proteases, contributing to the inhibition of clot formation during blood feeding [30].

**Fig. 3:**
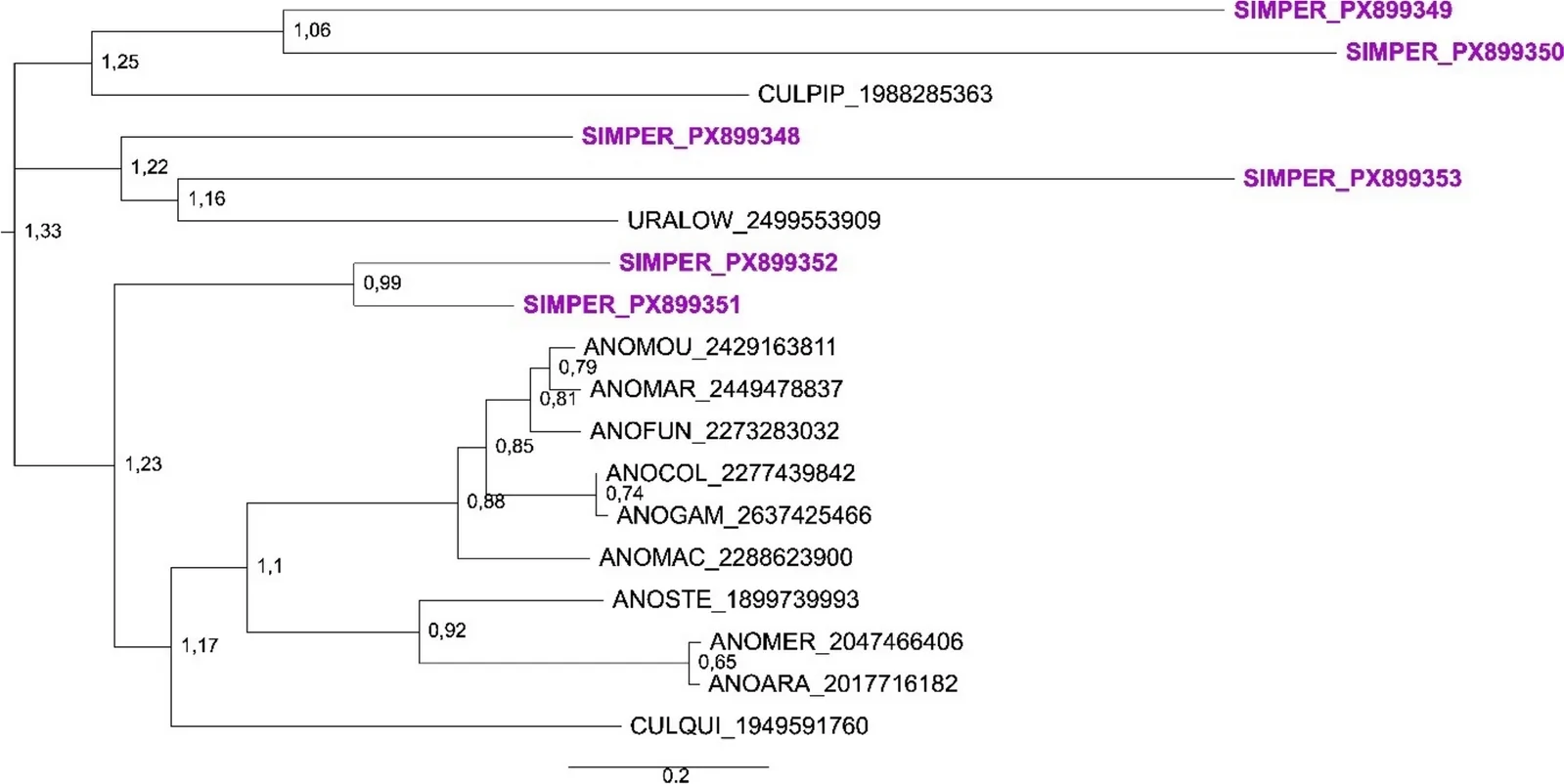
Phylogenetic analysis of sequences from the Kazal family. Phylogeny inferred from the alignment of the Kazal sequences using the MEGAX software after 10,000 bootstraps with the Neighbor-Joining algorithm. The sequences are identified by the first three letters of the genus name followed by the first three letters of the species name (e.g., SIMPER for *S. pertinax*), along with their respective NCBI accession numbers. Sequences from *S. pertinax* are highlighted in purple. Bootstrap values are shown at the nodes. The scale bar represents the number of substitutions per site

Sequence identity analysis (Fig. S4) indicated that *S. pertinax* Kazal sequences share 32–42% identity with *Culicidae* homologs, with the highest similarity observed with *Uranotaenia lowii*. Phylogenetic analysis (Fig. 3) showed that the *S. pertinax* sequences are distributed across multiple branches rather than clustering into a single monophyletic clade separated from mosquito sequences, suggesting diversification of Kazal-related sequences within the *S. pertinax* repertoire. Internal branching among SIMPER sequences indicates the presence of multiple related sequences, compatible with possible gene duplication and subsequent diversification. Closely related SIMPER pairs can be observed, including SIMPER_PX899349/SIMPER_PX899350 and SIMPER_PX899351/SIMPER_PX899352, whereas SIMPER_PX899348 and SIMPER_PX899353 occupy separate branches within the tree. Overall, the topology supports diversification of Kazal-related sequences in *S. pertinax*, but not a strict phylogenetic separation between *Simulium* and *Culicidae* sequences.

### Serpin

Several transcripts in the *S. pertinax* sialotranscriptome were annotated as members of the Serpin family (serine protease inhibitor), known to regulate coagulation, inflammation, and immune responses [31].

**Fig. 4:**
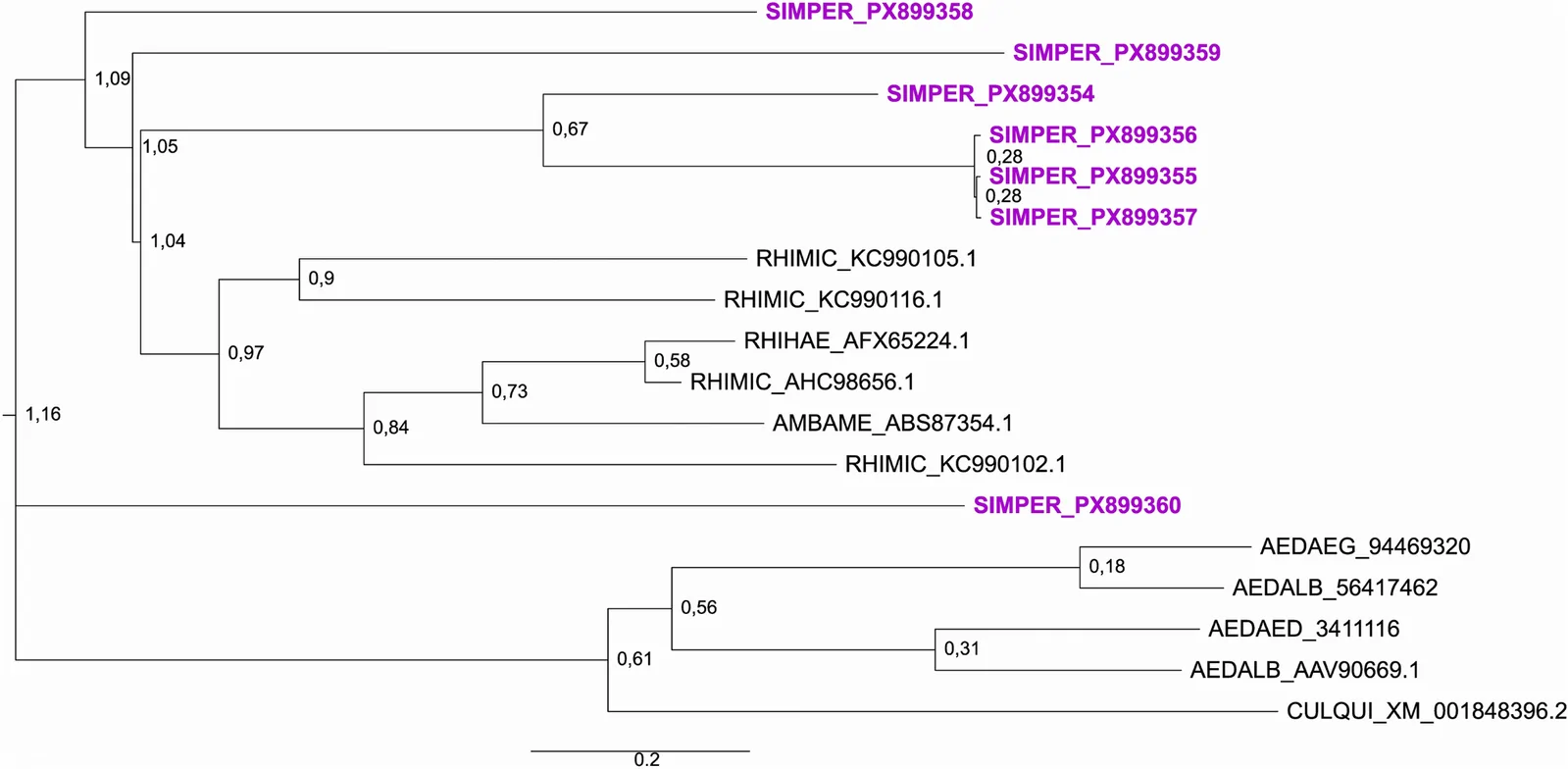
Phylogenetic analysis of sequences from the Serpin family*.* Phylogeny inferred from the alignment of the Serpin sequences using the MEGAX software with 10,000 bootstraps replicates and the Neighbor-Joining algorithm. Sequences are identified by the first three letters of the genus name, followed by the first three letters of the species name, along with their respective NCBI accession numbers. Sequences from *S. pertinax* are highlighted in purple. Bootstrap values are shown at the nodes. The scale bar represents the number of substitutions per site

Phylogenetic analysis (Fig. 4) demonstrated that most *S. pertinax* Serpins group within a SIMPER-enriched cluster separated from the Aedes/Culex mosquito homologs included in the analysis, indicating diversification of Serpin-related sequences within the *S. pertinax* repertoire. Within this SIMPER-enriched group, several transcripts grouped into closely related subclusters, suggesting possible gene duplication and diversification within the species. SIMPER_PX899360 appeared as a divergent *S. pertinax* Serpin positioned outside the main SIMPER cluster, indicating that the *S. pertinax* Serpin repertoire may include more than one divergent Serpin lineage.

Sequence comparison with characterized mosquito Serpins (Fig. S5) revealed moderate identity (~ 60%) with *Aedes aegypti* (AEDAEG_94469320), a factor Xa (fXa) inhibitor [32, 33], and its *Aedes albopictus* homolog (AEDALB_56417462). Although partial identity with characterized mosquito Serpins suggests possible conservation of Serpin-related inhibitory features, the divergence observed among *S. pertinax* sequences indicates lineage-associated diversification within its salivary Serpin repertoire.

### Apyrase

Apyrases are ectonucleotidases commonly found in the saliva of blood-feeding arthropods and are involved in preventing platelet aggregation during blood feeding by hydrolyzing ATP and ADP [2]. In this study, five putative salivary Apyrase sequences were detected in the *S. pertinax* sialotranscriptome, each encoding a single Apyrase domain.

**Fig. 5:**
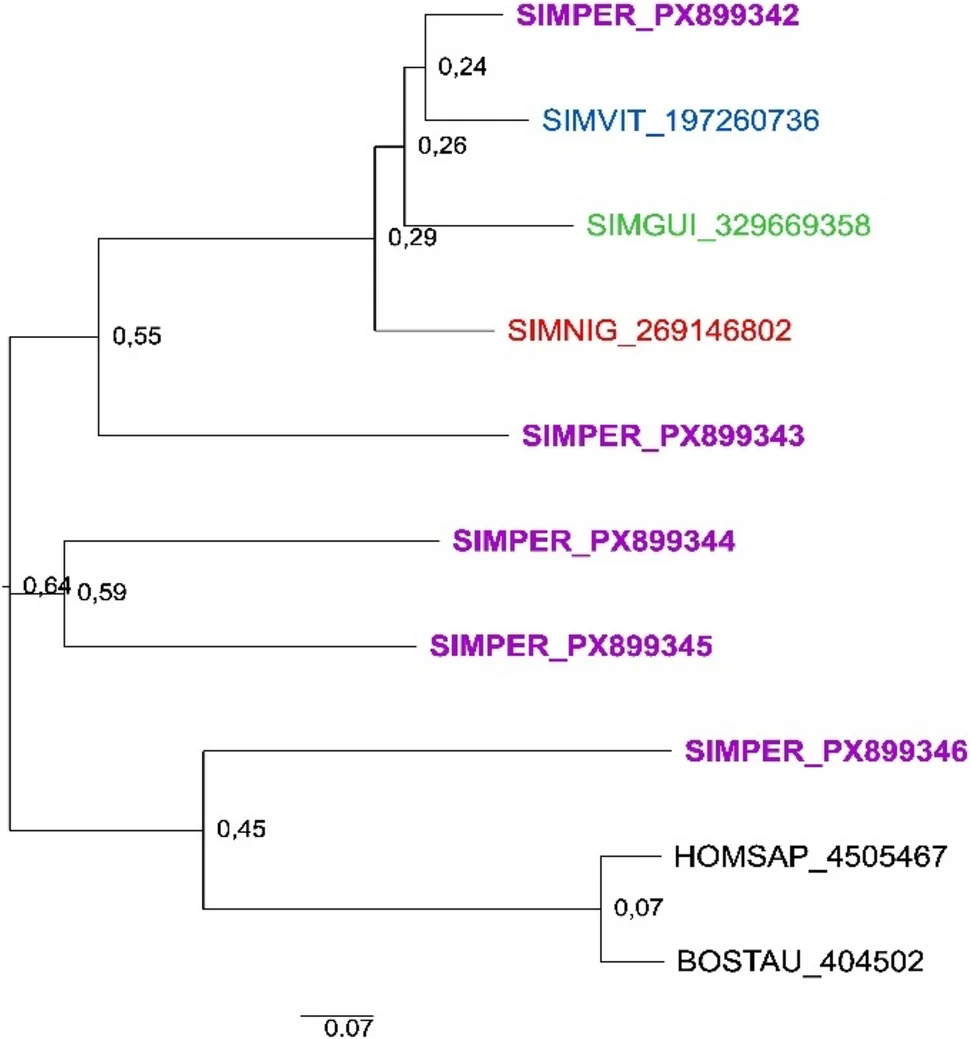
Phylogenetic analysis of sequences from the Apyrase family. Phylogeny inferred from the alignment of the Apyrase sequences using the MEGAX software with 10,000 bootstraps with the Neighbor-Joining algorithm. Sequences are identified by the first three letters of the genus name, followed by the first three letters of the species name, along with their respective NCBI accession numbers. Sequences from *S. pertinax* are highlighted in purple; homologs from *S. vittatum*, *S. guianense*, and *S. nigrimanum* are shown in blue, green, and red, respectively. Bootstrap values are shown at the nodes. The scale bar represents the number of substitutions per site

Alignment with homologs from other black flies and vertebrates (Fig. S6), including *Homo sapiens* and *Bos taurus*, revealed the absence of a C-terminal glycosylphosphatidylinositol (GPI) anchor site indicating that these enzymes exist in a secreted form. This observation is consistent with the secreted patterns reported in *S. guianense*, *S. vittatum*, and *S. nigrimanum* [10, 11, 22]. Furthermore, phylogenetic analysis showed that *S. pertinax* Apyrases are distributed across distinct branches rather than forming a single species-specific clade (Fig. 5). SIMPER_PX899342 clustered with Apyrases from other *Simulium* species, suggesting a conserved relationship among these homologs, whereas SIMPER_PX899343, SIMPER_PX899344, SIMPER_PX899345, and SIMPER_PX899346 occupied separate branches. In particular, SIMPER_PX899344 and SIMPER_PX899345 formed a closely related pair, while SIMPER_PX899346 was positioned closer to the vertebrate Apyrase homologs included in the analysis. This topology indicates diversification of the Apyrase repertoire in *S. pertinax*, including both conserved and more divergent members.

### SVEP vasodilator family

**Fig. 6:**
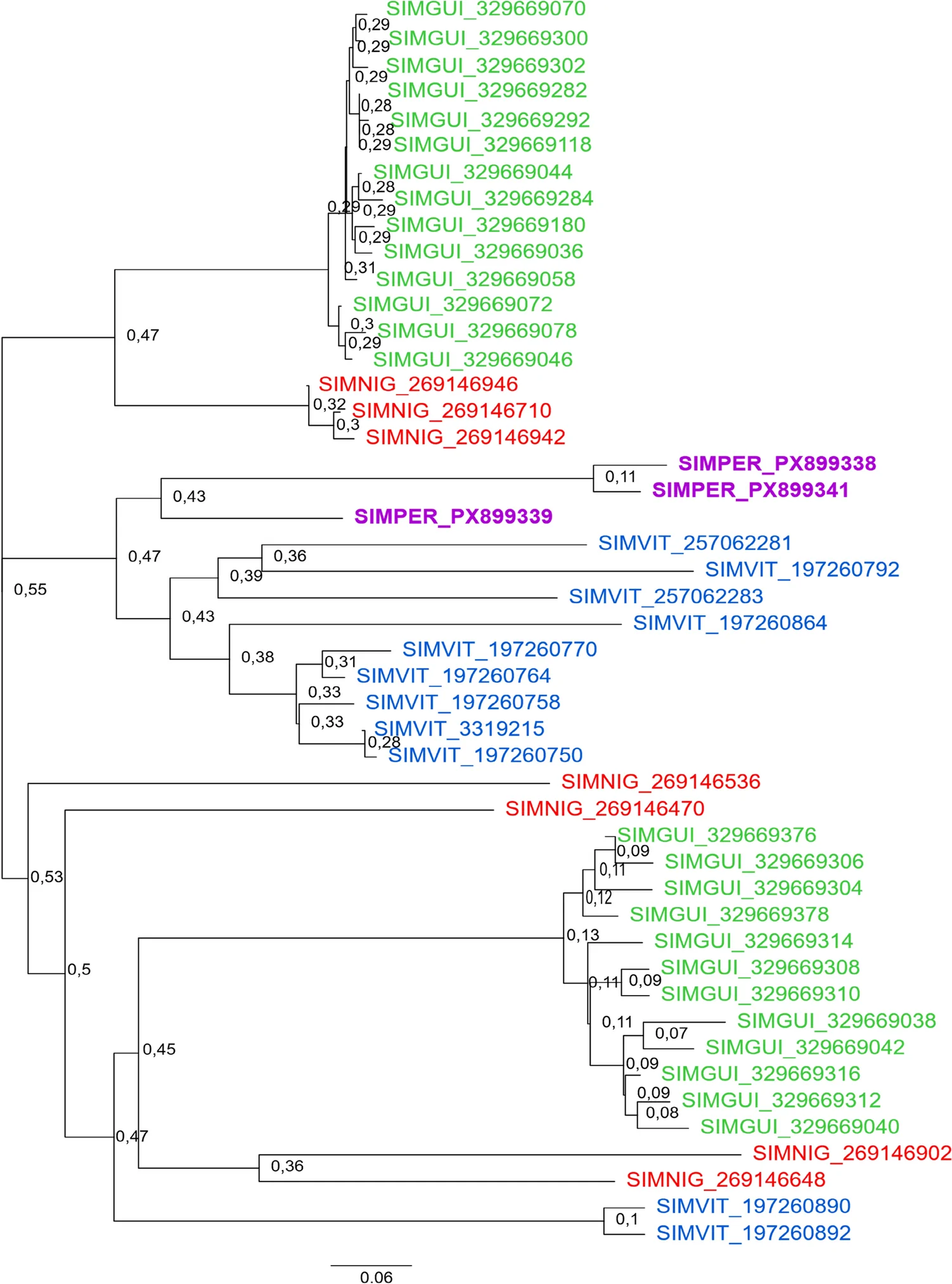
Phylogenetic analysis of sequences from the SVEP family. The phylogeny was inferred from the alignment of SVEP-related amino acid sequences using the Neighbor-Joining method implemented in MEGA X. The robustness of the inferred topology was evaluated using 10,000 bootstrap replicates. Sequences are identified by the first three letters of the genus and species names, followed by their respective NCBI accession numbers. Sequences from *S. pertinax* are highlighted in purple; homologs from *S. vittatum*, *S. guianense*, and *S. nigrimanum* are shown in blue, green, and red, respectively. Numerical values shown along the branches represent branch lengths, and the scale bar indicates the number of amino acid substitutions per site

The *S. pertinax* sialotranscriptome revealed three complete SVEP homologs sharing 30–65% amino acid identity with sequences from other *Simulium* species (Fig. S7). Sequence alignment revealed similar protein lengths but limited amino acid conservation. Phylogenetic analysis showed that the three *S. pertinax* sequences formed a distinct cluster within the simuliid SVEP tree (Fig. 6). Within this cluster, SIMPER_PX899338 and SIMPER_PX899341 formed the most closely related pair, whereas SIMPER_PX899339 occupied a more divergent position. The *S. pertinax* cluster was associated with a group of SVEP homologs from *S. vittatum*, while sequences from *S. guianense* and *S. nigrimanum* were distributed across other regions of the tree. This topology supports sequence diversification within the *S. pertinax* SVEP repertoire and broader evolutionary diversification of the family across the genus *Simulium*. The relationship between the *S. pertinax* and *S. vittatum* sequences is consistent with their shared ancestry within the SVEP family and may indicate that at least some structural or functional properties have been retained during their diversification.

The occurrence of multiple SVEP subgroups within *Simulium* suggests lineage-associated expansion and extensive sequence diversification of this vasodilatory protein family in black flies. The presence of several species-enriched groups, together with the distribution of *S. guianense*, *S. nigrimanum*, and *S. vittatum* homologs across different regions of the tree, is consistent with evolutionary remodeling of the SVEP repertoire among simuliid lineages. Within *S. pertinax*, the close relationship between SIMPER_PX899338 and SIMPER_PX899341, together with the more divergent position of SIMPER_PX899339, indicates that diversification also occurred within the sampled species-associated repertoire. This pattern is consistent with the limited amino acid conservation observed in the alignment and may reflect accelerated sequence evolution following the diversification of SVEP-related sequences in black flies. The apparent restriction of SVEP homologs to *Simulium* species further supports the hypothesis that this family represents a lineage-specific component of black fly saliva.

Overall, the phylogenetic analyses of multiple salivary protein families revealed lineage-associated diversification in *S. pertinax*, with several transcripts distributed across distinct branches within the genus *Simulium*, highlighting the evolutionary diversification of salivary components involved in blood feeding. Among the families analyzed, SVEP homologs showed one of the clearest patterns of diversification, combining species-associated clustering with extensive sequence divergence across the genus, and were therefore examined in greater detail.

### SpVaP cloning and expression

Although SVEP has been previously described as a vasodilator, detailed functional studies using recombinant protein are limited to only one work [3, 20]. Based on its high abundance in the sialotranscriptome and its putative role in blood-feeding, a novel member of the SVEP family was selected for investigation. Given its high expression in the *S. pertinax* transcriptome, its apparent restriction to black flies based on currently available sequences, and its potential role in host hemodynamic modulation, a highly represented SVEP homolog was selected for cloning, heterologous expression, and functional characterization. In view of its origin and demonstrated biological activity, this sequence was named *S. pertinax* Vasoactive Protein (SpVaP) and was selected for cloning and functional characterization. The *S. pertinax* SpVaP transcript SIMPER PX899338 encodes a 152-residue precursor with a predicted 20-residue signal peptide, yielding a mature protein of 132 amino acids with a predicted molecular mass of ~ 15 kDa (~ 16 kDa for the recombinant form carrying the C-terminal six-histidine tag), theoretical pI of 8.78, two cysteine residues, and no predicted N-glycosylation site.

**Fig. 7:**
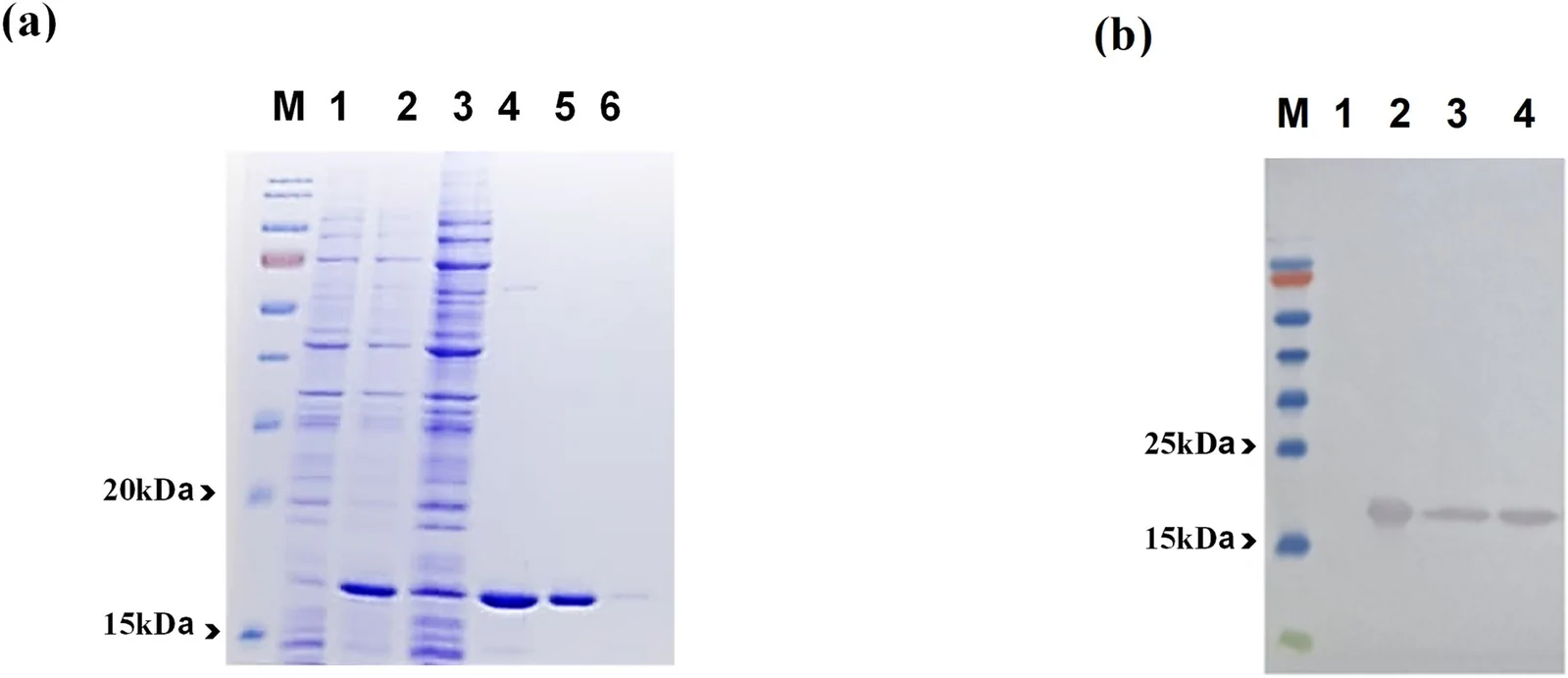
Expression, purification and dialysis of rSpVaP in the pET28a (+) vector. **a** 13.5% SDS-PAGE gel. The numbers on the left indicate the molecular weight marker positions (M) in kDa. The numbers above the lanes correspond to: 1 and 2—*BL21star™(DE3)* extract before and after IPTG induction, respectively; 3—soluble fraction of the cell lysate; 4—purified rSpVaP; 5 and 6—soluble and insoluble fractions of protein after dialysis, respectively. **b** Western blot analysis using anti-HisTag antibody. 1 and 2—*BL21star™(DE3)* extract before and after IPTG induction, respectively; 3—purified rSpVaP; 4—soluble rSpVaP after dialysis

SDS-PAGE analysis (Fig. 7a) confirmed successful IPTG-induced expression of an approximately 15 kDa protein in soluble form, which remained stable after purification and dialysis. Western blotting with anti-HisTag antibody (Fig. 7b) confirmed the presence of 6Histag int the protein and mass spectrometry analysis (Fig. S8) confirmed the identity of the recombinant form SpVaP (rSpVaP). Peptide mapping covered 67% of the mature protein sequence, supporting correct expression and identification. The molecular mass was consistent with the expected 15 kDa. The material used to generate Fig. S8 are provided as Supplementary Data. S9.

### Circular dichroism and structural model

**Fig. 8:**
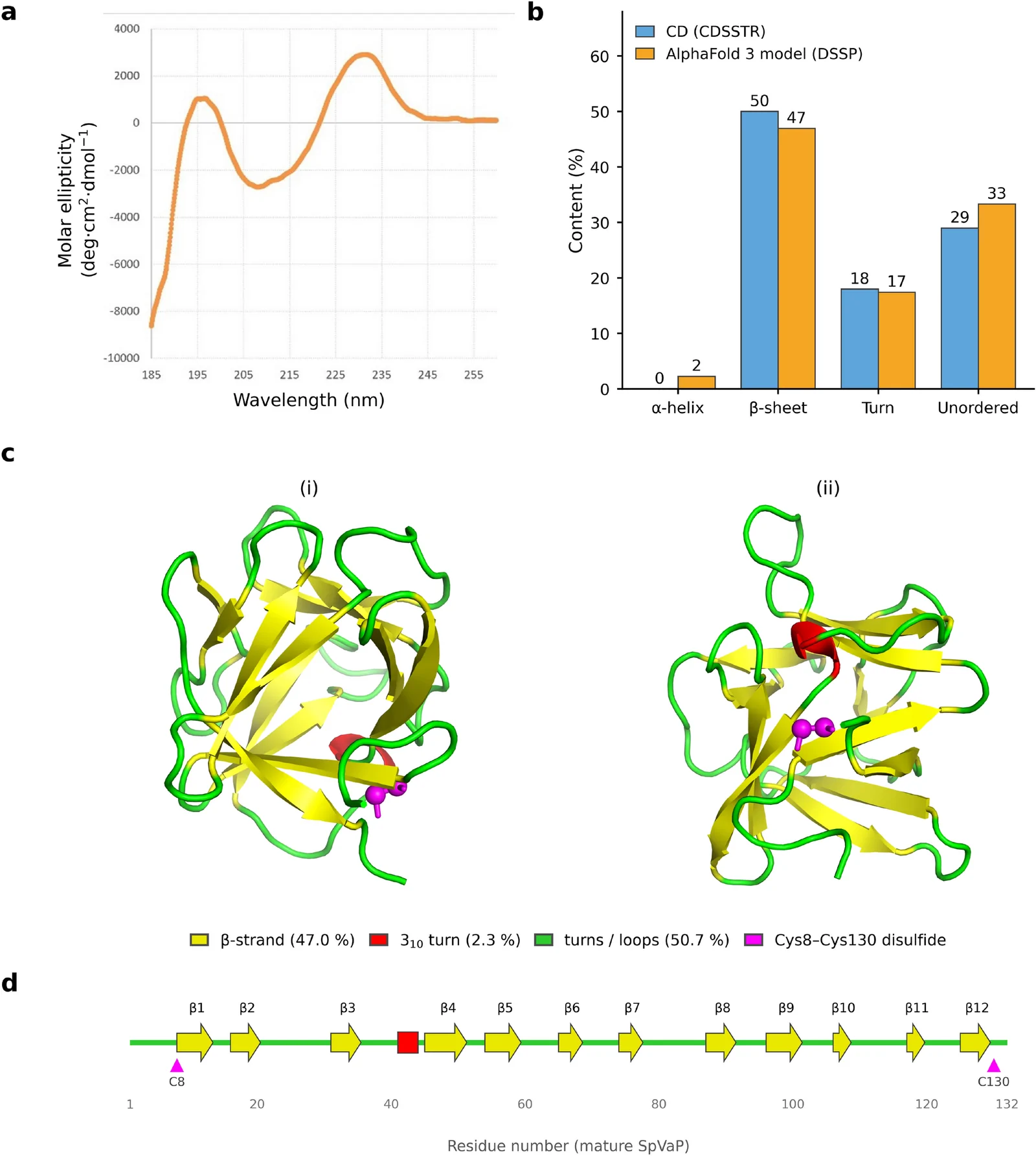
Circular dichroism analysis and structural model of rSpVaP. **a** Far-UV circular dichroism (CD) spectrum of rSpVaP recorded from 185 to 260 nm. **b** Secondary-structure content estimated from deconvolution of the CD spectrum (DichroWeb, CDSSTR algorithm; blue) compared with the content assigned to the AlphaFold 3 model by DSSP (Define Secondary Structure of Proteins; orange). **c** Model of the mature rSpVaP (residues Asp1–Thr132) predicted with AlphaFold 3, shown in two orientations, (i) and (ii), related by a rotation of approximately 160°: β-strands in yellow, the single 3_10_ turn (Asn41–Gln43) in red, turns and loops in green, and the Cys8–Cys130 disulfide bond as magenta sticks and spheres. The protein adopts a β-trefoil fold. **d** Secondary-structure topology along the mature sequence, with the twelve β-strands numbered and the disulfide-bonded cysteines indicated (magenta triangles). Molecular graphics were prepared with PyMOL; graphs were plotted in Python

To gain insight into the secondary structure of rSpVaP, we recorded a far-UV circular dichroism (CD) spectrum (Fig. 8a). The spectrum showed no minimum at 222 nm, indicating the absence of α-helical structure. Deconvolution estimated a composition of ~ 50 % β-sheet, ~ 29 % unordered and ~ 18 % turn, with no α-helix (Fig. 8b, blue bars). These values are in close agreement with the composition assigned by DSSP to the AlphaFold 3 model of the mature protein (47 % β-strand, 33 % unordered, 17 % turn; Fig. 8b, orange bars). The model consists of twelve β-strands arranged in a β-trefoil fold, with a disulfide bond between Cys8 and Cys130 (Fig. 8c, d); the 2 % helical content assigned to it corresponds to a single three-residue 3₁₀ turn.

### Intravital microscopy analysis of rSpVaP

**Fig. 9:**
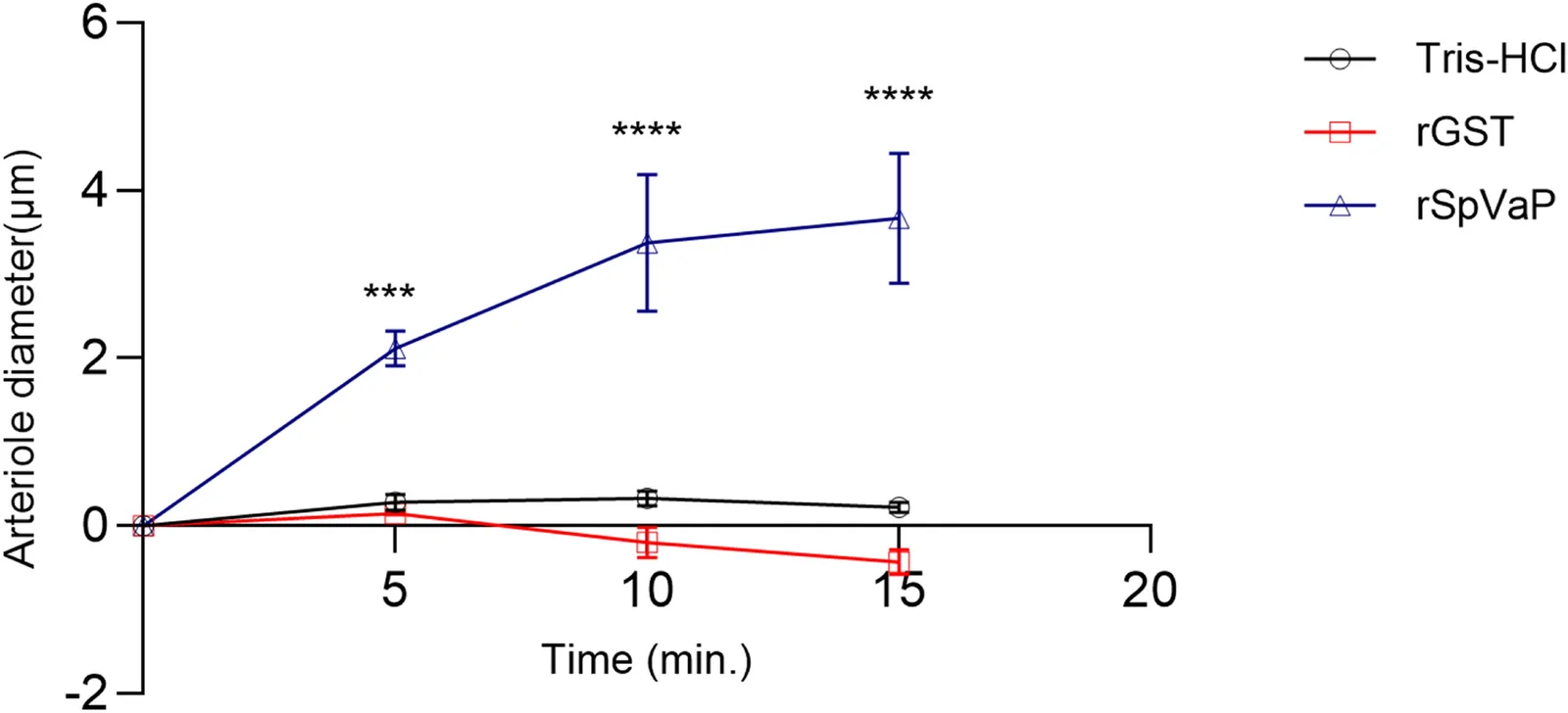
Intravital microscopy of the mouse cremaster muscle after topical application of rSpVaP (8.6 µM), rGST (8.6 µM), or Tris–HCl buffer (30 µL). Changes in arteriolar diameter (Δµm) were quantified at 0, 5, 10, and 15 min. Data are mean ± SEM (n = 7 mice/group). Statistics: two-way repeated-measures ANOVA (treatment × time) followed by Šídák’s multiple-comparisons test comparing rSpVaP vs Tris–HCl and rSpVaP vs rGST at the corresponding time points. ***p = 0.0005, ****p < 0.0001 (as indicated). Two independent experiments were performed

Given that sequence homologies and structural data strongly suggested that this novel protein family possesses potent vasoactive properties, we next evaluated the functional vasodilatory activity of rSpVaP *in vivo*. For this purpose, intravital microscopy of the mouse cremaster muscle was performed. Mice received topical application of 30 µL rSpVaP (8.6 µM), while 30 µL of rGST (8.6 µM), serving as an unrelated protein control, and 30 µL of Tris-HCl buffer served as control too. Arteriolar diameter was measured at 5, 10, and 15 min after application (Fig. 9).

rSpVaP induced significant vasodilation beginning at 5 min, with a clear time-dependent increase compared to controls. Neither rGST nor buffer produced significant changes in vessel diameter. As rGST was expressed and purified under identical conditions, these results indicate that the vasodilatory response is specifically attributable to rSpVaP activity. Given the marked vasodilatory response induced by rSpVaP, its ability to promote local inflammatory changes was subsequently investigated by assessing paw edema formation.

### Edematogenic activity

**Fig. 10:**
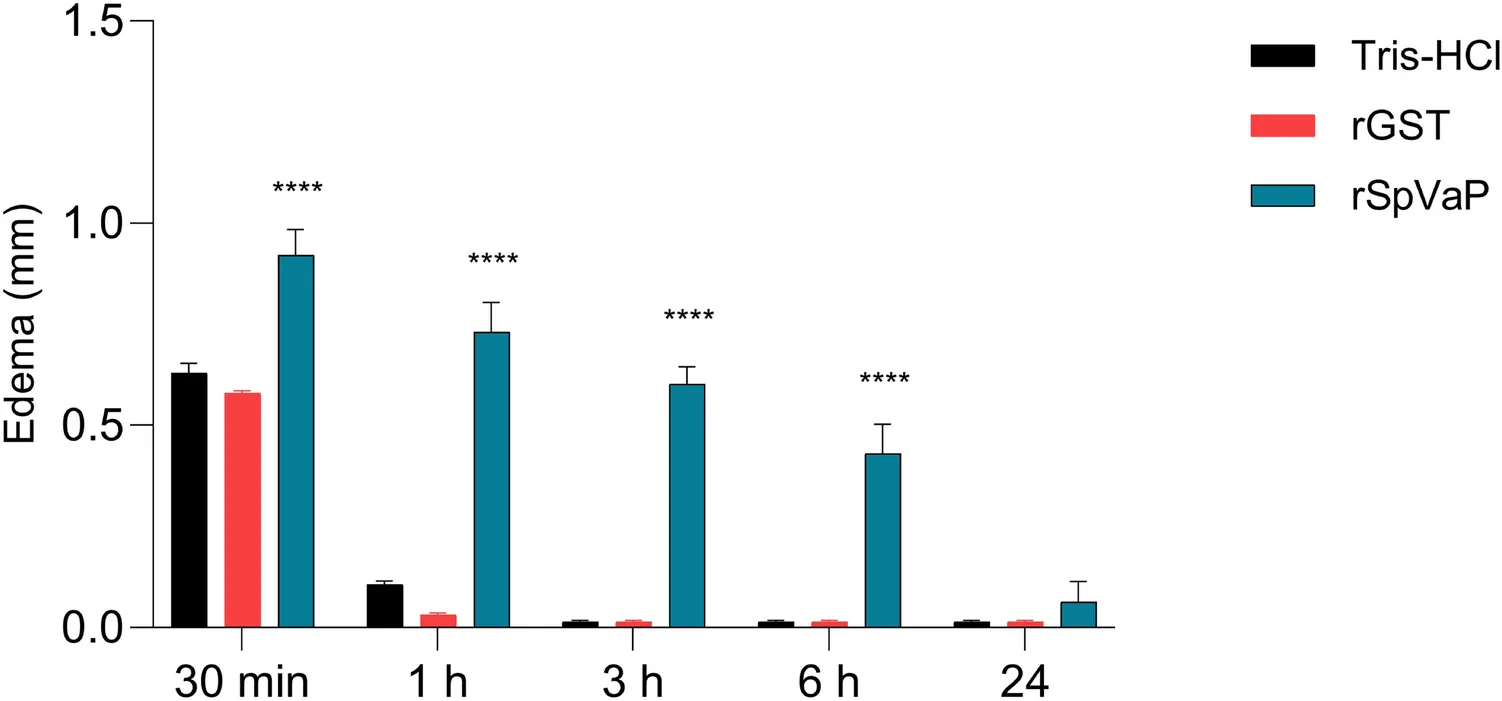
rSpVaP induces paw edema in mice. Paw thickness (edema) was measured after administration of 30 µL rSpVaP (12 µM) or rGST (12 µM); Tris–HCl buffer served as vehicle control. Measurements were taken at 30 min, 1 h, 3 h, 6 h, and 24 h after application. Data are presented as mean ± SEM (n = 5 mice/group). Statistics: two-way repeated-measures ANOVA (treatment × time) followed by Šídák’s multiple-comparisons test comparing rSpVaP vs Tris–HCl and rSpVaP vs rGST at the corresponding time points. ****p < 0.0001 (as indicated). Two independent experiments were performed

Because potent microvascular vasodilation is frequently accompanied by increased vascular permeability and plasma extravasation, we next investigated whether rSpVaP could induce local inflammatory tissue swelling. For this purpose, an edematogenic assay was performed by injecting the protein (12 µM, 30 µL) into mouse hind paws (Fig. 10).

rSpVaP induced a significant acute and sustained increase in paw thickness compared to Tris/HCl and rGST controls. Edema was detectable at 30 min and remained significantly elevated at 1, 3, and 6 h, returning to baseline at 24 h. No significant changes were observed in the rGST group at any time point, confirming that the edematogenic response was specific to rSpVaP.

## Discussion

This study provides the first integrated view of the *S. pertinax* salivary-gland transcriptome and links its predicted secretory repertoire to evolutionary and functional evidence. The coexistence of digestive enzymes, protease inhibitors, antiplatelet proteins, immune-related peptides, and vasoactive factors is consistent with the multifunctional nature of black fly saliva and broadly parallels other *Simulium* sialomes [10, 11, 22]. Because abundance was estimated from a single pooled female sample, however, the FPKM values should be interpreted as within-dataset family-level contributions, rather than as differential expression or female-specific enrichment.

The phylogenies of the selected families revealed a recurring combination of interspecific relationships and *S. pertinax*-enriched branches, rather than strict separation by species. This pattern is compatible with repeated duplication and divergence of salivary genes, processes that can expand the molecular repertoire available for host interaction. Nevertheless, the low or moderate support of several internal nodes limits inference of the precise sequence of these events. The trees therefore support repertoire diversification, but do not by themselves establish orthology, protease specificity, or physiological function.

Kunitz-domain proteins provide the strongest functional context for interpreting the protease inhibitors identified here. Simukunin from *S. vittatum*, Guianensin from *S. guianense*, and Sibanin from *S. bannaense* have experimentally demonstrated antihemostatic and/or anti-inflammatory activities, with Sibanin also affecting platelet function and pain signaling [25, 26, 34]. The association of SIMPER_PX745386 with Simukunin identifies this sequence as a relevant candidate for functional investigation. Conversely, the relationship of SIMPER_PX745387 with SV-170, which did not inhibit plasma coagulation under the conditions tested, reinforces that phylogenetic proximity within the Kunitz family is insufficient to predict activity [25]. More broadly, the coexistence of Kunitz, Kazal, and Serpin inhibitors suggests a layered salivary strategy for targeting host proteases. Because Kazal and Serpin transcripts were not reported in earlier black fly sialotranscriptomes [10, 11, 22], their detection may reflect biological differences among species, sampling, or annotation sensitivity. Their predicted functions and possible redundancy remain to be established experimentally.

The Apyrase repertoire extends this antihemostatic potential from coagulation proteases to platelet activation. The predicted secretion of the *S. pertinax* Apyrases and their evolutionary relationships with other simuliid homologs are consistent with the established role of salivary Apyrases in hydrolyzing extracellular ATP and ADP during blood feeding [2, 4, 10]. At the same time, their sequence diversification may reflect adaptation of salivary repertoires to different feeding contexts. Because enzymatic activity was not measured here, this interpretation remains a functional hypothesis derived from domain architecture and homology.

SVEP represents a particularly distinctive component of this repertoire because currently available homologs are associated with black flies and the family includes the vasoactive protein characterized in *S. vittatum* [3, 11, 35]. The relationship of the *S. pertinax* sequences with *S. vittatum* homologs supports shared ancestry and raises the possibility that vasoactive properties have been retained in at least part of the family; however, the extensive sequence divergence indicates that functional conservation cannot be assumed for every homolog. Selection of SIMPER_PX899338 for recombinant characterization was therefore supported by its complete coding sequence, high transcriptomic representation, and relationship to a previously characterized vasoactive family. The designation SpVaP reflects the taxonomic origin of the protein and the vascular activity demonstrated in the present study.

The agreement between circular-dichroism estimates and the AlphaFold model supports a folded, predominantly beta-sheet structure for rSpVaP, but does not identify the molecular mechanism underlying its activity. *In vivo*, rSpVaP induced arteriolar dilation and paw edema relative to both vehicle and recombinant-protein controls. These effects are biologically compatible with facilitation of blood feeding because increased local perfusion and changes in tissue-fluid dynamics could maintain access to blood at the bite site. The edema response also suggests that the effect of rSpVaP is not limited to vascular smooth-muscle relaxation. However, edema alone does not demonstrate increased endothelial permeability or direct inflammatory signaling, and neither endothelial-leukocyte interactions nor inflammatory mediators were measured. The assays were also performed at a single concentration, precluding conclusions about potency, threshold concentration, or dose dependence.

These limitations complement those of the transcriptomic design: the dataset derives from a single pooled female sample, the functions of most sequences remain predictions, and functional validation was restricted to one SVEP-related protein. Replicated comparisons of male and female salivary glands, biochemical characterization of the Kunitz, Kazal, Serpin, and Apyrase candidates, and concentration-response and mechanistic studies of rSpVaP will be needed to define the contribution of these molecules to blood feeding. Even with these constraints, the study establishes a reference transcriptomic resource for *S. pertinax* and provides direct evidence that one member of its SVEP repertoire is biologically active in the host microvasculature.

## Conclusion

This study provides the first characterization of the *S. pertinax* sialotranscriptome, offering important insights into the molecular repertoire of this poorly explored South American black fly. Our analysis mapped the secretory repertoire, revealing a complex array of functional families including digestive enzymes, serine protease inhibitors, antimicrobial peptides, and proteins involved in host hemostatic modulation. Phylogenetic analyses revealed conserved evolutionary relationships as well as lineage-associated sequence diversification within selected salivary protein families of *S. pertinax*, highlighting the evolutionary diversity of its salivary repertoire without implying strict species-specific separation for all families analyzed. As a functional proof-of-concept of this salivary complexity, we successfully expressed and characterized rSpVaP, confirming through *in vivo* assays marked vasodilatory activity and edematogenic effect during the acute phase of the response. Overall, this work demonstrates the value of integrating transcriptomic screening with molecular cloning and functional validation. The characterization of rSpVaP represents an initial step toward elucidating the biological roles of *S. pertinax* salivary proteins and establishes a framework for future studies aimed at understanding their role in vector–host interactions and the molecular basis of hematophagy.

## Supplementary Information

Below is the link to the electronic supplementary material.

Supplementary file1 (ZIP 384 KB)

Supplementary file2 (TIF 10099 KB)

Supplementary file3 (TIF 7264 KB)

Supplementary file4 (TIF 13573 KB)

Supplementary file5 (TIF 13254 KB)

Supplementary file6 (TIF 14175 KB)

Supplementary file7 (TIF 18874 KB)

Supplementary file8 (TIF 2212 KB)

Supplementary file9 (ZIP 3609 KB)

Supplementary file10 (DOCX 14 KB)

## Data Availability

The sequences identified in the sialome of S. pertinax and analyzed phylogenetically were deposited in the NCBI database. They will be made publicly available when the article is accepted for publication.

## Abbreviations

ADP: Adenosine diphosphate
ANOVA: Analysis of variance
ATP: Adenosine triphosphate
BCA: Bicinchoninic acid
bp: Base pairs
CD: Circular dichroism
cDNA: Complementary DNA
DTT: Dithiothreitol
E. coli: Escherichia coli
ESI: Electrospray ionization
FPKM: Fragments per kilobase of transcript per million mapped reads
fXa: Factor Xa
GO: Gene ontology
GPI: Glycosylphosphatidylinositol
rGST: Recombinant glutathione S-transferase
IMAC: Immobilized metal affinity chromatography
IPTG: Isopropyl β-D-1-thiogalactopyranoside
KEGG: Kyoto encyclopedia of genes and genomes
KO: KEGG orthology
KOG: Eukaryotic orthologous groups
LB: Luria–Bertani medium
LC–MS/MS: Liquid chromatography tandem mass spectrometry
MF: Molecular function
MRE: Mean residue ellipticity
mRNA: Messenger RNA
MS/MS: Tandem mass spectrometry
NR: Non-Redundant database
OD600: Optical density at 600 nm
PBS: Phosphate-buffered saline
PFAM: Protein families database
RNA: Ribonucleic acid
Rpm: Revolutions per minute
rSpVaP: Recombinant *S. pertinax* vasoactive protein
rSVEP: Recombinant SVEP
*S. guianense*: *Simulium guianense*
*S. pertinax*: *Simulium pertinax*
*S. vittatum*: *Simulium vittatum*
SD: Standard deviation
SDS-PAGE: Sodium dodecyl sulfate polyacrylamide gel Electrophoresis
SEM: Standard error of the mean
SG: Salivary gland
SpVaP: *S. pertinax* Vasoactive protein
SVEP: *Simulium* Vasodilator erythema protein
UFLC: Ultra-fast liquid chromatography
