# Supplementary material for: Sialotranscriptome of Simulium pertinax and functional characterization of SpVaP: a novel recombinant simuliid-specific vasodilator
Source: Mol Cell Biochem. 2026 Sep 3;481(10):5231–49. doi: 10.1007/s11010-026-05718-4 (PMC13687198; doi:10.1007/s11010-026-05718-4)
Supplement: Supplementary file 1 [file 11010_2026_5718_MOESM10_ESM.docx]

# **Supplementary Information**

**Supplementary Data. S1** Three-dimensional structure of SpVaP

**Fig. S2** Gene Ontology (GO) functional annotation. Distribution of the most representative functional categories across the 15,708 nuclear-enriched transcripts from the *S. pertinax* salivary gland de novo transcriptome assembly. Transcripts are classified under the three major GO domains: Biological Process (blue), Cellular Component (red), and Molecular Function (green). The X-axis displays the specific GO terms; the Y-axis indicates the absolute number of transcripts assigned to each category

**Fig. S3** ClustalW alignment of the Kunitz family identified in the sialotranscriptome of *S. pertinax,* together with homologous proteins from various culicids available in the NCBI non-redundant protein database. The conservation histogram and consensus sequence displayed below the alignment summarize residue conservation among the analyzed sequences

**Fig. S4** ClustalW alignment of the Kazal domain (aa 41–96) identified in the sialotranscriptome of *S. pertinax,* together with homologous proteins from various culicids available in the NCBI non-redundant protein database. Only the conserved domain is shown to improve visualization of the alignment. The conservation histogram and consensus sequence displayed below the alignment summarize residue conservation among the analyzed sequences

**Fig. S5** ClustalW alignment of the Serpin domain (aa 1–370) identified in the sialotranscriptome of *S. pertinax* together with homologous proteins from various culicids available in the NCBI non-redundant protein database. Only the conserved domain is shown to improve visualization of the alignment. The conservation histogram and consensus sequence displayed below the alignment summarize residue conservation among the analyzed sequences

**Fig. S6** ClustalW alignment of the Apyrase domain (aa 338–502) identified in the sialotranscriptome of *S. pertinax* together with homologous proteins from other black flies and from *Homo sapiens* and *Bos taurus*, available in the NCBI non-redundant protein database. Only the conserved domain is shown to improve visualization of the alignment. The conservation histogram and consensus sequence displayed below the alignment summarize residue conservation among the analyzed sequences

**Fig. S7** ClustalW alignment of SpVaP (PX899338) identified in the sialotranscriptome of *S. pertinax* together with homologous proteins from various *Simulium* species available in the NCBI non-redundant protein database. Only the conserved domain is shown to improve visualization of the alignment. The conservation histogram and consensus sequence displayed below the alignment summarize residue conservation among the analyzed sequences

**Fig. S8-** LC–MS/MS peptide mapping of purified rSpVaP. Coverage map of the mature SpVaP sequence (132 residues, corresponding to GenBank accession PX899338 after removal of the 20-residue predicted signal peptide), according to Peaks Studio analysis. Shaded residues indicate the regions covered by proteomically matched peptides, shown as blue bars below the sequence; sequence coverage was 67%. The residue marked at position 33 corresponds to an oxidised methionine (+15.99 Da)

**Supplementary Data. S9** Mass spectrometry analysis
