# Supplementary material for: Sialotranscriptome of Simulium pertinax and functional characterization of SpVaP: a novel recombinant simuliid-specific vasodilator
Source: Mol Cell Biochem. 2026 Sep 3;481(10):5231–49. doi: 10.1007/s11010-026-05718-4 (PMC13687198; doi:10.1007/s11010-026-05718-4)
Supplement: Supplementary file 3 [file 11010_2026_5718_MOESM9_ESM.zip › SpVap Mass Spec/protein.html]

proteins


Summary

  

# 1. Notes

# 2. Result Statistics

**Figure 1.**
False discovery rate (FDR) curve. X axis is the number of peptide-spectrum matches (PSM) being kept. Y axis is the corresponding FDR.

  


  

**Figure 2.**
PSM score distribution.  **(a)**
Distribution of PEAKS peptide score;  **(b)**
Scatterplot of PEAKS peptide score versus precursor mass error.

**(a)**

**(b)**

**Figure 3.**
De novo result validation. Distribution of residue local confidence:  **(a)**
Residues in de novo sequences validated by confident database peptide assignment;  **(b)**
Residues in "de novo only" sequences.

**(a)**

**(b)**

**Table 1.**
Statistics of data.

|  |  |
| --- | --- |
| # of MS Scans | 1504 |
| # of MS/MS Scans | 1504 |


**Table 2.**
Result filtration parameters.

|  |  |
| --- | --- |
| Peptide -10lgP | ≥15 |
| Protein -10lgP | ≥20 |
| Proteins unique peptides | ≥0 |
| De novo ALC Score | ≥50% |


**Table 3.**
Statistics of filtered result.

|  |  |
| --- | --- |
| Peptide-Spectrum Matches | 45 |
| Peptide Sequences | 28 |
| Protein Groups | 6 |
| Proteins | 441 |
| Proteins (#Unique Peptides) | 2 (>2); 5 (=2); 434 (=1); |
| FDR (Peptide-Spectrum Matches) | 68.9% |
| FDR (Peptide Sequences) | 100.0% |
| FDR (Protein) | 1.4% |
| De Novo Only Spectra | 6 |

**Table 4.**
PTM profile.

| Name | ∆Mass | #PSM | Position || Oxidation | 15.99 | 1 | M |

# 3. Experiment Control

**Figure 4.**
Precursor mass error of peptide-spectrum matches (PSM) in filtered result.  **(a)**
Distribution of precursor mass error in ppm;  **(b)**
Scatterplot of precursor m/z versus precursor mass error in ppm.

**(a)**

**(b)**

**Table 5.**
Number of identified peptides in each sample by the number of missed cleavages

|  |  |  |  |  |  |
| --- | --- | --- | --- | --- | --- |
| Missed Cleavages | 0 | 1 | 2 | 3 | 4+ |
| SVEP-Camila | 21 | 5 | 2 | 0 | 0 |

# 4. Other Information

**Table 6.**
Search parameters.

Search Engine Name: PEAKS 7.0  
Parent Mass Error Tolerance: 0.5 Da  
Fragment Mass Error Tolerance: 0.5 Da  
Precursor Mass Search Type: monoisotopic  
Enzyme: Trypsin  
Max Missed Cleavages: 3  
Non-specific Cleavage: one  
Fixed Modifications:   
  Carbamidomethylation: 57.02  
Variable Modifications:   
  Oxidation (M): 15.99  
Max variable PTM per peptide: 3  
Database: Simuliidae  
Taxon: All  
Searched Entry: 6801  
FDR Estimation: Enabled  
Merge Options: 0.1 min. 0.1 Da  
Precursor Options: corrected  
Charge Options: no correction  
Filter Options: no filter  
Process: true

**Table 7.**
Instrument parameters.

Fractions: SVEP\_Seg1Ev1.mzXML  
Ion Source: ESI(nano-spray)  
Fragmentation Mode: CID, CAD(y and b ions)  
MS Scan Mode: 3D Ion Trap  
MS/MS Scan Mode: 3D Ion Trap

  

Protein List

  

| Protein Group | Protein ID | Accession | -10lgP | Coverage (%) | #Peptides | #Unique | PTM | Avg. Mass | Description |
| --- | --- | --- | --- | --- | --- | --- | --- | --- | --- |
| 1 | 61568 | SpVaP\_mature | 138.48 | 67 | 12 | 12 | Y | 15251 | SpVaP\_mature |
| 1 | 54175 | SVEP | 138.48 | 55 | 12 | 12 | Y | 18072 | S.pertinax |
| 6 | 1879 | tr\|A0A0S1N2H3\|A0A0S1N2H3\_9DIPT | 28.78 | 6 | 2 | 1 | N | 18787 | Cytochrome c oxidase subunit 1 (Fragment) OS=Simulium sp. BOLD:AAA4264 OX=1695073 GN=COI PE=3 SV=1 |
| 5 | 454 | tr\|A0A068FD86\|A0A068FD86\_9DIPT | 25.17 | 5 | 2 | 1 | N | 21875 | Cytochrome c oxidase subunit 1 (Fragment) OS=Simulium arcticum sensu lato CW22\_9 OX=1513956 GN=COI PE=3 SV=1 |
| 5 | 1163 | tr\|A0A0H4SK32\|A0A0H4SK32\_9DIPT | 25.17 | 5 | 2 | 1 | N | 21913 | Cytochrome c oxidase subunit 1 (Fragment) OS=Simulium takaokai OX=1537663 GN=COI PE=3 SV=1 |
| 5 | 1730 | tr\|A0A0C4KQM0\|A0A0C4KQM0\_9DIPT | 25.17 | 5 | 2 | 1 | N | 19469 | Cytochrome c oxidase subunit 1 (Fragment) OS=Simulium verecundum OX=109159 GN=COI PE=3 SV=1 |
| 5 | 1732 | tr\|A0A0C4KJS0\|A0A0C4KJS0\_9DIPT | 25.17 | 5 | 2 | 1 | N | 19508 | Cytochrome c oxidase subunit 1 (Fragment) OS=Simulium bracteatum OX=626788 GN=COI PE=3 SV=1 |
| 5 | 934 | tr\|A0A0C4KIW4\|A0A0C4KIW4\_9DIPT | 25.17 | 5 | 2 | 1 | N | 19943 | Cytochrome c oxidase subunit 1 (Fragment) OS=Simulium tuberosum OX=357430 GN=COI PE=3 SV=1 |
| 5 | 938 | tr\|A0A0C4KIY0\|A0A0C4KIY0\_9DIPT | 25.17 | 5 | 2 | 1 | N | 19943 | Cytochrome c oxidase subunit 1 (Fragment) OS=Simulium verecundum OX=109159 GN=COI PE=3 SV=1 |
| 5 | 955 | tr\|A0A0C4KU88\|A0A0C4KU88\_9DIPT | 25.17 | 5 | 2 | 1 | N | 20270 | Cytochrome c oxidase subunit 1 (Fragment) OS=Simulium sp. BOLD:ACI0635 OX=1494722 GN=COI PE=3 SV=1 |
| 5 | 979 | tr\|A0A0C4KKB9\|A0A0C4KKB9\_9DIPT | 25.17 | 5 | 2 | 1 | N | 20385 | Cytochrome c oxidase subunit 1 (Fragment) OS=Simulium verecundum OX=109159 GN=COI PE=3 SV=1 |
| 5 | 1736 | tr\|A0A0C4KQA3\|A0A0C4KQA3\_9DIPT | 25.17 | 5 | 2 | 1 | N | 19674 | Cytochrome c oxidase subunit 1 (Fragment) OS=Simulium sp. BOLD:ACI0635 OX=1494722 GN=COI PE=3 SV=1 |
| 5 | 944 | tr\|A0A0C4KUC8\|A0A0C4KUC8\_9DIPT | 25.17 | 5 | 2 | 1 | N | 20090 | Cytochrome c oxidase subunit 1 (Fragment) OS=Simulium sp. BOLD:ACI0635 OX=1494722 GN=COI PE=3 SV=1 |
| 5 | 981 | tr\|A0A0C4KVL3\|A0A0C4KVL3\_9DIPT | 25.17 | 5 | 2 | 1 | N | 20417 | Cytochrome c oxidase subunit 1 (Fragment) OS=Simulium sp. BOLD:ACI0635 OX=1494722 GN=COI PE=3 SV=1 |
| 5 | 995 | tr\|A0A0C4KVJ8\|A0A0C4KVJ8\_9DIPT | 25.17 | 5 | 2 | 1 | N | 20533 | Cytochrome c oxidase subunit 1 (Fragment) OS=Simulium sp. BOLD:ACI0635 OX=1494722 GN=COI PE=3 SV=1 |
| 5 | 1002 | tr\|A0A0C4KIH2\|A0A0C4KIH2\_9DIPT | 25.17 | 5 | 2 | 1 | N | 20533 | Cytochrome c oxidase subunit 1 (Fragment) OS=Simulium decorum OX=7193 GN=COI PE=3 SV=1 |
| 5 | 1011 | tr\|A0A0C4KVN9\|A0A0C4KVN9\_9DIPT | 25.17 | 5 | 2 | 1 | N | 20689 | Cytochrome c oxidase subunit 1 (Fragment) OS=Simulium sp. BOLD:ACI0635 OX=1494722 GN=COI PE=3 SV=1 |
| 5 | 1070 | tr\|A0A0C4KQR5\|A0A0C4KQR5\_SIMVI | 25.17 | 5 | 2 | 1 | N | 21379 | Cytochrome c oxidase subunit 1 (Fragment) OS=Simulium vittatum OX=7192 GN=COI PE=3 SV=1 |
| 5 | 945 | tr\|A0A0P0AU98\|A0A0P0AU98\_9DIPT | 25.17 | 5 | 2 | 1 | N | 20090 | Cytochrome c oxidase subunit 1 (Fragment) OS=Simulium sp. BOLD:AAA4264 OX=1695073 GN=COI PE=3 SV=1 |
| 5 | 959 | tr\|A0A0P0C8G4\|A0A0P0C8G4\_9DIPT | 25.17 | 5 | 2 | 1 | N | 20316 | Cytochrome c oxidase subunit 1 (Fragment) OS=Simulium sp. BOLD:AAA4264 OX=1695073 GN=COI PE=3 SV=1 |
| 5 | 994 | tr\|A0A0P0A5K3\|A0A0P0A5K3\_9DIPT | 25.17 | 5 | 2 | 1 | N | 20533 | Cytochrome c oxidase subunit 1 (Fragment) OS=Simulium sp. BOLD:AAA8954 OX=1333387 GN=COI PE=3 SV=1 |
| 5 | 997 | tr\|A0A0N9G0Y1\|A0A0N9G0Y1\_SIMVI | 25.17 | 5 | 2 | 1 | N | 20547 | Cytochrome c oxidase subunit 1 (Fragment) OS=Simulium vittatum OX=7192 GN=COI PE=3 SV=1 |
| 5 | 1001 | tr\|A0A0P0C7Y2\|A0A0P0C7Y2\_9DIPT | 25.17 | 5 | 2 | 1 | N | 20533 | Cytochrome c oxidase subunit 1 (Fragment) OS=Simulium sp. BOLD:AAA4264 OX=1695073 GN=COI PE=3 SV=1 |
| 5 | 1725 | tr\|A0A0C4KIN5\|A0A0C4KIN5\_9DIPT | 25.17 | 6 | 2 | 1 | N | 19413 | Cytochrome c oxidase subunit 1 (Fragment) OS=Simulium sp. BOLD:ACI0635 OX=1494722 GN=COI PE=3 SV=1 |
| 5 | 1726 | tr\|A0A0C4KJ12\|A0A0C4KJ12\_SIMVI | 25.17 | 5 | 2 | 1 | N | 19514 | Cytochrome c oxidase subunit 1 (Fragment) OS=Simulium vittatum OX=7192 GN=COI PE=3 SV=1 |
| 5 | 1728 | tr\|A0A0C4KJT1\|A0A0C4KJT1\_9DIPT | 25.17 | 5 | 2 | 1 | N | 19500 | Cytochrome c oxidase subunit 1 (Fragment) OS=Simulium sp. BOLD:ACI0635 OX=1494722 GN=COI PE=3 SV=1 |
| 5 | 1814 | tr\|A0A0C4KVU9\|A0A0C4KVU9\_SIMVI | 25.17 | 5 | 2 | 1 | N | 19641 | Cytochrome c oxidase subunit 1 (Fragment) OS=Simulium vittatum OX=7192 GN=COI PE=3 SV=1 |
| 5 | 1734 | tr\|A0A0C4KUR6\|A0A0C4KUR6\_SIMVI | 25.17 | 5 | 2 | 1 | N | 19824 | Cytochrome c oxidase subunit 1 (Fragment) OS=Simulium vittatum OX=7192 GN=COI PE=3 SV=1 |
| 5 | 1735 | tr\|A0A0C4KIN8\|A0A0C4KIN8\_9DIPT | 25.17 | 5 | 2 | 1 | N | 19810 | Cytochrome c oxidase subunit 1 (Fragment) OS=Simulium sp. BOLD:ACI0635 OX=1494722 GN=COI PE=3 SV=1 |
| 5 | 1737 | tr\|A0A0C4KUP5\|A0A0C4KUP5\_SIMVI | 25.17 | 5 | 2 | 1 | N | 19937 | Cytochrome c oxidase subunit 1 (Fragment) OS=Simulium vittatum OX=7192 GN=COI PE=3 SV=1 |
| 5 | 1738 | tr\|A0A0C4KK16\|A0A0C4KK16\_9DIPT | 25.17 | 5 | 2 | 1 | N | 19923 | Cytochrome c oxidase subunit 1 (Fragment) OS=Simulium sp. BOLD:ACI0635 OX=1494722 GN=COI PE=3 SV=1 |
| 5 | 1739 | tr\|A0A0C4KJY8\|A0A0C4KJY8\_9DIPT | 25.17 | 5 | 2 | 1 | N | 19994 | Cytochrome c oxidase subunit 1 (Fragment) OS=Simulium sp. BOLD:ACI0635 OX=1494722 GN=COI PE=3 SV=1 |
| 5 | 1740 | tr\|A0A0C4KKB4\|A0A0C4KKB4\_9DIPT | 25.17 | 5 | 2 | 1 | N | 19994 | Cytochrome c oxidase subunit 1 (Fragment) OS=Simulium venustum OX=7194 GN=COI PE=3 SV=1 |
| 5 | 1741 | tr\|A0A0C4KQQ2\|A0A0C4KQQ2\_SIMVI | 25.17 | 5 | 2 | 1 | N | 20008 | Cytochrome c oxidase subunit 1 (Fragment) OS=Simulium vittatum OX=7192 GN=COI PE=3 SV=1 |
| 5 | 1742 | tr\|A0A0C4KKA9\|A0A0C4KKA9\_9DIPT | 25.17 | 5 | 2 | 1 | N | 19994 | Cytochrome c oxidase subunit 1 (Fragment) OS=Simulium tuberosum OX=357430 GN=COI PE=3 SV=1 |
| 5 | 1743 | tr\|A0A0C4KIZ2\|A0A0C4KIZ2\_9DIPT | 25.17 | 5 | 2 | 1 | N | 19994 | Cytochrome c oxidase subunit 1 (Fragment) OS=Simulium verecundum OX=109159 GN=COI PE=3 SV=1 |
| 5 | 1744 | tr\|A0A0C4KQL8\|A0A0C4KQL8\_9DIPT | 25.17 | 5 | 2 | 1 | N | 20051 | Cytochrome c oxidase subunit 1 (Fragment) OS=Simulium verecundum OX=109159 GN=COI PE=3 SV=1 |
| 5 | 1745 | tr\|A0A0C4KIW0\|A0A0C4KIW0\_9DIPT | 25.17 | 5 | 2 | 1 | N | 20051 | Cytochrome c oxidase subunit 1 (Fragment) OS=Simulium tuberosum OX=357430 GN=COI PE=3 SV=1 |
| 5 | 1746 | tr\|A0A0C4KVR0\|A0A0C4KVR0\_9DIPT | 25.17 | 5 | 2 | 1 | N | 20051 | Cytochrome c oxidase subunit 1 (Fragment) OS=Simulium venustum OX=7194 GN=COI PE=3 SV=1 |
| 5 | 1747 | tr\|A0A0C4KKI2\|A0A0C4KKI2\_SIMVI | 25.17 | 5 | 2 | 1 | N | 20065 | Cytochrome c oxidase subunit 1 (Fragment) OS=Simulium vittatum OX=7192 GN=COI PE=3 SV=1 |
| 5 | 1748 | tr\|A0A0C4KVK2\|A0A0C4KVK2\_9DIPT | 25.17 | 5 | 2 | 1 | N | 20051 | Cytochrome c oxidase subunit 1 (Fragment) OS=Simulium sp. BOLD:ACI0635 OX=1494722 GN=COI PE=3 SV=1 |
| 5 | 974 | tr\|A0A0C4KJ37\|A0A0C4KJ37\_SIMVI | 25.17 | 5 | 2 | 1 | N | 20482 | Cytochrome c oxidase subunit 1 (Fragment) OS=Simulium vittatum OX=7192 GN=COI PE=3 SV=1 |
| 5 | 978 | tr\|A0A0C4KVN2\|A0A0C4KVN2\_9DIPT | 25.17 | 5 | 2 | 1 | N | 20482 | Cytochrome c oxidase subunit 1 (Fragment) OS=Simulium sp. BOLD:ACI0635 OX=1494722 GN=COI PE=3 SV=1 |
| 5 | 980 | tr\|A0A0C4KUJ6\|A0A0C4KUJ6\_9DIPT | 25.17 | 5 | 2 | 1 | N | 20467 | Cytochrome c oxidase subunit 1 (Fragment) OS=Simulium tuberosum OX=357430 GN=COI PE=3 SV=1 |
| 5 | 982 | tr\|A0A0C4KQJ7\|A0A0C4KQJ7\_9DIPT | 25.17 | 5 | 2 | 1 | N | 20467 | Cytochrome c oxidase subunit 1 (Fragment) OS=Simulium sp. BOLD:ACI0635 OX=1494722 GN=COI PE=3 SV=1 |
| 5 | 984 | tr\|A0A0C4KIX6\|A0A0C4KIX6\_9DIPT | 25.17 | 5 | 2 | 1 | N | 20467 | Cytochrome c oxidase subunit 1 (Fragment) OS=Simulium verecundum OX=109159 GN=COI PE=3 SV=1 |
| 5 | 985 | tr\|A0A0C4KK32\|A0A0C4KK32\_9DIPT | 25.17 | 5 | 2 | 1 | N | 20487 | Cytochrome c oxidase subunit 1 (Fragment) OS=Simulium sp. BOLD:ACI0635 OX=1494722 GN=COI PE=3 SV=1 |
| 5 | 996 | tr\|A0A0C4KV04\|A0A0C4KV04\_SIMVI | 25.17 | 5 | 2 | 1 | N | 20595 | Cytochrome c oxidase subunit 1 (Fragment) OS=Simulium vittatum OX=7192 GN=COI PE=3 SV=1 |
| 5 | 1006 | tr\|A0A0C4KK35\|A0A0C4KK35\_9DIPT | 25.17 | 5 | 2 | 1 | N | 20694 | Cytochrome c oxidase subunit 1 (Fragment) OS=Simulium sp. BOLD:ACI0635 OX=1494722 GN=COI PE=3 SV=1 |
| 5 | 1007 | tr\|A0A0C4KQI0\|A0A0C4KQI0\_9DIPT | 25.17 | 5 | 2 | 1 | N | 20700 | Cytochrome c oxidase subunit 1 (Fragment) OS=Simulium sp. BOLD:ACI0635 OX=1494722 GN=COI PE=3 SV=1 |
| 5 | 1008 | tr\|A0A0C4KVF6\|A0A0C4KVF6\_9DIPT | 25.17 | 5 | 2 | 1 | N | 20694 | Cytochrome c oxidase subunit 1 (Fragment) OS=Simulium decorum OX=7193 GN=COI PE=3 SV=1 |
| 5 | 1009 | tr\|A0A0C4KQT6\|A0A0C4KQT6\_SIMVI | 25.17 | 5 | 2 | 1 | N | 20708 | Cytochrome c oxidase subunit 1 (Fragment) OS=Simulium vittatum OX=7192 GN=COI PE=3 SV=1 |
| 5 | 1018 | tr\|A0A0C4KUK9\|A0A0C4KUK9\_9DIPT | 25.17 | 5 | 2 | 1 | N | 20795 | Cytochrome c oxidase subunit 1 (Fragment) OS=Simulium verecundum OX=109159 GN=COI PE=3 SV=1 |
| 5 | 1022 | tr\|A0A0C4KQF5\|A0A0C4KQF5\_9DIPT | 25.17 | 5 | 2 | 1 | N | 20795 | Cytochrome c oxidase subunit 1 (Fragment) OS=Simulium sp. BOLD:ACI0635 OX=1494722 GN=COI PE=3 SV=1 |
| 5 | 1041 | tr\|A0A0C4KQN9\|A0A0C4KQN9\_9DIPT | 25.17 | 5 | 2 | 1 | N | 20910 | Cytochrome c oxidase subunit 1 (Fragment) OS=Simulium verecundum OX=109159 GN=COI PE=3 SV=1 |
| 5 | 1042 | tr\|A0A0C4KQQ5\|A0A0C4KQQ5\_SIMVI | 25.17 | 5 | 2 | 1 | N | 20924 | Cytochrome c oxidase subunit 1 (Fragment) OS=Simulium vittatum OX=7192 GN=COI PE=3 SV=1 |
| 5 | 1044 | tr\|A0A0C4KQL5\|A0A0C4KQL5\_9DIPT | 25.17 | 5 | 2 | 1 | N | 20910 | Cytochrome c oxidase subunit 1 (Fragment) OS=Simulium venustum OX=7194 GN=COI PE=3 SV=1 |
| 5 | 1045 | tr\|A0A0C4KQF3\|A0A0C4KQF3\_9DIPT | 25.17 | 5 | 2 | 1 | N | 20910 | Cytochrome c oxidase subunit 1 (Fragment) OS=Simulium sp. BOLD:ACI0635 OX=1494722 GN=COI PE=3 SV=1 |
| 5 | 1053 | tr\|A0A0C4KJ74\|A0A0C4KJ74\_SIMVI | 25.17 | 5 | 2 | 1 | N | 21080 | Cytochrome c oxidase subunit 1 (Fragment) OS=Simulium vittatum OX=7192 GN=COI PE=3 SV=1 |
| 5 | 1055 | tr\|A0A0C4KUA7\|A0A0C4KUA7\_9DIPT | 25.17 | 5 | 2 | 1 | N | 21066 | Cytochrome c oxidase subunit 1 (Fragment) OS=Simulium sp. BOLD:ACI0635 OX=1494722 GN=COI PE=3 SV=1 |
| 5 | 1056 | tr\|A0A0C4KVQ7\|A0A0C4KVQ7\_9DIPT | 25.17 | 5 | 2 | 1 | N | 21066 | Cytochrome c oxidase subunit 1 (Fragment) OS=Simulium tuberosum OX=357430 GN=COI PE=3 SV=1 |
| 5 | 1057 | tr\|A0A0C4KU78\|A0A0C4KU78\_9DIPT | 25.17 | 5 | 2 | 1 | N | 21066 | Cytochrome c oxidase subunit 1 (Fragment) OS=Simulium decorum OX=7193 GN=COI PE=3 SV=1 |
| 5 | 1060 | tr\|A0A0C4KVT0\|A0A0C4KVT0\_9DIPT | 25.17 | 5 | 2 | 1 | N | 21066 | Cytochrome c oxidase subunit 1 (Fragment) OS=Simulium verecundum OX=109159 GN=COI PE=3 SV=1 |
| 5 | 1066 | tr\|A0A0C4KQG5\|A0A0C4KQG5\_9DIPT | 25.17 | 5 | 2 | 1 | N | 21293 | Cytochrome c oxidase subunit 1 (Fragment) OS=Simulium sp. BOLD:ACI0635 OX=1494722 GN=COI PE=3 SV=1 |
| 5 | 1067 | tr\|A0A0C4KVY4\|A0A0C4KVY4\_SIMVI | 25.17 | 5 | 2 | 1 | N | 21307 | Cytochrome c oxidase subunit 1 (Fragment) OS=Simulium vittatum OX=7192 GN=COI PE=3 SV=1 |
| 5 | 1071 | tr\|A0A0C4KVX8\|A0A0C4KVX8\_SIMVI | 25.17 | 5 | 2 | 1 | N | 21422 | Cytochrome c oxidase subunit 1 (Fragment) OS=Simulium vittatum OX=7192 GN=COI PE=3 SV=1 |
| 5 | 1074 | tr\|A0A0C4KUN7\|A0A0C4KUN7\_9DIPT | 25.17 | 5 | 2 | 1 | N | 21408 | Cytochrome c oxidase subunit 1 (Fragment) OS=Simulium verecundum OX=109159 GN=COI PE=3 SV=1 |
| 5 | 1076 | tr\|A0A0C4KU72\|A0A0C4KU72\_9DIPT | 25.17 | 5 | 2 | 1 | N | 21361 | Cytochrome c oxidase subunit 1 (Fragment) OS=Simulium bracteatum OX=626788 GN=COI PE=3 SV=1 |
| 5 | 1077 | tr\|A0A0C4KUE0\|A0A0C4KUE0\_9DIPT | 25.17 | 5 | 2 | 1 | N | 21408 | Cytochrome c oxidase subunit 1 (Fragment) OS=Simulium sp. BOLD:ACI0635 OX=1494722 GN=COI PE=3 SV=1 |
| 5 | 1080 | tr\|A0A0C4KUF8\|A0A0C4KUF8\_9DIPT | 25.17 | 5 | 2 | 1 | N | 21509 | Cytochrome c oxidase subunit 1 (Fragment) OS=Simulium sp. BOLD:ACI0635 OX=1494722 GN=COI PE=3 SV=1 |
| 5 | 1082 | tr\|A0A0C4KQL2\|A0A0C4KQL2\_9DIPT | 25.17 | 5 | 2 | 1 | N | 21509 | Cytochrome c oxidase subunit 1 (Fragment) OS=Simulium tuberosum OX=357430 GN=COI PE=3 SV=1 |
| 5 | 1090 | tr\|A0A0C4KK84\|A0A0C4KK84\_9DIPT | 25.17 | 5 | 2 | 1 | N | 21743 | Cytochrome c oxidase subunit 1 (Fragment) OS=Simulium sp. BOLD:ACI0635 OX=1494722 GN=COI PE=3 SV=1 |
| 5 | 1092 | tr\|A0A0C4KK98\|A0A0C4KK98\_9DIPT | 25.17 | 5 | 2 | 1 | N | 21743 | Cytochrome c oxidase subunit 1 (Fragment) OS=Simulium tuberosum OX=357430 GN=COI PE=3 SV=1 |
| 5 | 1097 | tr\|A0A0C4KUJ0\|A0A0C4KUJ0\_9DIPT | 25.17 | 5 | 2 | 1 | N | 21890 | Cytochrome c oxidase subunit 1 (Fragment) OS=Simulium sp. BOLD:ACI0635 OX=1494722 GN=COI PE=3 SV=1 |
| 5 | 1117 | tr\|A0A0C4KQV6\|A0A0C4KQV6\_SIMVI | 25.17 | 5 | 2 | 1 | N | 22019 | Cytochrome c oxidase subunit 1 (Fragment) OS=Simulium vittatum OX=7192 GN=COI PE=3 SV=1 |
| 5 | 1119 | tr\|A0A0C4KVQ4\|A0A0C4KVQ4\_9DIPT | 25.17 | 5 | 2 | 1 | N | 22005 | Cytochrome c oxidase subunit 1 (Fragment) OS=Simulium tuberosum OX=357430 GN=COI PE=3 SV=1 |
| 5 | 1125 | tr\|A0A0C4KVT4\|A0A0C4KVT4\_SIMVI | 25.17 | 5 | 2 | 1 | N | 22120 | Cytochrome c oxidase subunit 1 (Fragment) OS=Simulium vittatum OX=7192 GN=COI PE=3 SV=1 |
| 5 | 1127 | tr\|A0A0C4KQT9\|A0A0C4KQT9\_SIMVI | 25.17 | 5 | 2 | 1 | N | 22116 | Cytochrome c oxidase subunit 1 (Fragment) OS=Simulium vittatum OX=7192 GN=COI PE=3 SV=1 |
| 5 | 1128 | tr\|A0A0C4KKF6\|A0A0C4KKF6\_9DIPT | 25.17 | 5 | 2 | 1 | N | 22102 | Cytochrome c oxidase subunit 1 (Fragment) OS=Simulium verecundum OX=109159 GN=COI PE=3 SV=1 |
| 5 | 1133 | tr\|A0A0C4KK79\|A0A0C4KK79\_9DIPT | 25.17 | 5 | 2 | 1 | N | 22102 | Cytochrome c oxidase subunit 1 (Fragment) OS=Simulium sp. BOLD:ACI0635 OX=1494722 GN=COI PE=3 SV=1 |
| 5 | 1160 | tr\|A0A0C4KJ00\|A0A0C4KJ00\_SIMVI | 25.17 | 5 | 2 | 1 | N | 22187 | Cytochrome c oxidase subunit 1 (Fragment) OS=Simulium vittatum OX=7192 GN=COI PE=3 SV=1 |
| 5 | 1161 | tr\|A0A0C4KIU8\|A0A0C4KIU8\_9DIPT | 25.17 | 5 | 2 | 1 | N | 22173 | Cytochrome c oxidase subunit 1 (Fragment) OS=Simulium sp. BOLD:ACI0635 OX=1494722 GN=COI PE=3 SV=1 |
| 5 | 1162 | tr\|A0A0C4KIV7\|A0A0C4KIV7\_9DIPT | 25.17 | 5 | 2 | 1 | N | 22173 | Cytochrome c oxidase subunit 1 (Fragment) OS=Simulium tuberosum OX=357430 GN=COI PE=3 SV=1 |
| 5 | 1171 | tr\|A0A0C4KIG5\|A0A0C4KIG5\_9DIPT | 25.17 | 5 | 2 | 1 | N | 22212 | Cytochrome c oxidase subunit 1 (Fragment) OS=Simulium bracteatum OX=626788 GN=COI PE=3 SV=1 |
| 5 | 1173 | tr\|A0A0C4KKI8\|A0A0C4KKI8\_SIMVI | 25.17 | 5 | 2 | 1 | N | 22244 | Cytochrome c oxidase subunit 1 (Fragment) OS=Simulium vittatum OX=7192 GN=COI PE=3 SV=1 |
| 5 | 1174 | tr\|A0A0C4KQN7\|A0A0C4KQN7\_9DIPT | 25.17 | 5 | 2 | 1 | N | 22230 | Cytochrome c oxidase subunit 1 (Fragment) OS=Simulium verecundum OX=109159 GN=COI PE=3 SV=1 |
| 5 | 1175 | tr\|A0A0C4KUG1\|A0A0C4KUG1\_9DIPT | 25.17 | 5 | 2 | 1 | N | 22230 | Cytochrome c oxidase subunit 1 (Fragment) OS=Simulium sp. BOLD:ACI0635 OX=1494722 GN=COI PE=3 SV=1 |
| 5 | 1186 | tr\|A0A0C4KIU5\|A0A0C4KIU5\_9DIPT | 25.17 | 5 | 2 | 1 | N | 22287 | Cytochrome c oxidase subunit 1 (Fragment) OS=Simulium sp. BOLD:ACI0635 OX=1494722 GN=COI PE=3 SV=1 |
| 5 | 1187 | tr\|A0A0C4KUM4\|A0A0C4KUM4\_9DIPT | 25.17 | 5 | 2 | 1 | N | 22287 | Cytochrome c oxidase subunit 1 (Fragment) OS=Simulium verecundum OX=109159 GN=COI PE=3 SV=1 |
| 5 | 1188 | tr\|A0A0C4KKL2\|A0A0C4KKL2\_SIMVI | 25.17 | 5 | 2 | 1 | N | 22301 | Cytochrome c oxidase subunit 1 (Fragment) OS=Simulium vittatum OX=7192 GN=COI PE=3 SV=1 |
| 5 | 1199 | tr\|A0A0C4KQT1\|A0A0C4KQT1\_SIMVI | 25.17 | 5 | 2 | 1 | N | 22359 | Cytochrome c oxidase subunit 1 (Fragment) OS=Simulium vittatum OX=7192 GN=COI PE=3 SV=1 |
| 5 | 1202 | tr\|A0A0C4KQK9\|A0A0C4KQK9\_9DIPT | 25.17 | 5 | 2 | 1 | N | 22347 | Cytochrome c oxidase subunit 1 (Fragment) OS=Simulium tuberosum OX=357430 GN=COI PE=3 SV=1 |
| 5 | 1203 | tr\|A0A0C4KK50\|A0A0C4KK50\_9DIPT | 25.17 | 5 | 2 | 1 | N | 22345 | Cytochrome c oxidase subunit 1 (Fragment) OS=Simulium sp. BOLD:ACI0635 OX=1494722 GN=COI PE=3 SV=1 |
| 5 | 1204 | tr\|A0A0C4KVQ1\|A0A0C4KVQ1\_9DIPT | 25.17 | 5 | 2 | 1 | N | 22345 | Cytochrome c oxidase subunit 1 (Fragment) OS=Simulium tuberosum OX=357430 GN=COI PE=3 SV=1 |
| 5 | 1205 | tr\|A0A0C4KUV1\|A0A0C4KUV1\_SIMVI | 25.17 | 5 | 2 | 1 | N | 22364 | Cytochrome c oxidase subunit 1 (Fragment) OS=Simulium vittatum OX=7192 GN=COI PE=3 SV=1 |
| 5 | 1206 | tr\|A0A0C4KIV2\|A0A0C4KIV2\_9DIPT | 25.17 | 5 | 2 | 1 | N | 22460 | Cytochrome c oxidase subunit 1 (Fragment) OS=Simulium tuberosum OX=357430 GN=COI PE=3 SV=1 |
| 5 | 1207 | tr\|A0A0C4KQR8\|A0A0C4KQR8\_SIMVI | 25.17 | 5 | 2 | 1 | N | 22474 | Cytochrome c oxidase subunit 1 (Fragment) OS=Simulium vittatum OX=7192 GN=COI PE=3 SV=1 |
| 5 | 1208 | tr\|A0A0C4KVP2\|A0A0C4KVP2\_9DIPT | 25.17 | 5 | 2 | 1 | N | 22460 | Cytochrome c oxidase subunit 1 (Fragment) OS=Simulium sp. BOLD:ACI0635 OX=1494722 GN=COI PE=3 SV=1 |
| 5 | 1209 | tr\|A0A0C4KKD0\|A0A0C4KKD0\_9DIPT | 25.17 | 5 | 2 | 1 | N | 22460 | Cytochrome c oxidase subunit 1 (Fragment) OS=Simulium verecundum OX=109159 GN=COI PE=3 SV=1 |
| 5 | 1210 | tr\|A0A0C4KQP5\|A0A0C4KQP5\_SIMVI | 25.17 | 5 | 2 | 1 | N | 22508 | Cytochrome c oxidase subunit 1 (Fragment) OS=Simulium vittatum OX=7192 GN=COI PE=3 SV=1 |
| 5 | 1211 | tr\|A0A0C4KVW9\|A0A0C4KVW9\_SIMVI | 25.17 | 5 | 2 | 1 | N | 22502 | Cytochrome c oxidase subunit 1 (Fragment) OS=Simulium vittatum OX=7192 GN=COI PE=3 SV=1 |
| 5 | 1219 | tr\|A0A0C4KQK3\|A0A0C4KQK3\_9DIPT | 25.17 | 5 | 2 | 1 | N | 22557 | Cytochrome c oxidase subunit 1 (Fragment) OS=Simulium tuberosum OX=357430 GN=COI PE=3 SV=1 |
| 5 | 1221 | tr\|A0A0C4KUX6\|A0A0C4KUX6\_SIMVI | 25.17 | 5 | 2 | 1 | N | 22571 | Cytochrome c oxidase subunit 1 (Fragment) OS=Simulium vittatum OX=7192 GN=COI PE=3 SV=1 |
| 5 | 1224 | tr\|A0A0C4KIQ1\|A0A0C4KIQ1\_9DIPT | 25.17 | 5 | 2 | 1 | N | 22557 | Cytochrome c oxidase subunit 1 (Fragment) OS=Simulium sp. BOLD:ACI0635 OX=1494722 GN=COI PE=3 SV=1 |
| 5 | 1225 | tr\|A0A0C4KVR5\|A0A0C4KVR5\_9DIPT | 25.17 | 5 | 2 | 1 | N | 22557 | Cytochrome c oxidase subunit 1 (Fragment) OS=Simulium verecundum OX=109159 GN=COI PE=3 SV=1 |
| 5 | 497 | tr\|A0A059T2B6\|A0A059T2B6\_9DIPT | 25.17 | 5 | 2 | 1 | N | 23534 | Cytochrome c oxidase subunit 1 (Fragment) OS=Simulium quadrivittatum OX=1452913 GN=COI PE=3 SV=1 |
| 5 | 36 | tr\|A0A059T209\|A0A059T209\_9DIPT | 25.17 | 5 | 2 | 1 | N | 23486 | Cytochrome c oxidase subunit 1 (Fragment) OS=Simulium exiguum s.l. BOLD:AAD3982 OX=1452922 GN=COI PE=3 SV=1 |
| 5 | 37 | tr\|A0A059T1R5\|A0A059T1R5\_9DIPT | 25.17 | 5 | 2 | 1 | N | 23530 | Cytochrome c oxidase subunit 1 (Fragment) OS=Simulium ganalesense OX=1452906 GN=COI PE=3 SV=1 |
| 5 | 38 | tr\|A0A059T279\|A0A059T279\_9DIPT | 25.17 | 5 | 2 | 1 | N | 23454 | Cytochrome c oxidase subunit 1 (Fragment) OS=Simulium damnosum complex sp. BOLD:ABV4542 OX=1452917 GN=COI PE=3 SV=1 |
| 5 | 39 | tr\|A0A059T276\|A0A059T276\_9DIPT | 25.17 | 5 | 2 | 1 | N | 23468 | Cytochrome c oxidase subunit 1 (Fragment) OS=Gigantodax nr. lazoi BOLD:ABA7591 OX=1452902 GN=COI PE=3 SV=1 |
| 5 | 498 | tr\|A0A059T288\|A0A059T288\_9DIPT | 25.17 | 5 | 2 | 1 | N | 23486 | Cytochrome c oxidase subunit 1 (Fragment) OS=Simulium callidum OX=1452903 GN=COI PE=3 SV=1 |
| 5 | 40 | tr\|A0A059T1S6\|A0A059T1S6\_9DIPT | 25.17 | 5 | 2 | 1 | N | 23472 | Cytochrome c oxidase subunit 1 (Fragment) OS=Simulium ochraceum s.l. BOLD:ABZ1370 OX=1452949 GN=COI PE=3 SV=1 |
| 5 | 499 | tr\|A0A059T2A1\|A0A059T2A1\_9DIPT | 25.17 | 5 | 2 | 1 | N | 23486 | Cytochrome c oxidase subunit 1 (Fragment) OS=Simulium inaequale OX=1452910 GN=COI PE=3 SV=1 |
| 5 | 41 | tr\|A0A059T236\|A0A059T236\_9DIPT | 25.17 | 5 | 2 | 1 | N | 23472 | Cytochrome c oxidase subunit 1 (Fragment) OS=Simulium ochraceum s.l. BOLD:ABU7853 OX=1452948 GN=COI PE=3 SV=1 |
| 5 | 42 | tr\|A0A059T1Q5\|A0A059T1Q5\_9DIPT | 25.17 | 5 | 2 | 1 | N | 23468 | Cytochrome c oxidase subunit 1 (Fragment) OS=Gigantodax basinflatus OX=1452895 GN=COI PE=3 SV=1 |
| 5 | 43 | tr\|A0A059T200\|A0A059T200\_9DIPT | 25.17 | 5 | 2 | 1 | N | 23472 | Cytochrome c oxidase subunit 1 (Fragment) OS=Simulium samboni OX=1452914 GN=COI PE=3 SV=1 |
| 5 | 44 | tr\|A0A059T1X3\|A0A059T1X3\_9DIPT | 25.17 | 5 | 2 | 1 | N | 23472 | Cytochrome c oxidase subunit 1 (Fragment) OS=Simulium haematopotum OX=1452908 GN=COI PE=3 SV=1 |
| 5 | 45 | tr\|A0A059T1V5\|A0A059T1V5\_9DIPT | 25.17 | 5 | 2 | 1 | N | 23468 | Cytochrome c oxidase subunit 1 (Fragment) OS=Gigantodax abalosi OX=1452894 GN=COI PE=3 SV=1 |
| 5 | 1727 | tr\|A0A0M3ZEE9\|A0A0M3ZEE9\_9DIPT | 25.17 | 5 | 2 | 1 | N | 19500 | Cytochrome c oxidase subunit 1 (Fragment) OS=Simulium venustum complex sp. BOLD:AAA4264 OX=1304597 GN=COI PE=3 SV=1 |
| 5 | 1733 | tr\|A0A0N9G4L4\|A0A0N9G4L4\_SIMVI | 25.17 | 5 | 2 | 1 | N | 19778 | Cytochrome c oxidase subunit 1 (Fragment) OS=Simulium vittatum OX=7192 GN=COI PE=3 SV=1 |
| 5 | 1815 | tr\|A0A0P0BPL0\|A0A0P0BPL0\_9DIPT | 25.17 | 5 | 2 | 1 | N | 20051 | Cytochrome c oxidase subunit 1 (Fragment) OS=Simulium sp. BOLD:AAA4264 OX=1695073 GN=COI PE=3 SV=1 |
| 5 | 1816 | tr\|A0A0P0BTB1\|A0A0P0BTB1\_9DIPT | 25.17 | 5 | 2 | 1 | N | 20051 | Cytochrome c oxidase subunit 1 (Fragment) OS=Prosimulium sp. BOLD:AAA1296 OX=1087820 GN=COI PE=3 SV=1 |
| 5 | 975 | tr\|A0A0P0AGA7\|A0A0P0AGA7\_9DIPT | 25.17 | 5 | 2 | 1 | N | 20482 | Cytochrome c oxidase subunit 1 (Fragment) OS=Simulium sp. BOLD:AAA4121 OX=1107295 GN=COI PE=3 SV=1 |
| 5 | 1027 | tr\|A0A0N9FVC2\|A0A0N9FVC2\_SIMVI | 25.17 | 5 | 2 | 1 | N | 20809 | Cytochrome c oxidase subunit 1 (Fragment) OS=Simulium vittatum OX=7192 GN=COI PE=3 SV=1 |
| 5 | 1043 | tr\|A0A0P0B3K8\|A0A0P0B3K8\_9DIPT | 25.17 | 5 | 2 | 1 | N | 20910 | Cytochrome c oxidase subunit 1 (Fragment) OS=Simulium sp. BOLD:AAA8954 OX=1333387 GN=COI PE=3 SV=1 |
| 5 | 1046 | tr\|A0A0P0BGV1\|A0A0P0BGV1\_9DIPT | 25.17 | 5 | 2 | 1 | N | 20910 | Cytochrome c oxidase subunit 1 (Fragment) OS=Prosimulium sp. BOLD:AAA1296 OX=1087820 GN=COI PE=3 SV=1 |
| 5 | 1050 | tr\|A0A0R6MJ84\|A0A0R6MJ84\_9DIPT | 25.17 | 5 | 2 | 1 | N | 21066 | Cytochrome c oxidase subunit 1 (Fragment) OS=Simulium nr. samboni BOLD:ABW2062 OX=1653161 GN=COI PE=3 SV=1 |
| 5 | 1051 | tr\|A0A0N9FTH1\|A0A0N9FTH1\_9DIPT | 25.17 | 5 | 2 | 1 | N | 21066 | Cytochrome c oxidase subunit 1 (Fragment) OS=Simulium sp. BOLD:AAB7749 OX=1695076 GN=COI PE=3 SV=1 |
| 5 | 1052 | tr\|A0A0P0B7G0\|A0A0P0B7G0\_9DIPT | 25.17 | 5 | 2 | 1 | N | 21066 | Cytochrome c oxidase subunit 1 (Fragment) OS=Simulium sp. BOLD:AAA9710 OX=1695075 GN=COI PE=3 SV=1 |
| 5 | 1054 | tr\|A0A0P0AC59\|A0A0P0AC59\_9DIPT | 25.17 | 5 | 2 | 1 | N | 21048 | Cytochrome c oxidase subunit 1 (Fragment) OS=Simulium sp. BOLD:AAA8323 OX=1695074 GN=COI PE=3 SV=1 |
| 5 | 1058 | tr\|A0A0P0B5Z6\|A0A0P0B5Z6\_9DIPT | 25.17 | 5 | 2 | 1 | N | 21066 | Cytochrome c oxidase subunit 1 (Fragment) OS=Simulium sp. BOLD:AAA1697 OX=1695072 GN=COI PE=3 SV=1 |
| 5 | 1059 | tr\|A0A0N7HRP6\|A0A0N7HRP6\_9DIPT | 25.17 | 5 | 2 | 1 | N | 21066 | Cytochrome c oxidase subunit 1 (Fragment) OS=Simulium sp. BOLD:AAA4264 OX=1695073 GN=COI PE=3 SV=1 |
| 5 | 1063 | tr\|A0A0P0BKN0\|A0A0P0BKN0\_9DIPT | 25.17 | 5 | 2 | 1 | N | 21180 | Cytochrome c oxidase subunit 1 (Fragment) OS=Simulium sp. BOLD:AAA4264 OX=1695073 GN=COI PE=3 SV=1 |
| 5 | 1064 | tr\|A0A0N9FSA0\|A0A0N9FSA0\_SIMVI | 25.17 | 5 | 2 | 1 | N | 21194 | Cytochrome c oxidase subunit 1 (Fragment) OS=Simulium vittatum OX=7192 GN=COI PE=3 SV=1 |
| 5 | 1075 | tr\|A0A0P0B802\|A0A0P0B802\_9DIPT | 25.17 | 5 | 2 | 1 | N | 21390 | Cytochrome c oxidase subunit 1 (Fragment) OS=Simulium sp. BOLD:AAB1114 OX=1333388 GN=COI PE=3 SV=1 |
| 5 | 1079 | tr\|A0A0P0C1B9\|A0A0P0C1B9\_9DIPT | 25.17 | 5 | 2 | 1 | N | 21539 | Cytochrome c oxidase subunit 1 (Fragment) OS=Simulium sp. BOLD:AAA4264 OX=1695073 GN=COI PE=3 SV=1 |
| 5 | 1081 | tr\|A0A0N9H6N0\|A0A0N9H6N0\_SIMVI | 25.17 | 5 | 2 | 1 | N | 21523 | Cytochrome c oxidase subunit 1 (Fragment) OS=Simulium vittatum OX=7192 GN=COI PE=3 SV=1 |
| 5 | 1087 | tr\|A0A0P0BW44\|A0A0P0BW44\_9DIPT | 25.17 | 5 | 2 | 1 | N | 21747 | Cytochrome c oxidase subunit 1 (Fragment) OS=Simulium sp. BOLD:AAA1697 OX=1695072 GN=COI PE=3 SV=1 |
| 5 | 1088 | tr\|A0A0N9G389\|A0A0N9G389\_SIMVI | 25.17 | 5 | 2 | 1 | N | 21757 | Cytochrome c oxidase subunit 1 (Fragment) OS=Simulium vittatum OX=7192 GN=COI PE=3 SV=1 |
| 5 | 1089 | tr\|A0A0P0BB57\|A0A0P0BB57\_9DIPT | 25.17 | 5 | 2 | 1 | N | 21761 | Cytochrome c oxidase subunit 1 (Fragment) OS=Simulium sp. BOLD:AAA1697 OX=1695072 GN=COI PE=3 SV=1 |
| 5 | 1091 | tr\|A0A0P0B9E8\|A0A0P0B9E8\_9DIPT | 25.17 | 5 | 2 | 1 | N | 21719 | Cytochrome c oxidase subunit 1 (Fragment) OS=Simulium sp. BOLD:AAA4264 OX=1695073 GN=COI PE=3 SV=1 |
| 5 | 1094 | tr\|A0A0P0BGZ8\|A0A0P0BGZ8\_9DIPT | 25.17 | 5 | 2 | 1 | N | 21713 | Cytochrome c oxidase subunit 1 (Fragment) OS=Simulium sp. BOLD:AAA4264 OX=1695073 GN=COI PE=3 SV=1 |
| 5 | 1098 | tr\|A0A0N7HU16\|A0A0N7HU16\_9DIPT | 25.17 | 5 | 2 | 1 | N | 21862 | Cytochrome c oxidase subunit 1 (Fragment) OS=Simulium sp. BOLD:AAA1697 OX=1695072 GN=COI PE=3 SV=1 |
| 5 | 1099 | tr\|A0A0P0BHK7\|A0A0P0BHK7\_9DIPT | 25.17 | 5 | 2 | 1 | N | 21867 | Cytochrome c oxidase subunit 1 (Fragment) OS=Simulium sp. BOLD:AAA1697 OX=1695072 GN=COI PE=3 SV=1 |
| 5 | 1101 | tr\|A0A0P0B7K9\|A0A0P0B7K9\_9DIPT | 25.17 | 5 | 2 | 1 | N | 21890 | Cytochrome c oxidase subunit 1 (Fragment) OS=Simulium sp. BOLD:AAB7749 OX=1695076 GN=COI PE=3 SV=1 |
| 5 | 1102 | tr\|A0A0R6MN48\|A0A0R6MN48\_9DIPT | 25.17 | 5 | 2 | 1 | N | 21872 | Cytochrome c oxidase subunit 1 (Fragment) OS=Gigantodax willei OX=1653088 GN=COI PE=3 SV=1 |
| 5 | 1113 | tr\|A0A0R6MM96\|A0A0R6MM96\_9DIPT | 25.17 | 5 | 2 | 1 | N | 22019 | Cytochrome c oxidase subunit 1 (Fragment) OS=Simulium exiguum s.l. BOLD:AAD3982 OX=1452922 GN=COI PE=3 SV=1 |
| 5 | 1116 | tr\|A0A0P0AY76\|A0A0P0AY76\_9DIPT | 25.17 | 5 | 2 | 1 | N | 22011 | Cytochrome c oxidase subunit 1 (Fragment) OS=Simulium sp. BOLD:AAA1697 OX=1695072 GN=COI PE=3 SV=1 |
| 5 | 1118 | tr\|A0A0N7HN65\|A0A0N7HN65\_9DIPT | 25.17 | 5 | 2 | 1 | N | 22005 | Cytochrome c oxidase subunit 1 (Fragment) OS=Simulium sp. BOLD:AAA1697 OX=1695072 GN=COI PE=3 SV=1 |
| 5 | 1136 | tr\|A0A0P0BQ60\|A0A0P0BQ60\_9DIPT | 25.17 | 5 | 2 | 1 | N | 22102 | Cytochrome c oxidase subunit 1 (Fragment) OS=Simulium sp. BOLD:AAA4264 OX=1695073 GN=COI PE=3 SV=1 |
| 5 | 1138 | tr\|A0A0P0AU82\|A0A0P0AU82\_9DIPT | 25.17 | 5 | 2 | 1 | N | 22074 | Cytochrome c oxidase subunit 1 (Fragment) OS=Simulium sp. BOLD:AAA1697 OX=1695072 GN=COI PE=3 SV=1 |
| 5 | 1158 | tr\|A0A0P0BZT2\|A0A0P0BZT2\_9DIPT | 25.17 | 5 | 2 | 1 | N | 22173 | Cytochrome c oxidase subunit 1 (Fragment) OS=Simulium sp. BOLD:AAA1697 OX=1695072 GN=COI PE=3 SV=1 |
| 5 | 1159 | tr\|A0A0P0BBX2\|A0A0P0BBX2\_9DIPT | 25.17 | 5 | 2 | 1 | N | 22173 | Cytochrome c oxidase subunit 1 (Fragment) OS=Simulium sp. BOLD:AAA4264 OX=1695073 GN=COI PE=3 SV=1 |
| 5 | 1176 | tr\|A0A0R6MLI4\|A0A0R6MLI4\_9DIPT | 25.17 | 5 | 2 | 1 | N | 22244 | Cytochrome c oxidase subunit 1 (Fragment) OS=Simulium exiguum s.l. BOLD:AAD3982 OX=1452922 GN=COI PE=3 SV=1 |
| 5 | 1181 | tr\|A0A0P0BXR7\|A0A0P0BXR7\_9DIPT | 25.17 | 5 | 2 | 1 | N | 22287 | Cytochrome c oxidase subunit 1 (Fragment) OS=Simulium sp. BOLD:AAB7749 OX=1695076 GN=COI PE=3 SV=1 |
| 5 | 1182 | tr\|A0A0P0AUV9\|A0A0P0AUV9\_9DIPT | 25.17 | 5 | 2 | 1 | N | 22263 | Cytochrome c oxidase subunit 1 (Fragment) OS=Simulium sp. BOLD:AAA1697 OX=1695072 GN=COI PE=3 SV=1 |
| 5 | 1183 | tr\|A0A0P0B1U2\|A0A0P0B1U2\_9DIPT | 25.17 | 5 | 2 | 1 | N | 22307 | Cytochrome c oxidase subunit 1 (Fragment) OS=Simulium sp. BOLD:AAA1697 OX=1695072 GN=COI PE=3 SV=1 |
| 5 | 1184 | tr\|A0A0P0BG39\|A0A0P0BG39\_9DIPT | 25.17 | 5 | 2 | 1 | N | 22317 | Cytochrome c oxidase subunit 1 (Fragment) OS=Simulium sp. BOLD:AAA4264 OX=1695073 GN=COI PE=3 SV=1 |
| 5 | 1185 | tr\|A0A0P0BEY2\|A0A0P0BEY2\_9DIPT | 25.17 | 5 | 2 | 1 | N | 22287 | Cytochrome c oxidase subunit 1 (Fragment) OS=Simulium sp. BOLD:AAA4264 OX=1695073 GN=COI PE=3 SV=1 |
| 5 | 1194 | tr\|A0A0P0BBW3\|A0A0P0BBW3\_9DIPT | 25.17 | 5 | 2 | 1 | N | 22345 | Cytochrome c oxidase subunit 1 (Fragment) OS=Simulium sp. BOLD:AAA4264 OX=1695073 GN=COI PE=3 SV=1 |
| 5 | 1195 | tr\|A0A0P0AY79\|A0A0P0AY79\_9DIPT | 25.17 | 5 | 2 | 1 | N | 22350 | Cytochrome c oxidase subunit 1 (Fragment) OS=Simulium sp. BOLD:AAA1697 OX=1695072 GN=COI PE=3 SV=1 |
| 5 | 1196 | tr\|A0A0P0BJD9\|A0A0P0BJD9\_9DIPT | 25.17 | 5 | 2 | 1 | N | 22410 | Cytochrome c oxidase subunit 1 (Fragment) OS=Simulium sp. BOLD:AAA1697 OX=1695072 GN=COI PE=3 SV=1 |
| 5 | 1197 | tr\|A0A0P0BHY9\|A0A0P0BHY9\_9DIPT | 25.17 | 5 | 2 | 1 | N | 22394 | Cytochrome c oxidase subunit 1 (Fragment) OS=Simulium sp. BOLD:AAA4264 OX=1695073 GN=COI PE=3 SV=1 |
| 5 | 1198 | tr\|A0A0P0B5D2\|A0A0P0B5D2\_9DIPT | 25.17 | 5 | 2 | 1 | N | 22424 | Cytochrome c oxidase subunit 1 (Fragment) OS=Simulium sp. BOLD:AAA1697 OX=1695072 GN=COI PE=3 SV=1 |
| 5 | 1201 | tr\|A0A0P0BQN5\|A0A0P0BQN5\_9DIPT | 25.17 | 5 | 2 | 1 | N | 22316 | Cytochrome c oxidase subunit 1 (Fragment) OS=Simulium sp. BOLD:AAA1697 OX=1695072 GN=COI PE=3 SV=1 |
| 5 | 1215 | tr\|A0A0P0BC13\|A0A0P0BC13\_9DIPT | 25.17 | 5 | 2 | 1 | N | 22557 | Cytochrome c oxidase subunit 1 (Fragment) OS=Simulium sp. BOLD:AAA4264 OX=1695073 GN=COI PE=3 SV=1 |
| 5 | 1216 | tr\|A0A0P0B3Q6\|A0A0P0B3Q6\_9DIPT | 25.17 | 5 | 2 | 1 | N | 22561 | Cytochrome c oxidase subunit 1 (Fragment) OS=Simulium sp. BOLD:AAA1697 OX=1695072 GN=COI PE=3 SV=1 |
| 5 | 1218 | tr\|A0A0R6MMH7\|A0A0R6MMH7\_9DIPT | 25.17 | 5 | 2 | 1 | N | 22571 | Cytochrome c oxidase subunit 1 (Fragment) OS=Simulium metallicum s.l. BOLD:ACF2593 OX=1653141 GN=COI PE=3 SV=1 |
| 5 | 1220 | tr\|A0A0P0AAN3\|A0A0P0AAN3\_9DIPT | 25.17 | 5 | 2 | 1 | N | 22532 | Cytochrome c oxidase subunit 1 (Fragment) OS=Simulium sp. BOLD:AAA1697 OX=1695072 GN=COI PE=3 SV=1 |
| 5 | 1223 | tr\|A0A0P0AUP5\|A0A0P0AUP5\_9DIPT | 25.17 | 5 | 2 | 1 | N | 22564 | Cytochrome c oxidase subunit 1 (Fragment) OS=Simulium sp. BOLD:AAA1697 OX=1695072 GN=COI PE=3 SV=1 |
| 5 | 1230 | tr\|A0A0R6MLN2\|A0A0R6MLN2\_9DIPT | 25.17 | 5 | 2 | 1 | N | 22797 | Cytochrome c oxidase subunit 1 (Fragment) OS=Simulium exiguum s.l. BOLD:AAD3982 OX=1452922 GN=COI PE=3 SV=1 |
| 5 | 1236 | tr\|A0A0R6MK33\|A0A0R6MK33\_9DIPT | 25.17 | 5 | 2 | 1 | N | 22960 | Cytochrome c oxidase subunit 1 (Fragment) OS=Simulium nr. puigi BOLD:ACF2922 OX=1653159 GN=COI PE=3 SV=1 |
| 5 | 1242 | tr\|A0A0R6MNV2\|A0A0R6MNV2\_9DIPT | 25.17 | 5 | 2 | 1 | N | 23088 | Cytochrome c oxidase subunit 1 (Fragment) OS=Simulium exiguum s.l. BOLD:AAD3982 OX=1452922 GN=COI PE=3 SV=1 |
| 5 | 1715 | tr\|A0A0R6MMX5\|A0A0R6MMX5\_9DIPT | 25.17 | 5 | 2 | 1 | N | 23539 | Cytochrome c oxidase subunit 1 (Fragment) OS=Simulium tarsatum OX=1265453 GN=COI PE=3 SV=1 |
| 5 | 1317 | tr\|A0A0R6MM23\|A0A0R6MM23\_9DIPT | 25.17 | 5 | 2 | 1 | N | 23486 | Cytochrome c oxidase subunit 1 (Fragment) OS=Simulium puigi OX=1653095 GN=COI PE=3 SV=1 |
| 5 | 1319 | tr\|A0A0R6ML68\|A0A0R6ML68\_9DIPT | 25.17 | 5 | 2 | 1 | N | 23486 | Cytochrome c oxidase subunit 1 (Fragment) OS=Simulium metallicum s.l. BOLD:ACF2593 OX=1653141 GN=COI PE=3 SV=1 |
| 5 | 1322 | tr\|A0A0R6MLM8\|A0A0R6MLM8\_9DIPT | 25.17 | 5 | 2 | 1 | N | 23486 | Cytochrome c oxidase subunit 1 (Fragment) OS=Simulium metallicum s.l. BOLD:ACE7650 OX=1653138 GN=COI PE=3 SV=1 |
| 5 | 1324 | tr\|A0A0R6MN43\|A0A0R6MN43\_9DIPT | 25.17 | 5 | 2 | 1 | N | 23497 | Cytochrome c oxidase subunit 1 (Fragment) OS=Simulium tarsatum OX=1265453 GN=COI PE=3 SV=1 |
| 5 | 1325 | tr\|A0A0R6MMC8\|A0A0R6MMC8\_9DIPT | 25.17 | 5 | 2 | 1 | N | 23486 | Cytochrome c oxidase subunit 1 (Fragment) OS=Simulium nr. inaequale BOLD:ABX4894 OX=1653148 GN=COI PE=3 SV=1 |
| 5 | 1326 | tr\|A0A0R6ML96\|A0A0R6ML96\_9DIPT | 25.17 | 5 | 2 | 1 | N | 23518 | Cytochrome c oxidase subunit 1 (Fragment) OS=Simulium tarsatum OX=1265453 GN=COI PE=3 SV=1 |
| 5 | 1327 | tr\|A0A0R6MLY7\|A0A0R6MLY7\_9DIPT | 25.17 | 5 | 2 | 1 | N | 23486 | Cytochrome c oxidase subunit 1 (Fragment) OS=Simulium paynei s.l. BOLD:AAF3742 OX=1653167 GN=COI PE=3 SV=1 |
| 5 | 1328 | tr\|A0A0R6MLQ9\|A0A0R6MLQ9\_9DIPT | 25.17 | 5 | 2 | 1 | N | 23491 | Cytochrome c oxidase subunit 1 (Fragment) OS=Simulium nr. metallicum s.l. BOLD:ACF2593 OX=1653151 GN=COI PE=3 SV=1 |
| 5 | 1330 | tr\|A0A0R6MJ59\|A0A0R6MJ59\_9DIPT | 25.17 | 5 | 2 | 1 | N | 23500 | Cytochrome c oxidase subunit 1 (Fragment) OS=Simulium gonzalezi OX=1452907 GN=COI PE=3 SV=1 |
| 5 | 1331 | tr\|A0A0R6MLJ4\|A0A0R6MLJ4\_9DIPT | 25.17 | 5 | 2 | 1 | N | 23486 | Cytochrome c oxidase subunit 1 (Fragment) OS=Simulium metallicum s.l. BOLD:ABA1844 OX=1452930 GN=COI PE=3 SV=1 |
| 5 | 1332 | tr\|A0A0R6MM06\|A0A0R6MM06\_9DIPT | 25.17 | 5 | 2 | 1 | N | 23486 | Cytochrome c oxidase subunit 1 (Fragment) OS=Simulium canadense OX=626791 GN=COI PE=3 SV=1 |
| 5 | 1333 | tr\|A0A0R6MJD2\|A0A0R6MJD2\_9DIPT | 25.17 | 5 | 2 | 1 | N | 23530 | Cytochrome c oxidase subunit 1 (Fragment) OS=Simulium jobbinsi OX=1653092 GN=COI PE=3 SV=1 |
| 5 | 1334 | tr\|A0A0R6MMP7\|A0A0R6MMP7\_9DIPT | 25.17 | 5 | 2 | 1 | N | 23486 | Cytochrome c oxidase subunit 1 (Fragment) OS=Simulium nr. metallicum s.l. BOLD:ACF2594 OX=1653152 GN=COI PE=3 SV=1 |
| 5 | 1335 | tr\|A0A0R6MJN2\|A0A0R6MJN2\_9DIPT | 25.17 | 5 | 2 | 1 | N | 23486 | Cytochrome c oxidase subunit 1 (Fragment) OS=Simulium travisi OX=1653096 GN=COI PE=3 SV=1 |
| 5 | 1336 | tr\|A0A0R6MLC4\|A0A0R6MLC4\_9DIPT | 25.17 | 5 | 2 | 1 | N | 23516 | Cytochrome c oxidase subunit 1 (Fragment) OS=Simulium metallicum s.l. BOLD:ABA1845 OX=1653135 GN=COI PE=3 SV=1 |
| 5 | 1338 | tr\|A0A0R6MLJ5\|A0A0R6MLJ5\_9DIPT | 25.17 | 5 | 2 | 1 | N | 23486 | Cytochrome c oxidase subunit 1 (Fragment) OS=Simulium nr. metallicum s.l. BOLD:ABA1845 OX=1653149 GN=COI PE=3 SV=1 |
| 5 | 1339 | tr\|A0A0R6MJB8\|A0A0R6MJB8\_9DIPT | 25.17 | 5 | 2 | 1 | N | 23438 | Cytochrome c oxidase subunit 1 (Fragment) OS=Simulium ochraceum s.l. BOLD:ABA1884 OX=1653165 GN=COI PE=3 SV=1 |
| 5 | 1340 | tr\|A0A0R6MN78\|A0A0R6MN78\_9DIPT | 25.17 | 5 | 2 | 1 | N | 23486 | Cytochrome c oxidase subunit 1 (Fragment) OS=Simulium nr. metallicum s.l. BOLD:ACF1735 OX=1653150 GN=COI PE=3 SV=1 |
| 5 | 1342 | tr\|A0A0R6MIH8\|A0A0R6MIH8\_9DIPT | 25.17 | 5 | 2 | 1 | N | 23472 | Cytochrome c oxidase subunit 1 (Fragment) OS=Simulium ochraceum s.l. BOLD:AAB8989 OX=1653163 GN=COI PE=3 SV=1 |
| 5 | 1343 | tr\|A0A0R6MMX3\|A0A0R6MMX3\_9DIPT | 25.17 | 5 | 2 | 1 | N | 23476 | Cytochrome c oxidase subunit 1 (Fragment) OS=Simulium ochraceum s.l. BOLD:AAB8990 OX=1653164 GN=COI PE=3 SV=1 |
| 5 | 1344 | tr\|A0A0R6MMZ6\|A0A0R6MMZ6\_9DIPT | 25.17 | 5 | 2 | 1 | N | 23486 | Cytochrome c oxidase subunit 1 (Fragment) OS=Simulium nr. puigi BOLD:AAB3268 OX=1653156 GN=COI PE=3 SV=1 |
| 5 | 1345 | tr\|A0A0R6MM51\|A0A0R6MM51\_9DIPT | 25.17 | 5 | 2 | 1 | N | 23472 | Cytochrome c oxidase subunit 1 (Fragment) OS=Simulium nr. samboni BOLD:AAB8870 OX=1653160 GN=COI PE=3 SV=1 |
| 5 | 1346 | tr\|A0A0R6MMR3\|A0A0R6MMR3\_9DIPT | 25.17 | 5 | 2 | 1 | N | 23486 | Cytochrome c oxidase subunit 1 (Fragment) OS=Simulium nr. puigi BOLD:ABA1872 OX=1653158 GN=COI PE=3 SV=1 |
| 5 | 1347 | tr\|A0A0R6MMM3\|A0A0R6MMM3\_9DIPT | 25.17 | 5 | 2 | 1 | N | 23512 | Cytochrome c oxidase subunit 1 (Fragment) OS=Simulium metallicum s.l. BOLD:ACF1735 OX=1653140 GN=COI PE=3 SV=1 |
| 5 | 1348 | tr\|A0A0R6MIQ7\|A0A0R6MIQ7\_9DIPT | 25.17 | 5 | 2 | 1 | N | 23486 | Cytochrome c oxidase subunit 1 (Fragment) OS=Simulium horacioi OX=1653090 GN=COI PE=3 SV=1 |
| 5 | 1350 | tr\|A0A0R6MIK0\|A0A0R6MIK0\_9DIPT | 25.17 | 5 | 2 | 1 | N | 23470 | Cytochrome c oxidase subunit 1 (Fragment) OS=Simulium callidum OX=1452903 GN=COI PE=3 SV=1 |
| 5 | 1351 | tr\|A0A0R6MMW5\|A0A0R6MMW5\_9DIPT | 25.17 | 5 | 2 | 1 | N | 23454 | Cytochrome c oxidase subunit 1 (Fragment) OS=Gigantodax willei OX=1653088 GN=COI PE=3 SV=1 |
| 5 | 1352 | tr\|A0A0R6MLU6\|A0A0R6MLU6\_9DIPT | 25.17 | 5 | 2 | 1 | N | 23484 | Cytochrome c oxidase subunit 1 (Fragment) OS=Simulium callidum OX=1452903 GN=COI PE=3 SV=1 |
| 5 | 1355 | tr\|A0A0R6MMT6\|A0A0R6MMT6\_9DIPT | 25.17 | 5 | 2 | 1 | N | 23486 | Cytochrome c oxidase subunit 1 (Fragment) OS=Simulium paynei s.l. BOLD:AAC4424 OX=1653166 GN=COI PE=3 SV=1 |
| 5 | 1356 | tr\|A0A0R6MNY2\|A0A0R6MNY2\_9DIPT | 25.17 | 5 | 2 | 1 | N | 23486 | Cytochrome c oxidase subunit 1 (Fragment) OS=Simulium metallicum s.l. BOLD:ABA1849 OX=1653137 GN=COI PE=3 SV=1 |
| 5 | 1357 | tr\|A0A0R6MLP4\|A0A0R6MLP4\_9DIPT | 25.17 | 5 | 2 | 1 | N | 23472 | Cytochrome c oxidase subunit 1 (Fragment) OS=Simulium ochraceum s.l. BOLD:AAB8988 OX=1653162 GN=COI PE=3 SV=1 |
| 5 | 1358 | tr\|A0A0R6MM54\|A0A0R6MM54\_9DIPT | 25.17 | 5 | 2 | 1 | N | 23516 | Cytochrome c oxidase subunit 1 (Fragment) OS=Simulium tarsatum OX=1265453 GN=COI PE=3 SV=1 |
| 5 | 1361 | tr\|A0A0R6MLH0\|A0A0R6MLH0\_9DIPT | 25.17 | 5 | 2 | 1 | N | 23508 | Cytochrome c oxidase subunit 1 (Fragment) OS=Simulium metallicum s.l. BOLD:ACF2593 OX=1653141 GN=COI PE=3 SV=1 |
| 5 | 1362 | tr\|A0A0R6MMQ7\|A0A0R6MMQ7\_9DIPT | 25.17 | 5 | 2 | 1 | N | 23486 | Cytochrome c oxidase subunit 1 (Fragment) OS=Simulium metallicum s.l. BOLD:ACF2594 OX=1653142 GN=COI PE=3 SV=1 |
| 5 | 1363 | tr\|A0A0R6MM84\|A0A0R6MM84\_9DIPT | 25.17 | 5 | 2 | 1 | N | 23486 | Cytochrome c oxidase subunit 1 (Fragment) OS=Simulium nr. panamense BOLD:AAB1113 OX=1653154 GN=COI PE=3 SV=1 |
| 5 | 1364 | tr\|A0A0M3YWA1\|A0A0M3YWA1\_9DIPT | 25.17 | 5 | 2 | 1 | N | 23472 | Cytochrome c oxidase subunit 1 (Fragment) OS=Simulium aff. venustum/truncatum BOLD:AAA4264 OX=1690413 GN=COI PE=3 SV=1 |
| 5 | 1367 | tr\|A0A0R6MLC8\|A0A0R6MLC8\_9DIPT | 25.17 | 5 | 2 | 1 | N | 23486 | Cytochrome c oxidase subunit 1 (Fragment) OS=Simulium nr. puigi BOLD:ACF2922 OX=1653159 GN=COI PE=3 SV=1 |
| 5 | 1368 | tr\|A0A0R6MLS7\|A0A0R6MLS7\_9DIPT | 25.17 | 5 | 2 | 1 | N | 23514 | Cytochrome c oxidase subunit 1 (Fragment) OS=Simulium tarsatum OX=1265453 GN=COI PE=3 SV=1 |
| 5 | 1369 | tr\|A0A0R6MLM6\|A0A0R6MLM6\_9DIPT | 25.17 | 5 | 2 | 1 | N | 23486 | Cytochrome c oxidase subunit 1 (Fragment) OS=Simulium nr. metallicum s.l. BOLD:ACF2593 OX=1653151 GN=COI PE=3 SV=1 |
| 5 | 1370 | tr\|A0A0R6MNC0\|A0A0R6MNC0\_9DIPT | 25.17 | 5 | 2 | 1 | N | 23486 | Cytochrome c oxidase subunit 1 (Fragment) OS=Simulium iriartei OX=1653091 GN=COI PE=3 SV=1 |
| 5 | 1371 | tr\|A0A0R6MLV9\|A0A0R6MLV9\_9DIPT | 25.17 | 5 | 2 | 1 | N | 23486 | Cytochrome c oxidase subunit 1 (Fragment) OS=Simulium metallicum s.l. BOLD:ABA1846 OX=1653136 GN=COI PE=3 SV=1 |
| 5 | 1373 | tr\|A0A0P0BAK3\|A0A0P0BAK3\_9DIPT | 25.17 | 5 | 2 | 1 | N | 23502 | Cytochrome c oxidase subunit 1 (Fragment) OS=Twinnia nova OX=61071 GN=COI PE=3 SV=1 |
| 5 | 1374 | tr\|A0A0R6MME0\|A0A0R6MME0\_9DIPT | 25.17 | 5 | 2 | 1 | N | 23472 | Cytochrome c oxidase subunit 1 (Fragment) OS=Simulium puigi OX=1653095 GN=COI PE=3 SV=1 |
| 5 | 1375 | tr\|A0A0R6MJ34\|A0A0R6MJ34\_9DIPT | 25.17 | 5 | 2 | 1 | N | 23486 | Cytochrome c oxidase subunit 1 (Fragment) OS=Simulium nr. puigi BOLD:ABA1847 OX=1653157 GN=COI PE=3 SV=1 |
| 5 | 1377 | tr\|A0A0R6MLG8\|A0A0R6MLG8\_9DIPT | 25.17 | 5 | 2 | 1 | N | 23472 | Cytochrome c oxidase subunit 1 (Fragment) OS=Simulium nr. metallicum s.l. BOLD:ACF1735 OX=1653150 GN=COI PE=3 SV=1 |
| 5 | 1378 | tr\|A0A0R6MMU3\|A0A0R6MMU3\_9DIPT | 25.17 | 5 | 2 | 1 | N | 23486 | Cytochrome c oxidase subunit 1 (Fragment) OS=Simulium nr. pseudocallidum BOLD:AAH9661 OX=1653155 GN=COI PE=3 SV=1 |
| 5 | 1380 | tr\|A0A0R6ML78\|A0A0R6ML78\_9DIPT | 25.17 | 5 | 2 | 1 | N | 23514 | Cytochrome c oxidase subunit 1 (Fragment) OS=Simulium gonzalezi OX=1452907 GN=COI PE=3 SV=1 |
| 5 | 1381 | tr\|A0A0R6MMJ5\|A0A0R6MMJ5\_9DIPT | 25.17 | 5 | 2 | 1 | N | 23486 | Cytochrome c oxidase subunit 1 (Fragment) OS=Simulium hieroglyphicum OX=1266554 GN=COI PE=3 SV=1 |
| 5 | 1383 | tr\|A0A0R6MIV4\|A0A0R6MIV4\_9DIPT | 25.17 | 5 | 2 | 1 | N | 23504 | Cytochrome c oxidase subunit 1 (Fragment) OS=Simulium gonzalezi OX=1452907 GN=COI PE=3 SV=1 |
| 5 | 1384 | tr\|A0A0R6MMS1\|A0A0R6MMS1\_9DIPT | 25.17 | 5 | 2 | 1 | N | 23472 | Cytochrome c oxidase subunit 1 (Fragment) OS=Simulium ochraceum s.l. BOLD:ABA1884 OX=1653165 GN=COI PE=3 SV=1 |
| 5 | 1385 | tr\|A0A0R6MM62\|A0A0R6MM62\_9DIPT | 25.17 | 5 | 2 | 1 | N | 23486 | Cytochrome c oxidase subunit 1 (Fragment) OS=Simulium exiguum s.l. BOLD:ABX7122 OX=1653127 GN=COI PE=3 SV=1 |
| 5 | 1722 | tr\|A0A0C4KJ04\|A0A0C4KJ04\_SIMVI | 25.17 | 6 | 2 | 1 | N | 19152 | Cytochrome c oxidase subunit 1 (Fragment) OS=Simulium vittatum OX=7192 GN=COI PE=3 SV=1 |
| 5 | 1729 | tr\|A0A0C4KVS0\|A0A0C4KVS0\_9DIPT | 25.17 | 5 | 2 | 1 | N | 19398 | Cytochrome c oxidase subunit 1 (Fragment) OS=Simulium verecundum OX=109159 GN=COI PE=3 SV=1 |
| 5 | 1731 | tr\|A0A0C4KJZ7\|A0A0C4KJZ7\_9DIPT | 25.17 | 5 | 2 | 1 | N | 19413 | Cytochrome c oxidase subunit 1 (Fragment) OS=Simulium sp. BOLD:ACI0635 OX=1494722 GN=COI PE=3 SV=1 |
| 5 | 925 | tr\|A0A0C4KJV3\|A0A0C4KJV3\_9DIPT | 25.17 | 5 | 2 | 1 | N | 19740 | Cytochrome c oxidase subunit 1 (Fragment) OS=Simulium sp. BOLD:ACI0635 OX=1494722 GN=COI PE=3 SV=1 |
| 5 | 928 | tr\|A0A0C4KQW3\|A0A0C4KQW3\_SIMVI | 25.17 | 5 | 2 | 1 | N | 19896 | Cytochrome c oxidase subunit 1 (Fragment) OS=Simulium vittatum OX=7192 GN=COI PE=3 SV=1 |
| 5 | 957 | tr\|A0A0C4KUB8\|A0A0C4KUB8\_9DIPT | 25.17 | 5 | 2 | 1 | N | 20272 | Cytochrome c oxidase subunit 1 (Fragment) OS=Simulium sp. BOLD:ACI0635 OX=1494722 GN=COI PE=3 SV=1 |
| 5 | 958 | tr\|A0A0C4KUJ2\|A0A0C4KUJ2\_9DIPT | 25.17 | 5 | 2 | 1 | N | 20366 | Cytochrome c oxidase subunit 1 (Fragment) OS=Simulium tuberosum OX=357430 GN=COI PE=3 SV=1 |
| 5 | 976 | tr\|A0A0C4KU92\|A0A0C4KU92\_9DIPT | 25.17 | 5 | 2 | 1 | N | 20453 | Cytochrome c oxidase subunit 1 (Fragment) OS=Simulium sp. BOLD:ACI0635 OX=1494722 GN=COI PE=3 SV=1 |
| 5 | 998 | tr\|A0A0C4KVR6\|A0A0C4KVR6\_9DIPT | 25.17 | 5 | 2 | 1 | N | 20542 | Cytochrome c oxidase subunit 1 (Fragment) OS=Simulium verecundum OX=109159 GN=COI PE=3 SV=1 |
| 5 | 1115 | tr\|A0A068FG89\|A0A068FG89\_9DIPT | 25.17 | 5 | 2 | 1 | N | 21675 | Cytochrome c oxidase subunit 1 (Fragment) OS=Simulium arcticum sensu lato CW22\_8 OX=1513955 GN=COI PE=3 SV=1 |
| 5 | 440 | tr\|A0A068FF14\|A0A068FF14\_9DIPT | 25.17 | 5 | 2 | 1 | N | 21772 | Cytochrome c oxidase subunit 1 (Fragment) OS=Simulium arcticum sensu lato RC9\_13 OX=1513987 GN=COI PE=3 SV=1 |
| 5 | 441 | tr\|A0A068FF33\|A0A068FF33\_9DIPT | 25.17 | 5 | 2 | 1 | N | 21772 | Cytochrome c oxidase subunit 1 (Fragment) OS=Simulium arcticum sensu lato RC19\_32 OX=1513981 GN=COI PE=3 SV=1 |
| 5 | 1129 | tr\|A0A068FGA3\|A0A068FGA3\_9DIPT | 25.17 | 5 | 2 | 1 | N | 21772 | Cytochrome c oxidase subunit 1 (Fragment) OS=Simulium arcticum sensu lato RC9\_9 OX=1514000 GN=COI PE=3 SV=1 |
| 5 | 442 | tr\|A0A068FF22\|A0A068FF22\_9DIPT | 25.17 | 5 | 2 | 1 | N | 21772 | Cytochrome c oxidase subunit 1 (Fragment) OS=Simulium arcticum sensu lato RC19\_9 OX=1513985 GN=COI PE=3 SV=1 |
| 5 | 443 | tr\|A0A068FE44\|A0A068FE44\_9DIPT | 25.17 | 5 | 2 | 1 | N | 21772 | Cytochrome c oxidase subunit 1 (Fragment) OS=Simulium arcticum sensu lato RC9\_23 OX=1513993 GN=COI PE=3 SV=1 |
| 5 | 444 | tr\|A0A068FF02\|A0A068FF02\_9DIPT | 25.17 | 5 | 2 | 1 | N | 21772 | Cytochrome c oxidase subunit 1 (Fragment) OS=Simulium arcticum sensu lato CW22\_10 OX=1513943 GN=COI PE=3 SV=1 |
| 5 | 445 | tr\|A0A068FE49\|A0A068FE49\_9DIPT | 25.17 | 5 | 2 | 1 | N | 21772 | Cytochrome c oxidase subunit 1 (Fragment) OS=Simulium arcticum sensu lato RC19\_10 OX=1513971 GN=COI PE=3 SV=1 |
| 5 | 446 | tr\|A0A068FF29\|A0A068FF29\_9DIPT | 25.17 | 5 | 2 | 1 | N | 21772 | Cytochrome c oxidase subunit 1 (Fragment) OS=Simulium arcticum sensu lato RC19\_20 OX=1513975 GN=COI PE=3 SV=1 |
| 5 | 1142 | tr\|A0A068FJZ5\|A0A068FJZ5\_9DIPT | 25.17 | 5 | 2 | 1 | N | 21772 | Cytochrome c oxidase subunit 1 (Fragment) OS=Simulium arcticum sensu lato RC9\_8 OX=1513999 GN=COI PE=3 SV=1 |
| 5 | 1144 | tr\|A0A068FGB4\|A0A068FGB4\_9DIPT | 25.17 | 5 | 2 | 1 | N | 21772 | Cytochrome c oxidase subunit 1 (Fragment) OS=Simulium arcticum sensu lato RC19\_14 OX=1513973 GN=COI PE=3 SV=1 |
| 5 | 447 | tr\|A0A068FF18\|A0A068FF18\_9DIPT | 25.17 | 5 | 2 | 1 | N | 21772 | Cytochrome c oxidase subunit 1 (Fragment) OS=Simulium arcticum sensu lato RC9\_22 OX=1513992 GN=COI PE=3 SV=1 |
| 5 | 1149 | tr\|A0A0H3VZF8\|A0A0H3VZF8\_9DIPT | 25.17 | 5 | 2 | 1 | N | 21772 | Cytochrome c oxidase subunit 1 (Fragment) OS=Simulium arcticum sensu lato CAhyb40 OX=1654732 GN=COI PE=3 SV=1 |
| 5 | 448 | tr\|A0A068FE25\|A0A068FE25\_9DIPT | 25.17 | 5 | 2 | 1 | N | 21734 | Cytochrome c oxidase subunit 1 (Fragment) OS=Simulium arcticum sensu lato CW22\_6 OX=1513953 GN=COI PE=3 SV=1 |
| 5 | 1152 | tr\|A0A068FK01\|A0A068FK01\_9DIPT | 25.17 | 5 | 2 | 1 | N | 21772 | Cytochrome c oxidase subunit 1 (Fragment) OS=Simulium arcticum sensu lato RC9\_16 OX=1513989 GN=COI PE=3 SV=1 |
| 5 | 1153 | tr\|A0A068FG81\|A0A068FG81\_9DIPT | 25.17 | 5 | 2 | 1 | N | 21772 | Cytochrome c oxidase subunit 1 (Fragment) OS=Simulium arcticum OX=390676 GN=COI PE=3 SV=1 |
| 5 | 449 | tr\|A0A068FEX8\|A0A068FEX8\_9DIPT | 25.17 | 5 | 2 | 1 | N | 21772 | Cytochrome c oxidase subunit 1 (Fragment) OS=Simulium apricarium OX=390680 GN=COI PE=3 SV=1 |
| 5 | 450 | tr\|A0A068FE32\|A0A068FE32\_9DIPT | 25.17 | 5 | 2 | 1 | N | 21772 | Cytochrome c oxidase subunit 1 (Fragment) OS=Simulium arcticum sensu lato CW22\_17 OX=1513949 GN=COI PE=3 SV=1 |
| 5 | 451 | tr\|A0A068FDB5\|A0A068FDB5\_9DIPT | 25.17 | 5 | 2 | 1 | N | 21772 | Cytochrome c oxidase subunit 1 (Fragment) OS=Simulium arcticum sensu lato RC19\_19 OX=1513974 GN=COI PE=3 SV=1 |
| 5 | 452 | tr\|A0A068FDB0\|A0A068FDB0\_9DIPT | 25.17 | 5 | 2 | 1 | N | 21772 | Cytochrome c oxidase subunit 1 (Fragment) OS=Simulium arcticum sensu lato RC19\_7 OX=1513984 GN=COI PE=3 SV=1 |
| 5 | 453 | tr\|A0A068FE41\|A0A068FE41\_9DIPT | 25.17 | 5 | 2 | 1 | N | 21772 | Cytochrome c oxidase subunit 1 (Fragment) OS=Simulium arcticum sensu lato RC9\_14 OX=1513988 GN=COI PE=3 SV=1 |
| 5 | 455 | tr\|A0A068FE57\|A0A068FE57\_9DIPT | 25.17 | 5 | 2 | 1 | N | 21998 | Cytochrome c oxidase subunit 1 (Fragment) OS=Simulium arcticum sensu lato RC19\_35 OX=1513982 GN=COI PE=3 SV=1 |
| 5 | 456 | tr\|A0A068FE21\|A0A068FE21\_9DIPT | 25.17 | 5 | 2 | 1 | N | 22161 | Cytochrome c oxidase subunit 1 (Fragment) OS=Simulium arcticum OX=390676 GN=COI PE=3 SV=1 |
| 5 | 457 | tr\|A0A068FDC3\|A0A068FDC3\_9DIPT | 25.17 | 5 | 2 | 1 | N | 22829 | Cytochrome c oxidase subunit 1 (Fragment) OS=Simulium arcticum OX=390676 GN=COI PE=3 SV=1 |
| 5 | 458 | tr\|A0A068FEH9\|A0A068FEH9\_9DIPT | 25.17 | 5 | 2 | 1 | N | 22540 | Cytochrome c oxidase subunit 1 (Fragment) OS=Simulium murmanum OX=626804 GN=COI PE=3 SV=1 |
| 5 | 459 | tr\|A0A068FDD2\|A0A068FDD2\_9DIPT | 25.17 | 5 | 2 | 1 | N | 23234 | Cytochrome c oxidase subunit 1 (Fragment) OS=Simulium arcticum OX=390676 GN=COI PE=3 SV=1 |
| 5 | 1248 | tr\|A0A068FJX0\|A0A068FJX0\_9DIPT | 25.17 | 5 | 2 | 1 | N | 23224 | Cytochrome c oxidase subunit 1 (Fragment) OS=Simulium arcticum sensu lato CW22\_1 OX=1513942 GN=COI PE=3 SV=1 |
| 5 | 460 | tr\|A0A068FE08\|A0A068FE08\_9DIPT | 25.17 | 5 | 2 | 1 | N | 23224 | Cytochrome c oxidase subunit 1 (Fragment) OS=Simulium apricarium OX=390680 GN=COI PE=3 SV=1 |
| 5 | 1253 | tr\|A0A068FJY2\|A0A068FJY2\_9DIPT | 25.17 | 5 | 2 | 1 | N | 23362 | Cytochrome c oxidase subunit 1 (Fragment) OS=Simulium arcticum sensu lato CW22\_13 OX=1513945 GN=COI PE=3 SV=1 |
| 5 | 1254 | tr\|A0A068FKF2\|A0A068FKF2\_9DIPT | 25.17 | 5 | 2 | 1 | N | 23362 | Cytochrome c oxidase subunit 1 (Fragment) OS=Simulium arcticum sensu lato BL19\_10 OX=1513912 GN=COI PE=3 SV=1 |
| 5 | 1255 | tr\|A0A068FGL3\|A0A068FGL3\_9DIPT | 25.17 | 5 | 2 | 1 | N | 23362 | Cytochrome c oxidase subunit 1 (Fragment) OS=Simulium arcticum sensu lato BL19\_11 OX=1513913 GN=COI PE=3 SV=1 |
| 5 | 461 | tr\|A0A068FE96\|A0A068FE96\_9DIPT | 25.17 | 5 | 2 | 1 | N | 23362 | Cytochrome c oxidase subunit 1 (Fragment) OS=Simulium arcticum sensu lato LB18\_5 OX=1513966 GN=COI PE=3 SV=1 |
| 5 | 1256 | tr\|A0A068FG62\|A0A068FG62\_9DIPT | 25.17 | 5 | 2 | 1 | N | 23362 | Cytochrome c oxidase subunit 1 (Fragment) OS=Simulium apricarium OX=390680 GN=COI PE=3 SV=1 |
| 5 | 1257 | tr\|A0A068FK74\|A0A068FK74\_9DIPT | 25.17 | 5 | 2 | 1 | N | 23362 | Cytochrome c oxidase subunit 1 (Fragment) OS=Simulium arcticum sensu lato LB18\_11 OX=1513959 GN=COI PE=3 SV=1 |
| 5 | 462 | tr\|A0A068FEE7\|A0A068FEE7\_9DIPT | 25.17 | 5 | 2 | 1 | N | 23362 | Cytochrome c oxidase subunit 1 (Fragment) OS=Simulium arcticum sensu lato BL9\_1 OX=1513927 GN=COI PE=3 SV=1 |
| 5 | 463 | tr\|A0A068FEG2\|A0A068FEG2\_9DIPT | 25.17 | 5 | 2 | 1 | N | 23362 | Cytochrome c oxidase subunit 1 (Fragment) OS=Simulium arcticum sensu lato BL19\_3 OX=1513921 GN=COI PE=3 SV=1 |
| 5 | 464 | tr\|A0A068FD91\|A0A068FD91\_9DIPT | 25.17 | 5 | 2 | 1 | N | 23362 | Cytochrome c oxidase subunit 1 (Fragment) OS=Simulium arcticum sensu lato CW22\_15 OX=1513947 GN=COI PE=3 SV=1 |
| 5 | 465 | tr\|A0A068FE52\|A0A068FE52\_9DIPT | 25.17 | 5 | 2 | 1 | N | 23362 | Cytochrome c oxidase subunit 1 (Fragment) OS=Simulium arcticum sensu lato RC19\_23 OX=1513976 GN=COI PE=3 SV=1 |
| 5 | 466 | tr\|A0A068FEG7\|A0A068FEG7\_9DIPT | 25.17 | 5 | 2 | 1 | N | 23362 | Cytochrome c oxidase subunit 1 (Fragment) OS=Simulium arcticum sensu lato BL19\_8 OX=1513926 GN=COI PE=3 SV=1 |
| 5 | 467 | tr\|A0A068FEA3\|A0A068FEA3\_9DIPT | 25.17 | 5 | 2 | 1 | N | 23362 | Cytochrome c oxidase subunit 1 (Fragment) OS=Simulium arcticum sensu lato LB18\_10 OX=1513958 GN=COI PE=3 SV=1 |
| 5 | 1258 | tr\|A0A068FFD1\|A0A068FFD1\_9DIPT | 25.17 | 5 | 2 | 1 | N | 23392 | Cytochrome c oxidase subunit 1 (Fragment) OS=Simulium arcticum sensu lato BL19\_27 OX=1513920 GN=COI PE=3 SV=1 |
| 5 | 1259 | tr\|A0A068FKD6\|A0A068FKD6\_9DIPT | 25.17 | 5 | 2 | 1 | N | 23362 | Cytochrome c oxidase subunit 1 (Fragment) OS=Simulium arcticum sensu lato BL9\_28 OX=1513935 GN=COI PE=3 SV=1 |
| 5 | 1260 | tr\|A0A068FKB8\|A0A068FKB8\_9DIPT | 25.17 | 5 | 2 | 1 | N | 23362 | Cytochrome c oxidase subunit 1 (Fragment) OS=Simulium arcticum sensu lato BL9\_3 OX=1513937 GN=COI PE=3 SV=1 |
| 5 | 1261 | tr\|A0A068FJX6\|A0A068FJX6\_9DIPT | 25.17 | 5 | 2 | 1 | N | 23362 | Cytochrome c oxidase subunit 1 (Fragment) OS=Simulium arcticum sensu lato CW22\_7 OX=1513954 GN=COI PE=3 SV=1 |
| 5 | 1262 | tr\|A0A068FGB0\|A0A068FGB0\_9DIPT | 25.17 | 5 | 2 | 1 | N | 23362 | Cytochrome c oxidase subunit 1 (Fragment) OS=Simulium arcticum sensu lato RC19\_4 OX=1513983 GN=COI PE=3 SV=1 |
| 5 | 469 | tr\|A0A068FDM8\|A0A068FDM8\_9DIPT | 25.17 | 5 | 2 | 1 | N | 23351 | Cytochrome c oxidase subunit 1 (Fragment) OS=Simulium arcticum sensu lato BL19\_22 OX=1513919 GN=COI PE=3 SV=1 |
| 5 | 1263 | tr\|A0A068FK07\|A0A068FK07\_9DIPT | 25.17 | 5 | 2 | 1 | N | 23362 | Cytochrome c oxidase subunit 1 (Fragment) OS=Simulium arcticum sensu lato RC19\_3 OX=1513979 GN=COI PE=3 SV=1 |
| 5 | 1265 | tr\|A0A068FK49\|A0A068FK49\_9DIPT | 25.17 | 5 | 2 | 1 | N | 23362 | Cytochrome c oxidase subunit 1 (Fragment) OS=Simulium brevicercum OX=390678 GN=COI PE=3 SV=1 |
| 5 | 470 | tr\|A0A068FDL4\|A0A068FDL4\_9DIPT | 25.17 | 5 | 2 | 1 | N | 23362 | Cytochrome c oxidase subunit 1 (Fragment) OS=Simulium arcticum sensu lato BL9\_32 OX=1513938 GN=COI PE=3 SV=1 |
| 5 | 1267 | tr\|A0A068FKC8\|A0A068FKC8\_9DIPT | 25.17 | 5 | 2 | 1 | N | 23362 | Cytochrome c oxidase subunit 1 (Fragment) OS=Simulium arcticum sensu lato BL9\_12 OX=1513930 GN=COI PE=3 SV=1 |
| 5 | 1268 | tr\|A0A068FGK7\|A0A068FGK7\_9DIPT | 25.17 | 5 | 2 | 1 | N | 23362 | Cytochrome c oxidase subunit 1 (Fragment) OS=Simulium arcticum sensu lato BL19\_5 OX=1513923 GN=COI PE=3 SV=1 |
| 5 | 1269 | tr\|A0A068FGE3\|A0A068FGE3\_9DIPT | 25.17 | 5 | 2 | 1 | N | 23362 | Cytochrome c oxidase subunit 1 (Fragment) OS=Simulium arcticum sensu lato LB18\_2 OX=1513963 GN=COI PE=3 SV=1 |
| 5 | 1271 | tr\|A0A068FGK0\|A0A068FGK0\_9DIPT | 25.17 | 5 | 2 | 1 | N | 23362 | Cytochrome c oxidase subunit 1 (Fragment) OS=Simulium arcticum sensu lato BL9\_29 OX=1513936 GN=COI PE=3 SV=1 |
| 5 | 471 | tr\|A0A068FD80\|A0A068FD80\_9DIPT | 25.17 | 5 | 2 | 1 | N | 23362 | Cytochrome c oxidase subunit 1 (Fragment) OS=Simulium arcticum sensu lato CW22\_4 OX=1513951 GN=COI PE=3 SV=1 |
| 5 | 1273 | tr\|A0A068FGB7\|A0A068FGB7\_9DIPT | 25.17 | 5 | 2 | 1 | N | 23362 | Cytochrome c oxidase subunit 1 (Fragment) OS=Simulium arcticum sensu lato RC19\_26 OX=1513978 GN=COI PE=3 SV=1 |
| 5 | 1274 | tr\|A0A068FFA7\|A0A068FFA7\_9DIPT | 25.17 | 5 | 2 | 1 | N | 23362 | Cytochrome c oxidase subunit 1 (Fragment) OS=Simulium arcticum sensu lato BL9\_10 OX=1513928 GN=COI PE=3 SV=1 |
| 5 | 472 | tr\|A0A068FE29\|A0A068FE29\_9DIPT | 25.17 | 5 | 2 | 1 | N | 23362 | Cytochrome c oxidase subunit 1 (Fragment) OS=Simulium arcticum sensu lato CW22\_11 OX=1513944 GN=COI PE=3 SV=1 |
| 5 | 1275 | tr\|A0A068FFC1\|A0A068FFC1\_9DIPT | 25.17 | 5 | 2 | 1 | N | 23362 | Cytochrome c oxidase subunit 1 (Fragment) OS=Simulium arcticum sensu lato BL19\_7 OX=1513925 GN=COI PE=3 SV=1 |
| 5 | 473 | tr\|A0A068FDA0\|A0A068FDA0\_9DIPT | 25.17 | 5 | 2 | 1 | N | 23362 | Cytochrome c oxidase subunit 1 (Fragment) OS=Simulium arcticum sensu lato RC9\_11 OX=1513986 GN=COI PE=3 SV=1 |
| 5 | 474 | tr\|A0A068FF74\|A0A068FF74\_9DIPT | 25.17 | 5 | 2 | 1 | N | 23381 | Cytochrome c oxidase subunit 1 (Fragment) OS=Simulium arcticum sensu lato LB18\_14 OX=1513962 GN=COI PE=3 SV=1 |
| 5 | 1276 | tr\|A0A068FK98\|A0A068FK98\_9DIPT | 25.17 | 5 | 2 | 1 | N | 23362 | Cytochrome c oxidase subunit 1 (Fragment) OS=Simulium negativum OX=390681 GN=COI PE=3 SV=1 |
| 5 | 1277 | tr\|A0A068FKE3\|A0A068FKE3\_9DIPT | 25.17 | 5 | 2 | 1 | N | 23362 | Cytochrome c oxidase subunit 1 (Fragment) OS=Simulium arcticum sensu lato BL19\_4 OX=1513922 GN=COI PE=3 SV=1 |
| 5 | 1278 | tr\|A0A068FGL9\|A0A068FGL9\_9DIPT | 25.17 | 5 | 2 | 1 | N | 23362 | Cytochrome c oxidase subunit 1 (Fragment) OS=Simulium arcticum sensu lato BL19\_18 OX=1513918 GN=COI PE=3 SV=1 |
| 5 | 1279 | tr\|A0A068FJY9\|A0A068FJY9\_9DIPT | 25.17 | 5 | 2 | 1 | N | 23362 | Cytochrome c oxidase subunit 1 (Fragment) OS=Simulium arcticum sensu lato RC9\_3 OX=1513994 GN=COI PE=3 SV=1 |
| 5 | 475 | tr\|A0A068FF70\|A0A068FF70\_9DIPT | 25.17 | 5 | 2 | 1 | N | 23362 | Cytochrome c oxidase subunit 1 (Fragment) OS=Simulium arcticum sensu lato LB18\_9 OX=1513970 GN=COI PE=3 SV=1 |
| 5 | 1280 | tr\|A0A068FFB2\|A0A068FFB2\_9DIPT | 25.17 | 5 | 2 | 1 | N | 23362 | Cytochrome c oxidase subunit 1 (Fragment) OS=Simulium arcticum sensu lato BL9\_23 OX=1513933 GN=COI PE=3 SV=1 |
| 5 | 476 | tr\|A0A068FF09\|A0A068FF09\_9DIPT | 25.17 | 5 | 2 | 1 | N | 23362 | Cytochrome c oxidase subunit 1 (Fragment) OS=Simulium arcticum sensu lato RC9\_6 OX=1513997 GN=COI PE=3 SV=1 |
| 5 | 1282 | tr\|A0A068FK66\|A0A068FK66\_9DIPT | 25.17 | 5 | 2 | 1 | N | 23362 | Cytochrome c oxidase subunit 1 (Fragment) OS=Simulium arcticum sensu lato LB18\_6 OX=1513967 GN=COI PE=3 SV=1 |
| 5 | 477 | tr\|A0A068FDG6\|A0A068FDG6\_9DIPT | 25.17 | 5 | 2 | 1 | N | 23362 | Cytochrome c oxidase subunit 1 (Fragment) OS=Simulium arcticum sensu lato LB18\_13 OX=1513961 GN=COI PE=3 SV=1 |
| 5 | 1283 | tr\|A0A068FFB6\|A0A068FFB6\_9DIPT | 25.17 | 5 | 2 | 1 | N | 23392 | Cytochrome c oxidase subunit 1 (Fragment) OS=Simulium arcticum sensu lato BL9\_33 OX=1513939 GN=COI PE=3 SV=1 |
| 5 | 478 | tr\|A0A068FDG0\|A0A068FDG0\_9DIPT | 25.17 | 5 | 2 | 1 | N | 23362 | Cytochrome c oxidase subunit 1 (Fragment) OS=Simulium arcticum sensu lato LB18\_8 OX=1513969 GN=COI PE=3 SV=1 |
| 5 | 1284 | tr\|A0A068FGA6\|A0A068FGA6\_9DIPT | 25.17 | 5 | 2 | 1 | N | 23362 | Cytochrome c oxidase subunit 1 (Fragment) OS=Simulium arcticum sensu lato RC9\_17 OX=1513990 GN=COI PE=3 SV=1 |
| 5 | 479 | tr\|A0A068FEF8\|A0A068FEF8\_9DIPT | 25.17 | 5 | 2 | 1 | N | 23362 | Cytochrome c oxidase subunit 1 (Fragment) OS=Simulium arcticum sensu lato BL9\_27 OX=1513934 GN=COI PE=3 SV=1 |
| 5 | 480 | tr\|A0A068FEW5\|A0A068FEW5\_9DIPT | 25.17 | 5 | 2 | 1 | N | 23362 | Cytochrome c oxidase subunit 1 (Fragment) OS=Simulium arcticum OX=390676 GN=COI PE=3 SV=1 |
| 5 | 1285 | tr\|A0A068FG98\|A0A068FG98\_9DIPT | 25.17 | 5 | 2 | 1 | N | 23362 | Cytochrome c oxidase subunit 1 (Fragment) OS=Simulium arcticum sensu lato RC9\_4 OX=1513995 GN=COI PE=3 SV=1 |
| 5 | 1286 | tr\|A0A068FK59\|A0A068FK59\_9DIPT | 25.17 | 5 | 2 | 1 | N | 23362 | Cytochrome c oxidase subunit 1 (Fragment) OS=Simulium arcticum sensu lato LB18\_1 OX=1513957 GN=COI PE=3 SV=1 |
| 5 | 481 | tr\|A0A068FDM2\|A0A068FDM2\_9DIPT | 25.17 | 5 | 2 | 1 | N | 23362 | Cytochrome c oxidase subunit 1 (Fragment) OS=Simulium arcticum sensu lato BL19\_12 OX=1513914 GN=COI PE=3 SV=1 |
| 5 | 1287 | tr\|A0A068FFC7\|A0A068FFC7\_9DIPT | 25.17 | 5 | 2 | 1 | N | 23349 | Cytochrome c oxidase subunit 1 (Fragment) OS=Simulium arcticum sensu lato BL19\_13 OX=1513915 GN=COI PE=3 SV=1 |
| 5 | 1288 | tr\|A0A068FGI6\|A0A068FGI6\_9DIPT | 25.17 | 5 | 2 | 1 | N | 23362 | Cytochrome c oxidase subunit 1 (Fragment) OS=Simulium arcticum sensu lato BL9\_4 OX=1513940 GN=COI PE=3 SV=1 |
| 5 | 482 | tr\|A0A068FE36\|A0A068FE36\_9DIPT | 25.17 | 5 | 2 | 1 | N | 23362 | Cytochrome c oxidase subunit 1 (Fragment) OS=Simulium arcticum sensu lato RC9\_7 OX=1513998 GN=COI PE=3 SV=1 |
| 5 | 483 | tr\|A0A068FD97\|A0A068FD97\_9DIPT | 25.17 | 5 | 2 | 1 | N | 23362 | Cytochrome c oxidase subunit 1 (Fragment) OS=Simulium arcticum sensu lato RC9\_5 OX=1513996 GN=COI PE=3 SV=1 |
| 5 | 484 | tr\|A0A068FDL8\|A0A068FDL8\_9DIPT | 25.17 | 5 | 2 | 1 | N | 23362 | Cytochrome c oxidase subunit 1 (Fragment) OS=Simulium arcticum sensu lato BL19\_6 OX=1513924 GN=COI PE=3 SV=1 |
| 5 | 486 | tr\|A0A068FE04\|A0A068FE04\_9DIPT | 25.17 | 5 | 2 | 1 | N | 23372 | Cytochrome c oxidase subunit 1 (Fragment) OS=Simulium apricarium OX=390680 GN=COI PE=3 SV=1 |
| 5 | 485 | tr\|A0A068FDA5\|A0A068FDA5\_9DIPT | 25.17 | 5 | 2 | 1 | N | 23362 | Cytochrome c oxidase subunit 1 (Fragment) OS=Simulium arcticum sensu lato RC9\_19 OX=1513991 GN=COI PE=3 SV=1 |
| 5 | 1290 | tr\|A0A068FG87\|A0A068FG87\_9DIPT | 25.17 | 5 | 2 | 1 | N | 23362 | Cytochrome c oxidase subunit 1 (Fragment) OS=Simulium arcticum sensu lato CW22\_3 OX=1513950 GN=COI PE=3 SV=1 |
| 5 | 1291 | tr\|A0A068FGC1\|A0A068FGC1\_9DIPT | 25.17 | 5 | 2 | 1 | N | 23344 | Cytochrome c oxidase subunit 1 (Fragment) OS=Simulium arcticum OX=390676 GN=COI PE=3 SV=1 |
| 5 | 487 | tr\|A0A068FDL0\|A0A068FDL0\_9DIPT | 25.17 | 5 | 2 | 1 | N | 23423 | Cytochrome c oxidase subunit 1 (Fragment) OS=Simulium arcticum sensu lato BL9\_19 OX=1513932 GN=COI PE=3 SV=1 |
| 5 | 488 | tr\|A0A068FDF4\|A0A068FDF4\_9DIPT | 25.17 | 5 | 2 | 1 | N | 23362 | Cytochrome c oxidase subunit 1 (Fragment) OS=Simulium arcticum sensu lato LB18\_3 OX=1513964 GN=COI PE=3 SV=1 |
| 5 | 489 | tr\|A0A068FD62\|A0A068FD62\_9DIPT | 25.17 | 5 | 2 | 1 | N | 23361 | Cytochrome c oxidase subunit 1 (Fragment) OS=Simulium arcticum OX=390676 GN=COI PE=3 SV=1 |
| 5 | 490 | tr\|A0A068FDC0\|A0A068FDC0\_9DIPT | 25.17 | 5 | 2 | 1 | N | 23362 | Cytochrome c oxidase subunit 1 (Fragment) OS=Simulium arcticum sensu lato RC19\_30 OX=1513980 GN=COI PE=3 SV=1 |
| 5 | 1292 | tr\|A0A068FKG1\|A0A068FKG1\_9DIPT | 25.17 | 5 | 2 | 1 | N | 23423 | Cytochrome c oxidase subunit 1 (Fragment) OS=Simulium arcticum sensu lato BL19\_17 OX=1513917 GN=COI PE=3 SV=1 |
| 5 | 491 | tr\|A0A068FEF2\|A0A068FEF2\_9DIPT | 25.17 | 5 | 2 | 1 | N | 23362 | Cytochrome c oxidase subunit 1 (Fragment) OS=Simulium arcticum sensu lato BL9\_11 OX=1513929 GN=COI PE=3 SV=1 |
| 5 | 492 | tr\|A0A068FEH2\|A0A068FEH2\_9DIPT | 25.17 | 5 | 2 | 1 | N | 23362 | Cytochrome c oxidase subunit 1 (Fragment) OS=Simulium arcticum sensu lato BL19\_16 OX=1513916 GN=COI PE=3 SV=1 |
| 5 | 1293 | tr\|A0A068FG94\|A0A068FG94\_9DIPT | 25.17 | 5 | 2 | 1 | N | 23362 | Cytochrome c oxidase subunit 1 (Fragment) OS=Simulium arcticum sensu lato CW22\_14 OX=1513946 GN=COI PE=3 SV=1 |
| 5 | 493 | tr\|A0A068FEZ8\|A0A068FEZ8\_9DIPT | 25.17 | 5 | 2 | 1 | N | 23362 | Cytochrome c oxidase subunit 1 (Fragment) OS=Simulium arcticum sensu lato CW22\_5 OX=1513952 GN=COI PE=3 SV=1 |
| 5 | 1294 | tr\|A0A068FK19\|A0A068FK19\_9DIPT | 25.17 | 5 | 2 | 1 | N | 23362 | Cytochrome c oxidase subunit 1 (Fragment) OS=Simulium arcticum sensu lato RC19\_24 OX=1513977 GN=COI PE=3 SV=1 |
| 5 | 494 | tr\|A0A068FEC3\|A0A068FEC3\_9DIPT | 25.17 | 5 | 2 | 1 | N | 23378 | Cytochrome c oxidase subunit 1 (Fragment) OS=Simulium arcticum OX=390676 GN=COI PE=3 SV=1 |
| 5 | 495 | tr\|A0A068FF05\|A0A068FF05\_9DIPT | 25.17 | 5 | 2 | 1 | N | 23362 | Cytochrome c oxidase subunit 1 (Fragment) OS=Simulium arcticum sensu lato CW22\_16 OX=1513948 GN=COI PE=3 SV=1 |
| 5 | 496 | tr\|A0A068FF65\|A0A068FF65\_9DIPT | 25.17 | 5 | 2 | 1 | N | 23362 | Cytochrome c oxidase subunit 1 (Fragment) OS=Simulium arcticum sensu lato LB18\_4 OX=1513965 GN=COI PE=3 SV=1 |
| 5 | 1397 | tr\|A0A0G2STG6\|A0A0G2STG6\_9DIPT | 25.17 | 4 | 2 | 1 | N | 23975 | Cytochrome c oxidase subunit 1 (Fragment) OS=Simulium timorense OX=1642037 GN=COI PE=3 SV=1 |
| 5 | 1398 | tr\|A0A0G2STG2\|A0A0G2STG2\_9DIPT | 25.17 | 4 | 2 | 1 | N | 23975 | Cytochrome c oxidase subunit 1 (Fragment) OS=Simulium nodosum OX=236047 GN=COI PE=3 SV=1 |
| 5 | 1399 | tr\|A0A0G2STA7\|A0A0G2STA7\_9DIPT | 25.17 | 4 | 2 | 1 | N | 23975 | Cytochrome c oxidase subunit 1 (Fragment) OS=Simulium nobile OX=210187 GN=COI PE=3 SV=1 |
| 5 | 1400 | tr\|A0A0G2STC9\|A0A0G2STC9\_9DIPT | 25.17 | 4 | 2 | 1 | N | 23975 | Cytochrome c oxidase subunit 1 (Fragment) OS=Simulium shirakii OX=1642036 GN=COI PE=3 SV=1 |
| 5 | 1401 | tr\|A0A0G2STC8\|A0A0G2STC8\_9DIPT | 25.17 | 4 | 2 | 1 | N | 24007 | Cytochrome c oxidase subunit 1 (Fragment) OS=Simulium nodosum OX=236047 GN=COI PE=3 SV=1 |
| 5 | 1403 | tr\|A0A0F6PRQ8\|A0A0F6PRQ8\_9DIPT | 25.17 | 4 | 2 | 1 | N | 24087 | Cytochrome c oxidase subunit 1 (Fragment) OS=Simulium sofiani OX=1642034 GN=COI PE=3 SV=1 |
| 5 | 1404 | tr\|A0A0F6PPG4\|A0A0F6PPG4\_9DIPT | 25.17 | 4 | 2 | 1 | N | 24087 | Cytochrome c oxidase subunit 1 (Fragment) OS=Simulium leparense OX=1642031 GN=COI PE=3 SV=1 |
| 5 | 1405 | tr\|A0A0F6PRP5\|A0A0F6PRP5\_9DIPT | 25.17 | 4 | 2 | 1 | N | 24087 | Cytochrome c oxidase subunit 1 (Fragment) OS=Simulium asakoae OX=333336 GN=COI PE=3 SV=1 |
| 5 | 1406 | tr\|A0A0F6PQP9\|A0A0F6PQP9\_9DIPT | 25.17 | 4 | 2 | 1 | N | 24087 | Cytochrome c oxidase subunit 1 (Fragment) OS=Simulium trangense OX=866626 GN=COI PE=3 SV=1 |
| 5 | 1407 | tr\|A0A0F6PRN0\|A0A0F6PRN0\_9DIPT | 25.17 | 4 | 2 | 1 | N | 24091 | Cytochrome c oxidase subunit 1 (Fragment) OS=Simulium tani OX=210195 GN=COI PE=3 SV=1 |
| 5 | 1408 | tr\|A0A0F6PRR4\|A0A0F6PRR4\_9DIPT | 25.17 | 4 | 2 | 1 | N | 24087 | Cytochrome c oxidase subunit 1 (Fragment) OS=Simulium sheilae OX=236038 GN=COI PE=3 SV=1 |
| 5 | 1409 | tr\|A0A0F6PQP5\|A0A0F6PQP5\_9DIPT | 25.17 | 4 | 2 | 1 | N | 24087 | Cytochrome c oxidase subunit 1 (Fragment) OS=Simulium lurauense OX=1642032 GN=COI PE=3 SV=1 |
| 5 | 1410 | tr\|A0A0F6PPF2\|A0A0F6PPF2\_9DIPT | 25.17 | 4 | 2 | 1 | N | 24087 | Cytochrome c oxidase subunit 1 (Fragment) OS=Simulium brinchangense OX=1642029 GN=COI PE=3 SV=1 |
| 5 | 1411 | tr\|A0A0F6PQN3\|A0A0F6PQN3\_9DIPT | 25.17 | 4 | 2 | 1 | N | 24087 | Cytochrome c oxidase subunit 1 (Fragment) OS=Simulium izuae OX=1642030 GN=COI PE=3 SV=1 |
| 5 | 1412 | tr\|A0A0F6PQN7\|A0A0F6PQN7\_9DIPT | 25.17 | 4 | 2 | 1 | N | 24087 | Cytochrome c oxidase subunit 1 (Fragment) OS=Simulium tanahrataense OX=1642035 GN=COI PE=3 SV=1 |
| 5 | 1413 | tr\|A0A0F6PRN6\|A0A0F6PRN6\_9DIPT | 25.17 | 4 | 2 | 1 | N | 24087 | Cytochrome c oxidase subunit 1 (Fragment) OS=Simulium roslihashimi OX=1642033 GN=COI PE=3 SV=1 |
| 5 | 1437 | tr\|A0A077D6R9\|A0A077D6R9\_9DIPT | 25.17 | 4 | 2 | 1 | N | 25355 | Cytochrome c oxidase subunit 1 (Fragment) OS=Simulium bezzii OX=1419335 GN=COI PE=3 SV=1 |
| 5 | 1438 | tr\|A0A077D482\|A0A077D482\_9DIPT | 25.17 | 4 | 2 | 1 | N | 25325 | Cytochrome c oxidase subunit 1 (Fragment) OS=Simulium bezzii OX=1419335 GN=COI PE=3 SV=1 |
| 5 | 1439 | tr\|A0A077D9S9\|A0A077D9S9\_9DIPT | 25.17 | 4 | 2 | 1 | N | 25349 | Cytochrome c oxidase subunit 1 (Fragment) OS=Simulium velutinum OX=716703 GN=COI PE=3 SV=1 |
| 5 | 1440 | tr\|A0A077D8K9\|A0A077D8K9\_9DIPT | 25.17 | 4 | 2 | 1 | N | 25325 | Cytochrome c oxidase subunit 1 (Fragment) OS=Simulium ornatum OX=304233 GN=COI PE=3 SV=1 |
| 5 | 1717 | tr\|A0A0N9FUK8\|A0A0N9FUK8\_9DIPT | 25.17 | 6 | 2 | 1 | N | 18259 | Cytochrome c oxidase subunit 1 (Fragment) OS=Prosimulium mixtum OX=994300 GN=COI PE=3 SV=1 |
| 5 | 1718 | tr\|A0A0N7HMJ8\|A0A0N7HMJ8\_9DIPT | 25.17 | 6 | 2 | 1 | N | 18635 | Cytochrome c oxidase subunit 1 (Fragment) OS=Simulium sp. BOLD:AAA1697 OX=1695072 GN=COI PE=3 SV=1 |
| 5 | 891 | tr\|A0A0N9FT07\|A0A0N9FT07\_SIMVI | 25.17 | 6 | 2 | 1 | N | 18779 | Cytochrome c oxidase subunit 1 (Fragment) OS=Simulium vittatum OX=7192 GN=COI PE=3 SV=1 |
| 5 | 1719 | tr\|A0A0N9FVD5\|A0A0N9FVD5\_SIMVI | 25.17 | 6 | 2 | 1 | N | 18859 | Cytochrome c oxidase subunit 1 (Fragment) OS=Simulium vittatum OX=7192 GN=COI PE=3 SV=1 |
| 5 | 1720 | tr\|A0A0N9G0S6\|A0A0N9G0S6\_SIMVI | 25.17 | 6 | 2 | 1 | N | 19095 | Cytochrome c oxidase subunit 1 (Fragment) OS=Simulium vittatum OX=7192 GN=COI PE=3 SV=1 |
| 5 | 1721 | tr\|A0A0N9FRZ2\|A0A0N9FRZ2\_SIMVI | 25.17 | 6 | 2 | 1 | N | 18993 | Cytochrome c oxidase subunit 1 (Fragment) OS=Simulium vittatum OX=7192 GN=COI PE=3 SV=1 |
| 5 | 900 | tr\|A0A0N9FVN2\|A0A0N9FVN2\_SIMVI | 25.17 | 6 | 2 | 1 | N | 19152 | Cytochrome c oxidase subunit 1 (Fragment) OS=Simulium vittatum OX=7192 GN=COI PE=3 SV=1 |
| 5 | 901 | tr\|A0A0N9FRW7\|A0A0N9FRW7\_SIMVI | 25.17 | 6 | 2 | 1 | N | 19123 | Cytochrome c oxidase subunit 1 (Fragment) OS=Simulium vittatum OX=7192 GN=COI PE=3 SV=1 |
| 5 | 1723 | tr\|A0A0N9G226\|A0A0N9G226\_SIMVI | 25.17 | 6 | 2 | 1 | N | 19152 | Cytochrome c oxidase subunit 1 (Fragment) OS=Simulium vittatum OX=7192 GN=COI PE=3 SV=1 |
| 5 | 1724 | tr\|A0A0N7HQ75\|A0A0N7HQ75\_9DIPT | 25.17 | 6 | 2 | 1 | N | 19065 | Cytochrome c oxidase subunit 1 (Fragment) OS=Simulium sp. BOLD:AAA1697 OX=1695072 GN=COI PE=3 SV=1 |
| 5 | 906 | tr\|A0A0P0B2U5\|A0A0P0B2U5\_9DIPT | 25.17 | 5 | 2 | 1 | N | 19297 | Cytochrome c oxidase subunit 1 (Fragment) OS=Simulium sp. BOLD:AAA1697 OX=1695072 GN=COI PE=3 SV=1 |
| 5 | 909 | tr\|A0A0N9G394\|A0A0N9G394\_SIMVI | 25.17 | 5 | 2 | 1 | N | 19382 | Cytochrome c oxidase subunit 1 (Fragment) OS=Simulium vittatum OX=7192 GN=COI PE=3 SV=1 |
| 5 | 913 | tr\|A0A0N9G330\|A0A0N9G330\_SIMVI | 25.17 | 5 | 2 | 1 | N | 19495 | Cytochrome c oxidase subunit 1 (Fragment) OS=Simulium vittatum OX=7192 GN=COI PE=3 SV=1 |
| 5 | 914 | tr\|A0A0N9FZW7\|A0A0N9FZW7\_SIMVI | 25.17 | 5 | 2 | 1 | N | 19568 | Cytochrome c oxidase subunit 1 (Fragment) OS=Simulium vittatum OX=7192 GN=COI PE=3 SV=1 |
| 5 | 918 | tr\|A0A0N9G108\|A0A0N9G108\_SIMVI | 25.17 | 5 | 2 | 1 | N | 19682 | Cytochrome c oxidase subunit 1 (Fragment) OS=Simulium vittatum OX=7192 GN=COI PE=3 SV=1 |
| 5 | 924 | tr\|A0A0N9FZA3\|A0A0N9FZA3\_SIMVI | 25.17 | 5 | 2 | 1 | N | 19777 | Cytochrome c oxidase subunit 1 (Fragment) OS=Simulium vittatum OX=7192 GN=COI PE=3 SV=1 |
| 5 | 929 | tr\|A0A0N9FXU3\|A0A0N9FXU3\_SIMVI | 25.17 | 5 | 2 | 1 | N | 19825 | Cytochrome c oxidase subunit 1 (Fragment) OS=Simulium vittatum OX=7192 GN=COI PE=3 SV=1 |
| 5 | 935 | tr\|A0A0N9G1J3\|A0A0N9G1J3\_SIMVI | 25.17 | 5 | 2 | 1 | N | 20011 | Cytochrome c oxidase subunit 1 (Fragment) OS=Simulium vittatum OX=7192 GN=COI PE=3 SV=1 |
| 5 | 937 | tr\|A0A0N9FYE2\|A0A0N9FYE2\_SIMVI | 25.17 | 5 | 2 | 1 | N | 19957 | Cytochrome c oxidase subunit 1 (Fragment) OS=Simulium vittatum OX=7192 GN=COI PE=3 SV=1 |
| 5 | 960 | tr\|A0A0P0AVW9\|A0A0P0AVW9\_9DIPT | 25.17 | 5 | 2 | 1 | N | 20270 | Cytochrome c oxidase subunit 1 (Fragment) OS=Simulium sp. BOLD:AAA1697 OX=1695072 GN=COI PE=3 SV=1 |
| 5 | 961 | tr\|A0A0R6MKB5\|A0A0R6MKB5\_9DIPT | 25.17 | 5 | 2 | 1 | N | 20446 | Cytochrome c oxidase subunit 1 (Fragment) OS=Simulium metallicum s.l. BOLD:ACF2593 OX=1653141 GN=COI PE=3 SV=1 |
| 5 | 983 | tr\|A0A0N9FUS4\|A0A0N9FUS4\_SIMVI | 25.17 | 5 | 2 | 1 | N | 20487 | Cytochrome c oxidase subunit 1 (Fragment) OS=Simulium vittatum OX=7192 GN=COI PE=3 SV=1 |
| 5 | 999 | tr\|A0A0P0BTU2\|A0A0P0BTU2\_9DIPT | 25.17 | 5 | 2 | 1 | N | 20542 | Cytochrome c oxidase subunit 1 (Fragment) OS=Simulium sp. BOLD:AAA4264 OX=1695073 GN=COI PE=3 SV=1 |
| 5 | 1000 | tr\|A0A0N7ESR4\|A0A0N7ESR4\_9DIPT | 25.17 | 5 | 2 | 1 | N | 20556 | Cytochrome c oxidase subunit 1 (Fragment) OS=Prosimulium arvum OX=626814 GN=COI PE=3 SV=1 |
| 5 | 1072 | tr\|A0A0H3VZQ4\|A0A0H3VZQ4\_9DIPT | 25.17 | 5 | 2 | 1 | N | 21198 | Cytochrome c oxidase subunit 1 (Fragment) OS=Simulium arcticum sensu lato CAhyb2 OX=1654725 GN=COI PE=3 SV=1 |
| 5 | 1073 | tr\|A0A0P0AR86\|A0A0P0AR86\_9DIPT | 25.17 | 5 | 2 | 1 | N | 21425 | Cytochrome c oxidase subunit 1 (Fragment) OS=Simulium sp. BOLD:AAA1697 OX=1695072 GN=COI PE=3 SV=1 |
| 5 | 1086 | tr\|A0A0P0A1S1\|A0A0P0A1S1\_9DIPT | 25.17 | 5 | 2 | 1 | N | 21761 | Cytochrome c oxidase subunit 1 (Fragment) OS=Simulium sp. BOLD:AAA1697 OX=1695072 GN=COI PE=3 SV=1 |
| 5 | 1093 | tr\|A0A0R6MN67\|A0A0R6MN67\_9DIPT | 25.17 | 5 | 2 | 1 | N | 21783 | Cytochrome c oxidase subunit 1 (Fragment) OS=Simulium metallicum s.l. BOLD:ACF2593 OX=1653141 GN=COI PE=3 SV=1 |
| 5 | 1100 | tr\|A0A0R6MJZ1\|A0A0R6MJZ1\_9DIPT | 25.17 | 5 | 2 | 1 | N | 21870 | Cytochrome c oxidase subunit 1 (Fragment) OS=Simulium metallicum s.l. BOLD:ACF2593 OX=1653141 GN=COI PE=3 SV=1 |
| 5 | 1126 | tr\|A0A0H3W048\|A0A0H3W048\_9DIPT | 25.17 | 5 | 2 | 1 | N | 21772 | Cytochrome c oxidase subunit 1 (Fragment) OS=Simulium arcticum sensu lato CAhyb17 OX=1654724 GN=COI PE=3 SV=1 |
| 5 | 1130 | tr\|A0A0H3W0E2\|A0A0H3W0E2\_9DIPT | 25.17 | 5 | 2 | 1 | N | 21796 | Cytochrome c oxidase subunit 1 (Fragment) OS=Simulium arcticum OX=390676 GN=COI PE=3 SV=1 |
| 5 | 1134 | tr\|A0A0H3W044\|A0A0H3W044\_9DIPT | 25.17 | 5 | 2 | 1 | N | 21881 | Cytochrome c oxidase subunit 1 (Fragment) OS=Simulium saxosum OX=390679 GN=COI PE=3 SV=1 |
| 5 | 1137 | tr\|A0A0H3W046\|A0A0H3W046\_9DIPT | 25.17 | 5 | 2 | 1 | N | 21772 | Cytochrome c oxidase subunit 1 (Fragment) OS=Simulium arcticum sensu lato CAhyb4 OX=1654731 GN=COI PE=3 SV=1 |
| 5 | 1139 | tr\|A0A0H3VZQ5\|A0A0H3VZQ5\_9DIPT | 25.17 | 5 | 2 | 1 | N | 21772 | Cytochrome c oxidase subunit 1 (Fragment) OS=Simulium arcticum sensu lato CAhyb13 OX=1654723 GN=COI PE=3 SV=1 |
| 5 | 1140 | tr\|A0A0H3W049\|A0A0H3W049\_9DIPT | 25.17 | 5 | 2 | 1 | N | 21772 | Cytochrome c oxidase subunit 1 (Fragment) OS=Simulium arcticum sensu lato CAhyb37 OX=1654730 GN=COI PE=3 SV=1 |
| 5 | 1143 | tr\|A0A0H3W0E7\|A0A0H3W0E7\_9DIPT | 25.17 | 5 | 2 | 1 | N | 21772 | Cytochrome c oxidase subunit 1 (Fragment) OS=Simulium arcticum sensu lato CAhyb30 OX=1654727 GN=COI PE=3 SV=1 |
| 5 | 1145 | tr\|A0A0H3VZQ1\|A0A0H3VZQ1\_9DIPT | 25.17 | 5 | 2 | 1 | N | 21762 | Cytochrome c oxidase subunit 1 (Fragment) OS=Simulium saxosum OX=390679 GN=COI PE=3 SV=1 |
| 5 | 1146 | tr\|A0A0H3W051\|A0A0H3W051\_9DIPT | 25.17 | 5 | 2 | 1 | N | 21772 | Cytochrome c oxidase subunit 1 (Fragment) OS=Simulium saxosum OX=390679 GN=COI PE=3 SV=1 |
| 5 | 1147 | tr\|A0A0H3VZQ6\|A0A0H3VZQ6\_9DIPT | 25.17 | 5 | 2 | 1 | N | 21772 | Cytochrome c oxidase subunit 1 (Fragment) OS=Simulium arcticum sensu lato CAhyb35 OX=1654729 GN=COI PE=3 SV=1 |
| 5 | 1148 | tr\|A0A0H3W0E4\|A0A0H3W0E4\_9DIPT | 25.17 | 5 | 2 | 1 | N | 21820 | Cytochrome c oxidase subunit 1 (Fragment) OS=Simulium saxosum OX=390679 GN=COI PE=3 SV=1 |
| 5 | 1150 | tr\|A0A0H3W0R9\|A0A0H3W0R9\_9DIPT | 25.17 | 5 | 2 | 1 | N | 21772 | Cytochrome c oxidase subunit 1 (Fragment) OS=Simulium arcticum sensu lato CAhyb47 OX=1654734 GN=COI PE=3 SV=1 |
| 5 | 1151 | tr\|A0A0H3W0E8\|A0A0H3W0E8\_9DIPT | 25.17 | 5 | 2 | 1 | N | 21772 | Cytochrome c oxidase subunit 1 (Fragment) OS=Simulium arcticum sensu lato CAhyb42 OX=1654733 GN=COI PE=3 SV=1 |
| 5 | 1154 | tr\|A0A0H3W037\|A0A0H3W037\_9DIPT | 25.17 | 5 | 2 | 1 | N | 21759 | Cytochrome c oxidase subunit 1 (Fragment) OS=Simulium arcticum OX=390676 GN=COI PE=3 SV=1 |
| 5 | 1157 | tr\|A0A0R6MM04\|A0A0R6MM04\_9DIPT | 25.17 | 5 | 2 | 1 | N | 22127 | Cytochrome c oxidase subunit 1 (Fragment) OS=Simulium metallicum s.l. BOLD:ACF2593 OX=1653141 GN=COI PE=3 SV=1 |
| 5 | 1178 | tr\|A0A0H3W0E6\|A0A0H3W0E6\_9DIPT | 25.17 | 5 | 2 | 1 | N | 21998 | Cytochrome c oxidase subunit 1 (Fragment) OS=Simulium arcticum sensu lato CAhyb10 OX=1654721 GN=COI PE=3 SV=1 |
| 5 | 1193 | tr\|A0A0P0AJX7\|A0A0P0AJX7\_9DIPT | 25.17 | 5 | 2 | 1 | N | 22363 | Cytochrome c oxidase subunit 1 (Fragment) OS=Simulium sp. BOLD:AAA1697 OX=1695072 GN=COI PE=3 SV=1 |
| 5 | 1217 | tr\|A0A0R6MM60\|A0A0R6MM60\_9DIPT | 25.17 | 5 | 2 | 1 | N | 22644 | Cytochrome c oxidase subunit 1 (Fragment) OS=Simulium paynei s.l. BOLD:AAC4424 OX=1653166 GN=COI PE=3 SV=1 |
| 5 | 1238 | tr\|A0A0K0PVD6\|A0A0K0PVD6\_9DIPT | 25.17 | 5 | 2 | 1 | N | 23002 | Cytochrome c oxidase subunit 1 (Fragment) OS=Simulium panagudiense OX=1564295 GN=COI PE=3 SV=1 |
| 5 | 1266 | tr\|A0A068FGJ4\|A0A068FGJ4\_9DIPT | 25.17 | 5 | 2 | 1 | N | 23403 | Cytochrome c oxidase subunit 1 (Fragment) OS=Simulium arcticum sensu lato BL9\_14 OX=1513931 GN=COI PE=3 SV=1 |
| 5 | 1281 | tr\|A0A0H3W042\|A0A0H3W042\_9DIPT | 25.17 | 5 | 2 | 1 | N | 23362 | Cytochrome c oxidase subunit 1 (Fragment) OS=Simulium saxosum OX=390679 GN=COI PE=3 SV=1 |
| 5 | 1289 | tr\|A0A0H3W0R7\|A0A0H3W0R7\_9DIPT | 25.17 | 5 | 2 | 1 | N | 23362 | Cytochrome c oxidase subunit 1 (Fragment) OS=Simulium arcticum sensu lato CAhyb12 OX=1654722 GN=COI PE=3 SV=1 |
| 5 | 1433 | tr\|A0A0H4SZ59\|A0A0H4SZ59\_9DIPT | 25.17 | 4 | 2 | 1 | N | 24537 | Cytochrome c oxidase subunit 1 (Fragment) OS=Simulium peteri OX=1537664 GN=COI PE=3 SV=1 |
| 7 | 854 | tr\|A0A023J419\|A0A023J419\_9DIPT | 25.12 | 2 | 2 | 2 | N | 72920 | 5intG (Fragment) OS=Simulium chlorum OX=1028768 PE=4 SV=1 |
| 7 | 406 | tr\|A0A023J489\|A0A023J489\_9DIPT | 25.12 | 1 | 2 | 2 | N | 72627 | 5intG (Fragment) OS=Simulium chlorum OX=1028768 PE=4 SV=1 |
| 7 | 3657 | tr\|A0A023J3Z8\|A0A023J3Z8\_9DIPT | 25.12 | 2 | 2 | 2 | N | 72637 | 5intG (Fragment) OS=Simulium dixiense OX=357426 PE=4 SV=1 |
| 7 | 3671 | tr\|A0A023J469\|A0A023J469\_9DIPT | 25.12 | 1 | 2 | 2 | N | 72876 | 5intG (Fragment) OS=Simulium fibrinflatum OX=1028770 PE=4 SV=1 |
| 9 | 61570 | tr\|B5M0P8\|B5M0P8\_SIMVI | 24.09 | 10 | 2 | 2 | N | 11139 | Hypothetical secreted protein OS=Simulium vittatum OX=7192 PE=2 SV=1 |
| 4 | 1763 | tr\|A0A023GYN2\|A0A023GYN2\_9DIPT | 22.47 | 1 | 1 | 1 | N | 75260 | Big zinc finger (Fragment) OS=Simulium infenestrum OX=1028772 PE=4 SV=1 |
| 4 | 1761 | tr\|A0A023GYR7\|A0A023GYR7\_9DIPT | 22.47 | 1 | 1 | 1 | N | 75311 | Big zinc finger (Fragment) OS=Simulium podostemi OX=1028778 PE=4 SV=1 |
| 4 | 1762 | tr\|A0A023GYQ2\|A0A023GYQ2\_9DIPT | 22.47 | 1 | 1 | 1 | N | 75341 | Big zinc finger (Fragment) OS=Simulium podostemi OX=1028778 PE=4 SV=1 |
| 4 | 1765 | tr\|A0A023GYL6\|A0A023GYL6\_9DIPT | 22.47 | 1 | 1 | 1 | N | 75282 | Big zinc finger (Fragment) OS=Simulium fibrinflatum OX=1028770 PE=4 SV=1 |
| 4 | 1766 | tr\|A0A023GYN6\|A0A023GYN6\_9DIPT | 22.47 | 1 | 1 | 1 | N | 75274 | Big zinc finger (Fragment) OS=Simulium penobscotense OX=1028777 PE=4 SV=1 |
| 4 | 1767 | tr\|A0A023GYR1\|A0A023GYR1\_9DIPT | 22.47 | 1 | 1 | 1 | N | 75275 | Big zinc finger (Fragment) OS=Simulium fibrinflatum OX=1028770 PE=4 SV=1 |
| 4 | 1769 | tr\|A0A023GYR8\|A0A023GYR8\_9DIPT | 22.47 | 1 | 1 | 1 | N | 75394 | Big zinc finger (Fragment) OS=Simulium nyssa OX=40478 PE=4 SV=1 |
| 4 | 1770 | tr\|A0A023GYL2\|A0A023GYL2\_9DIPT | 22.47 | 1 | 1 | 1 | N | 75310 | Big zinc finger (Fragment) OS=Simulium anchistinum OX=1028766 PE=4 SV=1 |
| 4 | 1771 | tr\|A0A023GYM4\|A0A023GYM4\_9DIPT | 22.47 | 1 | 1 | 1 | N | 75254 | Big zinc finger (Fragment) OS=Simulium snowi OX=1028780 PE=4 SV=1 |
| 4 | 1772 | tr\|A0A023GYN1\|A0A023GYN1\_9DIPT | 22.47 | 1 | 1 | 1 | N | 75284 | Big zinc finger (Fragment) OS=Simulium fibrinflatum OX=1028770 PE=4 SV=1 |
| 4 | 1773 | tr\|A0A023GYQ5\|A0A023GYQ5\_9DIPT | 22.47 | 1 | 1 | 1 | N | 75368 | Big zinc finger (Fragment) OS=Simulium fibrinflatum OX=1028770 PE=4 SV=1 |
| 4 | 1775 | tr\|A0A023GYP6\|A0A023GYP6\_9DIPT | 22.47 | 1 | 1 | 1 | N | 75292 | Big zinc finger (Fragment) OS=Simulium fibrinflatum OX=1028770 PE=4 SV=1 |
| 4 | 1776 | tr\|A0A023GYM1\|A0A023GYM1\_9DIPT | 22.47 | 1 | 1 | 1 | N | 75385 | Big zinc finger (Fragment) OS=Simulium penobscotense OX=1028777 PE=3 SV=1 |
| 4 | 1777 | tr\|A0A023GYT1\|A0A023GYT1\_9DIPT | 22.47 | 1 | 1 | 1 | N | 75363 | Big zinc finger (Fragment) OS=Simulium penobscotense OX=1028777 PE=4 SV=1 |
| 4 | 1778 | tr\|A0A023GYQ7\|A0A023GYQ7\_9DIPT | 22.47 | 1 | 1 | 1 | N | 75410 | Big zinc finger (Fragment) OS=Simulium anchistinum OX=1028766 PE=4 SV=1 |
| 4 | 1779 | tr\|A0A023GYN7\|A0A023GYN7\_9DIPT | 22.47 | 1 | 1 | 1 | N | 75314 | Big zinc finger (Fragment) OS=Simulium notiale OX=1028775 PE=3 SV=1 |
| 4 | 1780 | tr\|A0A023GYS0\|A0A023GYS0\_9DIPT | 22.47 | 1 | 1 | 1 | N | 75302 | Big zinc finger (Fragment) OS=Simulium fibrinflatum OX=1028770 PE=4 SV=1 |
| 4 | 1781 | tr\|A0A023GYT3\|A0A023GYT3\_9DIPT | 22.47 | 1 | 1 | 1 | N | 75316 | Big zinc finger (Fragment) OS=Simulium nyssa OX=40478 PE=4 SV=1 |
| 4 | 1782 | tr\|A0A023GYM5\|A0A023GYM5\_9DIPT | 22.47 | 1 | 1 | 1 | N | 75284 | Big zinc finger (Fragment) OS=Simulium fibrinflatum OX=1028770 PE=3 SV=1 |
| 4 | 1783 | tr\|A0A023GYL7\|A0A023GYL7\_9DIPT | 22.47 | 1 | 1 | 1 | N | 75287 | Big zinc finger (Fragment) OS=Simulium infenestrum OX=1028772 PE=4 SV=1 |
| 4 | 1784 | tr\|A0A023GYN8\|A0A023GYN8\_9DIPT | 22.47 | 1 | 1 | 1 | N | 75300 | Big zinc finger (Fragment) OS=Simulium nyssa OX=40478 PE=4 SV=1 |
| 4 | 1785 | tr\|A0A023GYM3\|A0A023GYM3\_9DIPT | 22.47 | 1 | 1 | 1 | N | 75364 | Big zinc finger (Fragment) OS=Simulium nyssa OX=40478 PE=3 SV=1 |
| 4 | 1786 | tr\|A0A023GYT2\|A0A023GYT2\_9DIPT | 22.47 | 1 | 1 | 1 | N | 75268 | Big zinc finger (Fragment) OS=Simulium notiale OX=1028775 PE=4 SV=1 |
| 4 | 1788 | tr\|A0A023GYP8\|A0A023GYP8\_9DIPT | 22.47 | 1 | 1 | 1 | N | 75471 | Big zinc finger (Fragment) OS=Simulium jenningsi OX=357422 PE=4 SV=1 |
| 4 | 1789 | tr\|A0A023GYR3\|A0A023GYR3\_9DIPT | 22.47 | 1 | 1 | 1 | N | 75385 | Big zinc finger (Fragment) OS=Simulium jenningsi OX=357422 PE=4 SV=1 |
| 4 | 1790 | tr\|A0A023GYQ3\|A0A023GYQ3\_9DIPT | 22.47 | 1 | 1 | 1 | N | 75319 | Big zinc finger (Fragment) OS=Simulium notiale OX=1028775 PE=3 SV=1 |
| 4 | 1791 | tr\|A0A023GYL8\|A0A023GYL8\_9DIPT | 22.47 | 1 | 1 | 1 | N | 75467 | Big zinc finger (Fragment) OS=Simulium jenningsi OX=357422 PE=4 SV=1 |
| 4 | 1768 | tr\|A0A023GYS5\|A0A023GYS5\_9DIPT | 22.47 | 1 | 1 | 1 | N | 75315 | Big zinc finger (Fragment) OS=Simulium fibrinflatum OX=1028770 PE=4 SV=1 |
| 4 | 1774 | tr\|A0A023GYP0\|A0A023GYP0\_9DIPT | 22.47 | 1 | 1 | 1 | N | 75481 | Big zinc finger (Fragment) OS=Simulium anchistinum OX=1028766 PE=4 SV=1 |
| 4 | 1787 | tr\|A0A023GYM2\|A0A023GYM2\_9DIPT | 22.47 | 1 | 1 | 1 | N | 75432 | Big zinc finger (Fragment) OS=Simulium podostemi OX=1028778 PE=4 SV=1 |
| 4 | 1764 | tr\|A0A023GYS9\|A0A023GYS9\_9DIPT | 22.47 | 1 | 1 | 1 | N | 75401 | Big zinc finger (Fragment) OS=Simulium lakei OX=1028774 PE=4 SV=1 |
| total 441 proteins |  |  |  |  |  |  |  |  |  |
| --- | --- | --- | --- | --- | --- | --- | --- | --- | --- |

  

SpVaP\_mature

back to list

  

| Protein Coverage
| Supporting Peptides
|

Protein Coverage:

Supporting Peptides:

| Peptide | Uniq | -10lgP | Mass | ppm | m/z | z | RT | Scan | #Spec | Start | End | PTM |
| --- | --- | --- | --- | --- | --- | --- | --- | --- | --- | --- | --- | --- |
| K.AGHIYQFYLYM(+15.99)VPK.D | Y | 58.26 | 1744.8646 | -45.3 | 873.4000 | 2 | 49.45 | 1526 | 2 | 23 | 36 | Oxidation (M) |
| R.FKSDGNGKYDLASK.G | Y | 52.55 | 1528.7521 | -41.8 | 510.5700 | 3 | 24.66 | 676 | 4 | 89 | 102 |  |
| K.SVGNDYYTLK.N | Y | 47.43 | 1158.5557 | -43.3 | 580.2600 | 2 | 31.14 | 898 | 6 | 49 | 58 |  |
| K.TDGVRDQFR.F | Y | 45.22 | 1092.5312 | -41.9 | 547.2500 | 2 | 21.51 | 568 | 5 | 80 | 88 |  |
| R.SRGEGLSVAK.D | Y | 35.66 | 1002.5458 | 877.4 | 502.7200 | 2 | 19.64 | 504 | 4 | 109 | 118 |  |
| K.SVGNDYYTLKNK.H | Y | 29.83 | 1400.6936 | -34.4 | 701.3300 | 2 | 28.22 | 798 | 2 | 49 | 60 |  |
| K.DADEHYFTIK.E | Y | 27.38 | 1237.5615 | -29.1 | 619.7700 | 2 | 33.53 | 980 | 2 | 119 | 128 |  |
| K.SVGNDYYTLKNKHSGR.F | Y | 22.83 | 1837.9071 | -37.5 | 613.6200 | 3 | 26.18 | 728 | 2 | 49 | 64 |  |
| R.FMTYDRR.A | Y | 22.27 | 987.4596 | -34.6 | 494.7200 | 2 | 23.73 | 644 | 1 | 65 | 71 |  |
| D.GVRDQFR.F | Y | 21.13 | 876.4565 | -35.5 | 439.2200 | 2 | 21.86 | 580 | 1 | 82 | 88 |  |
| R.AYFHTDSK.T | Y | 19.51 | 967.4399 | 625.9 | 485.0300 | 2 | 19.41 | 496 | 4 | 72 | 79 |  |
| K.SDGNG.K | Y | 19.08 | 448.1554 | -2014.2 | 448.2600 | 1 | 65.32 | 2070 | 1 | 91 | 95 |  |
| total 12 peptides |  |  |  |  |  |  |  |  |  |  |  |  |
| --- | --- | --- | --- | --- | --- | --- | --- | --- | --- | --- | --- | --- |

SVEP

back to list

  

| Protein Coverage
| Supporting Peptides
|

Protein Coverage:

Supporting Peptides:

| Peptide | Uniq | -10lgP | Mass | ppm | m/z | z | RT | Scan | #Spec | Start | End | PTM |
| --- | --- | --- | --- | --- | --- | --- | --- | --- | --- | --- | --- | --- |
| K.AGHIYQFYLYM(+15.99)VPK.D | Y | 58.26 | 1744.8646 | -45.3 | 873.4000 | 2 | 49.45 | 1526 | 2 | 50 | 63 | Oxidation (M) |
| R.FKSDGNGKYDLASK.G | Y | 52.55 | 1528.7521 | -41.8 | 510.5700 | 3 | 24.66 | 676 | 4 | 116 | 129 |  |
| K.SVGNDYYTLK.N | Y | 47.43 | 1158.5557 | -43.3 | 580.2600 | 2 | 31.14 | 898 | 6 | 76 | 85 |  |
| K.TDGVRDQFR.F | Y | 45.22 | 1092.5312 | -41.9 | 547.2500 | 2 | 21.51 | 568 | 5 | 107 | 115 |  |
| R.SRGEGLSVAK.D | Y | 35.66 | 1002.5458 | 877.4 | 502.7200 | 2 | 19.64 | 504 | 4 | 136 | 145 |  |
| K.SVGNDYYTLKNK.H | Y | 29.83 | 1400.6936 | -34.4 | 701.3300 | 2 | 28.22 | 798 | 2 | 76 | 87 |  |
| K.DADEHYFTIK.E | Y | 27.38 | 1237.5615 | -29.1 | 619.7700 | 2 | 33.53 | 980 | 2 | 146 | 155 |  |
| K.SVGNDYYTLKNKHSGR.F | Y | 22.83 | 1837.9071 | -37.5 | 613.6200 | 3 | 26.18 | 728 | 2 | 76 | 91 |  |
| R.FMTYDRR.A | Y | 22.27 | 987.4596 | -34.6 | 494.7200 | 2 | 23.73 | 644 | 1 | 92 | 98 |  |
| D.GVRDQFR.F | Y | 21.13 | 876.4565 | -35.5 | 439.2200 | 2 | 21.86 | 580 | 1 | 109 | 115 |  |
| R.AYFHTDSK.T | Y | 19.51 | 967.4399 | 625.9 | 485.0300 | 2 | 19.41 | 496 | 4 | 99 | 106 |  |
| K.SDGNG.K | Y | 19.08 | 448.1554 | -2014.2 | 448.2600 | 1 | 65.32 | 2070 | 1 | 118 | 122 |  |
| total 12 peptides |  |  |  |  |  |  |  |  |  |  |  |  |
| --- | --- | --- | --- | --- | --- | --- | --- | --- | --- | --- | --- | --- |

tr|A0A0S1N2H3|A0A0S1N2H3\_9DIPT

back to list

  

| Protein Coverage
| Supporting Peptides
|

Protein Coverage:

Supporting Peptides:

| Peptide | Uniq | -10lgP | Mass | ppm | m/z | z | RT | Scan | #Spec | Start | End | PTM |
| --- | --- | --- | --- | --- | --- | --- | --- | --- | --- | --- | --- | --- |
| X.PLSSG.I | Y | 20.38 | 459.2329 | -2700.6 | 459.0000 | 1 | 5.64 | 24 | 2 | 112 | 116 |  |
| L.SMLIR.A | N | 16.79 | 618.3523 | 1003.3 | 619.9800 | 1 | 54.06 | 1684 | 1 | 15 | 19 |  |
| total 2 peptides |  |  |  |  |  |  |  |  |  |  |  |  |
| --- | --- | --- | --- | --- | --- | --- | --- | --- | --- | --- | --- | --- |

tr|A0A068FD86|A0A068FD86\_9DIPT

back to list

  

| Protein Coverage
| Supporting Peptides
|

Protein Coverage:

Supporting Peptides:

| Peptide | Uniq | -10lgP | Mass | ppm | m/z | z | RT | Scan | #Spec | Start | End | PTM |
| --- | --- | --- | --- | --- | --- | --- | --- | --- | --- | --- | --- | --- |
| L.SMLIR.A | N | 16.79 | 618.3523 | 1003.3 | 619.9800 | 1 | 54.06 | 1684 | 1 | 12 | 16 |  |
| R.SNGIT.F | Y | 16.75 | 490.2387 | 2129.6 | 492.2900 | 1 | 66.67 | 2116 | 1 | 151 | 155 |  |
| total 2 peptides |  |  |  |  |  |  |  |  |  |  |  |  |
| --- | --- | --- | --- | --- | --- | --- | --- | --- | --- | --- | --- | --- |

tr|A0A0H4SK32|A0A0H4SK32\_9DIPT

back to list

  

| Protein Coverage
| Supporting Peptides
|

Protein Coverage:

Supporting Peptides:

| Peptide | Uniq | -10lgP | Mass | ppm | m/z | z | RT | Scan | #Spec | Start | End | PTM |
| --- | --- | --- | --- | --- | --- | --- | --- | --- | --- | --- | --- | --- |
| L.SMLIR.A | N | 16.79 | 618.3523 | 1003.3 | 619.9800 | 1 | 54.06 | 1684 | 1 | 12 | 16 |  |
| R.SNGIT.F | Y | 16.75 | 490.2387 | 2129.6 | 492.2900 | 1 | 66.67 | 2116 | 1 | 151 | 155 |  |
| total 2 peptides |  |  |  |  |  |  |  |  |  |  |  |  |
| --- | --- | --- | --- | --- | --- | --- | --- | --- | --- | --- | --- | --- |

tr|A0A0C4KQM0|A0A0C4KQM0\_9DIPT

back to list

  

| Protein Coverage
| Supporting Peptides
|

Protein Coverage:

Supporting Peptides:

| Peptide | Uniq | -10lgP | Mass | ppm | m/z | z | RT | Scan | #Spec | Start | End | PTM |
| --- | --- | --- | --- | --- | --- | --- | --- | --- | --- | --- | --- | --- |
| L.SMLIR.A | N | 16.79 | 618.3523 | 1003.3 | 619.9800 | 1 | 54.06 | 1684 | 1 | 14 | 18 |  |
| R.SNGIT.F | Y | 16.75 | 490.2387 | 2129.6 | 492.2900 | 1 | 66.67 | 2116 | 1 | 153 | 157 |  |
| total 2 peptides |  |  |  |  |  |  |  |  |  |  |  |  |
| --- | --- | --- | --- | --- | --- | --- | --- | --- | --- | --- | --- | --- |

tr|A0A0C4KJS0|A0A0C4KJS0\_9DIPT

back to list

  

| Protein Coverage
| Supporting Peptides
|

Protein Coverage:

Supporting Peptides:

| Peptide | Uniq | -10lgP | Mass | ppm | m/z | z | RT | Scan | #Spec | Start | End | PTM |
| --- | --- | --- | --- | --- | --- | --- | --- | --- | --- | --- | --- | --- |
| L.SMLIR.A | N | 16.79 | 618.3523 | 1003.3 | 619.9800 | 1 | 54.06 | 1684 | 1 | 14 | 18 |  |
| R.SNGIT.F | Y | 16.75 | 490.2387 | 2129.6 | 492.2900 | 1 | 66.67 | 2116 | 1 | 153 | 157 |  |
| total 2 peptides |  |  |  |  |  |  |  |  |  |  |  |  |
| --- | --- | --- | --- | --- | --- | --- | --- | --- | --- | --- | --- | --- |

tr|A0A0C4KIW4|A0A0C4KIW4\_9DIPT

back to list

  

| Protein Coverage
| Supporting Peptides
|

Protein Coverage:

Supporting Peptides:

| Peptide | Uniq | -10lgP | Mass | ppm | m/z | z | RT | Scan | #Spec | Start | End | PTM |
| --- | --- | --- | --- | --- | --- | --- | --- | --- | --- | --- | --- | --- |
| L.SMLIR.A | N | 16.79 | 618.3523 | 1003.3 | 619.9800 | 1 | 54.06 | 1684 | 1 | 14 | 18 |  |
| R.SNGIT.F | Y | 16.75 | 490.2387 | 2129.6 | 492.2900 | 1 | 66.67 | 2116 | 1 | 153 | 157 |  |
| total 2 peptides |  |  |  |  |  |  |  |  |  |  |  |  |
| --- | --- | --- | --- | --- | --- | --- | --- | --- | --- | --- | --- | --- |

tr|A0A0C4KIY0|A0A0C4KIY0\_9DIPT

back to list

  

| Protein Coverage
| Supporting Peptides
|

Protein Coverage:

Supporting Peptides:

| Peptide | Uniq | -10lgP | Mass | ppm | m/z | z | RT | Scan | #Spec | Start | End | PTM |
| --- | --- | --- | --- | --- | --- | --- | --- | --- | --- | --- | --- | --- |
| L.SMLIR.A | N | 16.79 | 618.3523 | 1003.3 | 619.9800 | 1 | 54.06 | 1684 | 1 | 14 | 18 |  |
| R.SNGIT.F | Y | 16.75 | 490.2387 | 2129.6 | 492.2900 | 1 | 66.67 | 2116 | 1 | 153 | 157 |  |
| total 2 peptides |  |  |  |  |  |  |  |  |  |  |  |  |
| --- | --- | --- | --- | --- | --- | --- | --- | --- | --- | --- | --- | --- |

tr|A0A0C4KU88|A0A0C4KU88\_9DIPT

back to list

  

| Protein Coverage
| Supporting Peptides
|

Protein Coverage:

Supporting Peptides:

| Peptide | Uniq | -10lgP | Mass | ppm | m/z | z | RT | Scan | #Spec | Start | End | PTM |
| --- | --- | --- | --- | --- | --- | --- | --- | --- | --- | --- | --- | --- |
| L.SMLIR.A | N | 16.79 | 618.3523 | 1003.3 | 619.9800 | 1 | 54.06 | 1684 | 1 | 14 | 18 |  |
| R.SNGIT.F | Y | 16.75 | 490.2387 | 2129.6 | 492.2900 | 1 | 66.67 | 2116 | 1 | 153 | 157 |  |
| total 2 peptides |  |  |  |  |  |  |  |  |  |  |  |  |
| --- | --- | --- | --- | --- | --- | --- | --- | --- | --- | --- | --- | --- |

tr|A0A0C4KKB9|A0A0C4KKB9\_9DIPT

back to list

  

| Protein Coverage
| Supporting Peptides
|

Protein Coverage:

Supporting Peptides:

| Peptide | Uniq | -10lgP | Mass | ppm | m/z | z | RT | Scan | #Spec | Start | End | PTM |
| --- | --- | --- | --- | --- | --- | --- | --- | --- | --- | --- | --- | --- |
| L.SMLIR.A | N | 16.79 | 618.3523 | 1003.3 | 619.9800 | 1 | 54.06 | 1684 | 1 | 14 | 18 |  |
| R.SNGIT.F | Y | 16.75 | 490.2387 | 2129.6 | 492.2900 | 1 | 66.67 | 2116 | 1 | 153 | 157 |  |
| total 2 peptides |  |  |  |  |  |  |  |  |  |  |  |  |
| --- | --- | --- | --- | --- | --- | --- | --- | --- | --- | --- | --- | --- |

tr|A0A0C4KQA3|A0A0C4KQA3\_9DIPT

back to list

  

| Protein Coverage
| Supporting Peptides
|

Protein Coverage:

Supporting Peptides:

| Peptide | Uniq | -10lgP | Mass | ppm | m/z | z | RT | Scan | #Spec | Start | End | PTM |
| --- | --- | --- | --- | --- | --- | --- | --- | --- | --- | --- | --- | --- |
| L.SMLIR.A | N | 16.79 | 618.3523 | 1003.3 | 619.9800 | 1 | 54.06 | 1684 | 1 | 15 | 19 |  |
| R.SNGIT.F | Y | 16.75 | 490.2387 | 2129.6 | 492.2900 | 1 | 66.67 | 2116 | 1 | 154 | 158 |  |
| total 2 peptides |  |  |  |  |  |  |  |  |  |  |  |  |
| --- | --- | --- | --- | --- | --- | --- | --- | --- | --- | --- | --- | --- |

tr|A0A0C4KUC8|A0A0C4KUC8\_9DIPT

back to list

  

| Protein Coverage
| Supporting Peptides
|

Protein Coverage:

Supporting Peptides:

| Peptide | Uniq | -10lgP | Mass | ppm | m/z | z | RT | Scan | #Spec | Start | End | PTM |
| --- | --- | --- | --- | --- | --- | --- | --- | --- | --- | --- | --- | --- |
| L.SMLIR.A | N | 16.79 | 618.3523 | 1003.3 | 619.9800 | 1 | 54.06 | 1684 | 1 | 15 | 19 |  |
| R.SNGIT.F | Y | 16.75 | 490.2387 | 2129.6 | 492.2900 | 1 | 66.67 | 2116 | 1 | 154 | 158 |  |
| total 2 peptides |  |  |  |  |  |  |  |  |  |  |  |  |
| --- | --- | --- | --- | --- | --- | --- | --- | --- | --- | --- | --- | --- |

tr|A0A0C4KVL3|A0A0C4KVL3\_9DIPT

back to list

  

| Protein Coverage
| Supporting Peptides
|

Protein Coverage:

Supporting Peptides:

| Peptide | Uniq | -10lgP | Mass | ppm | m/z | z | RT | Scan | #Spec | Start | End | PTM |
| --- | --- | --- | --- | --- | --- | --- | --- | --- | --- | --- | --- | --- |
| L.SMLIR.A | N | 16.79 | 618.3523 | 1003.3 | 619.9800 | 1 | 54.06 | 1684 | 1 | 15 | 19 |  |
| R.SNGIT.F | Y | 16.75 | 490.2387 | 2129.6 | 492.2900 | 1 | 66.67 | 2116 | 1 | 154 | 158 |  |
| total 2 peptides |  |  |  |  |  |  |  |  |  |  |  |  |
| --- | --- | --- | --- | --- | --- | --- | --- | --- | --- | --- | --- | --- |

tr|A0A0C4KVJ8|A0A0C4KVJ8\_9DIPT

back to list

  

| Protein Coverage
| Supporting Peptides
|

Protein Coverage:

Supporting Peptides:

| Peptide | Uniq | -10lgP | Mass | ppm | m/z | z | RT | Scan | #Spec | Start | End | PTM |
| --- | --- | --- | --- | --- | --- | --- | --- | --- | --- | --- | --- | --- |
| L.SMLIR.A | N | 16.79 | 618.3523 | 1003.3 | 619.9800 | 1 | 54.06 | 1684 | 1 | 15 | 19 |  |
| R.SNGIT.F | Y | 16.75 | 490.2387 | 2129.6 | 492.2900 | 1 | 66.67 | 2116 | 1 | 154 | 158 |  |
| total 2 peptides |  |  |  |  |  |  |  |  |  |  |  |  |
| --- | --- | --- | --- | --- | --- | --- | --- | --- | --- | --- | --- | --- |

tr|A0A0C4KIH2|A0A0C4KIH2\_9DIPT

back to list

  

| Protein Coverage
| Supporting Peptides
|

Protein Coverage:

Supporting Peptides:

| Peptide | Uniq | -10lgP | Mass | ppm | m/z | z | RT | Scan | #Spec | Start | End | PTM |
| --- | --- | --- | --- | --- | --- | --- | --- | --- | --- | --- | --- | --- |
| L.SMLIR.A | N | 16.79 | 618.3523 | 1003.3 | 619.9800 | 1 | 54.06 | 1684 | 1 | 15 | 19 |  |
| R.SNGIT.F | Y | 16.75 | 490.2387 | 2129.6 | 492.2900 | 1 | 66.67 | 2116 | 1 | 154 | 158 |  |
| total 2 peptides |  |  |  |  |  |  |  |  |  |  |  |  |
| --- | --- | --- | --- | --- | --- | --- | --- | --- | --- | --- | --- | --- |

tr|A0A0C4KVN9|A0A0C4KVN9\_9DIPT

back to list

  

| Protein Coverage
| Supporting Peptides
|

Protein Coverage:

Supporting Peptides:

| Peptide | Uniq | -10lgP | Mass | ppm | m/z | z | RT | Scan | #Spec | Start | End | PTM |
| --- | --- | --- | --- | --- | --- | --- | --- | --- | --- | --- | --- | --- |
| L.SMLIR.A | N | 16.79 | 618.3523 | 1003.3 | 619.9800 | 1 | 54.06 | 1684 | 1 | 15 | 19 |  |
| R.SNGIT.F | Y | 16.75 | 490.2387 | 2129.6 | 492.2900 | 1 | 66.67 | 2116 | 1 | 154 | 158 |  |
| total 2 peptides |  |  |  |  |  |  |  |  |  |  |  |  |
| --- | --- | --- | --- | --- | --- | --- | --- | --- | --- | --- | --- | --- |

tr|A0A0C4KQR5|A0A0C4KQR5\_SIMVI

back to list

  

| Protein Coverage
| Supporting Peptides
|

Protein Coverage:

Supporting Peptides:

| Peptide | Uniq | -10lgP | Mass | ppm | m/z | z | RT | Scan | #Spec | Start | End | PTM |
| --- | --- | --- | --- | --- | --- | --- | --- | --- | --- | --- | --- | --- |
| L.SMLIR.A | N | 16.79 | 618.3523 | 1003.3 | 619.9800 | 1 | 54.06 | 1684 | 1 | 15 | 19 |  |
| R.SNGIT.F | Y | 16.75 | 490.2387 | 2129.6 | 492.2900 | 1 | 66.67 | 2116 | 1 | 154 | 158 |  |
| total 2 peptides |  |  |  |  |  |  |  |  |  |  |  |  |
| --- | --- | --- | --- | --- | --- | --- | --- | --- | --- | --- | --- | --- |

tr|A0A0P0AU98|A0A0P0AU98\_9DIPT

back to list

  

| Protein Coverage
| Supporting Peptides
|

Protein Coverage:

Supporting Peptides:

| Peptide | Uniq | -10lgP | Mass | ppm | m/z | z | RT | Scan | #Spec | Start | End | PTM |
| --- | --- | --- | --- | --- | --- | --- | --- | --- | --- | --- | --- | --- |
| L.SMLIR.A | N | 16.79 | 618.3523 | 1003.3 | 619.9800 | 1 | 54.06 | 1684 | 1 | 15 | 19 |  |
| R.SNGIT.F | Y | 16.75 | 490.2387 | 2129.6 | 492.2900 | 1 | 66.67 | 2116 | 1 | 154 | 158 |  |
| total 2 peptides |  |  |  |  |  |  |  |  |  |  |  |  |
| --- | --- | --- | --- | --- | --- | --- | --- | --- | --- | --- | --- | --- |

tr|A0A0P0C8G4|A0A0P0C8G4\_9DIPT

back to list

  

| Protein Coverage
| Supporting Peptides
|

Protein Coverage:

Supporting Peptides:

| Peptide | Uniq | -10lgP | Mass | ppm | m/z | z | RT | Scan | #Spec | Start | End | PTM |
| --- | --- | --- | --- | --- | --- | --- | --- | --- | --- | --- | --- | --- |
| L.SMLIR.A | N | 16.79 | 618.3523 | 1003.3 | 619.9800 | 1 | 54.06 | 1684 | 1 | 15 | 19 |  |
| R.SNGIT.F | Y | 16.75 | 490.2387 | 2129.6 | 492.2900 | 1 | 66.67 | 2116 | 1 | 154 | 158 |  |
| total 2 peptides |  |  |  |  |  |  |  |  |  |  |  |  |
| --- | --- | --- | --- | --- | --- | --- | --- | --- | --- | --- | --- | --- |

tr|A0A0P0A5K3|A0A0P0A5K3\_9DIPT

back to list

  

| Protein Coverage
| Supporting Peptides
|

Protein Coverage:

Supporting Peptides:

| Peptide | Uniq | -10lgP | Mass | ppm | m/z | z | RT | Scan | #Spec | Start | End | PTM |
| --- | --- | --- | --- | --- | --- | --- | --- | --- | --- | --- | --- | --- |
| L.SMLIR.A | N | 16.79 | 618.3523 | 1003.3 | 619.9800 | 1 | 54.06 | 1684 | 1 | 15 | 19 |  |
| R.SNGIT.F | Y | 16.75 | 490.2387 | 2129.6 | 492.2900 | 1 | 66.67 | 2116 | 1 | 154 | 158 |  |
| total 2 peptides |  |  |  |  |  |  |  |  |  |  |  |  |
| --- | --- | --- | --- | --- | --- | --- | --- | --- | --- | --- | --- | --- |

tr|A0A0N9G0Y1|A0A0N9G0Y1\_SIMVI

back to list

  

| Protein Coverage
| Supporting Peptides
|

Protein Coverage:

Supporting Peptides:

| Peptide | Uniq | -10lgP | Mass | ppm | m/z | z | RT | Scan | #Spec | Start | End | PTM |
| --- | --- | --- | --- | --- | --- | --- | --- | --- | --- | --- | --- | --- |
| L.SMLIR.A | N | 16.79 | 618.3523 | 1003.3 | 619.9800 | 1 | 54.06 | 1684 | 1 | 15 | 19 |  |
| R.SNGIT.F | Y | 16.75 | 490.2387 | 2129.6 | 492.2900 | 1 | 66.67 | 2116 | 1 | 154 | 158 |  |
| total 2 peptides |  |  |  |  |  |  |  |  |  |  |  |  |
| --- | --- | --- | --- | --- | --- | --- | --- | --- | --- | --- | --- | --- |

tr|A0A0P0C7Y2|A0A0P0C7Y2\_9DIPT

back to list

  

| Protein Coverage
| Supporting Peptides
|

Protein Coverage:

Supporting Peptides:

| Peptide | Uniq | -10lgP | Mass | ppm | m/z | z | RT | Scan | #Spec | Start | End | PTM |
| --- | --- | --- | --- | --- | --- | --- | --- | --- | --- | --- | --- | --- |
| L.SMLIR.A | N | 16.79 | 618.3523 | 1003.3 | 619.9800 | 1 | 54.06 | 1684 | 1 | 15 | 19 |  |
| R.SNGIT.F | Y | 16.75 | 490.2387 | 2129.6 | 492.2900 | 1 | 66.67 | 2116 | 1 | 154 | 158 |  |
| total 2 peptides |  |  |  |  |  |  |  |  |  |  |  |  |
| --- | --- | --- | --- | --- | --- | --- | --- | --- | --- | --- | --- | --- |

tr|A0A0C4KIN5|A0A0C4KIN5\_9DIPT

back to list

  

| Protein Coverage
| Supporting Peptides
|

Protein Coverage:

Supporting Peptides:

| Peptide | Uniq | -10lgP | Mass | ppm | m/z | z | RT | Scan | #Spec | Start | End | PTM |
| --- | --- | --- | --- | --- | --- | --- | --- | --- | --- | --- | --- | --- |
| L.SMLIR.A | N | 16.79 | 618.3523 | 1003.3 | 619.9800 | 1 | 54.06 | 1684 | 1 | 18 | 22 |  |
| R.SNGIT.F | Y | 16.75 | 490.2387 | 2129.6 | 492.2900 | 1 | 66.67 | 2116 | 1 | 157 | 161 |  |
| total 2 peptides |  |  |  |  |  |  |  |  |  |  |  |  |
| --- | --- | --- | --- | --- | --- | --- | --- | --- | --- | --- | --- | --- |

tr|A0A0C4KJ12|A0A0C4KJ12\_SIMVI

back to list

  

| Protein Coverage
| Supporting Peptides
|

Protein Coverage:

Supporting Peptides:

| Peptide | Uniq | -10lgP | Mass | ppm | m/z | z | RT | Scan | #Spec | Start | End | PTM |
| --- | --- | --- | --- | --- | --- | --- | --- | --- | --- | --- | --- | --- |
| L.SMLIR.A | N | 16.79 | 618.3523 | 1003.3 | 619.9800 | 1 | 54.06 | 1684 | 1 | 18 | 22 |  |
| R.SNGIT.F | Y | 16.75 | 490.2387 | 2129.6 | 492.2900 | 1 | 66.67 | 2116 | 1 | 157 | 161 |  |
| total 2 peptides |  |  |  |  |  |  |  |  |  |  |  |  |
| --- | --- | --- | --- | --- | --- | --- | --- | --- | --- | --- | --- | --- |

tr|A0A0C4KJT1|A0A0C4KJT1\_9DIPT

back to list

  

| Protein Coverage
| Supporting Peptides
|

Protein Coverage:

Supporting Peptides:

| Peptide | Uniq | -10lgP | Mass | ppm | m/z | z | RT | Scan | #Spec | Start | End | PTM |
| --- | --- | --- | --- | --- | --- | --- | --- | --- | --- | --- | --- | --- |
| L.SMLIR.A | N | 16.79 | 618.3523 | 1003.3 | 619.9800 | 1 | 54.06 | 1684 | 1 | 18 | 22 |  |
| R.SNGIT.F | Y | 16.75 | 490.2387 | 2129.6 | 492.2900 | 1 | 66.67 | 2116 | 1 | 157 | 161 |  |
| total 2 peptides |  |  |  |  |  |  |  |  |  |  |  |  |
| --- | --- | --- | --- | --- | --- | --- | --- | --- | --- | --- | --- | --- |

tr|A0A0C4KVU9|A0A0C4KVU9\_SIMVI

back to list

  

| Protein Coverage
| Supporting Peptides
|

Protein Coverage:

Supporting Peptides:

| Peptide | Uniq | -10lgP | Mass | ppm | m/z | z | RT | Scan | #Spec | Start | End | PTM |
| --- | --- | --- | --- | --- | --- | --- | --- | --- | --- | --- | --- | --- |
| L.SMLIR.A | N | 16.79 | 618.3523 | 1003.3 | 619.9800 | 1 | 54.06 | 1684 | 1 | 18 | 22 |  |
| R.SNGIT.F | Y | 16.75 | 490.2387 | 2129.6 | 492.2900 | 1 | 66.67 | 2116 | 1 | 157 | 161 |  |
| total 2 peptides |  |  |  |  |  |  |  |  |  |  |  |  |
| --- | --- | --- | --- | --- | --- | --- | --- | --- | --- | --- | --- | --- |

tr|A0A0C4KUR6|A0A0C4KUR6\_SIMVI

back to list

  

| Protein Coverage
| Supporting Peptides
|

Protein Coverage:

Supporting Peptides:

| Peptide | Uniq | -10lgP | Mass | ppm | m/z | z | RT | Scan | #Spec | Start | End | PTM |
| --- | --- | --- | --- | --- | --- | --- | --- | --- | --- | --- | --- | --- |
| L.SMLIR.A | N | 16.79 | 618.3523 | 1003.3 | 619.9800 | 1 | 54.06 | 1684 | 1 | 18 | 22 |  |
| R.SNGIT.F | Y | 16.75 | 490.2387 | 2129.6 | 492.2900 | 1 | 66.67 | 2116 | 1 | 157 | 161 |  |
| total 2 peptides |  |  |  |  |  |  |  |  |  |  |  |  |
| --- | --- | --- | --- | --- | --- | --- | --- | --- | --- | --- | --- | --- |

tr|A0A0C4KIN8|A0A0C4KIN8\_9DIPT

back to list

  

| Protein Coverage
| Supporting Peptides
|

Protein Coverage:

Supporting Peptides:

| Peptide | Uniq | -10lgP | Mass | ppm | m/z | z | RT | Scan | #Spec | Start | End | PTM |
| --- | --- | --- | --- | --- | --- | --- | --- | --- | --- | --- | --- | --- |
| L.SMLIR.A | N | 16.79 | 618.3523 | 1003.3 | 619.9800 | 1 | 54.06 | 1684 | 1 | 18 | 22 |  |
| R.SNGIT.F | Y | 16.75 | 490.2387 | 2129.6 | 492.2900 | 1 | 66.67 | 2116 | 1 | 157 | 161 |  |
| total 2 peptides |  |  |  |  |  |  |  |  |  |  |  |  |
| --- | --- | --- | --- | --- | --- | --- | --- | --- | --- | --- | --- | --- |

tr|A0A0C4KUP5|A0A0C4KUP5\_SIMVI

back to list

  

| Protein Coverage
| Supporting Peptides
|

Protein Coverage:

Supporting Peptides:

| Peptide | Uniq | -10lgP | Mass | ppm | m/z | z | RT | Scan | #Spec | Start | End | PTM |
| --- | --- | --- | --- | --- | --- | --- | --- | --- | --- | --- | --- | --- |
| L.SMLIR.A | N | 16.79 | 618.3523 | 1003.3 | 619.9800 | 1 | 54.06 | 1684 | 1 | 18 | 22 |  |
| R.SNGIT.F | Y | 16.75 | 490.2387 | 2129.6 | 492.2900 | 1 | 66.67 | 2116 | 1 | 157 | 161 |  |
| total 2 peptides |  |  |  |  |  |  |  |  |  |  |  |  |
| --- | --- | --- | --- | --- | --- | --- | --- | --- | --- | --- | --- | --- |

tr|A0A0C4KK16|A0A0C4KK16\_9DIPT

back to list

  

| Protein Coverage
| Supporting Peptides
|

Protein Coverage:

Supporting Peptides:

| Peptide | Uniq | -10lgP | Mass | ppm | m/z | z | RT | Scan | #Spec | Start | End | PTM |
| --- | --- | --- | --- | --- | --- | --- | --- | --- | --- | --- | --- | --- |
| L.SMLIR.A | N | 16.79 | 618.3523 | 1003.3 | 619.9800 | 1 | 54.06 | 1684 | 1 | 18 | 22 |  |
| R.SNGIT.F | Y | 16.75 | 490.2387 | 2129.6 | 492.2900 | 1 | 66.67 | 2116 | 1 | 157 | 161 |  |
| total 2 peptides |  |  |  |  |  |  |  |  |  |  |  |  |
| --- | --- | --- | --- | --- | --- | --- | --- | --- | --- | --- | --- | --- |

tr|A0A0C4KJY8|A0A0C4KJY8\_9DIPT

back to list

  

| Protein Coverage
| Supporting Peptides
|

Protein Coverage:

Supporting Peptides:

| Peptide | Uniq | -10lgP | Mass | ppm | m/z | z | RT | Scan | #Spec | Start | End | PTM |
| --- | --- | --- | --- | --- | --- | --- | --- | --- | --- | --- | --- | --- |
| L.SMLIR.A | N | 16.79 | 618.3523 | 1003.3 | 619.9800 | 1 | 54.06 | 1684 | 1 | 18 | 22 |  |
| R.SNGIT.F | Y | 16.75 | 490.2387 | 2129.6 | 492.2900 | 1 | 66.67 | 2116 | 1 | 157 | 161 |  |
| total 2 peptides |  |  |  |  |  |  |  |  |  |  |  |  |
| --- | --- | --- | --- | --- | --- | --- | --- | --- | --- | --- | --- | --- |

tr|A0A0C4KKB4|A0A0C4KKB4\_9DIPT

back to list

  

| Protein Coverage
| Supporting Peptides
|

Protein Coverage:

Supporting Peptides:

| Peptide | Uniq | -10lgP | Mass | ppm | m/z | z | RT | Scan | #Spec | Start | End | PTM |
| --- | --- | --- | --- | --- | --- | --- | --- | --- | --- | --- | --- | --- |
| L.SMLIR.A | N | 16.79 | 618.3523 | 1003.3 | 619.9800 | 1 | 54.06 | 1684 | 1 | 18 | 22 |  |
| R.SNGIT.F | Y | 16.75 | 490.2387 | 2129.6 | 492.2900 | 1 | 66.67 | 2116 | 1 | 157 | 161 |  |
| total 2 peptides |  |  |  |  |  |  |  |  |  |  |  |  |
| --- | --- | --- | --- | --- | --- | --- | --- | --- | --- | --- | --- | --- |

tr|A0A0C4KQQ2|A0A0C4KQQ2\_SIMVI

back to list

  

| Protein Coverage
| Supporting Peptides
|

Protein Coverage:

Supporting Peptides:

| Peptide | Uniq | -10lgP | Mass | ppm | m/z | z | RT | Scan | #Spec | Start | End | PTM |
| --- | --- | --- | --- | --- | --- | --- | --- | --- | --- | --- | --- | --- |
| L.SMLIR.A | N | 16.79 | 618.3523 | 1003.3 | 619.9800 | 1 | 54.06 | 1684 | 1 | 18 | 22 |  |
| R.SNGIT.F | Y | 16.75 | 490.2387 | 2129.6 | 492.2900 | 1 | 66.67 | 2116 | 1 | 157 | 161 |  |
| total 2 peptides |  |  |  |  |  |  |  |  |  |  |  |  |
| --- | --- | --- | --- | --- | --- | --- | --- | --- | --- | --- | --- | --- |

tr|A0A0C4KKA9|A0A0C4KKA9\_9DIPT

back to list

  

| Protein Coverage
| Supporting Peptides
|

Protein Coverage:

Supporting Peptides:

| Peptide | Uniq | -10lgP | Mass | ppm | m/z | z | RT | Scan | #Spec | Start | End | PTM |
| --- | --- | --- | --- | --- | --- | --- | --- | --- | --- | --- | --- | --- |
| L.SMLIR.A | N | 16.79 | 618.3523 | 1003.3 | 619.9800 | 1 | 54.06 | 1684 | 1 | 18 | 22 |  |
| R.SNGIT.F | Y | 16.75 | 490.2387 | 2129.6 | 492.2900 | 1 | 66.67 | 2116 | 1 | 157 | 161 |  |
| total 2 peptides |  |  |  |  |  |  |  |  |  |  |  |  |
| --- | --- | --- | --- | --- | --- | --- | --- | --- | --- | --- | --- | --- |

tr|A0A0C4KIZ2|A0A0C4KIZ2\_9DIPT

back to list

  

| Protein Coverage
| Supporting Peptides
|

Protein Coverage:

Supporting Peptides:

| Peptide | Uniq | -10lgP | Mass | ppm | m/z | z | RT | Scan | #Spec | Start | End | PTM |
| --- | --- | --- | --- | --- | --- | --- | --- | --- | --- | --- | --- | --- |
| L.SMLIR.A | N | 16.79 | 618.3523 | 1003.3 | 619.9800 | 1 | 54.06 | 1684 | 1 | 18 | 22 |  |
| R.SNGIT.F | Y | 16.75 | 490.2387 | 2129.6 | 492.2900 | 1 | 66.67 | 2116 | 1 | 157 | 161 |  |
| total 2 peptides |  |  |  |  |  |  |  |  |  |  |  |  |
| --- | --- | --- | --- | --- | --- | --- | --- | --- | --- | --- | --- | --- |

tr|A0A0C4KQL8|A0A0C4KQL8\_9DIPT

back to list

  

| Protein Coverage
| Supporting Peptides
|

Protein Coverage:

Supporting Peptides:

| Peptide | Uniq | -10lgP | Mass | ppm | m/z | z | RT | Scan | #Spec | Start | End | PTM |
| --- | --- | --- | --- | --- | --- | --- | --- | --- | --- | --- | --- | --- |
| L.SMLIR.A | N | 16.79 | 618.3523 | 1003.3 | 619.9800 | 1 | 54.06 | 1684 | 1 | 18 | 22 |  |
| R.SNGIT.F | Y | 16.75 | 490.2387 | 2129.6 | 492.2900 | 1 | 66.67 | 2116 | 1 | 157 | 161 |  |
| total 2 peptides |  |  |  |  |  |  |  |  |  |  |  |  |
| --- | --- | --- | --- | --- | --- | --- | --- | --- | --- | --- | --- | --- |

tr|A0A0C4KIW0|A0A0C4KIW0\_9DIPT

back to list

  

| Protein Coverage
| Supporting Peptides
|

Protein Coverage:

Supporting Peptides:

| Peptide | Uniq | -10lgP | Mass | ppm | m/z | z | RT | Scan | #Spec | Start | End | PTM |
| --- | --- | --- | --- | --- | --- | --- | --- | --- | --- | --- | --- | --- |
| L.SMLIR.A | N | 16.79 | 618.3523 | 1003.3 | 619.9800 | 1 | 54.06 | 1684 | 1 | 18 | 22 |  |
| R.SNGIT.F | Y | 16.75 | 490.2387 | 2129.6 | 492.2900 | 1 | 66.67 | 2116 | 1 | 157 | 161 |  |
| total 2 peptides |  |  |  |  |  |  |  |  |  |  |  |  |
| --- | --- | --- | --- | --- | --- | --- | --- | --- | --- | --- | --- | --- |

tr|A0A0C4KVR0|A0A0C4KVR0\_9DIPT

back to list

  

| Protein Coverage
| Supporting Peptides
|

Protein Coverage:

Supporting Peptides:

| Peptide | Uniq | -10lgP | Mass | ppm | m/z | z | RT | Scan | #Spec | Start | End | PTM |
| --- | --- | --- | --- | --- | --- | --- | --- | --- | --- | --- | --- | --- |
| L.SMLIR.A | N | 16.79 | 618.3523 | 1003.3 | 619.9800 | 1 | 54.06 | 1684 | 1 | 18 | 22 |  |
| R.SNGIT.F | Y | 16.75 | 490.2387 | 2129.6 | 492.2900 | 1 | 66.67 | 2116 | 1 | 157 | 161 |  |
| total 2 peptides |  |  |  |  |  |  |  |  |  |  |  |  |
| --- | --- | --- | --- | --- | --- | --- | --- | --- | --- | --- | --- | --- |

tr|A0A0C4KKI2|A0A0C4KKI2\_SIMVI

back to list

  

| Protein Coverage
| Supporting Peptides
|

Protein Coverage:

Supporting Peptides:

| Peptide | Uniq | -10lgP | Mass | ppm | m/z | z | RT | Scan | #Spec | Start | End | PTM |
| --- | --- | --- | --- | --- | --- | --- | --- | --- | --- | --- | --- | --- |
| L.SMLIR.A | N | 16.79 | 618.3523 | 1003.3 | 619.9800 | 1 | 54.06 | 1684 | 1 | 18 | 22 |  |
| R.SNGIT.F | Y | 16.75 | 490.2387 | 2129.6 | 492.2900 | 1 | 66.67 | 2116 | 1 | 157 | 161 |  |
| total 2 peptides |  |  |  |  |  |  |  |  |  |  |  |  |
| --- | --- | --- | --- | --- | --- | --- | --- | --- | --- | --- | --- | --- |

tr|A0A0C4KVK2|A0A0C4KVK2\_9DIPT

back to list

  

| Protein Coverage
| Supporting Peptides
|

Protein Coverage:

Supporting Peptides:

| Peptide | Uniq | -10lgP | Mass | ppm | m/z | z | RT | Scan | #Spec | Start | End | PTM |
| --- | --- | --- | --- | --- | --- | --- | --- | --- | --- | --- | --- | --- |
| L.SMLIR.A | N | 16.79 | 618.3523 | 1003.3 | 619.9800 | 1 | 54.06 | 1684 | 1 | 18 | 22 |  |
| R.SNGIT.F | Y | 16.75 | 490.2387 | 2129.6 | 492.2900 | 1 | 66.67 | 2116 | 1 | 157 | 161 |  |
| total 2 peptides |  |  |  |  |  |  |  |  |  |  |  |  |
| --- | --- | --- | --- | --- | --- | --- | --- | --- | --- | --- | --- | --- |

tr|A0A0C4KJ37|A0A0C4KJ37\_SIMVI

back to list

  

| Protein Coverage
| Supporting Peptides
|

Protein Coverage:

Supporting Peptides:

| Peptide | Uniq | -10lgP | Mass | ppm | m/z | z | RT | Scan | #Spec | Start | End | PTM |
| --- | --- | --- | --- | --- | --- | --- | --- | --- | --- | --- | --- | --- |
| L.SMLIR.A | N | 16.79 | 618.3523 | 1003.3 | 619.9800 | 1 | 54.06 | 1684 | 1 | 18 | 22 |  |
| R.SNGIT.F | Y | 16.75 | 490.2387 | 2129.6 | 492.2900 | 1 | 66.67 | 2116 | 1 | 157 | 161 |  |
| total 2 peptides |  |  |  |  |  |  |  |  |  |  |  |  |
| --- | --- | --- | --- | --- | --- | --- | --- | --- | --- | --- | --- | --- |

tr|A0A0C4KVN2|A0A0C4KVN2\_9DIPT

back to list

  

| Protein Coverage
| Supporting Peptides
|

Protein Coverage:

Supporting Peptides:

| Peptide | Uniq | -10lgP | Mass | ppm | m/z | z | RT | Scan | #Spec | Start | End | PTM |
| --- | --- | --- | --- | --- | --- | --- | --- | --- | --- | --- | --- | --- |
| L.SMLIR.A | N | 16.79 | 618.3523 | 1003.3 | 619.9800 | 1 | 54.06 | 1684 | 1 | 18 | 22 |  |
| R.SNGIT.F | Y | 16.75 | 490.2387 | 2129.6 | 492.2900 | 1 | 66.67 | 2116 | 1 | 157 | 161 |  |
| total 2 peptides |  |  |  |  |  |  |  |  |  |  |  |  |
| --- | --- | --- | --- | --- | --- | --- | --- | --- | --- | --- | --- | --- |

tr|A0A0C4KUJ6|A0A0C4KUJ6\_9DIPT

back to list

  

| Protein Coverage
| Supporting Peptides
|

Protein Coverage:

Supporting Peptides:

| Peptide | Uniq | -10lgP | Mass | ppm | m/z | z | RT | Scan | #Spec | Start | End | PTM |
| --- | --- | --- | --- | --- | --- | --- | --- | --- | --- | --- | --- | --- |
| L.SMLIR.A | N | 16.79 | 618.3523 | 1003.3 | 619.9800 | 1 | 54.06 | 1684 | 1 | 18 | 22 |  |
| R.SNGIT.F | Y | 16.75 | 490.2387 | 2129.6 | 492.2900 | 1 | 66.67 | 2116 | 1 | 157 | 161 |  |
| total 2 peptides |  |  |  |  |  |  |  |  |  |  |  |  |
| --- | --- | --- | --- | --- | --- | --- | --- | --- | --- | --- | --- | --- |

tr|A0A0C4KQJ7|A0A0C4KQJ7\_9DIPT

back to list

  

| Protein Coverage
| Supporting Peptides
|

Protein Coverage:

Supporting Peptides:

| Peptide | Uniq | -10lgP | Mass | ppm | m/z | z | RT | Scan | #Spec | Start | End | PTM |
| --- | --- | --- | --- | --- | --- | --- | --- | --- | --- | --- | --- | --- |
| L.SMLIR.A | N | 16.79 | 618.3523 | 1003.3 | 619.9800 | 1 | 54.06 | 1684 | 1 | 18 | 22 |  |
| R.SNGIT.F | Y | 16.75 | 490.2387 | 2129.6 | 492.2900 | 1 | 66.67 | 2116 | 1 | 157 | 161 |  |
| total 2 peptides |  |  |  |  |  |  |  |  |  |  |  |  |
| --- | --- | --- | --- | --- | --- | --- | --- | --- | --- | --- | --- | --- |

tr|A0A0C4KIX6|A0A0C4KIX6\_9DIPT

back to list

  

| Protein Coverage
| Supporting Peptides
|

Protein Coverage:

Supporting Peptides:

| Peptide | Uniq | -10lgP | Mass | ppm | m/z | z | RT | Scan | #Spec | Start | End | PTM |
| --- | --- | --- | --- | --- | --- | --- | --- | --- | --- | --- | --- | --- |
| L.SMLIR.A | N | 16.79 | 618.3523 | 1003.3 | 619.9800 | 1 | 54.06 | 1684 | 1 | 18 | 22 |  |
| R.SNGIT.F | Y | 16.75 | 490.2387 | 2129.6 | 492.2900 | 1 | 66.67 | 2116 | 1 | 157 | 161 |  |
| total 2 peptides |  |  |  |  |  |  |  |  |  |  |  |  |
| --- | --- | --- | --- | --- | --- | --- | --- | --- | --- | --- | --- | --- |

tr|A0A0C4KK32|A0A0C4KK32\_9DIPT

back to list

  

| Protein Coverage
| Supporting Peptides
|

Protein Coverage:

Supporting Peptides:

| Peptide | Uniq | -10lgP | Mass | ppm | m/z | z | RT | Scan | #Spec | Start | End | PTM |
| --- | --- | --- | --- | --- | --- | --- | --- | --- | --- | --- | --- | --- |
| L.SMLIR.A | N | 16.79 | 618.3523 | 1003.3 | 619.9800 | 1 | 54.06 | 1684 | 1 | 18 | 22 |  |
| R.SNGIT.F | Y | 16.75 | 490.2387 | 2129.6 | 492.2900 | 1 | 66.67 | 2116 | 1 | 157 | 161 |  |
| total 2 peptides |  |  |  |  |  |  |  |  |  |  |  |  |
| --- | --- | --- | --- | --- | --- | --- | --- | --- | --- | --- | --- | --- |

tr|A0A0C4KV04|A0A0C4KV04\_SIMVI

back to list

  

| Protein Coverage
| Supporting Peptides
|

Protein Coverage:

Supporting Peptides:

| Peptide | Uniq | -10lgP | Mass | ppm | m/z | z | RT | Scan | #Spec | Start | End | PTM |
| --- | --- | --- | --- | --- | --- | --- | --- | --- | --- | --- | --- | --- |
| L.SMLIR.A | N | 16.79 | 618.3523 | 1003.3 | 619.9800 | 1 | 54.06 | 1684 | 1 | 18 | 22 |  |
| R.SNGIT.F | Y | 16.75 | 490.2387 | 2129.6 | 492.2900 | 1 | 66.67 | 2116 | 1 | 157 | 161 |  |
| total 2 peptides |  |  |  |  |  |  |  |  |  |  |  |  |
| --- | --- | --- | --- | --- | --- | --- | --- | --- | --- | --- | --- | --- |

tr|A0A0C4KK35|A0A0C4KK35\_9DIPT

back to list

  

| Protein Coverage
| Supporting Peptides
|

Protein Coverage:

Supporting Peptides:

| Peptide | Uniq | -10lgP | Mass | ppm | m/z | z | RT | Scan | #Spec | Start | End | PTM |
| --- | --- | --- | --- | --- | --- | --- | --- | --- | --- | --- | --- | --- |
| L.SMLIR.A | N | 16.79 | 618.3523 | 1003.3 | 619.9800 | 1 | 54.06 | 1684 | 1 | 18 | 22 |  |
| R.SNGIT.F | Y | 16.75 | 490.2387 | 2129.6 | 492.2900 | 1 | 66.67 | 2116 | 1 | 157 | 161 |  |
| total 2 peptides |  |  |  |  |  |  |  |  |  |  |  |  |
| --- | --- | --- | --- | --- | --- | --- | --- | --- | --- | --- | --- | --- |

tr|A0A0C4KQI0|A0A0C4KQI0\_9DIPT

back to list

  

| Protein Coverage
| Supporting Peptides
|

Protein Coverage:

Supporting Peptides:

| Peptide | Uniq | -10lgP | Mass | ppm | m/z | z | RT | Scan | #Spec | Start | End | PTM |
| --- | --- | --- | --- | --- | --- | --- | --- | --- | --- | --- | --- | --- |
| L.SMLIR.A | N | 16.79 | 618.3523 | 1003.3 | 619.9800 | 1 | 54.06 | 1684 | 1 | 18 | 22 |  |
| R.SNGIT.F | Y | 16.75 | 490.2387 | 2129.6 | 492.2900 | 1 | 66.67 | 2116 | 1 | 157 | 161 |  |
| total 2 peptides |  |  |  |  |  |  |  |  |  |  |  |  |
| --- | --- | --- | --- | --- | --- | --- | --- | --- | --- | --- | --- | --- |

tr|A0A0C4KVF6|A0A0C4KVF6\_9DIPT

back to list

  

| Protein Coverage
| Supporting Peptides
|

Protein Coverage:

Supporting Peptides:

| Peptide | Uniq | -10lgP | Mass | ppm | m/z | z | RT | Scan | #Spec | Start | End | PTM |
| --- | --- | --- | --- | --- | --- | --- | --- | --- | --- | --- | --- | --- |
| L.SMLIR.A | N | 16.79 | 618.3523 | 1003.3 | 619.9800 | 1 | 54.06 | 1684 | 1 | 18 | 22 |  |
| R.SNGIT.F | Y | 16.75 | 490.2387 | 2129.6 | 492.2900 | 1 | 66.67 | 2116 | 1 | 157 | 161 |  |
| total 2 peptides |  |  |  |  |  |  |  |  |  |  |  |  |
| --- | --- | --- | --- | --- | --- | --- | --- | --- | --- | --- | --- | --- |

tr|A0A0C4KQT6|A0A0C4KQT6\_SIMVI

back to list

  

| Protein Coverage
| Supporting Peptides
|

Protein Coverage:

Supporting Peptides:

| Peptide | Uniq | -10lgP | Mass | ppm | m/z | z | RT | Scan | #Spec | Start | End | PTM |
| --- | --- | --- | --- | --- | --- | --- | --- | --- | --- | --- | --- | --- |
| L.SMLIR.A | N | 16.79 | 618.3523 | 1003.3 | 619.9800 | 1 | 54.06 | 1684 | 1 | 18 | 22 |  |
| R.SNGIT.F | Y | 16.75 | 490.2387 | 2129.6 | 492.2900 | 1 | 66.67 | 2116 | 1 | 157 | 161 |  |
| total 2 peptides |  |  |  |  |  |  |  |  |  |  |  |  |
| --- | --- | --- | --- | --- | --- | --- | --- | --- | --- | --- | --- | --- |

tr|A0A0C4KUK9|A0A0C4KUK9\_9DIPT

back to list

  

| Protein Coverage
| Supporting Peptides
|

Protein Coverage:

Supporting Peptides:

| Peptide | Uniq | -10lgP | Mass | ppm | m/z | z | RT | Scan | #Spec | Start | End | PTM |
| --- | --- | --- | --- | --- | --- | --- | --- | --- | --- | --- | --- | --- |
| L.SMLIR.A | N | 16.79 | 618.3523 | 1003.3 | 619.9800 | 1 | 54.06 | 1684 | 1 | 18 | 22 |  |
| R.SNGIT.F | Y | 16.75 | 490.2387 | 2129.6 | 492.2900 | 1 | 66.67 | 2116 | 1 | 157 | 161 |  |
| total 2 peptides |  |  |  |  |  |  |  |  |  |  |  |  |
| --- | --- | --- | --- | --- | --- | --- | --- | --- | --- | --- | --- | --- |

tr|A0A0C4KQF5|A0A0C4KQF5\_9DIPT

back to list

  

| Protein Coverage
| Supporting Peptides
|

Protein Coverage:

Supporting Peptides:

| Peptide | Uniq | -10lgP | Mass | ppm | m/z | z | RT | Scan | #Spec | Start | End | PTM |
| --- | --- | --- | --- | --- | --- | --- | --- | --- | --- | --- | --- | --- |
| L.SMLIR.A | N | 16.79 | 618.3523 | 1003.3 | 619.9800 | 1 | 54.06 | 1684 | 1 | 18 | 22 |  |
| R.SNGIT.F | Y | 16.75 | 490.2387 | 2129.6 | 492.2900 | 1 | 66.67 | 2116 | 1 | 157 | 161 |  |
| total 2 peptides |  |  |  |  |  |  |  |  |  |  |  |  |
| --- | --- | --- | --- | --- | --- | --- | --- | --- | --- | --- | --- | --- |

tr|A0A0C4KQN9|A0A0C4KQN9\_9DIPT

back to list

  

| Protein Coverage
| Supporting Peptides
|

Protein Coverage:

Supporting Peptides:

| Peptide | Uniq | -10lgP | Mass | ppm | m/z | z | RT | Scan | #Spec | Start | End | PTM |
| --- | --- | --- | --- | --- | --- | --- | --- | --- | --- | --- | --- | --- |
| L.SMLIR.A | N | 16.79 | 618.3523 | 1003.3 | 619.9800 | 1 | 54.06 | 1684 | 1 | 18 | 22 |  |
| R.SNGIT.F | Y | 16.75 | 490.2387 | 2129.6 | 492.2900 | 1 | 66.67 | 2116 | 1 | 157 | 161 |  |
| total 2 peptides |  |  |  |  |  |  |  |  |  |  |  |  |
| --- | --- | --- | --- | --- | --- | --- | --- | --- | --- | --- | --- | --- |

tr|A0A0C4KQQ5|A0A0C4KQQ5\_SIMVI

back to list

  

| Protein Coverage
| Supporting Peptides
|

Protein Coverage:

Supporting Peptides:

| Peptide | Uniq | -10lgP | Mass | ppm | m/z | z | RT | Scan | #Spec | Start | End | PTM |
| --- | --- | --- | --- | --- | --- | --- | --- | --- | --- | --- | --- | --- |
| L.SMLIR.A | N | 16.79 | 618.3523 | 1003.3 | 619.9800 | 1 | 54.06 | 1684 | 1 | 18 | 22 |  |
| R.SNGIT.F | Y | 16.75 | 490.2387 | 2129.6 | 492.2900 | 1 | 66.67 | 2116 | 1 | 157 | 161 |  |
| total 2 peptides |  |  |  |  |  |  |  |  |  |  |  |  |
| --- | --- | --- | --- | --- | --- | --- | --- | --- | --- | --- | --- | --- |

tr|A0A0C4KQL5|A0A0C4KQL5\_9DIPT

back to list

  

| Protein Coverage
| Supporting Peptides
|

Protein Coverage:

Supporting Peptides:

| Peptide | Uniq | -10lgP | Mass | ppm | m/z | z | RT | Scan | #Spec | Start | End | PTM |
| --- | --- | --- | --- | --- | --- | --- | --- | --- | --- | --- | --- | --- |
| L.SMLIR.A | N | 16.79 | 618.3523 | 1003.3 | 619.9800 | 1 | 54.06 | 1684 | 1 | 18 | 22 |  |
| R.SNGIT.F | Y | 16.75 | 490.2387 | 2129.6 | 492.2900 | 1 | 66.67 | 2116 | 1 | 157 | 161 |  |
| total 2 peptides |  |  |  |  |  |  |  |  |  |  |  |  |
| --- | --- | --- | --- | --- | --- | --- | --- | --- | --- | --- | --- | --- |

tr|A0A0C4KQF3|A0A0C4KQF3\_9DIPT

back to list

  

| Protein Coverage
| Supporting Peptides
|

Protein Coverage:

Supporting Peptides:

| Peptide | Uniq | -10lgP | Mass | ppm | m/z | z | RT | Scan | #Spec | Start | End | PTM |
| --- | --- | --- | --- | --- | --- | --- | --- | --- | --- | --- | --- | --- |
| L.SMLIR.A | N | 16.79 | 618.3523 | 1003.3 | 619.9800 | 1 | 54.06 | 1684 | 1 | 18 | 22 |  |
| R.SNGIT.F | Y | 16.75 | 490.2387 | 2129.6 | 492.2900 | 1 | 66.67 | 2116 | 1 | 157 | 161 |  |
| total 2 peptides |  |  |  |  |  |  |  |  |  |  |  |  |
| --- | --- | --- | --- | --- | --- | --- | --- | --- | --- | --- | --- | --- |

tr|A0A0C4KJ74|A0A0C4KJ74\_SIMVI

back to list

  

| Protein Coverage
| Supporting Peptides
|

Protein Coverage:

Supporting Peptides:

| Peptide | Uniq | -10lgP | Mass | ppm | m/z | z | RT | Scan | #Spec | Start | End | PTM |
| --- | --- | --- | --- | --- | --- | --- | --- | --- | --- | --- | --- | --- |
| L.SMLIR.A | N | 16.79 | 618.3523 | 1003.3 | 619.9800 | 1 | 54.06 | 1684 | 1 | 18 | 22 |  |
| R.SNGIT.F | Y | 16.75 | 490.2387 | 2129.6 | 492.2900 | 1 | 66.67 | 2116 | 1 | 157 | 161 |  |
| total 2 peptides |  |  |  |  |  |  |  |  |  |  |  |  |
| --- | --- | --- | --- | --- | --- | --- | --- | --- | --- | --- | --- | --- |

tr|A0A0C4KUA7|A0A0C4KUA7\_9DIPT

back to list

  

| Protein Coverage
| Supporting Peptides
|

Protein Coverage:

Supporting Peptides:

| Peptide | Uniq | -10lgP | Mass | ppm | m/z | z | RT | Scan | #Spec | Start | End | PTM |
| --- | --- | --- | --- | --- | --- | --- | --- | --- | --- | --- | --- | --- |
| L.SMLIR.A | N | 16.79 | 618.3523 | 1003.3 | 619.9800 | 1 | 54.06 | 1684 | 1 | 18 | 22 |  |
| R.SNGIT.F | Y | 16.75 | 490.2387 | 2129.6 | 492.2900 | 1 | 66.67 | 2116 | 1 | 157 | 161 |  |
| total 2 peptides |  |  |  |  |  |  |  |  |  |  |  |  |
| --- | --- | --- | --- | --- | --- | --- | --- | --- | --- | --- | --- | --- |

tr|A0A0C4KVQ7|A0A0C4KVQ7\_9DIPT

back to list

  

| Protein Coverage
| Supporting Peptides
|

Protein Coverage:

Supporting Peptides:

| Peptide | Uniq | -10lgP | Mass | ppm | m/z | z | RT | Scan | #Spec | Start | End | PTM |
| --- | --- | --- | --- | --- | --- | --- | --- | --- | --- | --- | --- | --- |
| L.SMLIR.A | N | 16.79 | 618.3523 | 1003.3 | 619.9800 | 1 | 54.06 | 1684 | 1 | 18 | 22 |  |
| R.SNGIT.F | Y | 16.75 | 490.2387 | 2129.6 | 492.2900 | 1 | 66.67 | 2116 | 1 | 157 | 161 |  |
| total 2 peptides |  |  |  |  |  |  |  |  |  |  |  |  |
| --- | --- | --- | --- | --- | --- | --- | --- | --- | --- | --- | --- | --- |

tr|A0A0C4KU78|A0A0C4KU78\_9DIPT

back to list

  

| Protein Coverage
| Supporting Peptides
|

Protein Coverage:

Supporting Peptides:

| Peptide | Uniq | -10lgP | Mass | ppm | m/z | z | RT | Scan | #Spec | Start | End | PTM |
| --- | --- | --- | --- | --- | --- | --- | --- | --- | --- | --- | --- | --- |
| L.SMLIR.A | N | 16.79 | 618.3523 | 1003.3 | 619.9800 | 1 | 54.06 | 1684 | 1 | 18 | 22 |  |
| R.SNGIT.F | Y | 16.75 | 490.2387 | 2129.6 | 492.2900 | 1 | 66.67 | 2116 | 1 | 157 | 161 |  |
| total 2 peptides |  |  |  |  |  |  |  |  |  |  |  |  |
| --- | --- | --- | --- | --- | --- | --- | --- | --- | --- | --- | --- | --- |

tr|A0A0C4KVT0|A0A0C4KVT0\_9DIPT

back to list

  

| Protein Coverage
| Supporting Peptides
|

Protein Coverage:

Supporting Peptides:

| Peptide | Uniq | -10lgP | Mass | ppm | m/z | z | RT | Scan | #Spec | Start | End | PTM |
| --- | --- | --- | --- | --- | --- | --- | --- | --- | --- | --- | --- | --- |
| L.SMLIR.A | N | 16.79 | 618.3523 | 1003.3 | 619.9800 | 1 | 54.06 | 1684 | 1 | 18 | 22 |  |
| R.SNGIT.F | Y | 16.75 | 490.2387 | 2129.6 | 492.2900 | 1 | 66.67 | 2116 | 1 | 157 | 161 |  |
| total 2 peptides |  |  |  |  |  |  |  |  |  |  |  |  |
| --- | --- | --- | --- | --- | --- | --- | --- | --- | --- | --- | --- | --- |

tr|A0A0C4KQG5|A0A0C4KQG5\_9DIPT

back to list

  

| Protein Coverage
| Supporting Peptides
|

Protein Coverage:

Supporting Peptides:

| Peptide | Uniq | -10lgP | Mass | ppm | m/z | z | RT | Scan | #Spec | Start | End | PTM |
| --- | --- | --- | --- | --- | --- | --- | --- | --- | --- | --- | --- | --- |
| L.SMLIR.A | N | 16.79 | 618.3523 | 1003.3 | 619.9800 | 1 | 54.06 | 1684 | 1 | 18 | 22 |  |
| R.SNGIT.F | Y | 16.75 | 490.2387 | 2129.6 | 492.2900 | 1 | 66.67 | 2116 | 1 | 157 | 161 |  |
| total 2 peptides |  |  |  |  |  |  |  |  |  |  |  |  |
| --- | --- | --- | --- | --- | --- | --- | --- | --- | --- | --- | --- | --- |

tr|A0A0C4KVY4|A0A0C4KVY4\_SIMVI

back to list

  

| Protein Coverage
| Supporting Peptides
|

Protein Coverage:

Supporting Peptides:

| Peptide | Uniq | -10lgP | Mass | ppm | m/z | z | RT | Scan | #Spec | Start | End | PTM |
| --- | --- | --- | --- | --- | --- | --- | --- | --- | --- | --- | --- | --- |
| L.SMLIR.A | N | 16.79 | 618.3523 | 1003.3 | 619.9800 | 1 | 54.06 | 1684 | 1 | 18 | 22 |  |
| R.SNGIT.F | Y | 16.75 | 490.2387 | 2129.6 | 492.2900 | 1 | 66.67 | 2116 | 1 | 157 | 161 |  |
| total 2 peptides |  |  |  |  |  |  |  |  |  |  |  |  |
| --- | --- | --- | --- | --- | --- | --- | --- | --- | --- | --- | --- | --- |

tr|A0A0C4KVX8|A0A0C4KVX8\_SIMVI

back to list

  

| Protein Coverage
| Supporting Peptides
|

Protein Coverage:

Supporting Peptides:

| Peptide | Uniq | -10lgP | Mass | ppm | m/z | z | RT | Scan | #Spec | Start | End | PTM |
| --- | --- | --- | --- | --- | --- | --- | --- | --- | --- | --- | --- | --- |
| L.SMLIR.A | N | 16.79 | 618.3523 | 1003.3 | 619.9800 | 1 | 54.06 | 1684 | 1 | 18 | 22 |  |
| R.SNGIT.F | Y | 16.75 | 490.2387 | 2129.6 | 492.2900 | 1 | 66.67 | 2116 | 1 | 157 | 161 |  |
| total 2 peptides |  |  |  |  |  |  |  |  |  |  |  |  |
| --- | --- | --- | --- | --- | --- | --- | --- | --- | --- | --- | --- | --- |

tr|A0A0C4KUN7|A0A0C4KUN7\_9DIPT

back to list

  

| Protein Coverage
| Supporting Peptides
|

Protein Coverage:

Supporting Peptides:

| Peptide | Uniq | -10lgP | Mass | ppm | m/z | z | RT | Scan | #Spec | Start | End | PTM |
| --- | --- | --- | --- | --- | --- | --- | --- | --- | --- | --- | --- | --- |
| L.SMLIR.A | N | 16.79 | 618.3523 | 1003.3 | 619.9800 | 1 | 54.06 | 1684 | 1 | 18 | 22 |  |
| R.SNGIT.F | Y | 16.75 | 490.2387 | 2129.6 | 492.2900 | 1 | 66.67 | 2116 | 1 | 157 | 161 |  |
| total 2 peptides |  |  |  |  |  |  |  |  |  |  |  |  |
| --- | --- | --- | --- | --- | --- | --- | --- | --- | --- | --- | --- | --- |

tr|A0A0C4KU72|A0A0C4KU72\_9DIPT

back to list

  

| Protein Coverage
| Supporting Peptides
|

Protein Coverage:

Supporting Peptides:

| Peptide | Uniq | -10lgP | Mass | ppm | m/z | z | RT | Scan | #Spec | Start | End | PTM |
| --- | --- | --- | --- | --- | --- | --- | --- | --- | --- | --- | --- | --- |
| L.SMLIR.A | N | 16.79 | 618.3523 | 1003.3 | 619.9800 | 1 | 54.06 | 1684 | 1 | 18 | 22 |  |
| R.SNGIT.F | Y | 16.75 | 490.2387 | 2129.6 | 492.2900 | 1 | 66.67 | 2116 | 1 | 157 | 161 |  |
| total 2 peptides |  |  |  |  |  |  |  |  |  |  |  |  |
| --- | --- | --- | --- | --- | --- | --- | --- | --- | --- | --- | --- | --- |

tr|A0A0C4KUE0|A0A0C4KUE0\_9DIPT

back to list

  

| Protein Coverage
| Supporting Peptides
|

Protein Coverage:

Supporting Peptides:

| Peptide | Uniq | -10lgP | Mass | ppm | m/z | z | RT | Scan | #Spec | Start | End | PTM |
| --- | --- | --- | --- | --- | --- | --- | --- | --- | --- | --- | --- | --- |
| L.SMLIR.A | N | 16.79 | 618.3523 | 1003.3 | 619.9800 | 1 | 54.06 | 1684 | 1 | 18 | 22 |  |
| R.SNGIT.F | Y | 16.75 | 490.2387 | 2129.6 | 492.2900 | 1 | 66.67 | 2116 | 1 | 157 | 161 |  |
| total 2 peptides |  |  |  |  |  |  |  |  |  |  |  |  |
| --- | --- | --- | --- | --- | --- | --- | --- | --- | --- | --- | --- | --- |

tr|A0A0C4KUF8|A0A0C4KUF8\_9DIPT

back to list

  

| Protein Coverage
| Supporting Peptides
|

Protein Coverage:

Supporting Peptides:

| Peptide | Uniq | -10lgP | Mass | ppm | m/z | z | RT | Scan | #Spec | Start | End | PTM |
| --- | --- | --- | --- | --- | --- | --- | --- | --- | --- | --- | --- | --- |
| L.SMLIR.A | N | 16.79 | 618.3523 | 1003.3 | 619.9800 | 1 | 54.06 | 1684 | 1 | 18 | 22 |  |
| R.SNGIT.F | Y | 16.75 | 490.2387 | 2129.6 | 492.2900 | 1 | 66.67 | 2116 | 1 | 157 | 161 |  |
| total 2 peptides |  |  |  |  |  |  |  |  |  |  |  |  |
| --- | --- | --- | --- | --- | --- | --- | --- | --- | --- | --- | --- | --- |

tr|A0A0C4KQL2|A0A0C4KQL2\_9DIPT

back to list

  

| Protein Coverage
| Supporting Peptides
|

Protein Coverage:

Supporting Peptides:

| Peptide | Uniq | -10lgP | Mass | ppm | m/z | z | RT | Scan | #Spec | Start | End | PTM |
| --- | --- | --- | --- | --- | --- | --- | --- | --- | --- | --- | --- | --- |
| L.SMLIR.A | N | 16.79 | 618.3523 | 1003.3 | 619.9800 | 1 | 54.06 | 1684 | 1 | 18 | 22 |  |
| R.SNGIT.F | Y | 16.75 | 490.2387 | 2129.6 | 492.2900 | 1 | 66.67 | 2116 | 1 | 157 | 161 |  |
| total 2 peptides |  |  |  |  |  |  |  |  |  |  |  |  |
| --- | --- | --- | --- | --- | --- | --- | --- | --- | --- | --- | --- | --- |

tr|A0A0C4KK84|A0A0C4KK84\_9DIPT

back to list

  

| Protein Coverage
| Supporting Peptides
|

Protein Coverage:

Supporting Peptides:

| Peptide | Uniq | -10lgP | Mass | ppm | m/z | z | RT | Scan | #Spec | Start | End | PTM |
| --- | --- | --- | --- | --- | --- | --- | --- | --- | --- | --- | --- | --- |
| L.SMLIR.A | N | 16.79 | 618.3523 | 1003.3 | 619.9800 | 1 | 54.06 | 1684 | 1 | 18 | 22 |  |
| R.SNGIT.F | Y | 16.75 | 490.2387 | 2129.6 | 492.2900 | 1 | 66.67 | 2116 | 1 | 157 | 161 |  |
| total 2 peptides |  |  |  |  |  |  |  |  |  |  |  |  |
| --- | --- | --- | --- | --- | --- | --- | --- | --- | --- | --- | --- | --- |

tr|A0A0C4KK98|A0A0C4KK98\_9DIPT

back to list

  

| Protein Coverage
| Supporting Peptides
|

Protein Coverage:

Supporting Peptides:

| Peptide | Uniq | -10lgP | Mass | ppm | m/z | z | RT | Scan | #Spec | Start | End | PTM |
| --- | --- | --- | --- | --- | --- | --- | --- | --- | --- | --- | --- | --- |
| L.SMLIR.A | N | 16.79 | 618.3523 | 1003.3 | 619.9800 | 1 | 54.06 | 1684 | 1 | 18 | 22 |  |
| R.SNGIT.F | Y | 16.75 | 490.2387 | 2129.6 | 492.2900 | 1 | 66.67 | 2116 | 1 | 157 | 161 |  |
| total 2 peptides |  |  |  |  |  |  |  |  |  |  |  |  |
| --- | --- | --- | --- | --- | --- | --- | --- | --- | --- | --- | --- | --- |

tr|A0A0C4KUJ0|A0A0C4KUJ0\_9DIPT

back to list

  

| Protein Coverage
| Supporting Peptides
|

Protein Coverage:

Supporting Peptides:

| Peptide | Uniq | -10lgP | Mass | ppm | m/z | z | RT | Scan | #Spec | Start | End | PTM |
| --- | --- | --- | --- | --- | --- | --- | --- | --- | --- | --- | --- | --- |
| L.SMLIR.A | N | 16.79 | 618.3523 | 1003.3 | 619.9800 | 1 | 54.06 | 1684 | 1 | 18 | 22 |  |
| R.SNGIT.F | Y | 16.75 | 490.2387 | 2129.6 | 492.2900 | 1 | 66.67 | 2116 | 1 | 157 | 161 |  |
| total 2 peptides |  |  |  |  |  |  |  |  |  |  |  |  |
| --- | --- | --- | --- | --- | --- | --- | --- | --- | --- | --- | --- | --- |

tr|A0A0C4KQV6|A0A0C4KQV6\_SIMVI

back to list

  

| Protein Coverage
| Supporting Peptides
|

Protein Coverage:

Supporting Peptides:

| Peptide | Uniq | -10lgP | Mass | ppm | m/z | z | RT | Scan | #Spec | Start | End | PTM |
| --- | --- | --- | --- | --- | --- | --- | --- | --- | --- | --- | --- | --- |
| L.SMLIR.A | N | 16.79 | 618.3523 | 1003.3 | 619.9800 | 1 | 54.06 | 1684 | 1 | 18 | 22 |  |
| R.SNGIT.F | Y | 16.75 | 490.2387 | 2129.6 | 492.2900 | 1 | 66.67 | 2116 | 1 | 157 | 161 |  |
| total 2 peptides |  |  |  |  |  |  |  |  |  |  |  |  |
| --- | --- | --- | --- | --- | --- | --- | --- | --- | --- | --- | --- | --- |

tr|A0A0C4KVQ4|A0A0C4KVQ4\_9DIPT

back to list

  

| Protein Coverage
| Supporting Peptides
|

Protein Coverage:

Supporting Peptides:

| Peptide | Uniq | -10lgP | Mass | ppm | m/z | z | RT | Scan | #Spec | Start | End | PTM |
| --- | --- | --- | --- | --- | --- | --- | --- | --- | --- | --- | --- | --- |
| L.SMLIR.A | N | 16.79 | 618.3523 | 1003.3 | 619.9800 | 1 | 54.06 | 1684 | 1 | 18 | 22 |  |
| R.SNGIT.F | Y | 16.75 | 490.2387 | 2129.6 | 492.2900 | 1 | 66.67 | 2116 | 1 | 157 | 161 |  |
| total 2 peptides |  |  |  |  |  |  |  |  |  |  |  |  |
| --- | --- | --- | --- | --- | --- | --- | --- | --- | --- | --- | --- | --- |

tr|A0A0C4KVT4|A0A0C4KVT4\_SIMVI

back to list

  

| Protein Coverage
| Supporting Peptides
|

Protein Coverage:

Supporting Peptides:

| Peptide | Uniq | -10lgP | Mass | ppm | m/z | z | RT | Scan | #Spec | Start | End | PTM |
| --- | --- | --- | --- | --- | --- | --- | --- | --- | --- | --- | --- | --- |
| L.SMLIR.A | N | 16.79 | 618.3523 | 1003.3 | 619.9800 | 1 | 54.06 | 1684 | 1 | 18 | 22 |  |
| R.SNGIT.F | Y | 16.75 | 490.2387 | 2129.6 | 492.2900 | 1 | 66.67 | 2116 | 1 | 157 | 161 |  |
| total 2 peptides |  |  |  |  |  |  |  |  |  |  |  |  |
| --- | --- | --- | --- | --- | --- | --- | --- | --- | --- | --- | --- | --- |

tr|A0A0C4KQT9|A0A0C4KQT9\_SIMVI

back to list

  

| Protein Coverage
| Supporting Peptides
|

Protein Coverage:

Supporting Peptides:

| Peptide | Uniq | -10lgP | Mass | ppm | m/z | z | RT | Scan | #Spec | Start | End | PTM |
| --- | --- | --- | --- | --- | --- | --- | --- | --- | --- | --- | --- | --- |
| L.SMLIR.A | N | 16.79 | 618.3523 | 1003.3 | 619.9800 | 1 | 54.06 | 1684 | 1 | 18 | 22 |  |
| R.SNGIT.F | Y | 16.75 | 490.2387 | 2129.6 | 492.2900 | 1 | 66.67 | 2116 | 1 | 157 | 161 |  |
| total 2 peptides |  |  |  |  |  |  |  |  |  |  |  |  |
| --- | --- | --- | --- | --- | --- | --- | --- | --- | --- | --- | --- | --- |

tr|A0A0C4KKF6|A0A0C4KKF6\_9DIPT

back to list

  

| Protein Coverage
| Supporting Peptides
|

Protein Coverage:

Supporting Peptides:

| Peptide | Uniq | -10lgP | Mass | ppm | m/z | z | RT | Scan | #Spec | Start | End | PTM |
| --- | --- | --- | --- | --- | --- | --- | --- | --- | --- | --- | --- | --- |
| L.SMLIR.A | N | 16.79 | 618.3523 | 1003.3 | 619.9800 | 1 | 54.06 | 1684 | 1 | 18 | 22 |  |
| R.SNGIT.F | Y | 16.75 | 490.2387 | 2129.6 | 492.2900 | 1 | 66.67 | 2116 | 1 | 157 | 161 |  |
| total 2 peptides |  |  |  |  |  |  |  |  |  |  |  |  |
| --- | --- | --- | --- | --- | --- | --- | --- | --- | --- | --- | --- | --- |

tr|A0A0C4KK79|A0A0C4KK79\_9DIPT

back to list

  

| Protein Coverage
| Supporting Peptides
|

Protein Coverage:

Supporting Peptides:

| Peptide | Uniq | -10lgP | Mass | ppm | m/z | z | RT | Scan | #Spec | Start | End | PTM |
| --- | --- | --- | --- | --- | --- | --- | --- | --- | --- | --- | --- | --- |
| L.SMLIR.A | N | 16.79 | 618.3523 | 1003.3 | 619.9800 | 1 | 54.06 | 1684 | 1 | 18 | 22 |  |
| R.SNGIT.F | Y | 16.75 | 490.2387 | 2129.6 | 492.2900 | 1 | 66.67 | 2116 | 1 | 157 | 161 |  |
| total 2 peptides |  |  |  |  |  |  |  |  |  |  |  |  |
| --- | --- | --- | --- | --- | --- | --- | --- | --- | --- | --- | --- | --- |

tr|A0A0C4KJ00|A0A0C4KJ00\_SIMVI

back to list

  

| Protein Coverage
| Supporting Peptides
|

Protein Coverage:

Supporting Peptides:

| Peptide | Uniq | -10lgP | Mass | ppm | m/z | z | RT | Scan | #Spec | Start | End | PTM |
| --- | --- | --- | --- | --- | --- | --- | --- | --- | --- | --- | --- | --- |
| L.SMLIR.A | N | 16.79 | 618.3523 | 1003.3 | 619.9800 | 1 | 54.06 | 1684 | 1 | 18 | 22 |  |
| R.SNGIT.F | Y | 16.75 | 490.2387 | 2129.6 | 492.2900 | 1 | 66.67 | 2116 | 1 | 157 | 161 |  |
| total 2 peptides |  |  |  |  |  |  |  |  |  |  |  |  |
| --- | --- | --- | --- | --- | --- | --- | --- | --- | --- | --- | --- | --- |

tr|A0A0C4KIU8|A0A0C4KIU8\_9DIPT

back to list

  

| Protein Coverage
| Supporting Peptides
|

Protein Coverage:

Supporting Peptides:

| Peptide | Uniq | -10lgP | Mass | ppm | m/z | z | RT | Scan | #Spec | Start | End | PTM |
| --- | --- | --- | --- | --- | --- | --- | --- | --- | --- | --- | --- | --- |
| L.SMLIR.A | N | 16.79 | 618.3523 | 1003.3 | 619.9800 | 1 | 54.06 | 1684 | 1 | 18 | 22 |  |
| R.SNGIT.F | Y | 16.75 | 490.2387 | 2129.6 | 492.2900 | 1 | 66.67 | 2116 | 1 | 157 | 161 |  |
| total 2 peptides |  |  |  |  |  |  |  |  |  |  |  |  |
| --- | --- | --- | --- | --- | --- | --- | --- | --- | --- | --- | --- | --- |

tr|A0A0C4KIV7|A0A0C4KIV7\_9DIPT

back to list

  

| Protein Coverage
| Supporting Peptides
|

Protein Coverage:

Supporting Peptides:

| Peptide | Uniq | -10lgP | Mass | ppm | m/z | z | RT | Scan | #Spec | Start | End | PTM |
| --- | --- | --- | --- | --- | --- | --- | --- | --- | --- | --- | --- | --- |
| L.SMLIR.A | N | 16.79 | 618.3523 | 1003.3 | 619.9800 | 1 | 54.06 | 1684 | 1 | 18 | 22 |  |
| R.SNGIT.F | Y | 16.75 | 490.2387 | 2129.6 | 492.2900 | 1 | 66.67 | 2116 | 1 | 157 | 161 |  |
| total 2 peptides |  |  |  |  |  |  |  |  |  |  |  |  |
| --- | --- | --- | --- | --- | --- | --- | --- | --- | --- | --- | --- | --- |

tr|A0A0C4KIG5|A0A0C4KIG5\_9DIPT

back to list

  

| Protein Coverage
| Supporting Peptides
|

Protein Coverage:

Supporting Peptides:

| Peptide | Uniq | -10lgP | Mass | ppm | m/z | z | RT | Scan | #Spec | Start | End | PTM |
| --- | --- | --- | --- | --- | --- | --- | --- | --- | --- | --- | --- | --- |
| L.SMLIR.A | N | 16.79 | 618.3523 | 1003.3 | 619.9800 | 1 | 54.06 | 1684 | 1 | 18 | 22 |  |
| R.SNGIT.F | Y | 16.75 | 490.2387 | 2129.6 | 492.2900 | 1 | 66.67 | 2116 | 1 | 157 | 161 |  |
| total 2 peptides |  |  |  |  |  |  |  |  |  |  |  |  |
| --- | --- | --- | --- | --- | --- | --- | --- | --- | --- | --- | --- | --- |

tr|A0A0C4KKI8|A0A0C4KKI8\_SIMVI

back to list

  

| Protein Coverage
| Supporting Peptides
|

Protein Coverage:

Supporting Peptides:

| Peptide | Uniq | -10lgP | Mass | ppm | m/z | z | RT | Scan | #Spec | Start | End | PTM |
| --- | --- | --- | --- | --- | --- | --- | --- | --- | --- | --- | --- | --- |
| L.SMLIR.A | N | 16.79 | 618.3523 | 1003.3 | 619.9800 | 1 | 54.06 | 1684 | 1 | 18 | 22 |  |
| R.SNGIT.F | Y | 16.75 | 490.2387 | 2129.6 | 492.2900 | 1 | 66.67 | 2116 | 1 | 157 | 161 |  |
| total 2 peptides |  |  |  |  |  |  |  |  |  |  |  |  |
| --- | --- | --- | --- | --- | --- | --- | --- | --- | --- | --- | --- | --- |

tr|A0A0C4KQN7|A0A0C4KQN7\_9DIPT

back to list

  

| Protein Coverage
| Supporting Peptides
|

Protein Coverage:

Supporting Peptides:

| Peptide | Uniq | -10lgP | Mass | ppm | m/z | z | RT | Scan | #Spec | Start | End | PTM |
| --- | --- | --- | --- | --- | --- | --- | --- | --- | --- | --- | --- | --- |
| L.SMLIR.A | N | 16.79 | 618.3523 | 1003.3 | 619.9800 | 1 | 54.06 | 1684 | 1 | 18 | 22 |  |
| R.SNGIT.F | Y | 16.75 | 490.2387 | 2129.6 | 492.2900 | 1 | 66.67 | 2116 | 1 | 157 | 161 |  |
| total 2 peptides |  |  |  |  |  |  |  |  |  |  |  |  |
| --- | --- | --- | --- | --- | --- | --- | --- | --- | --- | --- | --- | --- |

tr|A0A0C4KUG1|A0A0C4KUG1\_9DIPT

back to list

  

| Protein Coverage
| Supporting Peptides
|

Protein Coverage:

Supporting Peptides:

| Peptide | Uniq | -10lgP | Mass | ppm | m/z | z | RT | Scan | #Spec | Start | End | PTM |
| --- | --- | --- | --- | --- | --- | --- | --- | --- | --- | --- | --- | --- |
| L.SMLIR.A | N | 16.79 | 618.3523 | 1003.3 | 619.9800 | 1 | 54.06 | 1684 | 1 | 18 | 22 |  |
| R.SNGIT.F | Y | 16.75 | 490.2387 | 2129.6 | 492.2900 | 1 | 66.67 | 2116 | 1 | 157 | 161 |  |
| total 2 peptides |  |  |  |  |  |  |  |  |  |  |  |  |
| --- | --- | --- | --- | --- | --- | --- | --- | --- | --- | --- | --- | --- |

tr|A0A0C4KIU5|A0A0C4KIU5\_9DIPT

back to list

  

| Protein Coverage
| Supporting Peptides
|

Protein Coverage:

Supporting Peptides:

| Peptide | Uniq | -10lgP | Mass | ppm | m/z | z | RT | Scan | #Spec | Start | End | PTM |
| --- | --- | --- | --- | --- | --- | --- | --- | --- | --- | --- | --- | --- |
| L.SMLIR.A | N | 16.79 | 618.3523 | 1003.3 | 619.9800 | 1 | 54.06 | 1684 | 1 | 18 | 22 |  |
| R.SNGIT.F | Y | 16.75 | 490.2387 | 2129.6 | 492.2900 | 1 | 66.67 | 2116 | 1 | 157 | 161 |  |
| total 2 peptides |  |  |  |  |  |  |  |  |  |  |  |  |
| --- | --- | --- | --- | --- | --- | --- | --- | --- | --- | --- | --- | --- |

tr|A0A0C4KUM4|A0A0C4KUM4\_9DIPT

back to list

  

| Protein Coverage
| Supporting Peptides
|

Protein Coverage:

Supporting Peptides:

| Peptide | Uniq | -10lgP | Mass | ppm | m/z | z | RT | Scan | #Spec | Start | End | PTM |
| --- | --- | --- | --- | --- | --- | --- | --- | --- | --- | --- | --- | --- |
| L.SMLIR.A | N | 16.79 | 618.3523 | 1003.3 | 619.9800 | 1 | 54.06 | 1684 | 1 | 18 | 22 |  |
| R.SNGIT.F | Y | 16.75 | 490.2387 | 2129.6 | 492.2900 | 1 | 66.67 | 2116 | 1 | 157 | 161 |  |
| total 2 peptides |  |  |  |  |  |  |  |  |  |  |  |  |
| --- | --- | --- | --- | --- | --- | --- | --- | --- | --- | --- | --- | --- |

tr|A0A0C4KKL2|A0A0C4KKL2\_SIMVI

back to list

  

| Protein Coverage
| Supporting Peptides
|

Protein Coverage:

Supporting Peptides:

| Peptide | Uniq | -10lgP | Mass | ppm | m/z | z | RT | Scan | #Spec | Start | End | PTM |
| --- | --- | --- | --- | --- | --- | --- | --- | --- | --- | --- | --- | --- |
| L.SMLIR.A | N | 16.79 | 618.3523 | 1003.3 | 619.9800 | 1 | 54.06 | 1684 | 1 | 18 | 22 |  |
| R.SNGIT.F | Y | 16.75 | 490.2387 | 2129.6 | 492.2900 | 1 | 66.67 | 2116 | 1 | 157 | 161 |  |
| total 2 peptides |  |  |  |  |  |  |  |  |  |  |  |  |
| --- | --- | --- | --- | --- | --- | --- | --- | --- | --- | --- | --- | --- |

tr|A0A0C4KQT1|A0A0C4KQT1\_SIMVI

back to list

  

| Protein Coverage
| Supporting Peptides
|

Protein Coverage:

Supporting Peptides:

| Peptide | Uniq | -10lgP | Mass | ppm | m/z | z | RT | Scan | #Spec | Start | End | PTM |
| --- | --- | --- | --- | --- | --- | --- | --- | --- | --- | --- | --- | --- |
| L.SMLIR.A | N | 16.79 | 618.3523 | 1003.3 | 619.9800 | 1 | 54.06 | 1684 | 1 | 18 | 22 |  |
| R.SNGIT.F | Y | 16.75 | 490.2387 | 2129.6 | 492.2900 | 1 | 66.67 | 2116 | 1 | 157 | 161 |  |
| total 2 peptides |  |  |  |  |  |  |  |  |  |  |  |  |
| --- | --- | --- | --- | --- | --- | --- | --- | --- | --- | --- | --- | --- |

tr|A0A0C4KQK9|A0A0C4KQK9\_9DIPT

back to list

  

| Protein Coverage
| Supporting Peptides
|

Protein Coverage:

Supporting Peptides:

| Peptide | Uniq | -10lgP | Mass | ppm | m/z | z | RT | Scan | #Spec | Start | End | PTM |
| --- | --- | --- | --- | --- | --- | --- | --- | --- | --- | --- | --- | --- |
| L.SMLIR.A | N | 16.79 | 618.3523 | 1003.3 | 619.9800 | 1 | 54.06 | 1684 | 1 | 18 | 22 |  |
| R.SNGIT.F | Y | 16.75 | 490.2387 | 2129.6 | 492.2900 | 1 | 66.67 | 2116 | 1 | 157 | 161 |  |
| total 2 peptides |  |  |  |  |  |  |  |  |  |  |  |  |
| --- | --- | --- | --- | --- | --- | --- | --- | --- | --- | --- | --- | --- |

tr|A0A0C4KK50|A0A0C4KK50\_9DIPT

back to list

  

| Protein Coverage
| Supporting Peptides
|

Protein Coverage:

Supporting Peptides:

| Peptide | Uniq | -10lgP | Mass | ppm | m/z | z | RT | Scan | #Spec | Start | End | PTM |
| --- | --- | --- | --- | --- | --- | --- | --- | --- | --- | --- | --- | --- |
| L.SMLIR.A | N | 16.79 | 618.3523 | 1003.3 | 619.9800 | 1 | 54.06 | 1684 | 1 | 18 | 22 |  |
| R.SNGIT.F | Y | 16.75 | 490.2387 | 2129.6 | 492.2900 | 1 | 66.67 | 2116 | 1 | 157 | 161 |  |
| total 2 peptides |  |  |  |  |  |  |  |  |  |  |  |  |
| --- | --- | --- | --- | --- | --- | --- | --- | --- | --- | --- | --- | --- |

tr|A0A0C4KVQ1|A0A0C4KVQ1\_9DIPT

back to list

  

| Protein Coverage
| Supporting Peptides
|

Protein Coverage:

Supporting Peptides:

| Peptide | Uniq | -10lgP | Mass | ppm | m/z | z | RT | Scan | #Spec | Start | End | PTM |
| --- | --- | --- | --- | --- | --- | --- | --- | --- | --- | --- | --- | --- |
| L.SMLIR.A | N | 16.79 | 618.3523 | 1003.3 | 619.9800 | 1 | 54.06 | 1684 | 1 | 18 | 22 |  |
| R.SNGIT.F | Y | 16.75 | 490.2387 | 2129.6 | 492.2900 | 1 | 66.67 | 2116 | 1 | 157 | 161 |  |
| total 2 peptides |  |  |  |  |  |  |  |  |  |  |  |  |
| --- | --- | --- | --- | --- | --- | --- | --- | --- | --- | --- | --- | --- |

tr|A0A0C4KUV1|A0A0C4KUV1\_SIMVI

back to list

  

| Protein Coverage
| Supporting Peptides
|

Protein Coverage:

Supporting Peptides:

| Peptide | Uniq | -10lgP | Mass | ppm | m/z | z | RT | Scan | #Spec | Start | End | PTM |
| --- | --- | --- | --- | --- | --- | --- | --- | --- | --- | --- | --- | --- |
| L.SMLIR.A | N | 16.79 | 618.3523 | 1003.3 | 619.9800 | 1 | 54.06 | 1684 | 1 | 18 | 22 |  |
| R.SNGIT.F | Y | 16.75 | 490.2387 | 2129.6 | 492.2900 | 1 | 66.67 | 2116 | 1 | 157 | 161 |  |
| total 2 peptides |  |  |  |  |  |  |  |  |  |  |  |  |
| --- | --- | --- | --- | --- | --- | --- | --- | --- | --- | --- | --- | --- |

tr|A0A0C4KIV2|A0A0C4KIV2\_9DIPT

back to list

  

| Protein Coverage
| Supporting Peptides
|

Protein Coverage:

Supporting Peptides:

| Peptide | Uniq | -10lgP | Mass | ppm | m/z | z | RT | Scan | #Spec | Start | End | PTM |
| --- | --- | --- | --- | --- | --- | --- | --- | --- | --- | --- | --- | --- |
| L.SMLIR.A | N | 16.79 | 618.3523 | 1003.3 | 619.9800 | 1 | 54.06 | 1684 | 1 | 18 | 22 |  |
| R.SNGIT.F | Y | 16.75 | 490.2387 | 2129.6 | 492.2900 | 1 | 66.67 | 2116 | 1 | 157 | 161 |  |
| total 2 peptides |  |  |  |  |  |  |  |  |  |  |  |  |
| --- | --- | --- | --- | --- | --- | --- | --- | --- | --- | --- | --- | --- |

tr|A0A0C4KQR8|A0A0C4KQR8\_SIMVI

back to list

  

| Protein Coverage
| Supporting Peptides
|

Protein Coverage:

Supporting Peptides:

| Peptide | Uniq | -10lgP | Mass | ppm | m/z | z | RT | Scan | #Spec | Start | End | PTM |
| --- | --- | --- | --- | --- | --- | --- | --- | --- | --- | --- | --- | --- |
| L.SMLIR.A | N | 16.79 | 618.3523 | 1003.3 | 619.9800 | 1 | 54.06 | 1684 | 1 | 18 | 22 |  |
| R.SNGIT.F | Y | 16.75 | 490.2387 | 2129.6 | 492.2900 | 1 | 66.67 | 2116 | 1 | 157 | 161 |  |
| total 2 peptides |  |  |  |  |  |  |  |  |  |  |  |  |
| --- | --- | --- | --- | --- | --- | --- | --- | --- | --- | --- | --- | --- |

tr|A0A0C4KVP2|A0A0C4KVP2\_9DIPT

back to list

  

| Protein Coverage
| Supporting Peptides
|

Protein Coverage:

Supporting Peptides:

| Peptide | Uniq | -10lgP | Mass | ppm | m/z | z | RT | Scan | #Spec | Start | End | PTM |
| --- | --- | --- | --- | --- | --- | --- | --- | --- | --- | --- | --- | --- |
| L.SMLIR.A | N | 16.79 | 618.3523 | 1003.3 | 619.9800 | 1 | 54.06 | 1684 | 1 | 18 | 22 |  |
| R.SNGIT.F | Y | 16.75 | 490.2387 | 2129.6 | 492.2900 | 1 | 66.67 | 2116 | 1 | 157 | 161 |  |
| total 2 peptides |  |  |  |  |  |  |  |  |  |  |  |  |
| --- | --- | --- | --- | --- | --- | --- | --- | --- | --- | --- | --- | --- |

tr|A0A0C4KKD0|A0A0C4KKD0\_9DIPT

back to list

  

| Protein Coverage
| Supporting Peptides
|

Protein Coverage:

Supporting Peptides:

| Peptide | Uniq | -10lgP | Mass | ppm | m/z | z | RT | Scan | #Spec | Start | End | PTM |
| --- | --- | --- | --- | --- | --- | --- | --- | --- | --- | --- | --- | --- |
| L.SMLIR.A | N | 16.79 | 618.3523 | 1003.3 | 619.9800 | 1 | 54.06 | 1684 | 1 | 18 | 22 |  |
| R.SNGIT.F | Y | 16.75 | 490.2387 | 2129.6 | 492.2900 | 1 | 66.67 | 2116 | 1 | 157 | 161 |  |
| total 2 peptides |  |  |  |  |  |  |  |  |  |  |  |  |
| --- | --- | --- | --- | --- | --- | --- | --- | --- | --- | --- | --- | --- |

tr|A0A0C4KQP5|A0A0C4KQP5\_SIMVI

back to list

  

| Protein Coverage
| Supporting Peptides
|

Protein Coverage:

Supporting Peptides:

| Peptide | Uniq | -10lgP | Mass | ppm | m/z | z | RT | Scan | #Spec | Start | End | PTM |
| --- | --- | --- | --- | --- | --- | --- | --- | --- | --- | --- | --- | --- |
| L.SMLIR.A | N | 16.79 | 618.3523 | 1003.3 | 619.9800 | 1 | 54.06 | 1684 | 1 | 18 | 22 |  |
| R.SNGIT.F | Y | 16.75 | 490.2387 | 2129.6 | 492.2900 | 1 | 66.67 | 2116 | 1 | 157 | 161 |  |
| total 2 peptides |  |  |  |  |  |  |  |  |  |  |  |  |
| --- | --- | --- | --- | --- | --- | --- | --- | --- | --- | --- | --- | --- |

tr|A0A0C4KVW9|A0A0C4KVW9\_SIMVI

back to list

  

| Protein Coverage
| Supporting Peptides
|

Protein Coverage:

Supporting Peptides:

| Peptide | Uniq | -10lgP | Mass | ppm | m/z | z | RT | Scan | #Spec | Start | End | PTM |
| --- | --- | --- | --- | --- | --- | --- | --- | --- | --- | --- | --- | --- |
| L.SMLIR.A | N | 16.79 | 618.3523 | 1003.3 | 619.9800 | 1 | 54.06 | 1684 | 1 | 18 | 22 |  |
| R.SNGIT.F | Y | 16.75 | 490.2387 | 2129.6 | 492.2900 | 1 | 66.67 | 2116 | 1 | 157 | 161 |  |
| total 2 peptides |  |  |  |  |  |  |  |  |  |  |  |  |
| --- | --- | --- | --- | --- | --- | --- | --- | --- | --- | --- | --- | --- |

tr|A0A0C4KQK3|A0A0C4KQK3\_9DIPT

back to list

  

| Protein Coverage
| Supporting Peptides
|

Protein Coverage:

Supporting Peptides:

| Peptide | Uniq | -10lgP | Mass | ppm | m/z | z | RT | Scan | #Spec | Start | End | PTM |
| --- | --- | --- | --- | --- | --- | --- | --- | --- | --- | --- | --- | --- |
| L.SMLIR.A | N | 16.79 | 618.3523 | 1003.3 | 619.9800 | 1 | 54.06 | 1684 | 1 | 18 | 22 |  |
| R.SNGIT.F | Y | 16.75 | 490.2387 | 2129.6 | 492.2900 | 1 | 66.67 | 2116 | 1 | 157 | 161 |  |
| total 2 peptides |  |  |  |  |  |  |  |  |  |  |  |  |
| --- | --- | --- | --- | --- | --- | --- | --- | --- | --- | --- | --- | --- |

tr|A0A0C4KUX6|A0A0C4KUX6\_SIMVI

back to list

  

| Protein Coverage
| Supporting Peptides
|

Protein Coverage:

Supporting Peptides:

| Peptide | Uniq | -10lgP | Mass | ppm | m/z | z | RT | Scan | #Spec | Start | End | PTM |
| --- | --- | --- | --- | --- | --- | --- | --- | --- | --- | --- | --- | --- |
| L.SMLIR.A | N | 16.79 | 618.3523 | 1003.3 | 619.9800 | 1 | 54.06 | 1684 | 1 | 18 | 22 |  |
| R.SNGIT.F | Y | 16.75 | 490.2387 | 2129.6 | 492.2900 | 1 | 66.67 | 2116 | 1 | 157 | 161 |  |
| total 2 peptides |  |  |  |  |  |  |  |  |  |  |  |  |
| --- | --- | --- | --- | --- | --- | --- | --- | --- | --- | --- | --- | --- |

tr|A0A0C4KIQ1|A0A0C4KIQ1\_9DIPT

back to list

  

| Protein Coverage
| Supporting Peptides
|

Protein Coverage:

Supporting Peptides:

| Peptide | Uniq | -10lgP | Mass | ppm | m/z | z | RT | Scan | #Spec | Start | End | PTM |
| --- | --- | --- | --- | --- | --- | --- | --- | --- | --- | --- | --- | --- |
| L.SMLIR.A | N | 16.79 | 618.3523 | 1003.3 | 619.9800 | 1 | 54.06 | 1684 | 1 | 18 | 22 |  |
| R.SNGIT.F | Y | 16.75 | 490.2387 | 2129.6 | 492.2900 | 1 | 66.67 | 2116 | 1 | 157 | 161 |  |
| total 2 peptides |  |  |  |  |  |  |  |  |  |  |  |  |
| --- | --- | --- | --- | --- | --- | --- | --- | --- | --- | --- | --- | --- |

tr|A0A0C4KVR5|A0A0C4KVR5\_9DIPT

back to list

  

| Protein Coverage
| Supporting Peptides
|

Protein Coverage:

Supporting Peptides:

| Peptide | Uniq | -10lgP | Mass | ppm | m/z | z | RT | Scan | #Spec | Start | End | PTM |
| --- | --- | --- | --- | --- | --- | --- | --- | --- | --- | --- | --- | --- |
| L.SMLIR.A | N | 16.79 | 618.3523 | 1003.3 | 619.9800 | 1 | 54.06 | 1684 | 1 | 18 | 22 |  |
| R.SNGIT.F | Y | 16.75 | 490.2387 | 2129.6 | 492.2900 | 1 | 66.67 | 2116 | 1 | 157 | 161 |  |
| total 2 peptides |  |  |  |  |  |  |  |  |  |  |  |  |
| --- | --- | --- | --- | --- | --- | --- | --- | --- | --- | --- | --- | --- |

tr|A0A059T2B6|A0A059T2B6\_9DIPT

back to list

  

| Protein Coverage
| Supporting Peptides
|

Protein Coverage:

Supporting Peptides:

| Peptide | Uniq | -10lgP | Mass | ppm | m/z | z | RT | Scan | #Spec | Start | End | PTM |
| --- | --- | --- | --- | --- | --- | --- | --- | --- | --- | --- | --- | --- |
| L.SMLIR.A | N | 16.79 | 618.3523 | 1003.3 | 619.9800 | 1 | 54.06 | 1684 | 1 | 18 | 22 |  |
| R.SNGIT.F | Y | 16.75 | 490.2387 | 2129.6 | 492.2900 | 1 | 66.67 | 2116 | 1 | 157 | 161 |  |
| total 2 peptides |  |  |  |  |  |  |  |  |  |  |  |  |
| --- | --- | --- | --- | --- | --- | --- | --- | --- | --- | --- | --- | --- |

tr|A0A059T209|A0A059T209\_9DIPT

back to list

  

| Protein Coverage
| Supporting Peptides
|

Protein Coverage:

Supporting Peptides:

| Peptide | Uniq | -10lgP | Mass | ppm | m/z | z | RT | Scan | #Spec | Start | End | PTM |
| --- | --- | --- | --- | --- | --- | --- | --- | --- | --- | --- | --- | --- |
| L.SMLIR.A | N | 16.79 | 618.3523 | 1003.3 | 619.9800 | 1 | 54.06 | 1684 | 1 | 18 | 22 |  |
| R.SNGIT.F | Y | 16.75 | 490.2387 | 2129.6 | 492.2900 | 1 | 66.67 | 2116 | 1 | 157 | 161 |  |
| total 2 peptides |  |  |  |  |  |  |  |  |  |  |  |  |
| --- | --- | --- | --- | --- | --- | --- | --- | --- | --- | --- | --- | --- |

tr|A0A059T1R5|A0A059T1R5\_9DIPT

back to list

  

| Protein Coverage
| Supporting Peptides
|

Protein Coverage:

Supporting Peptides:

| Peptide | Uniq | -10lgP | Mass | ppm | m/z | z | RT | Scan | #Spec | Start | End | PTM |
| --- | --- | --- | --- | --- | --- | --- | --- | --- | --- | --- | --- | --- |
| L.SMLIR.A | N | 16.79 | 618.3523 | 1003.3 | 619.9800 | 1 | 54.06 | 1684 | 1 | 18 | 22 |  |
| R.SNGIT.F | Y | 16.75 | 490.2387 | 2129.6 | 492.2900 | 1 | 66.67 | 2116 | 1 | 157 | 161 |  |
| total 2 peptides |  |  |  |  |  |  |  |  |  |  |  |  |
| --- | --- | --- | --- | --- | --- | --- | --- | --- | --- | --- | --- | --- |

tr|A0A059T279|A0A059T279\_9DIPT

back to list

  

| Protein Coverage
| Supporting Peptides
|

Protein Coverage:

Supporting Peptides:

| Peptide | Uniq | -10lgP | Mass | ppm | m/z | z | RT | Scan | #Spec | Start | End | PTM |
| --- | --- | --- | --- | --- | --- | --- | --- | --- | --- | --- | --- | --- |
| L.SMLIR.A | N | 16.79 | 618.3523 | 1003.3 | 619.9800 | 1 | 54.06 | 1684 | 1 | 18 | 22 |  |
| R.SNGIT.F | Y | 16.75 | 490.2387 | 2129.6 | 492.2900 | 1 | 66.67 | 2116 | 1 | 157 | 161 |  |
| total 2 peptides |  |  |  |  |  |  |  |  |  |  |  |  |
| --- | --- | --- | --- | --- | --- | --- | --- | --- | --- | --- | --- | --- |

tr|A0A059T276|A0A059T276\_9DIPT

back to list

  

| Protein Coverage
| Supporting Peptides
|

Protein Coverage:

Supporting Peptides:

| Peptide | Uniq | -10lgP | Mass | ppm | m/z | z | RT | Scan | #Spec | Start | End | PTM |
| --- | --- | --- | --- | --- | --- | --- | --- | --- | --- | --- | --- | --- |
| L.SMLIR.A | N | 16.79 | 618.3523 | 1003.3 | 619.9800 | 1 | 54.06 | 1684 | 1 | 18 | 22 |  |
| R.SNGIT.F | Y | 16.75 | 490.2387 | 2129.6 | 492.2900 | 1 | 66.67 | 2116 | 1 | 157 | 161 |  |
| total 2 peptides |  |  |  |  |  |  |  |  |  |  |  |  |
| --- | --- | --- | --- | --- | --- | --- | --- | --- | --- | --- | --- | --- |

tr|A0A059T288|A0A059T288\_9DIPT

back to list

  

| Protein Coverage
| Supporting Peptides
|

Protein Coverage:

Supporting Peptides:

| Peptide | Uniq | -10lgP | Mass | ppm | m/z | z | RT | Scan | #Spec | Start | End | PTM |
| --- | --- | --- | --- | --- | --- | --- | --- | --- | --- | --- | --- | --- |
| L.SMLIR.A | N | 16.79 | 618.3523 | 1003.3 | 619.9800 | 1 | 54.06 | 1684 | 1 | 18 | 22 |  |
| R.SNGIT.F | Y | 16.75 | 490.2387 | 2129.6 | 492.2900 | 1 | 66.67 | 2116 | 1 | 157 | 161 |  |
| total 2 peptides |  |  |  |  |  |  |  |  |  |  |  |  |
| --- | --- | --- | --- | --- | --- | --- | --- | --- | --- | --- | --- | --- |

tr|A0A059T1S6|A0A059T1S6\_9DIPT

back to list

  

| Protein Coverage
| Supporting Peptides
|

Protein Coverage:

Supporting Peptides:

| Peptide | Uniq | -10lgP | Mass | ppm | m/z | z | RT | Scan | #Spec | Start | End | PTM |
| --- | --- | --- | --- | --- | --- | --- | --- | --- | --- | --- | --- | --- |
| L.SMLIR.A | N | 16.79 | 618.3523 | 1003.3 | 619.9800 | 1 | 54.06 | 1684 | 1 | 18 | 22 |  |
| R.SNGIT.F | Y | 16.75 | 490.2387 | 2129.6 | 492.2900 | 1 | 66.67 | 2116 | 1 | 157 | 161 |  |
| total 2 peptides |  |  |  |  |  |  |  |  |  |  |  |  |
| --- | --- | --- | --- | --- | --- | --- | --- | --- | --- | --- | --- | --- |

tr|A0A059T2A1|A0A059T2A1\_9DIPT

back to list

  

| Protein Coverage
| Supporting Peptides
|

Protein Coverage:

Supporting Peptides:

| Peptide | Uniq | -10lgP | Mass | ppm | m/z | z | RT | Scan | #Spec | Start | End | PTM |
| --- | --- | --- | --- | --- | --- | --- | --- | --- | --- | --- | --- | --- |
| L.SMLIR.A | N | 16.79 | 618.3523 | 1003.3 | 619.9800 | 1 | 54.06 | 1684 | 1 | 18 | 22 |  |
| R.SNGIT.F | Y | 16.75 | 490.2387 | 2129.6 | 492.2900 | 1 | 66.67 | 2116 | 1 | 157 | 161 |  |
| total 2 peptides |  |  |  |  |  |  |  |  |  |  |  |  |
| --- | --- | --- | --- | --- | --- | --- | --- | --- | --- | --- | --- | --- |

tr|A0A059T236|A0A059T236\_9DIPT

back to list

  

| Protein Coverage
| Supporting Peptides
|

Protein Coverage:

Supporting Peptides:

| Peptide | Uniq | -10lgP | Mass | ppm | m/z | z | RT | Scan | #Spec | Start | End | PTM |
| --- | --- | --- | --- | --- | --- | --- | --- | --- | --- | --- | --- | --- |
| L.SMLIR.A | N | 16.79 | 618.3523 | 1003.3 | 619.9800 | 1 | 54.06 | 1684 | 1 | 18 | 22 |  |
| R.SNGIT.F | Y | 16.75 | 490.2387 | 2129.6 | 492.2900 | 1 | 66.67 | 2116 | 1 | 157 | 161 |  |
| total 2 peptides |  |  |  |  |  |  |  |  |  |  |  |  |
| --- | --- | --- | --- | --- | --- | --- | --- | --- | --- | --- | --- | --- |

tr|A0A059T1Q5|A0A059T1Q5\_9DIPT

back to list

  

| Protein Coverage
| Supporting Peptides
|

Protein Coverage:

Supporting Peptides:

| Peptide | Uniq | -10lgP | Mass | ppm | m/z | z | RT | Scan | #Spec | Start | End | PTM |
| --- | --- | --- | --- | --- | --- | --- | --- | --- | --- | --- | --- | --- |
| L.SMLIR.A | N | 16.79 | 618.3523 | 1003.3 | 619.9800 | 1 | 54.06 | 1684 | 1 | 18 | 22 |  |
| R.SNGIT.F | Y | 16.75 | 490.2387 | 2129.6 | 492.2900 | 1 | 66.67 | 2116 | 1 | 157 | 161 |  |
| total 2 peptides |  |  |  |  |  |  |  |  |  |  |  |  |
| --- | --- | --- | --- | --- | --- | --- | --- | --- | --- | --- | --- | --- |

tr|A0A059T200|A0A059T200\_9DIPT

back to list

  

| Protein Coverage
| Supporting Peptides
|

Protein Coverage:

Supporting Peptides:

| Peptide | Uniq | -10lgP | Mass | ppm | m/z | z | RT | Scan | #Spec | Start | End | PTM |
| --- | --- | --- | --- | --- | --- | --- | --- | --- | --- | --- | --- | --- |
| L.SMLIR.A | N | 16.79 | 618.3523 | 1003.3 | 619.9800 | 1 | 54.06 | 1684 | 1 | 18 | 22 |  |
| R.SNGIT.F | Y | 16.75 | 490.2387 | 2129.6 | 492.2900 | 1 | 66.67 | 2116 | 1 | 157 | 161 |  |
| total 2 peptides |  |  |  |  |  |  |  |  |  |  |  |  |
| --- | --- | --- | --- | --- | --- | --- | --- | --- | --- | --- | --- | --- |

tr|A0A059T1X3|A0A059T1X3\_9DIPT

back to list

  

| Protein Coverage
| Supporting Peptides
|

Protein Coverage:

Supporting Peptides:

| Peptide | Uniq | -10lgP | Mass | ppm | m/z | z | RT | Scan | #Spec | Start | End | PTM |
| --- | --- | --- | --- | --- | --- | --- | --- | --- | --- | --- | --- | --- |
| L.SMLIR.A | N | 16.79 | 618.3523 | 1003.3 | 619.9800 | 1 | 54.06 | 1684 | 1 | 18 | 22 |  |
| R.SNGIT.F | Y | 16.75 | 490.2387 | 2129.6 | 492.2900 | 1 | 66.67 | 2116 | 1 | 157 | 161 |  |
| total 2 peptides |  |  |  |  |  |  |  |  |  |  |  |  |
| --- | --- | --- | --- | --- | --- | --- | --- | --- | --- | --- | --- | --- |

tr|A0A059T1V5|A0A059T1V5\_9DIPT

back to list

  

| Protein Coverage
| Supporting Peptides
|

Protein Coverage:

Supporting Peptides:

| Peptide | Uniq | -10lgP | Mass | ppm | m/z | z | RT | Scan | #Spec | Start | End | PTM |
| --- | --- | --- | --- | --- | --- | --- | --- | --- | --- | --- | --- | --- |
| L.SMLIR.A | N | 16.79 | 618.3523 | 1003.3 | 619.9800 | 1 | 54.06 | 1684 | 1 | 18 | 22 |  |
| R.SNGIT.F | Y | 16.75 | 490.2387 | 2129.6 | 492.2900 | 1 | 66.67 | 2116 | 1 | 157 | 161 |  |
| total 2 peptides |  |  |  |  |  |  |  |  |  |  |  |  |
| --- | --- | --- | --- | --- | --- | --- | --- | --- | --- | --- | --- | --- |

tr|A0A0M3ZEE9|A0A0M3ZEE9\_9DIPT

back to list

  

| Protein Coverage
| Supporting Peptides
|

Protein Coverage:

Supporting Peptides:

| Peptide | Uniq | -10lgP | Mass | ppm | m/z | z | RT | Scan | #Spec | Start | End | PTM |
| --- | --- | --- | --- | --- | --- | --- | --- | --- | --- | --- | --- | --- |
| L.SMLIR.A | N | 16.79 | 618.3523 | 1003.3 | 619.9800 | 1 | 54.06 | 1684 | 1 | 18 | 22 |  |
| R.SNGIT.F | Y | 16.75 | 490.2387 | 2129.6 | 492.2900 | 1 | 66.67 | 2116 | 1 | 157 | 161 |  |
| total 2 peptides |  |  |  |  |  |  |  |  |  |  |  |  |
| --- | --- | --- | --- | --- | --- | --- | --- | --- | --- | --- | --- | --- |

tr|A0A0N9G4L4|A0A0N9G4L4\_SIMVI

back to list

  

| Protein Coverage
| Supporting Peptides
|

Protein Coverage:

Supporting Peptides:

| Peptide | Uniq | -10lgP | Mass | ppm | m/z | z | RT | Scan | #Spec | Start | End | PTM |
| --- | --- | --- | --- | --- | --- | --- | --- | --- | --- | --- | --- | --- |
| L.SMLIR.A | N | 16.79 | 618.3523 | 1003.3 | 619.9800 | 1 | 54.06 | 1684 | 1 | 18 | 22 |  |
| R.SNGIT.F | Y | 16.75 | 490.2387 | 2129.6 | 492.2900 | 1 | 66.67 | 2116 | 1 | 157 | 161 |  |
| total 2 peptides |  |  |  |  |  |  |  |  |  |  |  |  |
| --- | --- | --- | --- | --- | --- | --- | --- | --- | --- | --- | --- | --- |

tr|A0A0P0BPL0|A0A0P0BPL0\_9DIPT

back to list

  

| Protein Coverage
| Supporting Peptides
|

Protein Coverage:

Supporting Peptides:

| Peptide | Uniq | -10lgP | Mass | ppm | m/z | z | RT | Scan | #Spec | Start | End | PTM |
| --- | --- | --- | --- | --- | --- | --- | --- | --- | --- | --- | --- | --- |
| L.SMLIR.A | N | 16.79 | 618.3523 | 1003.3 | 619.9800 | 1 | 54.06 | 1684 | 1 | 18 | 22 |  |
| R.SNGIT.F | Y | 16.75 | 490.2387 | 2129.6 | 492.2900 | 1 | 66.67 | 2116 | 1 | 157 | 161 |  |
| total 2 peptides |  |  |  |  |  |  |  |  |  |  |  |  |
| --- | --- | --- | --- | --- | --- | --- | --- | --- | --- | --- | --- | --- |

tr|A0A0P0BTB1|A0A0P0BTB1\_9DIPT

back to list

  

| Protein Coverage
| Supporting Peptides
|

Protein Coverage:

Supporting Peptides:

| Peptide | Uniq | -10lgP | Mass | ppm | m/z | z | RT | Scan | #Spec | Start | End | PTM |
| --- | --- | --- | --- | --- | --- | --- | --- | --- | --- | --- | --- | --- |
| L.SMLIR.A | N | 16.79 | 618.3523 | 1003.3 | 619.9800 | 1 | 54.06 | 1684 | 1 | 18 | 22 |  |
| R.SNGIT.F | Y | 16.75 | 490.2387 | 2129.6 | 492.2900 | 1 | 66.67 | 2116 | 1 | 157 | 161 |  |
| total 2 peptides |  |  |  |  |  |  |  |  |  |  |  |  |
| --- | --- | --- | --- | --- | --- | --- | --- | --- | --- | --- | --- | --- |

tr|A0A0P0AGA7|A0A0P0AGA7\_9DIPT

back to list

  

| Protein Coverage
| Supporting Peptides
|

Protein Coverage:

Supporting Peptides:

| Peptide | Uniq | -10lgP | Mass | ppm | m/z | z | RT | Scan | #Spec | Start | End | PTM |
| --- | --- | --- | --- | --- | --- | --- | --- | --- | --- | --- | --- | --- |
| L.SMLIR.A | N | 16.79 | 618.3523 | 1003.3 | 619.9800 | 1 | 54.06 | 1684 | 1 | 18 | 22 |  |
| R.SNGIT.F | Y | 16.75 | 490.2387 | 2129.6 | 492.2900 | 1 | 66.67 | 2116 | 1 | 157 | 161 |  |
| total 2 peptides |  |  |  |  |  |  |  |  |  |  |  |  |
| --- | --- | --- | --- | --- | --- | --- | --- | --- | --- | --- | --- | --- |

tr|A0A0N9FVC2|A0A0N9FVC2\_SIMVI

back to list

  

| Protein Coverage
| Supporting Peptides
|

Protein Coverage:

Supporting Peptides:

| Peptide | Uniq | -10lgP | Mass | ppm | m/z | z | RT | Scan | #Spec | Start | End | PTM |
| --- | --- | --- | --- | --- | --- | --- | --- | --- | --- | --- | --- | --- |
| L.SMLIR.A | N | 16.79 | 618.3523 | 1003.3 | 619.9800 | 1 | 54.06 | 1684 | 1 | 18 | 22 |  |
| R.SNGIT.F | Y | 16.75 | 490.2387 | 2129.6 | 492.2900 | 1 | 66.67 | 2116 | 1 | 157 | 161 |  |
| total 2 peptides |  |  |  |  |  |  |  |  |  |  |  |  |
| --- | --- | --- | --- | --- | --- | --- | --- | --- | --- | --- | --- | --- |

tr|A0A0P0B3K8|A0A0P0B3K8\_9DIPT

back to list

  

| Protein Coverage
| Supporting Peptides
|

Protein Coverage:

Supporting Peptides:

| Peptide | Uniq | -10lgP | Mass | ppm | m/z | z | RT | Scan | #Spec | Start | End | PTM |
| --- | --- | --- | --- | --- | --- | --- | --- | --- | --- | --- | --- | --- |
| L.SMLIR.A | N | 16.79 | 618.3523 | 1003.3 | 619.9800 | 1 | 54.06 | 1684 | 1 | 18 | 22 |  |
| R.SNGIT.F | Y | 16.75 | 490.2387 | 2129.6 | 492.2900 | 1 | 66.67 | 2116 | 1 | 157 | 161 |  |
| total 2 peptides |  |  |  |  |  |  |  |  |  |  |  |  |
| --- | --- | --- | --- | --- | --- | --- | --- | --- | --- | --- | --- | --- |

tr|A0A0P0BGV1|A0A0P0BGV1\_9DIPT

back to list

  

| Protein Coverage
| Supporting Peptides
|

Protein Coverage:

Supporting Peptides:

| Peptide | Uniq | -10lgP | Mass | ppm | m/z | z | RT | Scan | #Spec | Start | End | PTM |
| --- | --- | --- | --- | --- | --- | --- | --- | --- | --- | --- | --- | --- |
| L.SMLIR.A | N | 16.79 | 618.3523 | 1003.3 | 619.9800 | 1 | 54.06 | 1684 | 1 | 18 | 22 |  |
| R.SNGIT.F | Y | 16.75 | 490.2387 | 2129.6 | 492.2900 | 1 | 66.67 | 2116 | 1 | 157 | 161 |  |
| total 2 peptides |  |  |  |  |  |  |  |  |  |  |  |  |
| --- | --- | --- | --- | --- | --- | --- | --- | --- | --- | --- | --- | --- |

tr|A0A0R6MJ84|A0A0R6MJ84\_9DIPT

back to list

  

| Protein Coverage
| Supporting Peptides
|

Protein Coverage:

Supporting Peptides:

| Peptide | Uniq | -10lgP | Mass | ppm | m/z | z | RT | Scan | #Spec | Start | End | PTM |
| --- | --- | --- | --- | --- | --- | --- | --- | --- | --- | --- | --- | --- |
| L.SMLIR.A | N | 16.79 | 618.3523 | 1003.3 | 619.9800 | 1 | 54.06 | 1684 | 1 | 18 | 22 |  |
| R.SNGIT.F | Y | 16.75 | 490.2387 | 2129.6 | 492.2900 | 1 | 66.67 | 2116 | 1 | 157 | 161 |  |
| total 2 peptides |  |  |  |  |  |  |  |  |  |  |  |  |
| --- | --- | --- | --- | --- | --- | --- | --- | --- | --- | --- | --- | --- |

tr|A0A0N9FTH1|A0A0N9FTH1\_9DIPT

back to list

  

| Protein Coverage
| Supporting Peptides
|

Protein Coverage:

Supporting Peptides:

| Peptide | Uniq | -10lgP | Mass | ppm | m/z | z | RT | Scan | #Spec | Start | End | PTM |
| --- | --- | --- | --- | --- | --- | --- | --- | --- | --- | --- | --- | --- |
| L.SMLIR.A | N | 16.79 | 618.3523 | 1003.3 | 619.9800 | 1 | 54.06 | 1684 | 1 | 18 | 22 |  |
| R.SNGIT.F | Y | 16.75 | 490.2387 | 2129.6 | 492.2900 | 1 | 66.67 | 2116 | 1 | 157 | 161 |  |
| total 2 peptides |  |  |  |  |  |  |  |  |  |  |  |  |
| --- | --- | --- | --- | --- | --- | --- | --- | --- | --- | --- | --- | --- |

tr|A0A0P0B7G0|A0A0P0B7G0\_9DIPT

back to list

  

| Protein Coverage
| Supporting Peptides
|

Protein Coverage:

Supporting Peptides:

| Peptide | Uniq | -10lgP | Mass | ppm | m/z | z | RT | Scan | #Spec | Start | End | PTM |
| --- | --- | --- | --- | --- | --- | --- | --- | --- | --- | --- | --- | --- |
| L.SMLIR.A | N | 16.79 | 618.3523 | 1003.3 | 619.9800 | 1 | 54.06 | 1684 | 1 | 18 | 22 |  |
| R.SNGIT.F | Y | 16.75 | 490.2387 | 2129.6 | 492.2900 | 1 | 66.67 | 2116 | 1 | 157 | 161 |  |
| total 2 peptides |  |  |  |  |  |  |  |  |  |  |  |  |
| --- | --- | --- | --- | --- | --- | --- | --- | --- | --- | --- | --- | --- |

tr|A0A0P0AC59|A0A0P0AC59\_9DIPT

back to list

  

| Protein Coverage
| Supporting Peptides
|

Protein Coverage:

Supporting Peptides:

| Peptide | Uniq | -10lgP | Mass | ppm | m/z | z | RT | Scan | #Spec | Start | End | PTM |
| --- | --- | --- | --- | --- | --- | --- | --- | --- | --- | --- | --- | --- |
| L.SMLIR.A | N | 16.79 | 618.3523 | 1003.3 | 619.9800 | 1 | 54.06 | 1684 | 1 | 18 | 22 |  |
| R.SNGIT.F | Y | 16.75 | 490.2387 | 2129.6 | 492.2900 | 1 | 66.67 | 2116 | 1 | 157 | 161 |  |
| total 2 peptides |  |  |  |  |  |  |  |  |  |  |  |  |
| --- | --- | --- | --- | --- | --- | --- | --- | --- | --- | --- | --- | --- |

tr|A0A0P0B5Z6|A0A0P0B5Z6\_9DIPT

back to list

  

| Protein Coverage
| Supporting Peptides
|

Protein Coverage:

Supporting Peptides:

| Peptide | Uniq | -10lgP | Mass | ppm | m/z | z | RT | Scan | #Spec | Start | End | PTM |
| --- | --- | --- | --- | --- | --- | --- | --- | --- | --- | --- | --- | --- |
| L.SMLIR.A | N | 16.79 | 618.3523 | 1003.3 | 619.9800 | 1 | 54.06 | 1684 | 1 | 18 | 22 |  |
| R.SNGIT.F | Y | 16.75 | 490.2387 | 2129.6 | 492.2900 | 1 | 66.67 | 2116 | 1 | 157 | 161 |  |
| total 2 peptides |  |  |  |  |  |  |  |  |  |  |  |  |
| --- | --- | --- | --- | --- | --- | --- | --- | --- | --- | --- | --- | --- |

tr|A0A0N7HRP6|A0A0N7HRP6\_9DIPT

back to list

  

| Protein Coverage
| Supporting Peptides
|

Protein Coverage:

Supporting Peptides:

| Peptide | Uniq | -10lgP | Mass | ppm | m/z | z | RT | Scan | #Spec | Start | End | PTM |
| --- | --- | --- | --- | --- | --- | --- | --- | --- | --- | --- | --- | --- |
| L.SMLIR.A | N | 16.79 | 618.3523 | 1003.3 | 619.9800 | 1 | 54.06 | 1684 | 1 | 18 | 22 |  |
| R.SNGIT.F | Y | 16.75 | 490.2387 | 2129.6 | 492.2900 | 1 | 66.67 | 2116 | 1 | 157 | 161 |  |
| total 2 peptides |  |  |  |  |  |  |  |  |  |  |  |  |
| --- | --- | --- | --- | --- | --- | --- | --- | --- | --- | --- | --- | --- |

tr|A0A0P0BKN0|A0A0P0BKN0\_9DIPT

back to list

  

| Protein Coverage
| Supporting Peptides
|

Protein Coverage:

Supporting Peptides:

| Peptide | Uniq | -10lgP | Mass | ppm | m/z | z | RT | Scan | #Spec | Start | End | PTM |
| --- | --- | --- | --- | --- | --- | --- | --- | --- | --- | --- | --- | --- |
| L.SMLIR.A | N | 16.79 | 618.3523 | 1003.3 | 619.9800 | 1 | 54.06 | 1684 | 1 | 18 | 22 |  |
| R.SNGIT.F | Y | 16.75 | 490.2387 | 2129.6 | 492.2900 | 1 | 66.67 | 2116 | 1 | 157 | 161 |  |
| total 2 peptides |  |  |  |  |  |  |  |  |  |  |  |  |
| --- | --- | --- | --- | --- | --- | --- | --- | --- | --- | --- | --- | --- |

tr|A0A0N9FSA0|A0A0N9FSA0\_SIMVI

back to list

  

| Protein Coverage
| Supporting Peptides
|

Protein Coverage:

Supporting Peptides:

| Peptide | Uniq | -10lgP | Mass | ppm | m/z | z | RT | Scan | #Spec | Start | End | PTM |
| --- | --- | --- | --- | --- | --- | --- | --- | --- | --- | --- | --- | --- |
| L.SMLIR.A | N | 16.79 | 618.3523 | 1003.3 | 619.9800 | 1 | 54.06 | 1684 | 1 | 18 | 22 |  |
| R.SNGIT.F | Y | 16.75 | 490.2387 | 2129.6 | 492.2900 | 1 | 66.67 | 2116 | 1 | 157 | 161 |  |
| total 2 peptides |  |  |  |  |  |  |  |  |  |  |  |  |
| --- | --- | --- | --- | --- | --- | --- | --- | --- | --- | --- | --- | --- |

tr|A0A0P0B802|A0A0P0B802\_9DIPT

back to list

  

| Protein Coverage
| Supporting Peptides
|

Protein Coverage:

Supporting Peptides:

| Peptide | Uniq | -10lgP | Mass | ppm | m/z | z | RT | Scan | #Spec | Start | End | PTM |
| --- | --- | --- | --- | --- | --- | --- | --- | --- | --- | --- | --- | --- |
| L.SMLIR.A | N | 16.79 | 618.3523 | 1003.3 | 619.9800 | 1 | 54.06 | 1684 | 1 | 18 | 22 |  |
| R.SNGIT.F | Y | 16.75 | 490.2387 | 2129.6 | 492.2900 | 1 | 66.67 | 2116 | 1 | 157 | 161 |  |
| total 2 peptides |  |  |  |  |  |  |  |  |  |  |  |  |
| --- | --- | --- | --- | --- | --- | --- | --- | --- | --- | --- | --- | --- |

tr|A0A0P0C1B9|A0A0P0C1B9\_9DIPT

back to list

  

| Protein Coverage
| Supporting Peptides
|

Protein Coverage:

Supporting Peptides:

| Peptide | Uniq | -10lgP | Mass | ppm | m/z | z | RT | Scan | #Spec | Start | End | PTM |
| --- | --- | --- | --- | --- | --- | --- | --- | --- | --- | --- | --- | --- |
| L.SMLIR.A | N | 16.79 | 618.3523 | 1003.3 | 619.9800 | 1 | 54.06 | 1684 | 1 | 18 | 22 |  |
| R.SNGIT.F | Y | 16.75 | 490.2387 | 2129.6 | 492.2900 | 1 | 66.67 | 2116 | 1 | 157 | 161 |  |
| total 2 peptides |  |  |  |  |  |  |  |  |  |  |  |  |
| --- | --- | --- | --- | --- | --- | --- | --- | --- | --- | --- | --- | --- |

tr|A0A0N9H6N0|A0A0N9H6N0\_SIMVI

back to list

  

| Protein Coverage
| Supporting Peptides
|

Protein Coverage:

Supporting Peptides:

| Peptide | Uniq | -10lgP | Mass | ppm | m/z | z | RT | Scan | #Spec | Start | End | PTM |
| --- | --- | --- | --- | --- | --- | --- | --- | --- | --- | --- | --- | --- |
| L.SMLIR.A | N | 16.79 | 618.3523 | 1003.3 | 619.9800 | 1 | 54.06 | 1684 | 1 | 18 | 22 |  |
| R.SNGIT.F | Y | 16.75 | 490.2387 | 2129.6 | 492.2900 | 1 | 66.67 | 2116 | 1 | 157 | 161 |  |
| total 2 peptides |  |  |  |  |  |  |  |  |  |  |  |  |
| --- | --- | --- | --- | --- | --- | --- | --- | --- | --- | --- | --- | --- |

tr|A0A0P0BW44|A0A0P0BW44\_9DIPT

back to list

  

| Protein Coverage
| Supporting Peptides
|

Protein Coverage:

Supporting Peptides:

| Peptide | Uniq | -10lgP | Mass | ppm | m/z | z | RT | Scan | #Spec | Start | End | PTM |
| --- | --- | --- | --- | --- | --- | --- | --- | --- | --- | --- | --- | --- |
| L.SMLIR.A | N | 16.79 | 618.3523 | 1003.3 | 619.9800 | 1 | 54.06 | 1684 | 1 | 18 | 22 |  |
| R.SNGIT.F | Y | 16.75 | 490.2387 | 2129.6 | 492.2900 | 1 | 66.67 | 2116 | 1 | 157 | 161 |  |
| total 2 peptides |  |  |  |  |  |  |  |  |  |  |  |  |
| --- | --- | --- | --- | --- | --- | --- | --- | --- | --- | --- | --- | --- |

tr|A0A0N9G389|A0A0N9G389\_SIMVI

back to list

  

| Protein Coverage
| Supporting Peptides
|

Protein Coverage:

Supporting Peptides:

| Peptide | Uniq | -10lgP | Mass | ppm | m/z | z | RT | Scan | #Spec | Start | End | PTM |
| --- | --- | --- | --- | --- | --- | --- | --- | --- | --- | --- | --- | --- |
| L.SMLIR.A | N | 16.79 | 618.3523 | 1003.3 | 619.9800 | 1 | 54.06 | 1684 | 1 | 18 | 22 |  |
| R.SNGIT.F | Y | 16.75 | 490.2387 | 2129.6 | 492.2900 | 1 | 66.67 | 2116 | 1 | 157 | 161 |  |
| total 2 peptides |  |  |  |  |  |  |  |  |  |  |  |  |
| --- | --- | --- | --- | --- | --- | --- | --- | --- | --- | --- | --- | --- |

tr|A0A0P0BB57|A0A0P0BB57\_9DIPT

back to list

  

| Protein Coverage
| Supporting Peptides
|

Protein Coverage:

Supporting Peptides:

| Peptide | Uniq | -10lgP | Mass | ppm | m/z | z | RT | Scan | #Spec | Start | End | PTM |
| --- | --- | --- | --- | --- | --- | --- | --- | --- | --- | --- | --- | --- |
| L.SMLIR.A | N | 16.79 | 618.3523 | 1003.3 | 619.9800 | 1 | 54.06 | 1684 | 1 | 18 | 22 |  |
| R.SNGIT.F | Y | 16.75 | 490.2387 | 2129.6 | 492.2900 | 1 | 66.67 | 2116 | 1 | 157 | 161 |  |
| total 2 peptides |  |  |  |  |  |  |  |  |  |  |  |  |
| --- | --- | --- | --- | --- | --- | --- | --- | --- | --- | --- | --- | --- |

tr|A0A0P0B9E8|A0A0P0B9E8\_9DIPT

back to list

  

| Protein Coverage
| Supporting Peptides
|

Protein Coverage:

Supporting Peptides:

| Peptide | Uniq | -10lgP | Mass | ppm | m/z | z | RT | Scan | #Spec | Start | End | PTM |
| --- | --- | --- | --- | --- | --- | --- | --- | --- | --- | --- | --- | --- |
| L.SMLIR.A | N | 16.79 | 618.3523 | 1003.3 | 619.9800 | 1 | 54.06 | 1684 | 1 | 18 | 22 |  |
| R.SNGIT.F | Y | 16.75 | 490.2387 | 2129.6 | 492.2900 | 1 | 66.67 | 2116 | 1 | 157 | 161 |  |
| total 2 peptides |  |  |  |  |  |  |  |  |  |  |  |  |
| --- | --- | --- | --- | --- | --- | --- | --- | --- | --- | --- | --- | --- |

tr|A0A0P0BGZ8|A0A0P0BGZ8\_9DIPT

back to list

  

| Protein Coverage
| Supporting Peptides
|

Protein Coverage:

Supporting Peptides:

| Peptide | Uniq | -10lgP | Mass | ppm | m/z | z | RT | Scan | #Spec | Start | End | PTM |
| --- | --- | --- | --- | --- | --- | --- | --- | --- | --- | --- | --- | --- |
| L.SMLIR.A | N | 16.79 | 618.3523 | 1003.3 | 619.9800 | 1 | 54.06 | 1684 | 1 | 18 | 22 |  |
| R.SNGIT.F | Y | 16.75 | 490.2387 | 2129.6 | 492.2900 | 1 | 66.67 | 2116 | 1 | 157 | 161 |  |
| total 2 peptides |  |  |  |  |  |  |  |  |  |  |  |  |
| --- | --- | --- | --- | --- | --- | --- | --- | --- | --- | --- | --- | --- |

tr|A0A0N7HU16|A0A0N7HU16\_9DIPT

back to list

  

| Protein Coverage
| Supporting Peptides
|

Protein Coverage:

Supporting Peptides:

| Peptide | Uniq | -10lgP | Mass | ppm | m/z | z | RT | Scan | #Spec | Start | End | PTM |
| --- | --- | --- | --- | --- | --- | --- | --- | --- | --- | --- | --- | --- |
| L.SMLIR.A | N | 16.79 | 618.3523 | 1003.3 | 619.9800 | 1 | 54.06 | 1684 | 1 | 18 | 22 |  |
| R.SNGIT.F | Y | 16.75 | 490.2387 | 2129.6 | 492.2900 | 1 | 66.67 | 2116 | 1 | 157 | 161 |  |
| total 2 peptides |  |  |  |  |  |  |  |  |  |  |  |  |
| --- | --- | --- | --- | --- | --- | --- | --- | --- | --- | --- | --- | --- |

tr|A0A0P0BHK7|A0A0P0BHK7\_9DIPT

back to list

  

| Protein Coverage
| Supporting Peptides
|

Protein Coverage:

Supporting Peptides:

| Peptide | Uniq | -10lgP | Mass | ppm | m/z | z | RT | Scan | #Spec | Start | End | PTM |
| --- | --- | --- | --- | --- | --- | --- | --- | --- | --- | --- | --- | --- |
| L.SMLIR.A | N | 16.79 | 618.3523 | 1003.3 | 619.9800 | 1 | 54.06 | 1684 | 1 | 18 | 22 |  |
| R.SNGIT.F | Y | 16.75 | 490.2387 | 2129.6 | 492.2900 | 1 | 66.67 | 2116 | 1 | 157 | 161 |  |
| total 2 peptides |  |  |  |  |  |  |  |  |  |  |  |  |
| --- | --- | --- | --- | --- | --- | --- | --- | --- | --- | --- | --- | --- |

tr|A0A0P0B7K9|A0A0P0B7K9\_9DIPT

back to list

  

| Protein Coverage
| Supporting Peptides
|

Protein Coverage:

Supporting Peptides:

| Peptide | Uniq | -10lgP | Mass | ppm | m/z | z | RT | Scan | #Spec | Start | End | PTM |
| --- | --- | --- | --- | --- | --- | --- | --- | --- | --- | --- | --- | --- |
| L.SMLIR.A | N | 16.79 | 618.3523 | 1003.3 | 619.9800 | 1 | 54.06 | 1684 | 1 | 18 | 22 |  |
| R.SNGIT.F | Y | 16.75 | 490.2387 | 2129.6 | 492.2900 | 1 | 66.67 | 2116 | 1 | 157 | 161 |  |
| total 2 peptides |  |  |  |  |  |  |  |  |  |  |  |  |
| --- | --- | --- | --- | --- | --- | --- | --- | --- | --- | --- | --- | --- |

tr|A0A0R6MN48|A0A0R6MN48\_9DIPT

back to list

  

| Protein Coverage
| Supporting Peptides
|

Protein Coverage:

Supporting Peptides:

| Peptide | Uniq | -10lgP | Mass | ppm | m/z | z | RT | Scan | #Spec | Start | End | PTM |
| --- | --- | --- | --- | --- | --- | --- | --- | --- | --- | --- | --- | --- |
| L.SMLIR.A | N | 16.79 | 618.3523 | 1003.3 | 619.9800 | 1 | 54.06 | 1684 | 1 | 18 | 22 |  |
| R.SNGIT.F | Y | 16.75 | 490.2387 | 2129.6 | 492.2900 | 1 | 66.67 | 2116 | 1 | 157 | 161 |  |
| total 2 peptides |  |  |  |  |  |  |  |  |  |  |  |  |
| --- | --- | --- | --- | --- | --- | --- | --- | --- | --- | --- | --- | --- |

tr|A0A0R6MM96|A0A0R6MM96\_9DIPT

back to list

  

| Protein Coverage
| Supporting Peptides
|

Protein Coverage:

Supporting Peptides:

| Peptide | Uniq | -10lgP | Mass | ppm | m/z | z | RT | Scan | #Spec | Start | End | PTM |
| --- | --- | --- | --- | --- | --- | --- | --- | --- | --- | --- | --- | --- |
| L.SMLIR.A | N | 16.79 | 618.3523 | 1003.3 | 619.9800 | 1 | 54.06 | 1684 | 1 | 18 | 22 |  |
| R.SNGIT.F | Y | 16.75 | 490.2387 | 2129.6 | 492.2900 | 1 | 66.67 | 2116 | 1 | 157 | 161 |  |
| total 2 peptides |  |  |  |  |  |  |  |  |  |  |  |  |
| --- | --- | --- | --- | --- | --- | --- | --- | --- | --- | --- | --- | --- |

tr|A0A0P0AY76|A0A0P0AY76\_9DIPT

back to list

  

| Protein Coverage
| Supporting Peptides
|

Protein Coverage:

Supporting Peptides:

| Peptide | Uniq | -10lgP | Mass | ppm | m/z | z | RT | Scan | #Spec | Start | End | PTM |
| --- | --- | --- | --- | --- | --- | --- | --- | --- | --- | --- | --- | --- |
| L.SMLIR.A | N | 16.79 | 618.3523 | 1003.3 | 619.9800 | 1 | 54.06 | 1684 | 1 | 18 | 22 |  |
| R.SNGIT.F | Y | 16.75 | 490.2387 | 2129.6 | 492.2900 | 1 | 66.67 | 2116 | 1 | 157 | 161 |  |
| total 2 peptides |  |  |  |  |  |  |  |  |  |  |  |  |
| --- | --- | --- | --- | --- | --- | --- | --- | --- | --- | --- | --- | --- |

tr|A0A0N7HN65|A0A0N7HN65\_9DIPT

back to list

  

| Protein Coverage
| Supporting Peptides
|

Protein Coverage:

Supporting Peptides:

| Peptide | Uniq | -10lgP | Mass | ppm | m/z | z | RT | Scan | #Spec | Start | End | PTM |
| --- | --- | --- | --- | --- | --- | --- | --- | --- | --- | --- | --- | --- |
| L.SMLIR.A | N | 16.79 | 618.3523 | 1003.3 | 619.9800 | 1 | 54.06 | 1684 | 1 | 18 | 22 |  |
| R.SNGIT.F | Y | 16.75 | 490.2387 | 2129.6 | 492.2900 | 1 | 66.67 | 2116 | 1 | 157 | 161 |  |
| total 2 peptides |  |  |  |  |  |  |  |  |  |  |  |  |
| --- | --- | --- | --- | --- | --- | --- | --- | --- | --- | --- | --- | --- |

tr|A0A0P0BQ60|A0A0P0BQ60\_9DIPT

back to list

  

| Protein Coverage
| Supporting Peptides
|

Protein Coverage:

Supporting Peptides:

| Peptide | Uniq | -10lgP | Mass | ppm | m/z | z | RT | Scan | #Spec | Start | End | PTM |
| --- | --- | --- | --- | --- | --- | --- | --- | --- | --- | --- | --- | --- |
| L.SMLIR.A | N | 16.79 | 618.3523 | 1003.3 | 619.9800 | 1 | 54.06 | 1684 | 1 | 18 | 22 |  |
| R.SNGIT.F | Y | 16.75 | 490.2387 | 2129.6 | 492.2900 | 1 | 66.67 | 2116 | 1 | 157 | 161 |  |
| total 2 peptides |  |  |  |  |  |  |  |  |  |  |  |  |
| --- | --- | --- | --- | --- | --- | --- | --- | --- | --- | --- | --- | --- |

tr|A0A0P0AU82|A0A0P0AU82\_9DIPT

back to list

  

| Protein Coverage
| Supporting Peptides
|

Protein Coverage:

Supporting Peptides:

| Peptide | Uniq | -10lgP | Mass | ppm | m/z | z | RT | Scan | #Spec | Start | End | PTM |
| --- | --- | --- | --- | --- | --- | --- | --- | --- | --- | --- | --- | --- |
| L.SMLIR.A | N | 16.79 | 618.3523 | 1003.3 | 619.9800 | 1 | 54.06 | 1684 | 1 | 18 | 22 |  |
| R.SNGIT.F | Y | 16.75 | 490.2387 | 2129.6 | 492.2900 | 1 | 66.67 | 2116 | 1 | 157 | 161 |  |
| total 2 peptides |  |  |  |  |  |  |  |  |  |  |  |  |
| --- | --- | --- | --- | --- | --- | --- | --- | --- | --- | --- | --- | --- |

tr|A0A0P0BZT2|A0A0P0BZT2\_9DIPT

back to list

  

| Protein Coverage
| Supporting Peptides
|

Protein Coverage:

Supporting Peptides:

| Peptide | Uniq | -10lgP | Mass | ppm | m/z | z | RT | Scan | #Spec | Start | End | PTM |
| --- | --- | --- | --- | --- | --- | --- | --- | --- | --- | --- | --- | --- |
| L.SMLIR.A | N | 16.79 | 618.3523 | 1003.3 | 619.9800 | 1 | 54.06 | 1684 | 1 | 18 | 22 |  |
| R.SNGIT.F | Y | 16.75 | 490.2387 | 2129.6 | 492.2900 | 1 | 66.67 | 2116 | 1 | 157 | 161 |  |
| total 2 peptides |  |  |  |  |  |  |  |  |  |  |  |  |
| --- | --- | --- | --- | --- | --- | --- | --- | --- | --- | --- | --- | --- |

tr|A0A0P0BBX2|A0A0P0BBX2\_9DIPT

back to list

  

| Protein Coverage
| Supporting Peptides
|

Protein Coverage:

Supporting Peptides:

| Peptide | Uniq | -10lgP | Mass | ppm | m/z | z | RT | Scan | #Spec | Start | End | PTM |
| --- | --- | --- | --- | --- | --- | --- | --- | --- | --- | --- | --- | --- |
| L.SMLIR.A | N | 16.79 | 618.3523 | 1003.3 | 619.9800 | 1 | 54.06 | 1684 | 1 | 18 | 22 |  |
| R.SNGIT.F | Y | 16.75 | 490.2387 | 2129.6 | 492.2900 | 1 | 66.67 | 2116 | 1 | 157 | 161 |  |
| total 2 peptides |  |  |  |  |  |  |  |  |  |  |  |  |
| --- | --- | --- | --- | --- | --- | --- | --- | --- | --- | --- | --- | --- |

tr|A0A0R6MLI4|A0A0R6MLI4\_9DIPT

back to list

  

| Protein Coverage
| Supporting Peptides
|

Protein Coverage:

Supporting Peptides:

| Peptide | Uniq | -10lgP | Mass | ppm | m/z | z | RT | Scan | #Spec | Start | End | PTM |
| --- | --- | --- | --- | --- | --- | --- | --- | --- | --- | --- | --- | --- |
| L.SMLIR.A | N | 16.79 | 618.3523 | 1003.3 | 619.9800 | 1 | 54.06 | 1684 | 1 | 18 | 22 |  |
| R.SNGIT.F | Y | 16.75 | 490.2387 | 2129.6 | 492.2900 | 1 | 66.67 | 2116 | 1 | 157 | 161 |  |
| total 2 peptides |  |  |  |  |  |  |  |  |  |  |  |  |
| --- | --- | --- | --- | --- | --- | --- | --- | --- | --- | --- | --- | --- |

tr|A0A0P0BXR7|A0A0P0BXR7\_9DIPT

back to list

  

| Protein Coverage
| Supporting Peptides
|

Protein Coverage:

Supporting Peptides:

| Peptide | Uniq | -10lgP | Mass | ppm | m/z | z | RT | Scan | #Spec | Start | End | PTM |
| --- | --- | --- | --- | --- | --- | --- | --- | --- | --- | --- | --- | --- |
| L.SMLIR.A | N | 16.79 | 618.3523 | 1003.3 | 619.9800 | 1 | 54.06 | 1684 | 1 | 18 | 22 |  |
| R.SNGIT.F | Y | 16.75 | 490.2387 | 2129.6 | 492.2900 | 1 | 66.67 | 2116 | 1 | 157 | 161 |  |
| total 2 peptides |  |  |  |  |  |  |  |  |  |  |  |  |
| --- | --- | --- | --- | --- | --- | --- | --- | --- | --- | --- | --- | --- |

tr|A0A0P0AUV9|A0A0P0AUV9\_9DIPT

back to list

  

| Protein Coverage
| Supporting Peptides
|

Protein Coverage:

Supporting Peptides:

| Peptide | Uniq | -10lgP | Mass | ppm | m/z | z | RT | Scan | #Spec | Start | End | PTM |
| --- | --- | --- | --- | --- | --- | --- | --- | --- | --- | --- | --- | --- |
| L.SMLIR.A | N | 16.79 | 618.3523 | 1003.3 | 619.9800 | 1 | 54.06 | 1684 | 1 | 18 | 22 |  |
| R.SNGIT.F | Y | 16.75 | 490.2387 | 2129.6 | 492.2900 | 1 | 66.67 | 2116 | 1 | 157 | 161 |  |
| total 2 peptides |  |  |  |  |  |  |  |  |  |  |  |  |
| --- | --- | --- | --- | --- | --- | --- | --- | --- | --- | --- | --- | --- |

tr|A0A0P0B1U2|A0A0P0B1U2\_9DIPT

back to list

  

| Protein Coverage
| Supporting Peptides
|

Protein Coverage:

Supporting Peptides:

| Peptide | Uniq | -10lgP | Mass | ppm | m/z | z | RT | Scan | #Spec | Start | End | PTM |
| --- | --- | --- | --- | --- | --- | --- | --- | --- | --- | --- | --- | --- |
| L.SMLIR.A | N | 16.79 | 618.3523 | 1003.3 | 619.9800 | 1 | 54.06 | 1684 | 1 | 18 | 22 |  |
| R.SNGIT.F | Y | 16.75 | 490.2387 | 2129.6 | 492.2900 | 1 | 66.67 | 2116 | 1 | 157 | 161 |  |
| total 2 peptides |  |  |  |  |  |  |  |  |  |  |  |  |
| --- | --- | --- | --- | --- | --- | --- | --- | --- | --- | --- | --- | --- |

tr|A0A0P0BG39|A0A0P0BG39\_9DIPT

back to list

  

| Protein Coverage
| Supporting Peptides
|

Protein Coverage:

Supporting Peptides:

| Peptide | Uniq | -10lgP | Mass | ppm | m/z | z | RT | Scan | #Spec | Start | End | PTM |
| --- | --- | --- | --- | --- | --- | --- | --- | --- | --- | --- | --- | --- |
| L.SMLIR.A | N | 16.79 | 618.3523 | 1003.3 | 619.9800 | 1 | 54.06 | 1684 | 1 | 18 | 22 |  |
| R.SNGIT.F | Y | 16.75 | 490.2387 | 2129.6 | 492.2900 | 1 | 66.67 | 2116 | 1 | 157 | 161 |  |
| total 2 peptides |  |  |  |  |  |  |  |  |  |  |  |  |
| --- | --- | --- | --- | --- | --- | --- | --- | --- | --- | --- | --- | --- |

tr|A0A0P0BEY2|A0A0P0BEY2\_9DIPT

back to list

  

| Protein Coverage
| Supporting Peptides
|

Protein Coverage:

Supporting Peptides:

| Peptide | Uniq | -10lgP | Mass | ppm | m/z | z | RT | Scan | #Spec | Start | End | PTM |
| --- | --- | --- | --- | --- | --- | --- | --- | --- | --- | --- | --- | --- |
| L.SMLIR.A | N | 16.79 | 618.3523 | 1003.3 | 619.9800 | 1 | 54.06 | 1684 | 1 | 18 | 22 |  |
| R.SNGIT.F | Y | 16.75 | 490.2387 | 2129.6 | 492.2900 | 1 | 66.67 | 2116 | 1 | 157 | 161 |  |
| total 2 peptides |  |  |  |  |  |  |  |  |  |  |  |  |
| --- | --- | --- | --- | --- | --- | --- | --- | --- | --- | --- | --- | --- |

tr|A0A0P0BBW3|A0A0P0BBW3\_9DIPT

back to list

  

| Protein Coverage
| Supporting Peptides
|

Protein Coverage:

Supporting Peptides:

| Peptide | Uniq | -10lgP | Mass | ppm | m/z | z | RT | Scan | #Spec | Start | End | PTM |
| --- | --- | --- | --- | --- | --- | --- | --- | --- | --- | --- | --- | --- |
| L.SMLIR.A | N | 16.79 | 618.3523 | 1003.3 | 619.9800 | 1 | 54.06 | 1684 | 1 | 18 | 22 |  |
| R.SNGIT.F | Y | 16.75 | 490.2387 | 2129.6 | 492.2900 | 1 | 66.67 | 2116 | 1 | 157 | 161 |  |
| total 2 peptides |  |  |  |  |  |  |  |  |  |  |  |  |
| --- | --- | --- | --- | --- | --- | --- | --- | --- | --- | --- | --- | --- |

tr|A0A0P0AY79|A0A0P0AY79\_9DIPT

back to list

  

| Protein Coverage
| Supporting Peptides
|

Protein Coverage:

Supporting Peptides:

| Peptide | Uniq | -10lgP | Mass | ppm | m/z | z | RT | Scan | #Spec | Start | End | PTM |
| --- | --- | --- | --- | --- | --- | --- | --- | --- | --- | --- | --- | --- |
| L.SMLIR.A | N | 16.79 | 618.3523 | 1003.3 | 619.9800 | 1 | 54.06 | 1684 | 1 | 18 | 22 |  |
| R.SNGIT.F | Y | 16.75 | 490.2387 | 2129.6 | 492.2900 | 1 | 66.67 | 2116 | 1 | 157 | 161 |  |
| total 2 peptides |  |  |  |  |  |  |  |  |  |  |  |  |
| --- | --- | --- | --- | --- | --- | --- | --- | --- | --- | --- | --- | --- |

tr|A0A0P0BJD9|A0A0P0BJD9\_9DIPT

back to list

  

| Protein Coverage
| Supporting Peptides
|

Protein Coverage:

Supporting Peptides:

| Peptide | Uniq | -10lgP | Mass | ppm | m/z | z | RT | Scan | #Spec | Start | End | PTM |
| --- | --- | --- | --- | --- | --- | --- | --- | --- | --- | --- | --- | --- |
| L.SMLIR.A | N | 16.79 | 618.3523 | 1003.3 | 619.9800 | 1 | 54.06 | 1684 | 1 | 18 | 22 |  |
| R.SNGIT.F | Y | 16.75 | 490.2387 | 2129.6 | 492.2900 | 1 | 66.67 | 2116 | 1 | 157 | 161 |  |
| total 2 peptides |  |  |  |  |  |  |  |  |  |  |  |  |
| --- | --- | --- | --- | --- | --- | --- | --- | --- | --- | --- | --- | --- |

tr|A0A0P0BHY9|A0A0P0BHY9\_9DIPT

back to list

  

| Protein Coverage
| Supporting Peptides
|

Protein Coverage:

Supporting Peptides:

| Peptide | Uniq | -10lgP | Mass | ppm | m/z | z | RT | Scan | #Spec | Start | End | PTM |
| --- | --- | --- | --- | --- | --- | --- | --- | --- | --- | --- | --- | --- |
| L.SMLIR.A | N | 16.79 | 618.3523 | 1003.3 | 619.9800 | 1 | 54.06 | 1684 | 1 | 18 | 22 |  |
| R.SNGIT.F | Y | 16.75 | 490.2387 | 2129.6 | 492.2900 | 1 | 66.67 | 2116 | 1 | 157 | 161 |  |
| total 2 peptides |  |  |  |  |  |  |  |  |  |  |  |  |
| --- | --- | --- | --- | --- | --- | --- | --- | --- | --- | --- | --- | --- |

tr|A0A0P0B5D2|A0A0P0B5D2\_9DIPT

back to list

  

| Protein Coverage
| Supporting Peptides
|

Protein Coverage:

Supporting Peptides:

| Peptide | Uniq | -10lgP | Mass | ppm | m/z | z | RT | Scan | #Spec | Start | End | PTM |
| --- | --- | --- | --- | --- | --- | --- | --- | --- | --- | --- | --- | --- |
| L.SMLIR.A | N | 16.79 | 618.3523 | 1003.3 | 619.9800 | 1 | 54.06 | 1684 | 1 | 18 | 22 |  |
| R.SNGIT.F | Y | 16.75 | 490.2387 | 2129.6 | 492.2900 | 1 | 66.67 | 2116 | 1 | 157 | 161 |  |
| total 2 peptides |  |  |  |  |  |  |  |  |  |  |  |  |
| --- | --- | --- | --- | --- | --- | --- | --- | --- | --- | --- | --- | --- |

tr|A0A0P0BQN5|A0A0P0BQN5\_9DIPT

back to list

  

| Protein Coverage
| Supporting Peptides
|

Protein Coverage:

Supporting Peptides:

| Peptide | Uniq | -10lgP | Mass | ppm | m/z | z | RT | Scan | #Spec | Start | End | PTM |
| --- | --- | --- | --- | --- | --- | --- | --- | --- | --- | --- | --- | --- |
| L.SMLIR.A | N | 16.79 | 618.3523 | 1003.3 | 619.9800 | 1 | 54.06 | 1684 | 1 | 18 | 22 |  |
| R.SNGIT.F | Y | 16.75 | 490.2387 | 2129.6 | 492.2900 | 1 | 66.67 | 2116 | 1 | 157 | 161 |  |
| total 2 peptides |  |  |  |  |  |  |  |  |  |  |  |  |
| --- | --- | --- | --- | --- | --- | --- | --- | --- | --- | --- | --- | --- |

tr|A0A0P0BC13|A0A0P0BC13\_9DIPT

back to list

  

| Protein Coverage
| Supporting Peptides
|

Protein Coverage:

Supporting Peptides:

| Peptide | Uniq | -10lgP | Mass | ppm | m/z | z | RT | Scan | #Spec | Start | End | PTM |
| --- | --- | --- | --- | --- | --- | --- | --- | --- | --- | --- | --- | --- |
| L.SMLIR.A | N | 16.79 | 618.3523 | 1003.3 | 619.9800 | 1 | 54.06 | 1684 | 1 | 18 | 22 |  |
| R.SNGIT.F | Y | 16.75 | 490.2387 | 2129.6 | 492.2900 | 1 | 66.67 | 2116 | 1 | 157 | 161 |  |
| total 2 peptides |  |  |  |  |  |  |  |  |  |  |  |  |
| --- | --- | --- | --- | --- | --- | --- | --- | --- | --- | --- | --- | --- |

tr|A0A0P0B3Q6|A0A0P0B3Q6\_9DIPT

back to list

  

| Protein Coverage
| Supporting Peptides
|

Protein Coverage:

Supporting Peptides:

| Peptide | Uniq | -10lgP | Mass | ppm | m/z | z | RT | Scan | #Spec | Start | End | PTM |
| --- | --- | --- | --- | --- | --- | --- | --- | --- | --- | --- | --- | --- |
| L.SMLIR.A | N | 16.79 | 618.3523 | 1003.3 | 619.9800 | 1 | 54.06 | 1684 | 1 | 18 | 22 |  |
| R.SNGIT.F | Y | 16.75 | 490.2387 | 2129.6 | 492.2900 | 1 | 66.67 | 2116 | 1 | 157 | 161 |  |
| total 2 peptides |  |  |  |  |  |  |  |  |  |  |  |  |
| --- | --- | --- | --- | --- | --- | --- | --- | --- | --- | --- | --- | --- |

tr|A0A0R6MMH7|A0A0R6MMH7\_9DIPT

back to list

  

| Protein Coverage
| Supporting Peptides
|

Protein Coverage:

Supporting Peptides:

| Peptide | Uniq | -10lgP | Mass | ppm | m/z | z | RT | Scan | #Spec | Start | End | PTM |
| --- | --- | --- | --- | --- | --- | --- | --- | --- | --- | --- | --- | --- |
| L.SMLIR.A | N | 16.79 | 618.3523 | 1003.3 | 619.9800 | 1 | 54.06 | 1684 | 1 | 18 | 22 |  |
| R.SNGIT.F | Y | 16.75 | 490.2387 | 2129.6 | 492.2900 | 1 | 66.67 | 2116 | 1 | 157 | 161 |  |
| total 2 peptides |  |  |  |  |  |  |  |  |  |  |  |  |
| --- | --- | --- | --- | --- | --- | --- | --- | --- | --- | --- | --- | --- |

tr|A0A0P0AAN3|A0A0P0AAN3\_9DIPT

back to list

  

| Protein Coverage
| Supporting Peptides
|

Protein Coverage:

Supporting Peptides:

| Peptide | Uniq | -10lgP | Mass | ppm | m/z | z | RT | Scan | #Spec | Start | End | PTM |
| --- | --- | --- | --- | --- | --- | --- | --- | --- | --- | --- | --- | --- |
| L.SMLIR.A | N | 16.79 | 618.3523 | 1003.3 | 619.9800 | 1 | 54.06 | 1684 | 1 | 18 | 22 |  |
| R.SNGIT.F | Y | 16.75 | 490.2387 | 2129.6 | 492.2900 | 1 | 66.67 | 2116 | 1 | 157 | 161 |  |
| total 2 peptides |  |  |  |  |  |  |  |  |  |  |  |  |
| --- | --- | --- | --- | --- | --- | --- | --- | --- | --- | --- | --- | --- |

tr|A0A0P0AUP5|A0A0P0AUP5\_9DIPT

back to list

  

| Protein Coverage
| Supporting Peptides
|

Protein Coverage:

Supporting Peptides:

| Peptide | Uniq | -10lgP | Mass | ppm | m/z | z | RT | Scan | #Spec | Start | End | PTM |
| --- | --- | --- | --- | --- | --- | --- | --- | --- | --- | --- | --- | --- |
| L.SMLIR.A | N | 16.79 | 618.3523 | 1003.3 | 619.9800 | 1 | 54.06 | 1684 | 1 | 18 | 22 |  |
| R.SNGIT.F | Y | 16.75 | 490.2387 | 2129.6 | 492.2900 | 1 | 66.67 | 2116 | 1 | 157 | 161 |  |
| total 2 peptides |  |  |  |  |  |  |  |  |  |  |  |  |
| --- | --- | --- | --- | --- | --- | --- | --- | --- | --- | --- | --- | --- |

tr|A0A0R6MLN2|A0A0R6MLN2\_9DIPT

back to list

  

| Protein Coverage
| Supporting Peptides
|

Protein Coverage:

Supporting Peptides:

| Peptide | Uniq | -10lgP | Mass | ppm | m/z | z | RT | Scan | #Spec | Start | End | PTM |
| --- | --- | --- | --- | --- | --- | --- | --- | --- | --- | --- | --- | --- |
| L.SMLIR.A | N | 16.79 | 618.3523 | 1003.3 | 619.9800 | 1 | 54.06 | 1684 | 1 | 18 | 22 |  |
| R.SNGIT.F | Y | 16.75 | 490.2387 | 2129.6 | 492.2900 | 1 | 66.67 | 2116 | 1 | 157 | 161 |  |
| total 2 peptides |  |  |  |  |  |  |  |  |  |  |  |  |
| --- | --- | --- | --- | --- | --- | --- | --- | --- | --- | --- | --- | --- |

tr|A0A0R6MK33|A0A0R6MK33\_9DIPT

back to list

  

| Protein Coverage
| Supporting Peptides
|

Protein Coverage:

Supporting Peptides:

| Peptide | Uniq | -10lgP | Mass | ppm | m/z | z | RT | Scan | #Spec | Start | End | PTM |
| --- | --- | --- | --- | --- | --- | --- | --- | --- | --- | --- | --- | --- |
| L.SMLIR.A | N | 16.79 | 618.3523 | 1003.3 | 619.9800 | 1 | 54.06 | 1684 | 1 | 18 | 22 |  |
| R.SNGIT.F | Y | 16.75 | 490.2387 | 2129.6 | 492.2900 | 1 | 66.67 | 2116 | 1 | 157 | 161 |  |
| total 2 peptides |  |  |  |  |  |  |  |  |  |  |  |  |
| --- | --- | --- | --- | --- | --- | --- | --- | --- | --- | --- | --- | --- |

tr|A0A0R6MNV2|A0A0R6MNV2\_9DIPT

back to list

  

| Protein Coverage
| Supporting Peptides
|

Protein Coverage:

Supporting Peptides:

| Peptide | Uniq | -10lgP | Mass | ppm | m/z | z | RT | Scan | #Spec | Start | End | PTM |
| --- | --- | --- | --- | --- | --- | --- | --- | --- | --- | --- | --- | --- |
| L.SMLIR.A | N | 16.79 | 618.3523 | 1003.3 | 619.9800 | 1 | 54.06 | 1684 | 1 | 18 | 22 |  |
| R.SNGIT.F | Y | 16.75 | 490.2387 | 2129.6 | 492.2900 | 1 | 66.67 | 2116 | 1 | 157 | 161 |  |
| total 2 peptides |  |  |  |  |  |  |  |  |  |  |  |  |
| --- | --- | --- | --- | --- | --- | --- | --- | --- | --- | --- | --- | --- |

tr|A0A0R6MMX5|A0A0R6MMX5\_9DIPT

back to list

  

| Protein Coverage
| Supporting Peptides
|

Protein Coverage:

Supporting Peptides:

| Peptide | Uniq | -10lgP | Mass | ppm | m/z | z | RT | Scan | #Spec | Start | End | PTM |
| --- | --- | --- | --- | --- | --- | --- | --- | --- | --- | --- | --- | --- |
| L.SMLIR.A | N | 16.79 | 618.3523 | 1003.3 | 619.9800 | 1 | 54.06 | 1684 | 1 | 18 | 22 |  |
| R.SNGIT.F | Y | 16.75 | 490.2387 | 2129.6 | 492.2900 | 1 | 66.67 | 2116 | 1 | 157 | 161 |  |
| total 2 peptides |  |  |  |  |  |  |  |  |  |  |  |  |
| --- | --- | --- | --- | --- | --- | --- | --- | --- | --- | --- | --- | --- |

tr|A0A0R6MM23|A0A0R6MM23\_9DIPT

back to list

  

| Protein Coverage
| Supporting Peptides
|

Protein Coverage:

Supporting Peptides:

| Peptide | Uniq | -10lgP | Mass | ppm | m/z | z | RT | Scan | #Spec | Start | End | PTM |
| --- | --- | --- | --- | --- | --- | --- | --- | --- | --- | --- | --- | --- |
| L.SMLIR.A | N | 16.79 | 618.3523 | 1003.3 | 619.9800 | 1 | 54.06 | 1684 | 1 | 18 | 22 |  |
| R.SNGIT.F | Y | 16.75 | 490.2387 | 2129.6 | 492.2900 | 1 | 66.67 | 2116 | 1 | 157 | 161 |  |
| total 2 peptides |  |  |  |  |  |  |  |  |  |  |  |  |
| --- | --- | --- | --- | --- | --- | --- | --- | --- | --- | --- | --- | --- |

tr|A0A0R6ML68|A0A0R6ML68\_9DIPT

back to list

  

| Protein Coverage
| Supporting Peptides
|

Protein Coverage:

Supporting Peptides:

| Peptide | Uniq | -10lgP | Mass | ppm | m/z | z | RT | Scan | #Spec | Start | End | PTM |
| --- | --- | --- | --- | --- | --- | --- | --- | --- | --- | --- | --- | --- |
| L.SMLIR.A | N | 16.79 | 618.3523 | 1003.3 | 619.9800 | 1 | 54.06 | 1684 | 1 | 18 | 22 |  |
| R.SNGIT.F | Y | 16.75 | 490.2387 | 2129.6 | 492.2900 | 1 | 66.67 | 2116 | 1 | 157 | 161 |  |
| total 2 peptides |  |  |  |  |  |  |  |  |  |  |  |  |
| --- | --- | --- | --- | --- | --- | --- | --- | --- | --- | --- | --- | --- |

tr|A0A0R6MLM8|A0A0R6MLM8\_9DIPT

back to list

  

| Protein Coverage
| Supporting Peptides
|

Protein Coverage:

Supporting Peptides:

| Peptide | Uniq | -10lgP | Mass | ppm | m/z | z | RT | Scan | #Spec | Start | End | PTM |
| --- | --- | --- | --- | --- | --- | --- | --- | --- | --- | --- | --- | --- |
| L.SMLIR.A | N | 16.79 | 618.3523 | 1003.3 | 619.9800 | 1 | 54.06 | 1684 | 1 | 18 | 22 |  |
| R.SNGIT.F | Y | 16.75 | 490.2387 | 2129.6 | 492.2900 | 1 | 66.67 | 2116 | 1 | 157 | 161 |  |
| total 2 peptides |  |  |  |  |  |  |  |  |  |  |  |  |
| --- | --- | --- | --- | --- | --- | --- | --- | --- | --- | --- | --- | --- |

tr|A0A0R6MN43|A0A0R6MN43\_9DIPT

back to list

  

| Protein Coverage
| Supporting Peptides
|

Protein Coverage:

Supporting Peptides:

| Peptide | Uniq | -10lgP | Mass | ppm | m/z | z | RT | Scan | #Spec | Start | End | PTM |
| --- | --- | --- | --- | --- | --- | --- | --- | --- | --- | --- | --- | --- |
| L.SMLIR.A | N | 16.79 | 618.3523 | 1003.3 | 619.9800 | 1 | 54.06 | 1684 | 1 | 18 | 22 |  |
| R.SNGIT.F | Y | 16.75 | 490.2387 | 2129.6 | 492.2900 | 1 | 66.67 | 2116 | 1 | 157 | 161 |  |
| total 2 peptides |  |  |  |  |  |  |  |  |  |  |  |  |
| --- | --- | --- | --- | --- | --- | --- | --- | --- | --- | --- | --- | --- |

tr|A0A0R6MMC8|A0A0R6MMC8\_9DIPT

back to list

  

| Protein Coverage
| Supporting Peptides
|

Protein Coverage:

Supporting Peptides:

| Peptide | Uniq | -10lgP | Mass | ppm | m/z | z | RT | Scan | #Spec | Start | End | PTM |
| --- | --- | --- | --- | --- | --- | --- | --- | --- | --- | --- | --- | --- |
| L.SMLIR.A | N | 16.79 | 618.3523 | 1003.3 | 619.9800 | 1 | 54.06 | 1684 | 1 | 18 | 22 |  |
| R.SNGIT.F | Y | 16.75 | 490.2387 | 2129.6 | 492.2900 | 1 | 66.67 | 2116 | 1 | 157 | 161 |  |
| total 2 peptides |  |  |  |  |  |  |  |  |  |  |  |  |
| --- | --- | --- | --- | --- | --- | --- | --- | --- | --- | --- | --- | --- |

tr|A0A0R6ML96|A0A0R6ML96\_9DIPT

back to list

  

| Protein Coverage
| Supporting Peptides
|

Protein Coverage:

Supporting Peptides:

| Peptide | Uniq | -10lgP | Mass | ppm | m/z | z | RT | Scan | #Spec | Start | End | PTM |
| --- | --- | --- | --- | --- | --- | --- | --- | --- | --- | --- | --- | --- |
| L.SMLIR.A | N | 16.79 | 618.3523 | 1003.3 | 619.9800 | 1 | 54.06 | 1684 | 1 | 18 | 22 |  |
| R.SNGIT.F | Y | 16.75 | 490.2387 | 2129.6 | 492.2900 | 1 | 66.67 | 2116 | 1 | 157 | 161 |  |
| total 2 peptides |  |  |  |  |  |  |  |  |  |  |  |  |
| --- | --- | --- | --- | --- | --- | --- | --- | --- | --- | --- | --- | --- |

tr|A0A0R6MLY7|A0A0R6MLY7\_9DIPT

back to list

  

| Protein Coverage
| Supporting Peptides
|

Protein Coverage:

Supporting Peptides:

| Peptide | Uniq | -10lgP | Mass | ppm | m/z | z | RT | Scan | #Spec | Start | End | PTM |
| --- | --- | --- | --- | --- | --- | --- | --- | --- | --- | --- | --- | --- |
| L.SMLIR.A | N | 16.79 | 618.3523 | 1003.3 | 619.9800 | 1 | 54.06 | 1684 | 1 | 18 | 22 |  |
| R.SNGIT.F | Y | 16.75 | 490.2387 | 2129.6 | 492.2900 | 1 | 66.67 | 2116 | 1 | 157 | 161 |  |
| total 2 peptides |  |  |  |  |  |  |  |  |  |  |  |  |
| --- | --- | --- | --- | --- | --- | --- | --- | --- | --- | --- | --- | --- |

tr|A0A0R6MLQ9|A0A0R6MLQ9\_9DIPT

back to list

  

| Protein Coverage
| Supporting Peptides
|

Protein Coverage:

Supporting Peptides:

| Peptide | Uniq | -10lgP | Mass | ppm | m/z | z | RT | Scan | #Spec | Start | End | PTM |
| --- | --- | --- | --- | --- | --- | --- | --- | --- | --- | --- | --- | --- |
| L.SMLIR.A | N | 16.79 | 618.3523 | 1003.3 | 619.9800 | 1 | 54.06 | 1684 | 1 | 18 | 22 |  |
| R.SNGIT.F | Y | 16.75 | 490.2387 | 2129.6 | 492.2900 | 1 | 66.67 | 2116 | 1 | 157 | 161 |  |
| total 2 peptides |  |  |  |  |  |  |  |  |  |  |  |  |
| --- | --- | --- | --- | --- | --- | --- | --- | --- | --- | --- | --- | --- |

tr|A0A0R6MJ59|A0A0R6MJ59\_9DIPT

back to list

  

| Protein Coverage
| Supporting Peptides
|

Protein Coverage:

Supporting Peptides:

| Peptide | Uniq | -10lgP | Mass | ppm | m/z | z | RT | Scan | #Spec | Start | End | PTM |
| --- | --- | --- | --- | --- | --- | --- | --- | --- | --- | --- | --- | --- |
| L.SMLIR.A | N | 16.79 | 618.3523 | 1003.3 | 619.9800 | 1 | 54.06 | 1684 | 1 | 18 | 22 |  |
| R.SNGIT.F | Y | 16.75 | 490.2387 | 2129.6 | 492.2900 | 1 | 66.67 | 2116 | 1 | 157 | 161 |  |
| total 2 peptides |  |  |  |  |  |  |  |  |  |  |  |  |
| --- | --- | --- | --- | --- | --- | --- | --- | --- | --- | --- | --- | --- |

tr|A0A0R6MLJ4|A0A0R6MLJ4\_9DIPT

back to list

  

| Protein Coverage
| Supporting Peptides
|

Protein Coverage:

Supporting Peptides:

| Peptide | Uniq | -10lgP | Mass | ppm | m/z | z | RT | Scan | #Spec | Start | End | PTM |
| --- | --- | --- | --- | --- | --- | --- | --- | --- | --- | --- | --- | --- |
| L.SMLIR.A | N | 16.79 | 618.3523 | 1003.3 | 619.9800 | 1 | 54.06 | 1684 | 1 | 18 | 22 |  |
| R.SNGIT.F | Y | 16.75 | 490.2387 | 2129.6 | 492.2900 | 1 | 66.67 | 2116 | 1 | 157 | 161 |  |
| total 2 peptides |  |  |  |  |  |  |  |  |  |  |  |  |
| --- | --- | --- | --- | --- | --- | --- | --- | --- | --- | --- | --- | --- |

tr|A0A0R6MM06|A0A0R6MM06\_9DIPT

back to list

  

| Protein Coverage
| Supporting Peptides
|

Protein Coverage:

Supporting Peptides:

| Peptide | Uniq | -10lgP | Mass | ppm | m/z | z | RT | Scan | #Spec | Start | End | PTM |
| --- | --- | --- | --- | --- | --- | --- | --- | --- | --- | --- | --- | --- |
| L.SMLIR.A | N | 16.79 | 618.3523 | 1003.3 | 619.9800 | 1 | 54.06 | 1684 | 1 | 18 | 22 |  |
| R.SNGIT.F | Y | 16.75 | 490.2387 | 2129.6 | 492.2900 | 1 | 66.67 | 2116 | 1 | 157 | 161 |  |
| total 2 peptides |  |  |  |  |  |  |  |  |  |  |  |  |
| --- | --- | --- | --- | --- | --- | --- | --- | --- | --- | --- | --- | --- |

tr|A0A0R6MJD2|A0A0R6MJD2\_9DIPT

back to list

  

| Protein Coverage
| Supporting Peptides
|

Protein Coverage:

Supporting Peptides:

| Peptide | Uniq | -10lgP | Mass | ppm | m/z | z | RT | Scan | #Spec | Start | End | PTM |
| --- | --- | --- | --- | --- | --- | --- | --- | --- | --- | --- | --- | --- |
| L.SMLIR.A | N | 16.79 | 618.3523 | 1003.3 | 619.9800 | 1 | 54.06 | 1684 | 1 | 18 | 22 |  |
| R.SNGIT.F | Y | 16.75 | 490.2387 | 2129.6 | 492.2900 | 1 | 66.67 | 2116 | 1 | 157 | 161 |  |
| total 2 peptides |  |  |  |  |  |  |  |  |  |  |  |  |
| --- | --- | --- | --- | --- | --- | --- | --- | --- | --- | --- | --- | --- |

tr|A0A0R6MMP7|A0A0R6MMP7\_9DIPT

back to list

  

| Protein Coverage
| Supporting Peptides
|

Protein Coverage:

Supporting Peptides:

| Peptide | Uniq | -10lgP | Mass | ppm | m/z | z | RT | Scan | #Spec | Start | End | PTM |
| --- | --- | --- | --- | --- | --- | --- | --- | --- | --- | --- | --- | --- |
| L.SMLIR.A | N | 16.79 | 618.3523 | 1003.3 | 619.9800 | 1 | 54.06 | 1684 | 1 | 18 | 22 |  |
| R.SNGIT.F | Y | 16.75 | 490.2387 | 2129.6 | 492.2900 | 1 | 66.67 | 2116 | 1 | 157 | 161 |  |
| total 2 peptides |  |  |  |  |  |  |  |  |  |  |  |  |
| --- | --- | --- | --- | --- | --- | --- | --- | --- | --- | --- | --- | --- |

tr|A0A0R6MJN2|A0A0R6MJN2\_9DIPT

back to list

  

| Protein Coverage
| Supporting Peptides
|

Protein Coverage:

Supporting Peptides:

| Peptide | Uniq | -10lgP | Mass | ppm | m/z | z | RT | Scan | #Spec | Start | End | PTM |
| --- | --- | --- | --- | --- | --- | --- | --- | --- | --- | --- | --- | --- |
| L.SMLIR.A | N | 16.79 | 618.3523 | 1003.3 | 619.9800 | 1 | 54.06 | 1684 | 1 | 18 | 22 |  |
| R.SNGIT.F | Y | 16.75 | 490.2387 | 2129.6 | 492.2900 | 1 | 66.67 | 2116 | 1 | 157 | 161 |  |
| total 2 peptides |  |  |  |  |  |  |  |  |  |  |  |  |
| --- | --- | --- | --- | --- | --- | --- | --- | --- | --- | --- | --- | --- |

tr|A0A0R6MLC4|A0A0R6MLC4\_9DIPT

back to list

  

| Protein Coverage
| Supporting Peptides
|

Protein Coverage:

Supporting Peptides:

| Peptide | Uniq | -10lgP | Mass | ppm | m/z | z | RT | Scan | #Spec | Start | End | PTM |
| --- | --- | --- | --- | --- | --- | --- | --- | --- | --- | --- | --- | --- |
| L.SMLIR.A | N | 16.79 | 618.3523 | 1003.3 | 619.9800 | 1 | 54.06 | 1684 | 1 | 18 | 22 |  |
| R.SNGIT.F | Y | 16.75 | 490.2387 | 2129.6 | 492.2900 | 1 | 66.67 | 2116 | 1 | 157 | 161 |  |
| total 2 peptides |  |  |  |  |  |  |  |  |  |  |  |  |
| --- | --- | --- | --- | --- | --- | --- | --- | --- | --- | --- | --- | --- |

tr|A0A0R6MLJ5|A0A0R6MLJ5\_9DIPT

back to list

  

| Protein Coverage
| Supporting Peptides
|

Protein Coverage:

Supporting Peptides:

| Peptide | Uniq | -10lgP | Mass | ppm | m/z | z | RT | Scan | #Spec | Start | End | PTM |
| --- | --- | --- | --- | --- | --- | --- | --- | --- | --- | --- | --- | --- |
| L.SMLIR.A | N | 16.79 | 618.3523 | 1003.3 | 619.9800 | 1 | 54.06 | 1684 | 1 | 18 | 22 |  |
| R.SNGIT.F | Y | 16.75 | 490.2387 | 2129.6 | 492.2900 | 1 | 66.67 | 2116 | 1 | 157 | 161 |  |
| total 2 peptides |  |  |  |  |  |  |  |  |  |  |  |  |
| --- | --- | --- | --- | --- | --- | --- | --- | --- | --- | --- | --- | --- |

tr|A0A0R6MJB8|A0A0R6MJB8\_9DIPT

back to list

  

| Protein Coverage
| Supporting Peptides
|

Protein Coverage:

Supporting Peptides:

| Peptide | Uniq | -10lgP | Mass | ppm | m/z | z | RT | Scan | #Spec | Start | End | PTM |
| --- | --- | --- | --- | --- | --- | --- | --- | --- | --- | --- | --- | --- |
| L.SMLIR.A | N | 16.79 | 618.3523 | 1003.3 | 619.9800 | 1 | 54.06 | 1684 | 1 | 18 | 22 |  |
| R.SNGIT.L | Y | 16.75 | 490.2387 | 2129.6 | 492.2900 | 1 | 66.67 | 2116 | 1 | 157 | 161 |  |
| total 2 peptides |  |  |  |  |  |  |  |  |  |  |  |  |
| --- | --- | --- | --- | --- | --- | --- | --- | --- | --- | --- | --- | --- |

tr|A0A0R6MN78|A0A0R6MN78\_9DIPT

back to list

  

| Protein Coverage
| Supporting Peptides
|

Protein Coverage:

Supporting Peptides:

| Peptide | Uniq | -10lgP | Mass | ppm | m/z | z | RT | Scan | #Spec | Start | End | PTM |
| --- | --- | --- | --- | --- | --- | --- | --- | --- | --- | --- | --- | --- |
| L.SMLIR.A | N | 16.79 | 618.3523 | 1003.3 | 619.9800 | 1 | 54.06 | 1684 | 1 | 18 | 22 |  |
| R.SNGIT.F | Y | 16.75 | 490.2387 | 2129.6 | 492.2900 | 1 | 66.67 | 2116 | 1 | 157 | 161 |  |
| total 2 peptides |  |  |  |  |  |  |  |  |  |  |  |  |
| --- | --- | --- | --- | --- | --- | --- | --- | --- | --- | --- | --- | --- |

tr|A0A0R6MIH8|A0A0R6MIH8\_9DIPT

back to list

  

| Protein Coverage
| Supporting Peptides
|

Protein Coverage:

Supporting Peptides:

| Peptide | Uniq | -10lgP | Mass | ppm | m/z | z | RT | Scan | #Spec | Start | End | PTM |
| --- | --- | --- | --- | --- | --- | --- | --- | --- | --- | --- | --- | --- |
| L.SMLIR.A | N | 16.79 | 618.3523 | 1003.3 | 619.9800 | 1 | 54.06 | 1684 | 1 | 18 | 22 |  |
| R.SNGIT.F | Y | 16.75 | 490.2387 | 2129.6 | 492.2900 | 1 | 66.67 | 2116 | 1 | 157 | 161 |  |
| total 2 peptides |  |  |  |  |  |  |  |  |  |  |  |  |
| --- | --- | --- | --- | --- | --- | --- | --- | --- | --- | --- | --- | --- |

tr|A0A0R6MMX3|A0A0R6MMX3\_9DIPT

back to list

  

| Protein Coverage
| Supporting Peptides
|

Protein Coverage:

Supporting Peptides:

| Peptide | Uniq | -10lgP | Mass | ppm | m/z | z | RT | Scan | #Spec | Start | End | PTM |
| --- | --- | --- | --- | --- | --- | --- | --- | --- | --- | --- | --- | --- |
| L.SMLIR.A | N | 16.79 | 618.3523 | 1003.3 | 619.9800 | 1 | 54.06 | 1684 | 1 | 18 | 22 |  |
| R.SNGIT.F | Y | 16.75 | 490.2387 | 2129.6 | 492.2900 | 1 | 66.67 | 2116 | 1 | 157 | 161 |  |
| total 2 peptides |  |  |  |  |  |  |  |  |  |  |  |  |
| --- | --- | --- | --- | --- | --- | --- | --- | --- | --- | --- | --- | --- |

tr|A0A0R6MMZ6|A0A0R6MMZ6\_9DIPT

back to list

  

| Protein Coverage
| Supporting Peptides
|

Protein Coverage:

Supporting Peptides:

| Peptide | Uniq | -10lgP | Mass | ppm | m/z | z | RT | Scan | #Spec | Start | End | PTM |
| --- | --- | --- | --- | --- | --- | --- | --- | --- | --- | --- | --- | --- |
| L.SMLIR.A | N | 16.79 | 618.3523 | 1003.3 | 619.9800 | 1 | 54.06 | 1684 | 1 | 18 | 22 |  |
| R.SNGIT.F | Y | 16.75 | 490.2387 | 2129.6 | 492.2900 | 1 | 66.67 | 2116 | 1 | 157 | 161 |  |
| total 2 peptides |  |  |  |  |  |  |  |  |  |  |  |  |
| --- | --- | --- | --- | --- | --- | --- | --- | --- | --- | --- | --- | --- |

tr|A0A0R6MM51|A0A0R6MM51\_9DIPT

back to list

  

| Protein Coverage
| Supporting Peptides
|

Protein Coverage:

Supporting Peptides:

| Peptide | Uniq | -10lgP | Mass | ppm | m/z | z | RT | Scan | #Spec | Start | End | PTM |
| --- | --- | --- | --- | --- | --- | --- | --- | --- | --- | --- | --- | --- |
| L.SMLIR.A | N | 16.79 | 618.3523 | 1003.3 | 619.9800 | 1 | 54.06 | 1684 | 1 | 18 | 22 |  |
| R.SNGIT.F | Y | 16.75 | 490.2387 | 2129.6 | 492.2900 | 1 | 66.67 | 2116 | 1 | 157 | 161 |  |
| total 2 peptides |  |  |  |  |  |  |  |  |  |  |  |  |
| --- | --- | --- | --- | --- | --- | --- | --- | --- | --- | --- | --- | --- |

tr|A0A0R6MMR3|A0A0R6MMR3\_9DIPT

back to list

  

| Protein Coverage
| Supporting Peptides
|

Protein Coverage:

Supporting Peptides:

| Peptide | Uniq | -10lgP | Mass | ppm | m/z | z | RT | Scan | #Spec | Start | End | PTM |
| --- | --- | --- | --- | --- | --- | --- | --- | --- | --- | --- | --- | --- |
| L.SMLIR.A | N | 16.79 | 618.3523 | 1003.3 | 619.9800 | 1 | 54.06 | 1684 | 1 | 18 | 22 |  |
| R.SNGIT.F | Y | 16.75 | 490.2387 | 2129.6 | 492.2900 | 1 | 66.67 | 2116 | 1 | 157 | 161 |  |
| total 2 peptides |  |  |  |  |  |  |  |  |  |  |  |  |
| --- | --- | --- | --- | --- | --- | --- | --- | --- | --- | --- | --- | --- |

tr|A0A0R6MMM3|A0A0R6MMM3\_9DIPT

back to list

  

| Protein Coverage
| Supporting Peptides
|

Protein Coverage:

Supporting Peptides:

| Peptide | Uniq | -10lgP | Mass | ppm | m/z | z | RT | Scan | #Spec | Start | End | PTM |
| --- | --- | --- | --- | --- | --- | --- | --- | --- | --- | --- | --- | --- |
| L.SMLIR.A | N | 16.79 | 618.3523 | 1003.3 | 619.9800 | 1 | 54.06 | 1684 | 1 | 18 | 22 |  |
| R.SNGIT.F | Y | 16.75 | 490.2387 | 2129.6 | 492.2900 | 1 | 66.67 | 2116 | 1 | 157 | 161 |  |
| total 2 peptides |  |  |  |  |  |  |  |  |  |  |  |  |
| --- | --- | --- | --- | --- | --- | --- | --- | --- | --- | --- | --- | --- |

tr|A0A0R6MIQ7|A0A0R6MIQ7\_9DIPT

back to list

  

| Protein Coverage
| Supporting Peptides
|

Protein Coverage:

Supporting Peptides:

| Peptide | Uniq | -10lgP | Mass | ppm | m/z | z | RT | Scan | #Spec | Start | End | PTM |
| --- | --- | --- | --- | --- | --- | --- | --- | --- | --- | --- | --- | --- |
| L.SMLIR.A | N | 16.79 | 618.3523 | 1003.3 | 619.9800 | 1 | 54.06 | 1684 | 1 | 18 | 22 |  |
| R.SNGIT.F | Y | 16.75 | 490.2387 | 2129.6 | 492.2900 | 1 | 66.67 | 2116 | 1 | 157 | 161 |  |
| total 2 peptides |  |  |  |  |  |  |  |  |  |  |  |  |
| --- | --- | --- | --- | --- | --- | --- | --- | --- | --- | --- | --- | --- |

tr|A0A0R6MIK0|A0A0R6MIK0\_9DIPT

back to list

  

| Protein Coverage
| Supporting Peptides
|

Protein Coverage:

Supporting Peptides:

| Peptide | Uniq | -10lgP | Mass | ppm | m/z | z | RT | Scan | #Spec | Start | End | PTM |
| --- | --- | --- | --- | --- | --- | --- | --- | --- | --- | --- | --- | --- |
| L.SMLIR.A | N | 16.79 | 618.3523 | 1003.3 | 619.9800 | 1 | 54.06 | 1684 | 1 | 18 | 22 |  |
| R.SNGIT.F | Y | 16.75 | 490.2387 | 2129.6 | 492.2900 | 1 | 66.67 | 2116 | 1 | 157 | 161 |  |
| total 2 peptides |  |  |  |  |  |  |  |  |  |  |  |  |
| --- | --- | --- | --- | --- | --- | --- | --- | --- | --- | --- | --- | --- |

tr|A0A0R6MMW5|A0A0R6MMW5\_9DIPT

back to list

  

| Protein Coverage
| Supporting Peptides
|

Protein Coverage:

Supporting Peptides:

| Peptide | Uniq | -10lgP | Mass | ppm | m/z | z | RT | Scan | #Spec | Start | End | PTM |
| --- | --- | --- | --- | --- | --- | --- | --- | --- | --- | --- | --- | --- |
| L.SMLIR.A | N | 16.79 | 618.3523 | 1003.3 | 619.9800 | 1 | 54.06 | 1684 | 1 | 18 | 22 |  |
| R.SNGIT.F | Y | 16.75 | 490.2387 | 2129.6 | 492.2900 | 1 | 66.67 | 2116 | 1 | 157 | 161 |  |
| total 2 peptides |  |  |  |  |  |  |  |  |  |  |  |  |
| --- | --- | --- | --- | --- | --- | --- | --- | --- | --- | --- | --- | --- |

tr|A0A0R6MLU6|A0A0R6MLU6\_9DIPT

back to list

  

| Protein Coverage
| Supporting Peptides
|

Protein Coverage:

Supporting Peptides:

| Peptide | Uniq | -10lgP | Mass | ppm | m/z | z | RT | Scan | #Spec | Start | End | PTM |
| --- | --- | --- | --- | --- | --- | --- | --- | --- | --- | --- | --- | --- |
| L.SMLIR.A | N | 16.79 | 618.3523 | 1003.3 | 619.9800 | 1 | 54.06 | 1684 | 1 | 18 | 22 |  |
| R.SNGIT.F | Y | 16.75 | 490.2387 | 2129.6 | 492.2900 | 1 | 66.67 | 2116 | 1 | 157 | 161 |  |
| total 2 peptides |  |  |  |  |  |  |  |  |  |  |  |  |
| --- | --- | --- | --- | --- | --- | --- | --- | --- | --- | --- | --- | --- |

tr|A0A0R6MMT6|A0A0R6MMT6\_9DIPT

back to list

  

| Protein Coverage
| Supporting Peptides
|

Protein Coverage:

Supporting Peptides:

| Peptide | Uniq | -10lgP | Mass | ppm | m/z | z | RT | Scan | #Spec | Start | End | PTM |
| --- | --- | --- | --- | --- | --- | --- | --- | --- | --- | --- | --- | --- |
| L.SMLIR.A | N | 16.79 | 618.3523 | 1003.3 | 619.9800 | 1 | 54.06 | 1684 | 1 | 18 | 22 |  |
| R.SNGIT.F | Y | 16.75 | 490.2387 | 2129.6 | 492.2900 | 1 | 66.67 | 2116 | 1 | 157 | 161 |  |
| total 2 peptides |  |  |  |  |  |  |  |  |  |  |  |  |
| --- | --- | --- | --- | --- | --- | --- | --- | --- | --- | --- | --- | --- |

tr|A0A0R6MNY2|A0A0R6MNY2\_9DIPT

back to list

  

| Protein Coverage
| Supporting Peptides
|

Protein Coverage:

Supporting Peptides:

| Peptide | Uniq | -10lgP | Mass | ppm | m/z | z | RT | Scan | #Spec | Start | End | PTM |
| --- | --- | --- | --- | --- | --- | --- | --- | --- | --- | --- | --- | --- |
| L.SMLIR.A | N | 16.79 | 618.3523 | 1003.3 | 619.9800 | 1 | 54.06 | 1684 | 1 | 18 | 22 |  |
| R.SNGIT.F | Y | 16.75 | 490.2387 | 2129.6 | 492.2900 | 1 | 66.67 | 2116 | 1 | 157 | 161 |  |
| total 2 peptides |  |  |  |  |  |  |  |  |  |  |  |  |
| --- | --- | --- | --- | --- | --- | --- | --- | --- | --- | --- | --- | --- |

tr|A0A0R6MLP4|A0A0R6MLP4\_9DIPT

back to list

  

| Protein Coverage
| Supporting Peptides
|

Protein Coverage:

Supporting Peptides:

| Peptide | Uniq | -10lgP | Mass | ppm | m/z | z | RT | Scan | #Spec | Start | End | PTM |
| --- | --- | --- | --- | --- | --- | --- | --- | --- | --- | --- | --- | --- |
| L.SMLIR.A | N | 16.79 | 618.3523 | 1003.3 | 619.9800 | 1 | 54.06 | 1684 | 1 | 18 | 22 |  |
| R.SNGIT.F | Y | 16.75 | 490.2387 | 2129.6 | 492.2900 | 1 | 66.67 | 2116 | 1 | 157 | 161 |  |
| total 2 peptides |  |  |  |  |  |  |  |  |  |  |  |  |
| --- | --- | --- | --- | --- | --- | --- | --- | --- | --- | --- | --- | --- |

tr|A0A0R6MM54|A0A0R6MM54\_9DIPT

back to list

  

| Protein Coverage
| Supporting Peptides
|

Protein Coverage:

Supporting Peptides:

| Peptide | Uniq | -10lgP | Mass | ppm | m/z | z | RT | Scan | #Spec | Start | End | PTM |
| --- | --- | --- | --- | --- | --- | --- | --- | --- | --- | --- | --- | --- |
| L.SMLIR.A | N | 16.79 | 618.3523 | 1003.3 | 619.9800 | 1 | 54.06 | 1684 | 1 | 18 | 22 |  |
| R.SNGIT.F | Y | 16.75 | 490.2387 | 2129.6 | 492.2900 | 1 | 66.67 | 2116 | 1 | 157 | 161 |  |
| total 2 peptides |  |  |  |  |  |  |  |  |  |  |  |  |
| --- | --- | --- | --- | --- | --- | --- | --- | --- | --- | --- | --- | --- |

tr|A0A0R6MLH0|A0A0R6MLH0\_9DIPT

back to list

  

| Protein Coverage
| Supporting Peptides
|

Protein Coverage:

Supporting Peptides:

| Peptide | Uniq | -10lgP | Mass | ppm | m/z | z | RT | Scan | #Spec | Start | End | PTM |
| --- | --- | --- | --- | --- | --- | --- | --- | --- | --- | --- | --- | --- |
| L.SMLIR.A | N | 16.79 | 618.3523 | 1003.3 | 619.9800 | 1 | 54.06 | 1684 | 1 | 18 | 22 |  |
| R.SNGIT.F | Y | 16.75 | 490.2387 | 2129.6 | 492.2900 | 1 | 66.67 | 2116 | 1 | 157 | 161 |  |
| total 2 peptides |  |  |  |  |  |  |  |  |  |  |  |  |
| --- | --- | --- | --- | --- | --- | --- | --- | --- | --- | --- | --- | --- |

tr|A0A0R6MMQ7|A0A0R6MMQ7\_9DIPT

back to list

  

| Protein Coverage
| Supporting Peptides
|

Protein Coverage:

Supporting Peptides:

| Peptide | Uniq | -10lgP | Mass | ppm | m/z | z | RT | Scan | #Spec | Start | End | PTM |
| --- | --- | --- | --- | --- | --- | --- | --- | --- | --- | --- | --- | --- |
| L.SMLIR.A | N | 16.79 | 618.3523 | 1003.3 | 619.9800 | 1 | 54.06 | 1684 | 1 | 18 | 22 |  |
| R.SNGIT.F | Y | 16.75 | 490.2387 | 2129.6 | 492.2900 | 1 | 66.67 | 2116 | 1 | 157 | 161 |  |
| total 2 peptides |  |  |  |  |  |  |  |  |  |  |  |  |
| --- | --- | --- | --- | --- | --- | --- | --- | --- | --- | --- | --- | --- |

tr|A0A0R6MM84|A0A0R6MM84\_9DIPT

back to list

  

| Protein Coverage
| Supporting Peptides
|

Protein Coverage:

Supporting Peptides:

| Peptide | Uniq | -10lgP | Mass | ppm | m/z | z | RT | Scan | #Spec | Start | End | PTM |
| --- | --- | --- | --- | --- | --- | --- | --- | --- | --- | --- | --- | --- |
| L.SMLIR.A | N | 16.79 | 618.3523 | 1003.3 | 619.9800 | 1 | 54.06 | 1684 | 1 | 18 | 22 |  |
| R.SNGIT.F | Y | 16.75 | 490.2387 | 2129.6 | 492.2900 | 1 | 66.67 | 2116 | 1 | 157 | 161 |  |
| total 2 peptides |  |  |  |  |  |  |  |  |  |  |  |  |
| --- | --- | --- | --- | --- | --- | --- | --- | --- | --- | --- | --- | --- |

tr|A0A0M3YWA1|A0A0M3YWA1\_9DIPT

back to list

  

| Protein Coverage
| Supporting Peptides
|

Protein Coverage:

Supporting Peptides:

| Peptide | Uniq | -10lgP | Mass | ppm | m/z | z | RT | Scan | #Spec | Start | End | PTM |
| --- | --- | --- | --- | --- | --- | --- | --- | --- | --- | --- | --- | --- |
| L.SMLIR.A | N | 16.79 | 618.3523 | 1003.3 | 619.9800 | 1 | 54.06 | 1684 | 1 | 18 | 22 |  |
| R.SNGIT.F | Y | 16.75 | 490.2387 | 2129.6 | 492.2900 | 1 | 66.67 | 2116 | 1 | 157 | 161 |  |
| total 2 peptides |  |  |  |  |  |  |  |  |  |  |  |  |
| --- | --- | --- | --- | --- | --- | --- | --- | --- | --- | --- | --- | --- |

tr|A0A0R6MLC8|A0A0R6MLC8\_9DIPT

back to list

  

| Protein Coverage
| Supporting Peptides
|

Protein Coverage:

Supporting Peptides:

| Peptide | Uniq | -10lgP | Mass | ppm | m/z | z | RT | Scan | #Spec | Start | End | PTM |
| --- | --- | --- | --- | --- | --- | --- | --- | --- | --- | --- | --- | --- |
| L.SMLIR.A | N | 16.79 | 618.3523 | 1003.3 | 619.9800 | 1 | 54.06 | 1684 | 1 | 18 | 22 |  |
| R.SNGIT.F | Y | 16.75 | 490.2387 | 2129.6 | 492.2900 | 1 | 66.67 | 2116 | 1 | 157 | 161 |  |
| total 2 peptides |  |  |  |  |  |  |  |  |  |  |  |  |
| --- | --- | --- | --- | --- | --- | --- | --- | --- | --- | --- | --- | --- |

tr|A0A0R6MLS7|A0A0R6MLS7\_9DIPT

back to list

  

| Protein Coverage
| Supporting Peptides
|

Protein Coverage:

Supporting Peptides:

| Peptide | Uniq | -10lgP | Mass | ppm | m/z | z | RT | Scan | #Spec | Start | End | PTM |
| --- | --- | --- | --- | --- | --- | --- | --- | --- | --- | --- | --- | --- |
| L.SMLIR.A | N | 16.79 | 618.3523 | 1003.3 | 619.9800 | 1 | 54.06 | 1684 | 1 | 18 | 22 |  |
| R.SNGIT.F | Y | 16.75 | 490.2387 | 2129.6 | 492.2900 | 1 | 66.67 | 2116 | 1 | 157 | 161 |  |
| total 2 peptides |  |  |  |  |  |  |  |  |  |  |  |  |
| --- | --- | --- | --- | --- | --- | --- | --- | --- | --- | --- | --- | --- |

tr|A0A0R6MLM6|A0A0R6MLM6\_9DIPT

back to list

  

| Protein Coverage
| Supporting Peptides
|

Protein Coverage:

Supporting Peptides:

| Peptide | Uniq | -10lgP | Mass | ppm | m/z | z | RT | Scan | #Spec | Start | End | PTM |
| --- | --- | --- | --- | --- | --- | --- | --- | --- | --- | --- | --- | --- |
| L.SMLIR.A | N | 16.79 | 618.3523 | 1003.3 | 619.9800 | 1 | 54.06 | 1684 | 1 | 18 | 22 |  |
| R.SNGIT.F | Y | 16.75 | 490.2387 | 2129.6 | 492.2900 | 1 | 66.67 | 2116 | 1 | 157 | 161 |  |
| total 2 peptides |  |  |  |  |  |  |  |  |  |  |  |  |
| --- | --- | --- | --- | --- | --- | --- | --- | --- | --- | --- | --- | --- |

tr|A0A0R6MNC0|A0A0R6MNC0\_9DIPT

back to list

  

| Protein Coverage
| Supporting Peptides
|

Protein Coverage:

Supporting Peptides:

| Peptide | Uniq | -10lgP | Mass | ppm | m/z | z | RT | Scan | #Spec | Start | End | PTM |
| --- | --- | --- | --- | --- | --- | --- | --- | --- | --- | --- | --- | --- |
| L.SMLIR.A | N | 16.79 | 618.3523 | 1003.3 | 619.9800 | 1 | 54.06 | 1684 | 1 | 18 | 22 |  |
| R.SNGIT.F | Y | 16.75 | 490.2387 | 2129.6 | 492.2900 | 1 | 66.67 | 2116 | 1 | 157 | 161 |  |
| total 2 peptides |  |  |  |  |  |  |  |  |  |  |  |  |
| --- | --- | --- | --- | --- | --- | --- | --- | --- | --- | --- | --- | --- |

tr|A0A0R6MLV9|A0A0R6MLV9\_9DIPT

back to list

  

| Protein Coverage
| Supporting Peptides
|

Protein Coverage:

Supporting Peptides:

| Peptide | Uniq | -10lgP | Mass | ppm | m/z | z | RT | Scan | #Spec | Start | End | PTM |
| --- | --- | --- | --- | --- | --- | --- | --- | --- | --- | --- | --- | --- |
| L.SMLIR.A | N | 16.79 | 618.3523 | 1003.3 | 619.9800 | 1 | 54.06 | 1684 | 1 | 18 | 22 |  |
| R.SNGIT.F | Y | 16.75 | 490.2387 | 2129.6 | 492.2900 | 1 | 66.67 | 2116 | 1 | 157 | 161 |  |
| total 2 peptides |  |  |  |  |  |  |  |  |  |  |  |  |
| --- | --- | --- | --- | --- | --- | --- | --- | --- | --- | --- | --- | --- |

tr|A0A0P0BAK3|A0A0P0BAK3\_9DIPT

back to list

  

| Protein Coverage
| Supporting Peptides
|

Protein Coverage:

Supporting Peptides:

| Peptide | Uniq | -10lgP | Mass | ppm | m/z | z | RT | Scan | #Spec | Start | End | PTM |
| --- | --- | --- | --- | --- | --- | --- | --- | --- | --- | --- | --- | --- |
| L.SMLIR.A | N | 16.79 | 618.3523 | 1003.3 | 619.9800 | 1 | 54.06 | 1684 | 1 | 18 | 22 |  |
| R.SNGIT.F | Y | 16.75 | 490.2387 | 2129.6 | 492.2900 | 1 | 66.67 | 2116 | 1 | 157 | 161 |  |
| total 2 peptides |  |  |  |  |  |  |  |  |  |  |  |  |
| --- | --- | --- | --- | --- | --- | --- | --- | --- | --- | --- | --- | --- |

tr|A0A0R6MME0|A0A0R6MME0\_9DIPT

back to list

  

| Protein Coverage
| Supporting Peptides
|

Protein Coverage:

Supporting Peptides:

| Peptide | Uniq | -10lgP | Mass | ppm | m/z | z | RT | Scan | #Spec | Start | End | PTM |
| --- | --- | --- | --- | --- | --- | --- | --- | --- | --- | --- | --- | --- |
| L.SMLIR.A | N | 16.79 | 618.3523 | 1003.3 | 619.9800 | 1 | 54.06 | 1684 | 1 | 18 | 22 |  |
| R.SNGIT.F | Y | 16.75 | 490.2387 | 2129.6 | 492.2900 | 1 | 66.67 | 2116 | 1 | 157 | 161 |  |
| total 2 peptides |  |  |  |  |  |  |  |  |  |  |  |  |
| --- | --- | --- | --- | --- | --- | --- | --- | --- | --- | --- | --- | --- |

tr|A0A0R6MJ34|A0A0R6MJ34\_9DIPT

back to list

  

| Protein Coverage
| Supporting Peptides
|

Protein Coverage:

Supporting Peptides:

| Peptide | Uniq | -10lgP | Mass | ppm | m/z | z | RT | Scan | #Spec | Start | End | PTM |
| --- | --- | --- | --- | --- | --- | --- | --- | --- | --- | --- | --- | --- |
| L.SMLIR.A | N | 16.79 | 618.3523 | 1003.3 | 619.9800 | 1 | 54.06 | 1684 | 1 | 18 | 22 |  |
| R.SNGIT.F | Y | 16.75 | 490.2387 | 2129.6 | 492.2900 | 1 | 66.67 | 2116 | 1 | 157 | 161 |  |
| total 2 peptides |  |  |  |  |  |  |  |  |  |  |  |  |
| --- | --- | --- | --- | --- | --- | --- | --- | --- | --- | --- | --- | --- |

tr|A0A0R6MLG8|A0A0R6MLG8\_9DIPT

back to list

  

| Protein Coverage
| Supporting Peptides
|

Protein Coverage:

Supporting Peptides:

| Peptide | Uniq | -10lgP | Mass | ppm | m/z | z | RT | Scan | #Spec | Start | End | PTM |
| --- | --- | --- | --- | --- | --- | --- | --- | --- | --- | --- | --- | --- |
| L.SMLIR.A | N | 16.79 | 618.3523 | 1003.3 | 619.9800 | 1 | 54.06 | 1684 | 1 | 18 | 22 |  |
| R.SNGIT.F | Y | 16.75 | 490.2387 | 2129.6 | 492.2900 | 1 | 66.67 | 2116 | 1 | 157 | 161 |  |
| total 2 peptides |  |  |  |  |  |  |  |  |  |  |  |  |
| --- | --- | --- | --- | --- | --- | --- | --- | --- | --- | --- | --- | --- |

tr|A0A0R6MMU3|A0A0R6MMU3\_9DIPT

back to list

  

| Protein Coverage
| Supporting Peptides
|

Protein Coverage:

Supporting Peptides:

| Peptide | Uniq | -10lgP | Mass | ppm | m/z | z | RT | Scan | #Spec | Start | End | PTM |
| --- | --- | --- | --- | --- | --- | --- | --- | --- | --- | --- | --- | --- |
| L.SMLIR.A | N | 16.79 | 618.3523 | 1003.3 | 619.9800 | 1 | 54.06 | 1684 | 1 | 18 | 22 |  |
| R.SNGIT.F | Y | 16.75 | 490.2387 | 2129.6 | 492.2900 | 1 | 66.67 | 2116 | 1 | 157 | 161 |  |
| total 2 peptides |  |  |  |  |  |  |  |  |  |  |  |  |
| --- | --- | --- | --- | --- | --- | --- | --- | --- | --- | --- | --- | --- |

tr|A0A0R6ML78|A0A0R6ML78\_9DIPT

back to list

  

| Protein Coverage
| Supporting Peptides
|

Protein Coverage:

Supporting Peptides:

| Peptide | Uniq | -10lgP | Mass | ppm | m/z | z | RT | Scan | #Spec | Start | End | PTM |
| --- | --- | --- | --- | --- | --- | --- | --- | --- | --- | --- | --- | --- |
| L.SMLIR.A | N | 16.79 | 618.3523 | 1003.3 | 619.9800 | 1 | 54.06 | 1684 | 1 | 18 | 22 |  |
| R.SNGIT.F | Y | 16.75 | 490.2387 | 2129.6 | 492.2900 | 1 | 66.67 | 2116 | 1 | 157 | 161 |  |
| total 2 peptides |  |  |  |  |  |  |  |  |  |  |  |  |
| --- | --- | --- | --- | --- | --- | --- | --- | --- | --- | --- | --- | --- |

tr|A0A0R6MMJ5|A0A0R6MMJ5\_9DIPT

back to list

  

| Protein Coverage
| Supporting Peptides
|

Protein Coverage:

Supporting Peptides:

| Peptide | Uniq | -10lgP | Mass | ppm | m/z | z | RT | Scan | #Spec | Start | End | PTM |
| --- | --- | --- | --- | --- | --- | --- | --- | --- | --- | --- | --- | --- |
| L.SMLIR.A | N | 16.79 | 618.3523 | 1003.3 | 619.9800 | 1 | 54.06 | 1684 | 1 | 18 | 22 |  |
| R.SNGIT.F | Y | 16.75 | 490.2387 | 2129.6 | 492.2900 | 1 | 66.67 | 2116 | 1 | 157 | 161 |  |
| total 2 peptides |  |  |  |  |  |  |  |  |  |  |  |  |
| --- | --- | --- | --- | --- | --- | --- | --- | --- | --- | --- | --- | --- |

tr|A0A0R6MIV4|A0A0R6MIV4\_9DIPT

back to list

  

| Protein Coverage
| Supporting Peptides
|

Protein Coverage:

Supporting Peptides:

| Peptide | Uniq | -10lgP | Mass | ppm | m/z | z | RT | Scan | #Spec | Start | End | PTM |
| --- | --- | --- | --- | --- | --- | --- | --- | --- | --- | --- | --- | --- |
| L.SMLIR.A | N | 16.79 | 618.3523 | 1003.3 | 619.9800 | 1 | 54.06 | 1684 | 1 | 18 | 22 |  |
| R.SNGIT.F | Y | 16.75 | 490.2387 | 2129.6 | 492.2900 | 1 | 66.67 | 2116 | 1 | 157 | 161 |  |
| total 2 peptides |  |  |  |  |  |  |  |  |  |  |  |  |
| --- | --- | --- | --- | --- | --- | --- | --- | --- | --- | --- | --- | --- |

tr|A0A0R6MMS1|A0A0R6MMS1\_9DIPT

back to list

  

| Protein Coverage
| Supporting Peptides
|

Protein Coverage:

Supporting Peptides:

| Peptide | Uniq | -10lgP | Mass | ppm | m/z | z | RT | Scan | #Spec | Start | End | PTM |
| --- | --- | --- | --- | --- | --- | --- | --- | --- | --- | --- | --- | --- |
| L.SMLIR.A | N | 16.79 | 618.3523 | 1003.3 | 619.9800 | 1 | 54.06 | 1684 | 1 | 18 | 22 |  |
| R.SNGIT.F | Y | 16.75 | 490.2387 | 2129.6 | 492.2900 | 1 | 66.67 | 2116 | 1 | 157 | 161 |  |
| total 2 peptides |  |  |  |  |  |  |  |  |  |  |  |  |
| --- | --- | --- | --- | --- | --- | --- | --- | --- | --- | --- | --- | --- |

tr|A0A0R6MM62|A0A0R6MM62\_9DIPT

back to list

  

| Protein Coverage
| Supporting Peptides
|

Protein Coverage:

Supporting Peptides:

| Peptide | Uniq | -10lgP | Mass | ppm | m/z | z | RT | Scan | #Spec | Start | End | PTM |
| --- | --- | --- | --- | --- | --- | --- | --- | --- | --- | --- | --- | --- |
| L.SMLIR.A | N | 16.79 | 618.3523 | 1003.3 | 619.9800 | 1 | 54.06 | 1684 | 1 | 18 | 22 |  |
| R.SNGIT.F | Y | 16.75 | 490.2387 | 2129.6 | 492.2900 | 1 | 66.67 | 2116 | 1 | 157 | 161 |  |
| total 2 peptides |  |  |  |  |  |  |  |  |  |  |  |  |
| --- | --- | --- | --- | --- | --- | --- | --- | --- | --- | --- | --- | --- |

tr|A0A0C4KJ04|A0A0C4KJ04\_SIMVI

back to list

  

| Protein Coverage
| Supporting Peptides
|

Protein Coverage:

Supporting Peptides:

| Peptide | Uniq | -10lgP | Mass | ppm | m/z | z | RT | Scan | #Spec | Start | End | PTM |
| --- | --- | --- | --- | --- | --- | --- | --- | --- | --- | --- | --- | --- |
| L.SMLIR.A | N | 16.79 | 618.3523 | 1003.3 | 619.9800 | 1 | 54.06 | 1684 | 1 | 10 | 14 |  |
| R.SNGIT.F | Y | 16.75 | 490.2387 | 2129.6 | 492.2900 | 1 | 66.67 | 2116 | 1 | 149 | 153 |  |
| total 2 peptides |  |  |  |  |  |  |  |  |  |  |  |  |
| --- | --- | --- | --- | --- | --- | --- | --- | --- | --- | --- | --- | --- |

tr|A0A0C4KVS0|A0A0C4KVS0\_9DIPT

back to list

  

| Protein Coverage
| Supporting Peptides
|

Protein Coverage:

Supporting Peptides:

| Peptide | Uniq | -10lgP | Mass | ppm | m/z | z | RT | Scan | #Spec | Start | End | PTM |
| --- | --- | --- | --- | --- | --- | --- | --- | --- | --- | --- | --- | --- |
| L.SMLIR.A | N | 16.79 | 618.3523 | 1003.3 | 619.9800 | 1 | 54.06 | 1684 | 1 | 14 | 18 |  |
| R.SNGIT.F | Y | 16.75 | 490.2387 | 2129.6 | 492.2900 | 1 | 66.67 | 2116 | 1 | 153 | 157 |  |
| total 2 peptides |  |  |  |  |  |  |  |  |  |  |  |  |
| --- | --- | --- | --- | --- | --- | --- | --- | --- | --- | --- | --- | --- |

tr|A0A0C4KJZ7|A0A0C4KJZ7\_9DIPT

back to list

  

| Protein Coverage
| Supporting Peptides
|

Protein Coverage:

Supporting Peptides:

| Peptide | Uniq | -10lgP | Mass | ppm | m/z | z | RT | Scan | #Spec | Start | End | PTM |
| --- | --- | --- | --- | --- | --- | --- | --- | --- | --- | --- | --- | --- |
| L.SMLIR.A | N | 16.79 | 618.3523 | 1003.3 | 619.9800 | 1 | 54.06 | 1684 | 1 | 13 | 17 |  |
| R.SNGIT.F | Y | 16.75 | 490.2387 | 2129.6 | 492.2900 | 1 | 66.67 | 2116 | 1 | 152 | 156 |  |
| total 2 peptides |  |  |  |  |  |  |  |  |  |  |  |  |
| --- | --- | --- | --- | --- | --- | --- | --- | --- | --- | --- | --- | --- |

tr|A0A0C4KJV3|A0A0C4KJV3\_9DIPT

back to list

  

| Protein Coverage
| Supporting Peptides
|

Protein Coverage:

Supporting Peptides:

| Peptide | Uniq | -10lgP | Mass | ppm | m/z | z | RT | Scan | #Spec | Start | End | PTM |
| --- | --- | --- | --- | --- | --- | --- | --- | --- | --- | --- | --- | --- |
| L.SMLIR.A | N | 16.79 | 618.3523 | 1003.3 | 619.9800 | 1 | 54.06 | 1684 | 1 | 8 | 12 |  |
| R.SNGIT.F | Y | 16.75 | 490.2387 | 2129.6 | 492.2900 | 1 | 66.67 | 2116 | 1 | 147 | 151 |  |
| total 2 peptides |  |  |  |  |  |  |  |  |  |  |  |  |
| --- | --- | --- | --- | --- | --- | --- | --- | --- | --- | --- | --- | --- |

tr|A0A0C4KQW3|A0A0C4KQW3\_SIMVI

back to list

  

| Protein Coverage
| Supporting Peptides
|

Protein Coverage:

Supporting Peptides:

| Peptide | Uniq | -10lgP | Mass | ppm | m/z | z | RT | Scan | #Spec | Start | End | PTM |
| --- | --- | --- | --- | --- | --- | --- | --- | --- | --- | --- | --- | --- |
| L.SMLIR.A | N | 16.79 | 618.3523 | 1003.3 | 619.9800 | 1 | 54.06 | 1684 | 1 | 10 | 14 |  |
| R.SNGIT.F | Y | 16.75 | 490.2387 | 2129.6 | 492.2900 | 1 | 66.67 | 2116 | 1 | 149 | 153 |  |
| total 2 peptides |  |  |  |  |  |  |  |  |  |  |  |  |
| --- | --- | --- | --- | --- | --- | --- | --- | --- | --- | --- | --- | --- |

tr|A0A0C4KUB8|A0A0C4KUB8\_9DIPT

back to list

  

| Protein Coverage
| Supporting Peptides
|

Protein Coverage:

Supporting Peptides:

| Peptide | Uniq | -10lgP | Mass | ppm | m/z | z | RT | Scan | #Spec | Start | End | PTM |
| --- | --- | --- | --- | --- | --- | --- | --- | --- | --- | --- | --- | --- |
| L.SMLIR.A | N | 16.79 | 618.3523 | 1003.3 | 619.9800 | 1 | 54.06 | 1684 | 1 | 13 | 17 |  |
| R.SNGIT.F | Y | 16.75 | 490.2387 | 2129.6 | 492.2900 | 1 | 66.67 | 2116 | 1 | 152 | 156 |  |
| total 2 peptides |  |  |  |  |  |  |  |  |  |  |  |  |
| --- | --- | --- | --- | --- | --- | --- | --- | --- | --- | --- | --- | --- |

tr|A0A0C4KUJ2|A0A0C4KUJ2\_9DIPT

back to list

  

| Protein Coverage
| Supporting Peptides
|

Protein Coverage:

Supporting Peptides:

| Peptide | Uniq | -10lgP | Mass | ppm | m/z | z | RT | Scan | #Spec | Start | End | PTM |
| --- | --- | --- | --- | --- | --- | --- | --- | --- | --- | --- | --- | --- |
| L.SMLIR.A | N | 16.79 | 618.3523 | 1003.3 | 619.9800 | 1 | 54.06 | 1684 | 1 | 17 | 21 |  |
| R.SNGIT.F | Y | 16.75 | 490.2387 | 2129.6 | 492.2900 | 1 | 66.67 | 2116 | 1 | 156 | 160 |  |
| total 2 peptides |  |  |  |  |  |  |  |  |  |  |  |  |
| --- | --- | --- | --- | --- | --- | --- | --- | --- | --- | --- | --- | --- |

tr|A0A0C4KU92|A0A0C4KU92\_9DIPT

back to list

  

| Protein Coverage
| Supporting Peptides
|

Protein Coverage:

Supporting Peptides:

| Peptide | Uniq | -10lgP | Mass | ppm | m/z | z | RT | Scan | #Spec | Start | End | PTM |
| --- | --- | --- | --- | --- | --- | --- | --- | --- | --- | --- | --- | --- |
| L.SMLIR.A | N | 16.79 | 618.3523 | 1003.3 | 619.9800 | 1 | 54.06 | 1684 | 1 | 18 | 22 |  |
| R.SNGIT.F | Y | 16.75 | 490.2387 | 2129.6 | 492.2900 | 1 | 66.67 | 2116 | 1 | 157 | 161 |  |
| total 2 peptides |  |  |  |  |  |  |  |  |  |  |  |  |
| --- | --- | --- | --- | --- | --- | --- | --- | --- | --- | --- | --- | --- |

tr|A0A0C4KVR6|A0A0C4KVR6\_9DIPT

back to list

  

| Protein Coverage
| Supporting Peptides
|

Protein Coverage:

Supporting Peptides:

| Peptide | Uniq | -10lgP | Mass | ppm | m/z | z | RT | Scan | #Spec | Start | End | PTM |
| --- | --- | --- | --- | --- | --- | --- | --- | --- | --- | --- | --- | --- |
| L.SMLIR.A | N | 16.79 | 618.3523 | 1003.3 | 619.9800 | 1 | 54.06 | 1684 | 1 | 14 | 18 |  |
| R.SNGIT.F | Y | 16.75 | 490.2387 | 2129.6 | 492.2900 | 1 | 66.67 | 2116 | 1 | 153 | 157 |  |
| total 2 peptides |  |  |  |  |  |  |  |  |  |  |  |  |
| --- | --- | --- | --- | --- | --- | --- | --- | --- | --- | --- | --- | --- |

tr|A0A068FG89|A0A068FG89\_9DIPT

back to list

  

| Protein Coverage
| Supporting Peptides
|

Protein Coverage:

Supporting Peptides:

| Peptide | Uniq | -10lgP | Mass | ppm | m/z | z | RT | Scan | #Spec | Start | End | PTM |
| --- | --- | --- | --- | --- | --- | --- | --- | --- | --- | --- | --- | --- |
| L.SMLIR.A | N | 16.79 | 618.3523 | 1003.3 | 619.9800 | 1 | 54.06 | 1684 | 1 | 12 | 16 |  |
| R.SNGIT.F | Y | 16.75 | 490.2387 | 2129.6 | 492.2900 | 1 | 66.67 | 2116 | 1 | 151 | 155 |  |
| total 2 peptides |  |  |  |  |  |  |  |  |  |  |  |  |
| --- | --- | --- | --- | --- | --- | --- | --- | --- | --- | --- | --- | --- |

tr|A0A068FF14|A0A068FF14\_9DIPT

back to list

  

| Protein Coverage
| Supporting Peptides
|

Protein Coverage:

Supporting Peptides:

| Peptide | Uniq | -10lgP | Mass | ppm | m/z | z | RT | Scan | #Spec | Start | End | PTM |
| --- | --- | --- | --- | --- | --- | --- | --- | --- | --- | --- | --- | --- |
| L.SMLIR.A | N | 16.79 | 618.3523 | 1003.3 | 619.9800 | 1 | 54.06 | 1684 | 1 | 12 | 16 |  |
| R.SNGIT.F | Y | 16.75 | 490.2387 | 2129.6 | 492.2900 | 1 | 66.67 | 2116 | 1 | 151 | 155 |  |
| total 2 peptides |  |  |  |  |  |  |  |  |  |  |  |  |
| --- | --- | --- | --- | --- | --- | --- | --- | --- | --- | --- | --- | --- |

tr|A0A068FF33|A0A068FF33\_9DIPT

back to list

  

| Protein Coverage
| Supporting Peptides
|

Protein Coverage:

Supporting Peptides:

| Peptide | Uniq | -10lgP | Mass | ppm | m/z | z | RT | Scan | #Spec | Start | End | PTM |
| --- | --- | --- | --- | --- | --- | --- | --- | --- | --- | --- | --- | --- |
| L.SMLIR.A | N | 16.79 | 618.3523 | 1003.3 | 619.9800 | 1 | 54.06 | 1684 | 1 | 12 | 16 |  |
| R.SNGIT.F | Y | 16.75 | 490.2387 | 2129.6 | 492.2900 | 1 | 66.67 | 2116 | 1 | 151 | 155 |  |
| total 2 peptides |  |  |  |  |  |  |  |  |  |  |  |  |
| --- | --- | --- | --- | --- | --- | --- | --- | --- | --- | --- | --- | --- |

tr|A0A068FGA3|A0A068FGA3\_9DIPT

back to list

  

| Protein Coverage
| Supporting Peptides
|

Protein Coverage:

Supporting Peptides:

| Peptide | Uniq | -10lgP | Mass | ppm | m/z | z | RT | Scan | #Spec | Start | End | PTM |
| --- | --- | --- | --- | --- | --- | --- | --- | --- | --- | --- | --- | --- |
| L.SMLIR.A | N | 16.79 | 618.3523 | 1003.3 | 619.9800 | 1 | 54.06 | 1684 | 1 | 12 | 16 |  |
| R.SNGIT.F | Y | 16.75 | 490.2387 | 2129.6 | 492.2900 | 1 | 66.67 | 2116 | 1 | 151 | 155 |  |
| total 2 peptides |  |  |  |  |  |  |  |  |  |  |  |  |
| --- | --- | --- | --- | --- | --- | --- | --- | --- | --- | --- | --- | --- |

tr|A0A068FF22|A0A068FF22\_9DIPT

back to list

  

| Protein Coverage
| Supporting Peptides
|

Protein Coverage:

Supporting Peptides:

| Peptide | Uniq | -10lgP | Mass | ppm | m/z | z | RT | Scan | #Spec | Start | End | PTM |
| --- | --- | --- | --- | --- | --- | --- | --- | --- | --- | --- | --- | --- |
| L.SMLIR.A | N | 16.79 | 618.3523 | 1003.3 | 619.9800 | 1 | 54.06 | 1684 | 1 | 12 | 16 |  |
| R.SNGIT.F | Y | 16.75 | 490.2387 | 2129.6 | 492.2900 | 1 | 66.67 | 2116 | 1 | 151 | 155 |  |
| total 2 peptides |  |  |  |  |  |  |  |  |  |  |  |  |
| --- | --- | --- | --- | --- | --- | --- | --- | --- | --- | --- | --- | --- |

tr|A0A068FE44|A0A068FE44\_9DIPT

back to list

  

| Protein Coverage
| Supporting Peptides
|

Protein Coverage:

Supporting Peptides:

| Peptide | Uniq | -10lgP | Mass | ppm | m/z | z | RT | Scan | #Spec | Start | End | PTM |
| --- | --- | --- | --- | --- | --- | --- | --- | --- | --- | --- | --- | --- |
| L.SMLIR.A | N | 16.79 | 618.3523 | 1003.3 | 619.9800 | 1 | 54.06 | 1684 | 1 | 12 | 16 |  |
| R.SNGIT.F | Y | 16.75 | 490.2387 | 2129.6 | 492.2900 | 1 | 66.67 | 2116 | 1 | 151 | 155 |  |
| total 2 peptides |  |  |  |  |  |  |  |  |  |  |  |  |
| --- | --- | --- | --- | --- | --- | --- | --- | --- | --- | --- | --- | --- |

tr|A0A068FF02|A0A068FF02\_9DIPT

back to list

  

| Protein Coverage
| Supporting Peptides
|

Protein Coverage:

Supporting Peptides:

| Peptide | Uniq | -10lgP | Mass | ppm | m/z | z | RT | Scan | #Spec | Start | End | PTM |
| --- | --- | --- | --- | --- | --- | --- | --- | --- | --- | --- | --- | --- |
| L.SMLIR.A | N | 16.79 | 618.3523 | 1003.3 | 619.9800 | 1 | 54.06 | 1684 | 1 | 12 | 16 |  |
| R.SNGIT.F | Y | 16.75 | 490.2387 | 2129.6 | 492.2900 | 1 | 66.67 | 2116 | 1 | 151 | 155 |  |
| total 2 peptides |  |  |  |  |  |  |  |  |  |  |  |  |
| --- | --- | --- | --- | --- | --- | --- | --- | --- | --- | --- | --- | --- |

tr|A0A068FE49|A0A068FE49\_9DIPT

back to list

  

| Protein Coverage
| Supporting Peptides
|

Protein Coverage:

Supporting Peptides:

| Peptide | Uniq | -10lgP | Mass | ppm | m/z | z | RT | Scan | #Spec | Start | End | PTM |
| --- | --- | --- | --- | --- | --- | --- | --- | --- | --- | --- | --- | --- |
| L.SMLIR.A | N | 16.79 | 618.3523 | 1003.3 | 619.9800 | 1 | 54.06 | 1684 | 1 | 12 | 16 |  |
| R.SNGIT.F | Y | 16.75 | 490.2387 | 2129.6 | 492.2900 | 1 | 66.67 | 2116 | 1 | 151 | 155 |  |
| total 2 peptides |  |  |  |  |  |  |  |  |  |  |  |  |
| --- | --- | --- | --- | --- | --- | --- | --- | --- | --- | --- | --- | --- |

tr|A0A068FF29|A0A068FF29\_9DIPT

back to list

  

| Protein Coverage
| Supporting Peptides
|

Protein Coverage:

Supporting Peptides:

| Peptide | Uniq | -10lgP | Mass | ppm | m/z | z | RT | Scan | #Spec | Start | End | PTM |
| --- | --- | --- | --- | --- | --- | --- | --- | --- | --- | --- | --- | --- |
| L.SMLIR.A | N | 16.79 | 618.3523 | 1003.3 | 619.9800 | 1 | 54.06 | 1684 | 1 | 12 | 16 |  |
| R.SNGIT.F | Y | 16.75 | 490.2387 | 2129.6 | 492.2900 | 1 | 66.67 | 2116 | 1 | 151 | 155 |  |
| total 2 peptides |  |  |  |  |  |  |  |  |  |  |  |  |
| --- | --- | --- | --- | --- | --- | --- | --- | --- | --- | --- | --- | --- |

tr|A0A068FJZ5|A0A068FJZ5\_9DIPT

back to list

  

| Protein Coverage
| Supporting Peptides
|

Protein Coverage:

Supporting Peptides:

| Peptide | Uniq | -10lgP | Mass | ppm | m/z | z | RT | Scan | #Spec | Start | End | PTM |
| --- | --- | --- | --- | --- | --- | --- | --- | --- | --- | --- | --- | --- |
| L.SMLIR.A | N | 16.79 | 618.3523 | 1003.3 | 619.9800 | 1 | 54.06 | 1684 | 1 | 12 | 16 |  |
| R.SNGIT.F | Y | 16.75 | 490.2387 | 2129.6 | 492.2900 | 1 | 66.67 | 2116 | 1 | 151 | 155 |  |
| total 2 peptides |  |  |  |  |  |  |  |  |  |  |  |  |
| --- | --- | --- | --- | --- | --- | --- | --- | --- | --- | --- | --- | --- |

tr|A0A068FGB4|A0A068FGB4\_9DIPT

back to list

  

| Protein Coverage
| Supporting Peptides
|

Protein Coverage:

Supporting Peptides:

| Peptide | Uniq | -10lgP | Mass | ppm | m/z | z | RT | Scan | #Spec | Start | End | PTM |
| --- | --- | --- | --- | --- | --- | --- | --- | --- | --- | --- | --- | --- |
| L.SMLIR.A | N | 16.79 | 618.3523 | 1003.3 | 619.9800 | 1 | 54.06 | 1684 | 1 | 12 | 16 |  |
| R.SNGIT.F | Y | 16.75 | 490.2387 | 2129.6 | 492.2900 | 1 | 66.67 | 2116 | 1 | 151 | 155 |  |
| total 2 peptides |  |  |  |  |  |  |  |  |  |  |  |  |
| --- | --- | --- | --- | --- | --- | --- | --- | --- | --- | --- | --- | --- |

tr|A0A068FF18|A0A068FF18\_9DIPT

back to list

  

| Protein Coverage
| Supporting Peptides
|

Protein Coverage:

Supporting Peptides:

| Peptide | Uniq | -10lgP | Mass | ppm | m/z | z | RT | Scan | #Spec | Start | End | PTM |
| --- | --- | --- | --- | --- | --- | --- | --- | --- | --- | --- | --- | --- |
| L.SMLIR.A | N | 16.79 | 618.3523 | 1003.3 | 619.9800 | 1 | 54.06 | 1684 | 1 | 12 | 16 |  |
| R.SNGIT.F | Y | 16.75 | 490.2387 | 2129.6 | 492.2900 | 1 | 66.67 | 2116 | 1 | 151 | 155 |  |
| total 2 peptides |  |  |  |  |  |  |  |  |  |  |  |  |
| --- | --- | --- | --- | --- | --- | --- | --- | --- | --- | --- | --- | --- |

tr|A0A0H3VZF8|A0A0H3VZF8\_9DIPT

back to list

  

| Protein Coverage
| Supporting Peptides
|

Protein Coverage:

Supporting Peptides:

| Peptide | Uniq | -10lgP | Mass | ppm | m/z | z | RT | Scan | #Spec | Start | End | PTM |
| --- | --- | --- | --- | --- | --- | --- | --- | --- | --- | --- | --- | --- |
| L.SMLIR.A | N | 16.79 | 618.3523 | 1003.3 | 619.9800 | 1 | 54.06 | 1684 | 1 | 12 | 16 |  |
| R.SNGIT.F | Y | 16.75 | 490.2387 | 2129.6 | 492.2900 | 1 | 66.67 | 2116 | 1 | 151 | 155 |  |
| total 2 peptides |  |  |  |  |  |  |  |  |  |  |  |  |
| --- | --- | --- | --- | --- | --- | --- | --- | --- | --- | --- | --- | --- |

tr|A0A068FE25|A0A068FE25\_9DIPT

back to list

  

| Protein Coverage
| Supporting Peptides
|

Protein Coverage:

Supporting Peptides:

| Peptide | Uniq | -10lgP | Mass | ppm | m/z | z | RT | Scan | #Spec | Start | End | PTM |
| --- | --- | --- | --- | --- | --- | --- | --- | --- | --- | --- | --- | --- |
| L.SMLIR.A | N | 16.79 | 618.3523 | 1003.3 | 619.9800 | 1 | 54.06 | 1684 | 1 | 12 | 16 |  |
| R.SNGIT.F | Y | 16.75 | 490.2387 | 2129.6 | 492.2900 | 1 | 66.67 | 2116 | 1 | 151 | 155 |  |
| total 2 peptides |  |  |  |  |  |  |  |  |  |  |  |  |
| --- | --- | --- | --- | --- | --- | --- | --- | --- | --- | --- | --- | --- |

tr|A0A068FK01|A0A068FK01\_9DIPT

back to list

  

| Protein Coverage
| Supporting Peptides
|

Protein Coverage:

Supporting Peptides:

| Peptide | Uniq | -10lgP | Mass | ppm | m/z | z | RT | Scan | #Spec | Start | End | PTM |
| --- | --- | --- | --- | --- | --- | --- | --- | --- | --- | --- | --- | --- |
| L.SMLIR.A | N | 16.79 | 618.3523 | 1003.3 | 619.9800 | 1 | 54.06 | 1684 | 1 | 12 | 16 |  |
| R.SNGIT.F | Y | 16.75 | 490.2387 | 2129.6 | 492.2900 | 1 | 66.67 | 2116 | 1 | 151 | 155 |  |
| total 2 peptides |  |  |  |  |  |  |  |  |  |  |  |  |
| --- | --- | --- | --- | --- | --- | --- | --- | --- | --- | --- | --- | --- |

tr|A0A068FG81|A0A068FG81\_9DIPT

back to list

  

| Protein Coverage
| Supporting Peptides
|

Protein Coverage:

Supporting Peptides:

| Peptide | Uniq | -10lgP | Mass | ppm | m/z | z | RT | Scan | #Spec | Start | End | PTM |
| --- | --- | --- | --- | --- | --- | --- | --- | --- | --- | --- | --- | --- |
| L.SMLIR.A | N | 16.79 | 618.3523 | 1003.3 | 619.9800 | 1 | 54.06 | 1684 | 1 | 12 | 16 |  |
| R.SNGIT.F | Y | 16.75 | 490.2387 | 2129.6 | 492.2900 | 1 | 66.67 | 2116 | 1 | 151 | 155 |  |
| total 2 peptides |  |  |  |  |  |  |  |  |  |  |  |  |
| --- | --- | --- | --- | --- | --- | --- | --- | --- | --- | --- | --- | --- |

tr|A0A068FEX8|A0A068FEX8\_9DIPT

back to list

  

| Protein Coverage
| Supporting Peptides
|

Protein Coverage:

Supporting Peptides:

| Peptide | Uniq | -10lgP | Mass | ppm | m/z | z | RT | Scan | #Spec | Start | End | PTM |
| --- | --- | --- | --- | --- | --- | --- | --- | --- | --- | --- | --- | --- |
| L.SMLIR.A | N | 16.79 | 618.3523 | 1003.3 | 619.9800 | 1 | 54.06 | 1684 | 1 | 12 | 16 |  |
| R.SNGIT.F | Y | 16.75 | 490.2387 | 2129.6 | 492.2900 | 1 | 66.67 | 2116 | 1 | 151 | 155 |  |
| total 2 peptides |  |  |  |  |  |  |  |  |  |  |  |  |
| --- | --- | --- | --- | --- | --- | --- | --- | --- | --- | --- | --- | --- |

tr|A0A068FE32|A0A068FE32\_9DIPT

back to list

  

| Protein Coverage
| Supporting Peptides
|

Protein Coverage:

Supporting Peptides:

| Peptide | Uniq | -10lgP | Mass | ppm | m/z | z | RT | Scan | #Spec | Start | End | PTM |
| --- | --- | --- | --- | --- | --- | --- | --- | --- | --- | --- | --- | --- |
| L.SMLIR.A | N | 16.79 | 618.3523 | 1003.3 | 619.9800 | 1 | 54.06 | 1684 | 1 | 12 | 16 |  |
| R.SNGIT.F | Y | 16.75 | 490.2387 | 2129.6 | 492.2900 | 1 | 66.67 | 2116 | 1 | 151 | 155 |  |
| total 2 peptides |  |  |  |  |  |  |  |  |  |  |  |  |
| --- | --- | --- | --- | --- | --- | --- | --- | --- | --- | --- | --- | --- |

tr|A0A068FDB5|A0A068FDB5\_9DIPT

back to list

  

| Protein Coverage
| Supporting Peptides
|

Protein Coverage:

Supporting Peptides:

| Peptide | Uniq | -10lgP | Mass | ppm | m/z | z | RT | Scan | #Spec | Start | End | PTM |
| --- | --- | --- | --- | --- | --- | --- | --- | --- | --- | --- | --- | --- |
| L.SMLIR.A | N | 16.79 | 618.3523 | 1003.3 | 619.9800 | 1 | 54.06 | 1684 | 1 | 12 | 16 |  |
| R.SNGIT.F | Y | 16.75 | 490.2387 | 2129.6 | 492.2900 | 1 | 66.67 | 2116 | 1 | 151 | 155 |  |
| total 2 peptides |  |  |  |  |  |  |  |  |  |  |  |  |
| --- | --- | --- | --- | --- | --- | --- | --- | --- | --- | --- | --- | --- |

tr|A0A068FDB0|A0A068FDB0\_9DIPT

back to list

  

| Protein Coverage
| Supporting Peptides
|

Protein Coverage:

Supporting Peptides:

| Peptide | Uniq | -10lgP | Mass | ppm | m/z | z | RT | Scan | #Spec | Start | End | PTM |
| --- | --- | --- | --- | --- | --- | --- | --- | --- | --- | --- | --- | --- |
| L.SMLIR.A | N | 16.79 | 618.3523 | 1003.3 | 619.9800 | 1 | 54.06 | 1684 | 1 | 12 | 16 |  |
| R.SNGIT.F | Y | 16.75 | 490.2387 | 2129.6 | 492.2900 | 1 | 66.67 | 2116 | 1 | 151 | 155 |  |
| total 2 peptides |  |  |  |  |  |  |  |  |  |  |  |  |
| --- | --- | --- | --- | --- | --- | --- | --- | --- | --- | --- | --- | --- |

tr|A0A068FE41|A0A068FE41\_9DIPT

back to list

  

| Protein Coverage
| Supporting Peptides
|

Protein Coverage:

Supporting Peptides:

| Peptide | Uniq | -10lgP | Mass | ppm | m/z | z | RT | Scan | #Spec | Start | End | PTM |
| --- | --- | --- | --- | --- | --- | --- | --- | --- | --- | --- | --- | --- |
| L.SMLIR.A | N | 16.79 | 618.3523 | 1003.3 | 619.9800 | 1 | 54.06 | 1684 | 1 | 12 | 16 |  |
| R.SNGIT.F | Y | 16.75 | 490.2387 | 2129.6 | 492.2900 | 1 | 66.67 | 2116 | 1 | 151 | 155 |  |
| total 2 peptides |  |  |  |  |  |  |  |  |  |  |  |  |
| --- | --- | --- | --- | --- | --- | --- | --- | --- | --- | --- | --- | --- |

tr|A0A068FE57|A0A068FE57\_9DIPT

back to list

  

| Protein Coverage
| Supporting Peptides
|

Protein Coverage:

Supporting Peptides:

| Peptide | Uniq | -10lgP | Mass | ppm | m/z | z | RT | Scan | #Spec | Start | End | PTM |
| --- | --- | --- | --- | --- | --- | --- | --- | --- | --- | --- | --- | --- |
| L.SMLIR.A | N | 16.79 | 618.3523 | 1003.3 | 619.9800 | 1 | 54.06 | 1684 | 1 | 12 | 16 |  |
| R.SNGIT.F | Y | 16.75 | 490.2387 | 2129.6 | 492.2900 | 1 | 66.67 | 2116 | 1 | 151 | 155 |  |
| total 2 peptides |  |  |  |  |  |  |  |  |  |  |  |  |
| --- | --- | --- | --- | --- | --- | --- | --- | --- | --- | --- | --- | --- |

tr|A0A068FE21|A0A068FE21\_9DIPT

back to list

  

| Protein Coverage
| Supporting Peptides
|

Protein Coverage:

Supporting Peptides:

| Peptide | Uniq | -10lgP | Mass | ppm | m/z | z | RT | Scan | #Spec | Start | End | PTM |
| --- | --- | --- | --- | --- | --- | --- | --- | --- | --- | --- | --- | --- |
| L.SMLIR.A | N | 16.79 | 618.3523 | 1003.3 | 619.9800 | 1 | 54.06 | 1684 | 1 | 12 | 16 |  |
| R.SNGIT.F | Y | 16.75 | 490.2387 | 2129.6 | 492.2900 | 1 | 66.67 | 2116 | 1 | 151 | 155 |  |
| total 2 peptides |  |  |  |  |  |  |  |  |  |  |  |  |
| --- | --- | --- | --- | --- | --- | --- | --- | --- | --- | --- | --- | --- |

tr|A0A068FDC3|A0A068FDC3\_9DIPT

back to list

  

| Protein Coverage
| Supporting Peptides
|

Protein Coverage:

Supporting Peptides:

| Peptide | Uniq | -10lgP | Mass | ppm | m/z | z | RT | Scan | #Spec | Start | End | PTM |
| --- | --- | --- | --- | --- | --- | --- | --- | --- | --- | --- | --- | --- |
| L.SMLIR.A | N | 16.79 | 618.3523 | 1003.3 | 619.9800 | 1 | 54.06 | 1684 | 1 | 5 | 9 |  |
| R.SNGIT.F | Y | 16.75 | 490.2387 | 2129.6 | 492.2900 | 1 | 66.67 | 2116 | 1 | 144 | 148 |  |
| total 2 peptides |  |  |  |  |  |  |  |  |  |  |  |  |
| --- | --- | --- | --- | --- | --- | --- | --- | --- | --- | --- | --- | --- |

tr|A0A068FEH9|A0A068FEH9\_9DIPT

back to list

  

| Protein Coverage
| Supporting Peptides
|

Protein Coverage:

Supporting Peptides:

| Peptide | Uniq | -10lgP | Mass | ppm | m/z | z | RT | Scan | #Spec | Start | End | PTM |
| --- | --- | --- | --- | --- | --- | --- | --- | --- | --- | --- | --- | --- |
| L.SMLIR.A | N | 16.79 | 618.3523 | 1003.3 | 619.9800 | 1 | 54.06 | 1684 | 1 | 12 | 16 |  |
| R.SNGIT.F | Y | 16.75 | 490.2387 | 2129.6 | 492.2900 | 1 | 66.67 | 2116 | 1 | 151 | 155 |  |
| total 2 peptides |  |  |  |  |  |  |  |  |  |  |  |  |
| --- | --- | --- | --- | --- | --- | --- | --- | --- | --- | --- | --- | --- |

tr|A0A068FDD2|A0A068FDD2\_9DIPT

back to list

  

| Protein Coverage
| Supporting Peptides
|

Protein Coverage:

Supporting Peptides:

| Peptide | Uniq | -10lgP | Mass | ppm | m/z | z | RT | Scan | #Spec | Start | End | PTM |
| --- | --- | --- | --- | --- | --- | --- | --- | --- | --- | --- | --- | --- |
| L.SMLIR.A | N | 16.79 | 618.3523 | 1003.3 | 619.9800 | 1 | 54.06 | 1684 | 1 | 10 | 14 |  |
| R.SNGIT.F | Y | 16.75 | 490.2387 | 2129.6 | 492.2900 | 1 | 66.67 | 2116 | 1 | 149 | 153 |  |
| total 2 peptides |  |  |  |  |  |  |  |  |  |  |  |  |
| --- | --- | --- | --- | --- | --- | --- | --- | --- | --- | --- | --- | --- |

tr|A0A068FJX0|A0A068FJX0\_9DIPT

back to list

  

| Protein Coverage
| Supporting Peptides
|

Protein Coverage:

Supporting Peptides:

| Peptide | Uniq | -10lgP | Mass | ppm | m/z | z | RT | Scan | #Spec | Start | End | PTM |
| --- | --- | --- | --- | --- | --- | --- | --- | --- | --- | --- | --- | --- |
| L.SMLIR.A | N | 16.79 | 618.3523 | 1003.3 | 619.9800 | 1 | 54.06 | 1684 | 1 | 12 | 16 |  |
| R.SNGIT.F | Y | 16.75 | 490.2387 | 2129.6 | 492.2900 | 1 | 66.67 | 2116 | 1 | 151 | 155 |  |
| total 2 peptides |  |  |  |  |  |  |  |  |  |  |  |  |
| --- | --- | --- | --- | --- | --- | --- | --- | --- | --- | --- | --- | --- |

tr|A0A068FE08|A0A068FE08\_9DIPT

back to list

  

| Protein Coverage
| Supporting Peptides
|

Protein Coverage:

Supporting Peptides:

| Peptide | Uniq | -10lgP | Mass | ppm | m/z | z | RT | Scan | #Spec | Start | End | PTM |
| --- | --- | --- | --- | --- | --- | --- | --- | --- | --- | --- | --- | --- |
| L.SMLIR.A | N | 16.79 | 618.3523 | 1003.3 | 619.9800 | 1 | 54.06 | 1684 | 1 | 12 | 16 |  |
| R.SNGIT.F | Y | 16.75 | 490.2387 | 2129.6 | 492.2900 | 1 | 66.67 | 2116 | 1 | 151 | 155 |  |
| total 2 peptides |  |  |  |  |  |  |  |  |  |  |  |  |
| --- | --- | --- | --- | --- | --- | --- | --- | --- | --- | --- | --- | --- |

tr|A0A068FJY2|A0A068FJY2\_9DIPT

back to list

  

| Protein Coverage
| Supporting Peptides
|

Protein Coverage:

Supporting Peptides:

| Peptide | Uniq | -10lgP | Mass | ppm | m/z | z | RT | Scan | #Spec | Start | End | PTM |
| --- | --- | --- | --- | --- | --- | --- | --- | --- | --- | --- | --- | --- |
| L.SMLIR.A | N | 16.79 | 618.3523 | 1003.3 | 619.9800 | 1 | 54.06 | 1684 | 1 | 12 | 16 |  |
| R.SNGIT.F | Y | 16.75 | 490.2387 | 2129.6 | 492.2900 | 1 | 66.67 | 2116 | 1 | 151 | 155 |  |
| total 2 peptides |  |  |  |  |  |  |  |  |  |  |  |  |
| --- | --- | --- | --- | --- | --- | --- | --- | --- | --- | --- | --- | --- |

tr|A0A068FKF2|A0A068FKF2\_9DIPT

back to list

  

| Protein Coverage
| Supporting Peptides
|

Protein Coverage:

Supporting Peptides:

| Peptide | Uniq | -10lgP | Mass | ppm | m/z | z | RT | Scan | #Spec | Start | End | PTM |
| --- | --- | --- | --- | --- | --- | --- | --- | --- | --- | --- | --- | --- |
| L.SMLIR.A | N | 16.79 | 618.3523 | 1003.3 | 619.9800 | 1 | 54.06 | 1684 | 1 | 12 | 16 |  |
| R.SNGIT.F | Y | 16.75 | 490.2387 | 2129.6 | 492.2900 | 1 | 66.67 | 2116 | 1 | 151 | 155 |  |
| total 2 peptides |  |  |  |  |  |  |  |  |  |  |  |  |
| --- | --- | --- | --- | --- | --- | --- | --- | --- | --- | --- | --- | --- |

tr|A0A068FGL3|A0A068FGL3\_9DIPT

back to list

  

| Protein Coverage
| Supporting Peptides
|

Protein Coverage:

Supporting Peptides:

| Peptide | Uniq | -10lgP | Mass | ppm | m/z | z | RT | Scan | #Spec | Start | End | PTM |
| --- | --- | --- | --- | --- | --- | --- | --- | --- | --- | --- | --- | --- |
| L.SMLIR.A | N | 16.79 | 618.3523 | 1003.3 | 619.9800 | 1 | 54.06 | 1684 | 1 | 12 | 16 |  |
| R.SNGIT.F | Y | 16.75 | 490.2387 | 2129.6 | 492.2900 | 1 | 66.67 | 2116 | 1 | 151 | 155 |  |
| total 2 peptides |  |  |  |  |  |  |  |  |  |  |  |  |
| --- | --- | --- | --- | --- | --- | --- | --- | --- | --- | --- | --- | --- |

tr|A0A068FE96|A0A068FE96\_9DIPT

back to list

  

| Protein Coverage
| Supporting Peptides
|

Protein Coverage:

Supporting Peptides:

| Peptide | Uniq | -10lgP | Mass | ppm | m/z | z | RT | Scan | #Spec | Start | End | PTM |
| --- | --- | --- | --- | --- | --- | --- | --- | --- | --- | --- | --- | --- |
| L.SMLIR.A | N | 16.79 | 618.3523 | 1003.3 | 619.9800 | 1 | 54.06 | 1684 | 1 | 12 | 16 |  |
| R.SNGIT.F | Y | 16.75 | 490.2387 | 2129.6 | 492.2900 | 1 | 66.67 | 2116 | 1 | 151 | 155 |  |
| total 2 peptides |  |  |  |  |  |  |  |  |  |  |  |  |
| --- | --- | --- | --- | --- | --- | --- | --- | --- | --- | --- | --- | --- |

tr|A0A068FG62|A0A068FG62\_9DIPT

back to list

  

| Protein Coverage
| Supporting Peptides
|

Protein Coverage:

Supporting Peptides:

| Peptide | Uniq | -10lgP | Mass | ppm | m/z | z | RT | Scan | #Spec | Start | End | PTM |
| --- | --- | --- | --- | --- | --- | --- | --- | --- | --- | --- | --- | --- |
| L.SMLIR.A | N | 16.79 | 618.3523 | 1003.3 | 619.9800 | 1 | 54.06 | 1684 | 1 | 12 | 16 |  |
| R.SNGIT.F | Y | 16.75 | 490.2387 | 2129.6 | 492.2900 | 1 | 66.67 | 2116 | 1 | 151 | 155 |  |
| total 2 peptides |  |  |  |  |  |  |  |  |  |  |  |  |
| --- | --- | --- | --- | --- | --- | --- | --- | --- | --- | --- | --- | --- |

tr|A0A068FK74|A0A068FK74\_9DIPT

back to list

  

| Protein Coverage
| Supporting Peptides
|

Protein Coverage:

Supporting Peptides:

| Peptide | Uniq | -10lgP | Mass | ppm | m/z | z | RT | Scan | #Spec | Start | End | PTM |
| --- | --- | --- | --- | --- | --- | --- | --- | --- | --- | --- | --- | --- |
| L.SMLIR.A | N | 16.79 | 618.3523 | 1003.3 | 619.9800 | 1 | 54.06 | 1684 | 1 | 12 | 16 |  |
| R.SNGIT.F | Y | 16.75 | 490.2387 | 2129.6 | 492.2900 | 1 | 66.67 | 2116 | 1 | 151 | 155 |  |
| total 2 peptides |  |  |  |  |  |  |  |  |  |  |  |  |
| --- | --- | --- | --- | --- | --- | --- | --- | --- | --- | --- | --- | --- |

tr|A0A068FEE7|A0A068FEE7\_9DIPT

back to list

  

| Protein Coverage
| Supporting Peptides
|

Protein Coverage:

Supporting Peptides:

| Peptide | Uniq | -10lgP | Mass | ppm | m/z | z | RT | Scan | #Spec | Start | End | PTM |
| --- | --- | --- | --- | --- | --- | --- | --- | --- | --- | --- | --- | --- |
| L.SMLIR.A | N | 16.79 | 618.3523 | 1003.3 | 619.9800 | 1 | 54.06 | 1684 | 1 | 12 | 16 |  |
| R.SNGIT.F | Y | 16.75 | 490.2387 | 2129.6 | 492.2900 | 1 | 66.67 | 2116 | 1 | 151 | 155 |  |
| total 2 peptides |  |  |  |  |  |  |  |  |  |  |  |  |
| --- | --- | --- | --- | --- | --- | --- | --- | --- | --- | --- | --- | --- |

tr|A0A068FEG2|A0A068FEG2\_9DIPT

back to list

  

| Protein Coverage
| Supporting Peptides
|

Protein Coverage:

Supporting Peptides:

| Peptide | Uniq | -10lgP | Mass | ppm | m/z | z | RT | Scan | #Spec | Start | End | PTM |
| --- | --- | --- | --- | --- | --- | --- | --- | --- | --- | --- | --- | --- |
| L.SMLIR.A | N | 16.79 | 618.3523 | 1003.3 | 619.9800 | 1 | 54.06 | 1684 | 1 | 12 | 16 |  |
| R.SNGIT.F | Y | 16.75 | 490.2387 | 2129.6 | 492.2900 | 1 | 66.67 | 2116 | 1 | 151 | 155 |  |
| total 2 peptides |  |  |  |  |  |  |  |  |  |  |  |  |
| --- | --- | --- | --- | --- | --- | --- | --- | --- | --- | --- | --- | --- |

tr|A0A068FD91|A0A068FD91\_9DIPT

back to list

  

| Protein Coverage
| Supporting Peptides
|

Protein Coverage:

Supporting Peptides:

| Peptide | Uniq | -10lgP | Mass | ppm | m/z | z | RT | Scan | #Spec | Start | End | PTM |
| --- | --- | --- | --- | --- | --- | --- | --- | --- | --- | --- | --- | --- |
| L.SMLIR.A | N | 16.79 | 618.3523 | 1003.3 | 619.9800 | 1 | 54.06 | 1684 | 1 | 12 | 16 |  |
| R.SNGIT.F | Y | 16.75 | 490.2387 | 2129.6 | 492.2900 | 1 | 66.67 | 2116 | 1 | 151 | 155 |  |
| total 2 peptides |  |  |  |  |  |  |  |  |  |  |  |  |
| --- | --- | --- | --- | --- | --- | --- | --- | --- | --- | --- | --- | --- |

tr|A0A068FE52|A0A068FE52\_9DIPT

back to list

  

| Protein Coverage
| Supporting Peptides
|

Protein Coverage:

Supporting Peptides:

| Peptide | Uniq | -10lgP | Mass | ppm | m/z | z | RT | Scan | #Spec | Start | End | PTM |
| --- | --- | --- | --- | --- | --- | --- | --- | --- | --- | --- | --- | --- |
| L.SMLIR.A | N | 16.79 | 618.3523 | 1003.3 | 619.9800 | 1 | 54.06 | 1684 | 1 | 12 | 16 |  |
| R.SNGIT.F | Y | 16.75 | 490.2387 | 2129.6 | 492.2900 | 1 | 66.67 | 2116 | 1 | 151 | 155 |  |
| total 2 peptides |  |  |  |  |  |  |  |  |  |  |  |  |
| --- | --- | --- | --- | --- | --- | --- | --- | --- | --- | --- | --- | --- |

tr|A0A068FEG7|A0A068FEG7\_9DIPT

back to list

  

| Protein Coverage
| Supporting Peptides
|

Protein Coverage:

Supporting Peptides:

| Peptide | Uniq | -10lgP | Mass | ppm | m/z | z | RT | Scan | #Spec | Start | End | PTM |
| --- | --- | --- | --- | --- | --- | --- | --- | --- | --- | --- | --- | --- |
| L.SMLIR.A | N | 16.79 | 618.3523 | 1003.3 | 619.9800 | 1 | 54.06 | 1684 | 1 | 12 | 16 |  |
| R.SNGIT.F | Y | 16.75 | 490.2387 | 2129.6 | 492.2900 | 1 | 66.67 | 2116 | 1 | 151 | 155 |  |
| total 2 peptides |  |  |  |  |  |  |  |  |  |  |  |  |
| --- | --- | --- | --- | --- | --- | --- | --- | --- | --- | --- | --- | --- |

tr|A0A068FEA3|A0A068FEA3\_9DIPT

back to list

  

| Protein Coverage
| Supporting Peptides
|

Protein Coverage:

Supporting Peptides:

| Peptide | Uniq | -10lgP | Mass | ppm | m/z | z | RT | Scan | #Spec | Start | End | PTM |
| --- | --- | --- | --- | --- | --- | --- | --- | --- | --- | --- | --- | --- |
| L.SMLIR.A | N | 16.79 | 618.3523 | 1003.3 | 619.9800 | 1 | 54.06 | 1684 | 1 | 12 | 16 |  |
| R.SNGIT.F | Y | 16.75 | 490.2387 | 2129.6 | 492.2900 | 1 | 66.67 | 2116 | 1 | 151 | 155 |  |
| total 2 peptides |  |  |  |  |  |  |  |  |  |  |  |  |
| --- | --- | --- | --- | --- | --- | --- | --- | --- | --- | --- | --- | --- |

tr|A0A068FFD1|A0A068FFD1\_9DIPT

back to list

  

| Protein Coverage
| Supporting Peptides
|

Protein Coverage:

Supporting Peptides:

| Peptide | Uniq | -10lgP | Mass | ppm | m/z | z | RT | Scan | #Spec | Start | End | PTM |
| --- | --- | --- | --- | --- | --- | --- | --- | --- | --- | --- | --- | --- |
| L.SMLIR.A | N | 16.79 | 618.3523 | 1003.3 | 619.9800 | 1 | 54.06 | 1684 | 1 | 12 | 16 |  |
| R.SNGIT.F | Y | 16.75 | 490.2387 | 2129.6 | 492.2900 | 1 | 66.67 | 2116 | 1 | 151 | 155 |  |
| total 2 peptides |  |  |  |  |  |  |  |  |  |  |  |  |
| --- | --- | --- | --- | --- | --- | --- | --- | --- | --- | --- | --- | --- |

tr|A0A068FKD6|A0A068FKD6\_9DIPT

back to list

  

| Protein Coverage
| Supporting Peptides
|

Protein Coverage:

Supporting Peptides:

| Peptide | Uniq | -10lgP | Mass | ppm | m/z | z | RT | Scan | #Spec | Start | End | PTM |
| --- | --- | --- | --- | --- | --- | --- | --- | --- | --- | --- | --- | --- |
| L.SMLIR.A | N | 16.79 | 618.3523 | 1003.3 | 619.9800 | 1 | 54.06 | 1684 | 1 | 12 | 16 |  |
| R.SNGIT.F | Y | 16.75 | 490.2387 | 2129.6 | 492.2900 | 1 | 66.67 | 2116 | 1 | 151 | 155 |  |
| total 2 peptides |  |  |  |  |  |  |  |  |  |  |  |  |
| --- | --- | --- | --- | --- | --- | --- | --- | --- | --- | --- | --- | --- |

tr|A0A068FKB8|A0A068FKB8\_9DIPT

back to list

  

| Protein Coverage
| Supporting Peptides
|

Protein Coverage:

Supporting Peptides:

| Peptide | Uniq | -10lgP | Mass | ppm | m/z | z | RT | Scan | #Spec | Start | End | PTM |
| --- | --- | --- | --- | --- | --- | --- | --- | --- | --- | --- | --- | --- |
| L.SMLIR.A | N | 16.79 | 618.3523 | 1003.3 | 619.9800 | 1 | 54.06 | 1684 | 1 | 12 | 16 |  |
| R.SNGIT.F | Y | 16.75 | 490.2387 | 2129.6 | 492.2900 | 1 | 66.67 | 2116 | 1 | 151 | 155 |  |
| total 2 peptides |  |  |  |  |  |  |  |  |  |  |  |  |
| --- | --- | --- | --- | --- | --- | --- | --- | --- | --- | --- | --- | --- |

tr|A0A068FJX6|A0A068FJX6\_9DIPT

back to list

  

| Protein Coverage
| Supporting Peptides
|

Protein Coverage:

Supporting Peptides:

| Peptide | Uniq | -10lgP | Mass | ppm | m/z | z | RT | Scan | #Spec | Start | End | PTM |
| --- | --- | --- | --- | --- | --- | --- | --- | --- | --- | --- | --- | --- |
| L.SMLIR.A | N | 16.79 | 618.3523 | 1003.3 | 619.9800 | 1 | 54.06 | 1684 | 1 | 12 | 16 |  |
| R.SNGIT.F | Y | 16.75 | 490.2387 | 2129.6 | 492.2900 | 1 | 66.67 | 2116 | 1 | 151 | 155 |  |
| total 2 peptides |  |  |  |  |  |  |  |  |  |  |  |  |
| --- | --- | --- | --- | --- | --- | --- | --- | --- | --- | --- | --- | --- |

tr|A0A068FGB0|A0A068FGB0\_9DIPT

back to list

  

| Protein Coverage
| Supporting Peptides
|

Protein Coverage:

Supporting Peptides:

| Peptide | Uniq | -10lgP | Mass | ppm | m/z | z | RT | Scan | #Spec | Start | End | PTM |
| --- | --- | --- | --- | --- | --- | --- | --- | --- | --- | --- | --- | --- |
| L.SMLIR.A | N | 16.79 | 618.3523 | 1003.3 | 619.9800 | 1 | 54.06 | 1684 | 1 | 12 | 16 |  |
| R.SNGIT.F | Y | 16.75 | 490.2387 | 2129.6 | 492.2900 | 1 | 66.67 | 2116 | 1 | 151 | 155 |  |
| total 2 peptides |  |  |  |  |  |  |  |  |  |  |  |  |
| --- | --- | --- | --- | --- | --- | --- | --- | --- | --- | --- | --- | --- |

tr|A0A068FDM8|A0A068FDM8\_9DIPT

back to list

  

| Protein Coverage
| Supporting Peptides
|

Protein Coverage:

Supporting Peptides:

| Peptide | Uniq | -10lgP | Mass | ppm | m/z | z | RT | Scan | #Spec | Start | End | PTM |
| --- | --- | --- | --- | --- | --- | --- | --- | --- | --- | --- | --- | --- |
| L.SMLIR.A | N | 16.79 | 618.3523 | 1003.3 | 619.9800 | 1 | 54.06 | 1684 | 1 | 12 | 16 |  |
| R.SNGIT.F | Y | 16.75 | 490.2387 | 2129.6 | 492.2900 | 1 | 66.67 | 2116 | 1 | 151 | 155 |  |
| total 2 peptides |  |  |  |  |  |  |  |  |  |  |  |  |
| --- | --- | --- | --- | --- | --- | --- | --- | --- | --- | --- | --- | --- |

tr|A0A068FK07|A0A068FK07\_9DIPT

back to list

  

| Protein Coverage
| Supporting Peptides
|

Protein Coverage:

Supporting Peptides:

| Peptide | Uniq | -10lgP | Mass | ppm | m/z | z | RT | Scan | #Spec | Start | End | PTM |
| --- | --- | --- | --- | --- | --- | --- | --- | --- | --- | --- | --- | --- |
| L.SMLIR.A | N | 16.79 | 618.3523 | 1003.3 | 619.9800 | 1 | 54.06 | 1684 | 1 | 12 | 16 |  |
| R.SNGIT.F | Y | 16.75 | 490.2387 | 2129.6 | 492.2900 | 1 | 66.67 | 2116 | 1 | 151 | 155 |  |
| total 2 peptides |  |  |  |  |  |  |  |  |  |  |  |  |
| --- | --- | --- | --- | --- | --- | --- | --- | --- | --- | --- | --- | --- |

tr|A0A068FK49|A0A068FK49\_9DIPT

back to list

  

| Protein Coverage
| Supporting Peptides
|

Protein Coverage:

Supporting Peptides:

| Peptide | Uniq | -10lgP | Mass | ppm | m/z | z | RT | Scan | #Spec | Start | End | PTM |
| --- | --- | --- | --- | --- | --- | --- | --- | --- | --- | --- | --- | --- |
| L.SMLIR.A | N | 16.79 | 618.3523 | 1003.3 | 619.9800 | 1 | 54.06 | 1684 | 1 | 12 | 16 |  |
| R.SNGIT.F | Y | 16.75 | 490.2387 | 2129.6 | 492.2900 | 1 | 66.67 | 2116 | 1 | 151 | 155 |  |
| total 2 peptides |  |  |  |  |  |  |  |  |  |  |  |  |
| --- | --- | --- | --- | --- | --- | --- | --- | --- | --- | --- | --- | --- |

tr|A0A068FDL4|A0A068FDL4\_9DIPT

back to list

  

| Protein Coverage
| Supporting Peptides
|

Protein Coverage:

Supporting Peptides:

| Peptide | Uniq | -10lgP | Mass | ppm | m/z | z | RT | Scan | #Spec | Start | End | PTM |
| --- | --- | --- | --- | --- | --- | --- | --- | --- | --- | --- | --- | --- |
| L.SMLIR.A | N | 16.79 | 618.3523 | 1003.3 | 619.9800 | 1 | 54.06 | 1684 | 1 | 12 | 16 |  |
| R.SNGIT.F | Y | 16.75 | 490.2387 | 2129.6 | 492.2900 | 1 | 66.67 | 2116 | 1 | 151 | 155 |  |
| total 2 peptides |  |  |  |  |  |  |  |  |  |  |  |  |
| --- | --- | --- | --- | --- | --- | --- | --- | --- | --- | --- | --- | --- |

tr|A0A068FKC8|A0A068FKC8\_9DIPT

back to list

  

| Protein Coverage
| Supporting Peptides
|

Protein Coverage:

Supporting Peptides:

| Peptide | Uniq | -10lgP | Mass | ppm | m/z | z | RT | Scan | #Spec | Start | End | PTM |
| --- | --- | --- | --- | --- | --- | --- | --- | --- | --- | --- | --- | --- |
| L.SMLIR.A | N | 16.79 | 618.3523 | 1003.3 | 619.9800 | 1 | 54.06 | 1684 | 1 | 12 | 16 |  |
| R.SNGIT.F | Y | 16.75 | 490.2387 | 2129.6 | 492.2900 | 1 | 66.67 | 2116 | 1 | 151 | 155 |  |
| total 2 peptides |  |  |  |  |  |  |  |  |  |  |  |  |
| --- | --- | --- | --- | --- | --- | --- | --- | --- | --- | --- | --- | --- |

tr|A0A068FGK7|A0A068FGK7\_9DIPT

back to list

  

| Protein Coverage
| Supporting Peptides
|

Protein Coverage:

Supporting Peptides:

| Peptide | Uniq | -10lgP | Mass | ppm | m/z | z | RT | Scan | #Spec | Start | End | PTM |
| --- | --- | --- | --- | --- | --- | --- | --- | --- | --- | --- | --- | --- |
| L.SMLIR.A | N | 16.79 | 618.3523 | 1003.3 | 619.9800 | 1 | 54.06 | 1684 | 1 | 12 | 16 |  |
| R.SNGIT.F | Y | 16.75 | 490.2387 | 2129.6 | 492.2900 | 1 | 66.67 | 2116 | 1 | 151 | 155 |  |
| total 2 peptides |  |  |  |  |  |  |  |  |  |  |  |  |
| --- | --- | --- | --- | --- | --- | --- | --- | --- | --- | --- | --- | --- |

tr|A0A068FGE3|A0A068FGE3\_9DIPT

back to list

  

| Protein Coverage
| Supporting Peptides
|

Protein Coverage:

Supporting Peptides:

| Peptide | Uniq | -10lgP | Mass | ppm | m/z | z | RT | Scan | #Spec | Start | End | PTM |
| --- | --- | --- | --- | --- | --- | --- | --- | --- | --- | --- | --- | --- |
| L.SMLIR.A | N | 16.79 | 618.3523 | 1003.3 | 619.9800 | 1 | 54.06 | 1684 | 1 | 12 | 16 |  |
| R.SNGIT.F | Y | 16.75 | 490.2387 | 2129.6 | 492.2900 | 1 | 66.67 | 2116 | 1 | 151 | 155 |  |
| total 2 peptides |  |  |  |  |  |  |  |  |  |  |  |  |
| --- | --- | --- | --- | --- | --- | --- | --- | --- | --- | --- | --- | --- |

tr|A0A068FGK0|A0A068FGK0\_9DIPT

back to list

  

| Protein Coverage
| Supporting Peptides
|

Protein Coverage:

Supporting Peptides:

| Peptide | Uniq | -10lgP | Mass | ppm | m/z | z | RT | Scan | #Spec | Start | End | PTM |
| --- | --- | --- | --- | --- | --- | --- | --- | --- | --- | --- | --- | --- |
| L.SMLIR.A | N | 16.79 | 618.3523 | 1003.3 | 619.9800 | 1 | 54.06 | 1684 | 1 | 12 | 16 |  |
| R.SNGIT.F | Y | 16.75 | 490.2387 | 2129.6 | 492.2900 | 1 | 66.67 | 2116 | 1 | 151 | 155 |  |
| total 2 peptides |  |  |  |  |  |  |  |  |  |  |  |  |
| --- | --- | --- | --- | --- | --- | --- | --- | --- | --- | --- | --- | --- |

tr|A0A068FD80|A0A068FD80\_9DIPT

back to list

  

| Protein Coverage
| Supporting Peptides
|

Protein Coverage:

Supporting Peptides:

| Peptide | Uniq | -10lgP | Mass | ppm | m/z | z | RT | Scan | #Spec | Start | End | PTM |
| --- | --- | --- | --- | --- | --- | --- | --- | --- | --- | --- | --- | --- |
| L.SMLIR.A | N | 16.79 | 618.3523 | 1003.3 | 619.9800 | 1 | 54.06 | 1684 | 1 | 12 | 16 |  |
| R.SNGIT.F | Y | 16.75 | 490.2387 | 2129.6 | 492.2900 | 1 | 66.67 | 2116 | 1 | 151 | 155 |  |
| total 2 peptides |  |  |  |  |  |  |  |  |  |  |  |  |
| --- | --- | --- | --- | --- | --- | --- | --- | --- | --- | --- | --- | --- |

tr|A0A068FGB7|A0A068FGB7\_9DIPT

back to list

  

| Protein Coverage
| Supporting Peptides
|

Protein Coverage:

Supporting Peptides:

| Peptide | Uniq | -10lgP | Mass | ppm | m/z | z | RT | Scan | #Spec | Start | End | PTM |
| --- | --- | --- | --- | --- | --- | --- | --- | --- | --- | --- | --- | --- |
| L.SMLIR.A | N | 16.79 | 618.3523 | 1003.3 | 619.9800 | 1 | 54.06 | 1684 | 1 | 12 | 16 |  |
| R.SNGIT.F | Y | 16.75 | 490.2387 | 2129.6 | 492.2900 | 1 | 66.67 | 2116 | 1 | 151 | 155 |  |
| total 2 peptides |  |  |  |  |  |  |  |  |  |  |  |  |
| --- | --- | --- | --- | --- | --- | --- | --- | --- | --- | --- | --- | --- |

tr|A0A068FFA7|A0A068FFA7\_9DIPT

back to list

  

| Protein Coverage
| Supporting Peptides
|

Protein Coverage:

Supporting Peptides:

| Peptide | Uniq | -10lgP | Mass | ppm | m/z | z | RT | Scan | #Spec | Start | End | PTM |
| --- | --- | --- | --- | --- | --- | --- | --- | --- | --- | --- | --- | --- |
| L.SMLIR.A | N | 16.79 | 618.3523 | 1003.3 | 619.9800 | 1 | 54.06 | 1684 | 1 | 12 | 16 |  |
| R.SNGIT.F | Y | 16.75 | 490.2387 | 2129.6 | 492.2900 | 1 | 66.67 | 2116 | 1 | 151 | 155 |  |
| total 2 peptides |  |  |  |  |  |  |  |  |  |  |  |  |
| --- | --- | --- | --- | --- | --- | --- | --- | --- | --- | --- | --- | --- |

tr|A0A068FE29|A0A068FE29\_9DIPT

back to list

  

| Protein Coverage
| Supporting Peptides
|

Protein Coverage:

Supporting Peptides:

| Peptide | Uniq | -10lgP | Mass | ppm | m/z | z | RT | Scan | #Spec | Start | End | PTM |
| --- | --- | --- | --- | --- | --- | --- | --- | --- | --- | --- | --- | --- |
| L.SMLIR.A | N | 16.79 | 618.3523 | 1003.3 | 619.9800 | 1 | 54.06 | 1684 | 1 | 12 | 16 |  |
| R.SNGIT.F | Y | 16.75 | 490.2387 | 2129.6 | 492.2900 | 1 | 66.67 | 2116 | 1 | 151 | 155 |  |
| total 2 peptides |  |  |  |  |  |  |  |  |  |  |  |  |
| --- | --- | --- | --- | --- | --- | --- | --- | --- | --- | --- | --- | --- |

tr|A0A068FFC1|A0A068FFC1\_9DIPT

back to list

  

| Protein Coverage
| Supporting Peptides
|

Protein Coverage:

Supporting Peptides:

| Peptide | Uniq | -10lgP | Mass | ppm | m/z | z | RT | Scan | #Spec | Start | End | PTM |
| --- | --- | --- | --- | --- | --- | --- | --- | --- | --- | --- | --- | --- |
| L.SMLIR.A | N | 16.79 | 618.3523 | 1003.3 | 619.9800 | 1 | 54.06 | 1684 | 1 | 12 | 16 |  |
| R.SNGIT.F | Y | 16.75 | 490.2387 | 2129.6 | 492.2900 | 1 | 66.67 | 2116 | 1 | 151 | 155 |  |
| total 2 peptides |  |  |  |  |  |  |  |  |  |  |  |  |
| --- | --- | --- | --- | --- | --- | --- | --- | --- | --- | --- | --- | --- |

tr|A0A068FDA0|A0A068FDA0\_9DIPT

back to list

  

| Protein Coverage
| Supporting Peptides
|

Protein Coverage:

Supporting Peptides:

| Peptide | Uniq | -10lgP | Mass | ppm | m/z | z | RT | Scan | #Spec | Start | End | PTM |
| --- | --- | --- | --- | --- | --- | --- | --- | --- | --- | --- | --- | --- |
| L.SMLIR.A | N | 16.79 | 618.3523 | 1003.3 | 619.9800 | 1 | 54.06 | 1684 | 1 | 12 | 16 |  |
| R.SNGIT.F | Y | 16.75 | 490.2387 | 2129.6 | 492.2900 | 1 | 66.67 | 2116 | 1 | 151 | 155 |  |
| total 2 peptides |  |  |  |  |  |  |  |  |  |  |  |  |
| --- | --- | --- | --- | --- | --- | --- | --- | --- | --- | --- | --- | --- |

tr|A0A068FF74|A0A068FF74\_9DIPT

back to list

  

| Protein Coverage
| Supporting Peptides
|

Protein Coverage:

Supporting Peptides:

| Peptide | Uniq | -10lgP | Mass | ppm | m/z | z | RT | Scan | #Spec | Start | End | PTM |
| --- | --- | --- | --- | --- | --- | --- | --- | --- | --- | --- | --- | --- |
| L.SMLIR.A | N | 16.79 | 618.3523 | 1003.3 | 619.9800 | 1 | 54.06 | 1684 | 1 | 12 | 16 |  |
| R.SNGIT.F | Y | 16.75 | 490.2387 | 2129.6 | 492.2900 | 1 | 66.67 | 2116 | 1 | 151 | 155 |  |
| total 2 peptides |  |  |  |  |  |  |  |  |  |  |  |  |
| --- | --- | --- | --- | --- | --- | --- | --- | --- | --- | --- | --- | --- |

tr|A0A068FK98|A0A068FK98\_9DIPT

back to list

  

| Protein Coverage
| Supporting Peptides
|

Protein Coverage:

Supporting Peptides:

| Peptide | Uniq | -10lgP | Mass | ppm | m/z | z | RT | Scan | #Spec | Start | End | PTM |
| --- | --- | --- | --- | --- | --- | --- | --- | --- | --- | --- | --- | --- |
| L.SMLIR.A | N | 16.79 | 618.3523 | 1003.3 | 619.9800 | 1 | 54.06 | 1684 | 1 | 12 | 16 |  |
| R.SNGIT.F | Y | 16.75 | 490.2387 | 2129.6 | 492.2900 | 1 | 66.67 | 2116 | 1 | 151 | 155 |  |
| total 2 peptides |  |  |  |  |  |  |  |  |  |  |  |  |
| --- | --- | --- | --- | --- | --- | --- | --- | --- | --- | --- | --- | --- |

tr|A0A068FKE3|A0A068FKE3\_9DIPT

back to list

  

| Protein Coverage
| Supporting Peptides
|

Protein Coverage:

Supporting Peptides:

| Peptide | Uniq | -10lgP | Mass | ppm | m/z | z | RT | Scan | #Spec | Start | End | PTM |
| --- | --- | --- | --- | --- | --- | --- | --- | --- | --- | --- | --- | --- |
| L.SMLIR.A | N | 16.79 | 618.3523 | 1003.3 | 619.9800 | 1 | 54.06 | 1684 | 1 | 12 | 16 |  |
| R.SNGIT.F | Y | 16.75 | 490.2387 | 2129.6 | 492.2900 | 1 | 66.67 | 2116 | 1 | 151 | 155 |  |
| total 2 peptides |  |  |  |  |  |  |  |  |  |  |  |  |
| --- | --- | --- | --- | --- | --- | --- | --- | --- | --- | --- | --- | --- |

tr|A0A068FGL9|A0A068FGL9\_9DIPT

back to list

  

| Protein Coverage
| Supporting Peptides
|

Protein Coverage:

Supporting Peptides:

| Peptide | Uniq | -10lgP | Mass | ppm | m/z | z | RT | Scan | #Spec | Start | End | PTM |
| --- | --- | --- | --- | --- | --- | --- | --- | --- | --- | --- | --- | --- |
| L.SMLIR.A | N | 16.79 | 618.3523 | 1003.3 | 619.9800 | 1 | 54.06 | 1684 | 1 | 12 | 16 |  |
| R.SNGIT.F | Y | 16.75 | 490.2387 | 2129.6 | 492.2900 | 1 | 66.67 | 2116 | 1 | 151 | 155 |  |
| total 2 peptides |  |  |  |  |  |  |  |  |  |  |  |  |
| --- | --- | --- | --- | --- | --- | --- | --- | --- | --- | --- | --- | --- |

tr|A0A068FJY9|A0A068FJY9\_9DIPT

back to list

  

| Protein Coverage
| Supporting Peptides
|

Protein Coverage:

Supporting Peptides:

| Peptide | Uniq | -10lgP | Mass | ppm | m/z | z | RT | Scan | #Spec | Start | End | PTM |
| --- | --- | --- | --- | --- | --- | --- | --- | --- | --- | --- | --- | --- |
| L.SMLIR.A | N | 16.79 | 618.3523 | 1003.3 | 619.9800 | 1 | 54.06 | 1684 | 1 | 12 | 16 |  |
| R.SNGIT.F | Y | 16.75 | 490.2387 | 2129.6 | 492.2900 | 1 | 66.67 | 2116 | 1 | 151 | 155 |  |
| total 2 peptides |  |  |  |  |  |  |  |  |  |  |  |  |
| --- | --- | --- | --- | --- | --- | --- | --- | --- | --- | --- | --- | --- |

tr|A0A068FF70|A0A068FF70\_9DIPT

back to list

  

| Protein Coverage
| Supporting Peptides
|

Protein Coverage:

Supporting Peptides:

| Peptide | Uniq | -10lgP | Mass | ppm | m/z | z | RT | Scan | #Spec | Start | End | PTM |
| --- | --- | --- | --- | --- | --- | --- | --- | --- | --- | --- | --- | --- |
| L.SMLIR.A | N | 16.79 | 618.3523 | 1003.3 | 619.9800 | 1 | 54.06 | 1684 | 1 | 12 | 16 |  |
| R.SNGIT.F | Y | 16.75 | 490.2387 | 2129.6 | 492.2900 | 1 | 66.67 | 2116 | 1 | 151 | 155 |  |
| total 2 peptides |  |  |  |  |  |  |  |  |  |  |  |  |
| --- | --- | --- | --- | --- | --- | --- | --- | --- | --- | --- | --- | --- |

tr|A0A068FFB2|A0A068FFB2\_9DIPT

back to list

  

| Protein Coverage
| Supporting Peptides
|

Protein Coverage:

Supporting Peptides:

| Peptide | Uniq | -10lgP | Mass | ppm | m/z | z | RT | Scan | #Spec | Start | End | PTM |
| --- | --- | --- | --- | --- | --- | --- | --- | --- | --- | --- | --- | --- |
| L.SMLIR.A | N | 16.79 | 618.3523 | 1003.3 | 619.9800 | 1 | 54.06 | 1684 | 1 | 12 | 16 |  |
| R.SNGIT.F | Y | 16.75 | 490.2387 | 2129.6 | 492.2900 | 1 | 66.67 | 2116 | 1 | 151 | 155 |  |
| total 2 peptides |  |  |  |  |  |  |  |  |  |  |  |  |
| --- | --- | --- | --- | --- | --- | --- | --- | --- | --- | --- | --- | --- |

tr|A0A068FF09|A0A068FF09\_9DIPT

back to list

  

| Protein Coverage
| Supporting Peptides
|

Protein Coverage:

Supporting Peptides:

| Peptide | Uniq | -10lgP | Mass | ppm | m/z | z | RT | Scan | #Spec | Start | End | PTM |
| --- | --- | --- | --- | --- | --- | --- | --- | --- | --- | --- | --- | --- |
| L.SMLIR.A | N | 16.79 | 618.3523 | 1003.3 | 619.9800 | 1 | 54.06 | 1684 | 1 | 12 | 16 |  |
| R.SNGIT.F | Y | 16.75 | 490.2387 | 2129.6 | 492.2900 | 1 | 66.67 | 2116 | 1 | 151 | 155 |  |
| total 2 peptides |  |  |  |  |  |  |  |  |  |  |  |  |
| --- | --- | --- | --- | --- | --- | --- | --- | --- | --- | --- | --- | --- |

tr|A0A068FK66|A0A068FK66\_9DIPT

back to list

  

| Protein Coverage
| Supporting Peptides
|

Protein Coverage:

Supporting Peptides:

| Peptide | Uniq | -10lgP | Mass | ppm | m/z | z | RT | Scan | #Spec | Start | End | PTM |
| --- | --- | --- | --- | --- | --- | --- | --- | --- | --- | --- | --- | --- |
| L.SMLIR.A | N | 16.79 | 618.3523 | 1003.3 | 619.9800 | 1 | 54.06 | 1684 | 1 | 12 | 16 |  |
| R.SNGIT.F | Y | 16.75 | 490.2387 | 2129.6 | 492.2900 | 1 | 66.67 | 2116 | 1 | 151 | 155 |  |
| total 2 peptides |  |  |  |  |  |  |  |  |  |  |  |  |
| --- | --- | --- | --- | --- | --- | --- | --- | --- | --- | --- | --- | --- |

tr|A0A068FDG6|A0A068FDG6\_9DIPT

back to list

  

| Protein Coverage
| Supporting Peptides
|

Protein Coverage:

Supporting Peptides:

| Peptide | Uniq | -10lgP | Mass | ppm | m/z | z | RT | Scan | #Spec | Start | End | PTM |
| --- | --- | --- | --- | --- | --- | --- | --- | --- | --- | --- | --- | --- |
| L.SMLIR.A | N | 16.79 | 618.3523 | 1003.3 | 619.9800 | 1 | 54.06 | 1684 | 1 | 12 | 16 |  |
| R.SNGIT.F | Y | 16.75 | 490.2387 | 2129.6 | 492.2900 | 1 | 66.67 | 2116 | 1 | 151 | 155 |  |
| total 2 peptides |  |  |  |  |  |  |  |  |  |  |  |  |
| --- | --- | --- | --- | --- | --- | --- | --- | --- | --- | --- | --- | --- |

tr|A0A068FFB6|A0A068FFB6\_9DIPT

back to list

  

| Protein Coverage
| Supporting Peptides
|

Protein Coverage:

Supporting Peptides:

| Peptide | Uniq | -10lgP | Mass | ppm | m/z | z | RT | Scan | #Spec | Start | End | PTM |
| --- | --- | --- | --- | --- | --- | --- | --- | --- | --- | --- | --- | --- |
| L.SMLIR.A | N | 16.79 | 618.3523 | 1003.3 | 619.9800 | 1 | 54.06 | 1684 | 1 | 12 | 16 |  |
| R.SNGIT.F | Y | 16.75 | 490.2387 | 2129.6 | 492.2900 | 1 | 66.67 | 2116 | 1 | 151 | 155 |  |
| total 2 peptides |  |  |  |  |  |  |  |  |  |  |  |  |
| --- | --- | --- | --- | --- | --- | --- | --- | --- | --- | --- | --- | --- |

tr|A0A068FDG0|A0A068FDG0\_9DIPT

back to list

  

| Protein Coverage
| Supporting Peptides
|

Protein Coverage:

Supporting Peptides:

| Peptide | Uniq | -10lgP | Mass | ppm | m/z | z | RT | Scan | #Spec | Start | End | PTM |
| --- | --- | --- | --- | --- | --- | --- | --- | --- | --- | --- | --- | --- |
| L.SMLIR.A | N | 16.79 | 618.3523 | 1003.3 | 619.9800 | 1 | 54.06 | 1684 | 1 | 12 | 16 |  |
| R.SNGIT.F | Y | 16.75 | 490.2387 | 2129.6 | 492.2900 | 1 | 66.67 | 2116 | 1 | 151 | 155 |  |
| total 2 peptides |  |  |  |  |  |  |  |  |  |  |  |  |
| --- | --- | --- | --- | --- | --- | --- | --- | --- | --- | --- | --- | --- |

tr|A0A068FGA6|A0A068FGA6\_9DIPT

back to list

  

| Protein Coverage
| Supporting Peptides
|

Protein Coverage:

Supporting Peptides:

| Peptide | Uniq | -10lgP | Mass | ppm | m/z | z | RT | Scan | #Spec | Start | End | PTM |
| --- | --- | --- | --- | --- | --- | --- | --- | --- | --- | --- | --- | --- |
| L.SMLIR.A | N | 16.79 | 618.3523 | 1003.3 | 619.9800 | 1 | 54.06 | 1684 | 1 | 12 | 16 |  |
| R.SNGIT.F | Y | 16.75 | 490.2387 | 2129.6 | 492.2900 | 1 | 66.67 | 2116 | 1 | 151 | 155 |  |
| total 2 peptides |  |  |  |  |  |  |  |  |  |  |  |  |
| --- | --- | --- | --- | --- | --- | --- | --- | --- | --- | --- | --- | --- |

tr|A0A068FEF8|A0A068FEF8\_9DIPT

back to list

  

| Protein Coverage
| Supporting Peptides
|

Protein Coverage:

Supporting Peptides:

| Peptide | Uniq | -10lgP | Mass | ppm | m/z | z | RT | Scan | #Spec | Start | End | PTM |
| --- | --- | --- | --- | --- | --- | --- | --- | --- | --- | --- | --- | --- |
| L.SMLIR.A | N | 16.79 | 618.3523 | 1003.3 | 619.9800 | 1 | 54.06 | 1684 | 1 | 12 | 16 |  |
| R.SNGIT.F | Y | 16.75 | 490.2387 | 2129.6 | 492.2900 | 1 | 66.67 | 2116 | 1 | 151 | 155 |  |
| total 2 peptides |  |  |  |  |  |  |  |  |  |  |  |  |
| --- | --- | --- | --- | --- | --- | --- | --- | --- | --- | --- | --- | --- |

tr|A0A068FEW5|A0A068FEW5\_9DIPT

back to list

  

| Protein Coverage
| Supporting Peptides
|

Protein Coverage:

Supporting Peptides:

| Peptide | Uniq | -10lgP | Mass | ppm | m/z | z | RT | Scan | #Spec | Start | End | PTM |
| --- | --- | --- | --- | --- | --- | --- | --- | --- | --- | --- | --- | --- |
| L.SMLIR.A | N | 16.79 | 618.3523 | 1003.3 | 619.9800 | 1 | 54.06 | 1684 | 1 | 12 | 16 |  |
| R.SNGIT.F | Y | 16.75 | 490.2387 | 2129.6 | 492.2900 | 1 | 66.67 | 2116 | 1 | 151 | 155 |  |
| total 2 peptides |  |  |  |  |  |  |  |  |  |  |  |  |
| --- | --- | --- | --- | --- | --- | --- | --- | --- | --- | --- | --- | --- |

tr|A0A068FG98|A0A068FG98\_9DIPT

back to list

  

| Protein Coverage
| Supporting Peptides
|

Protein Coverage:

Supporting Peptides:

| Peptide | Uniq | -10lgP | Mass | ppm | m/z | z | RT | Scan | #Spec | Start | End | PTM |
| --- | --- | --- | --- | --- | --- | --- | --- | --- | --- | --- | --- | --- |
| L.SMLIR.A | N | 16.79 | 618.3523 | 1003.3 | 619.9800 | 1 | 54.06 | 1684 | 1 | 12 | 16 |  |
| R.SNGIT.F | Y | 16.75 | 490.2387 | 2129.6 | 492.2900 | 1 | 66.67 | 2116 | 1 | 151 | 155 |  |
| total 2 peptides |  |  |  |  |  |  |  |  |  |  |  |  |
| --- | --- | --- | --- | --- | --- | --- | --- | --- | --- | --- | --- | --- |

tr|A0A068FK59|A0A068FK59\_9DIPT

back to list

  

| Protein Coverage
| Supporting Peptides
|

Protein Coverage:

Supporting Peptides:

| Peptide | Uniq | -10lgP | Mass | ppm | m/z | z | RT | Scan | #Spec | Start | End | PTM |
| --- | --- | --- | --- | --- | --- | --- | --- | --- | --- | --- | --- | --- |
| L.SMLIR.A | N | 16.79 | 618.3523 | 1003.3 | 619.9800 | 1 | 54.06 | 1684 | 1 | 12 | 16 |  |
| R.SNGIT.F | Y | 16.75 | 490.2387 | 2129.6 | 492.2900 | 1 | 66.67 | 2116 | 1 | 151 | 155 |  |
| total 2 peptides |  |  |  |  |  |  |  |  |  |  |  |  |
| --- | --- | --- | --- | --- | --- | --- | --- | --- | --- | --- | --- | --- |

tr|A0A068FDM2|A0A068FDM2\_9DIPT

back to list

  

| Protein Coverage
| Supporting Peptides
|

Protein Coverage:

Supporting Peptides:

| Peptide | Uniq | -10lgP | Mass | ppm | m/z | z | RT | Scan | #Spec | Start | End | PTM |
| --- | --- | --- | --- | --- | --- | --- | --- | --- | --- | --- | --- | --- |
| L.SMLIR.A | N | 16.79 | 618.3523 | 1003.3 | 619.9800 | 1 | 54.06 | 1684 | 1 | 12 | 16 |  |
| R.SNGIT.F | Y | 16.75 | 490.2387 | 2129.6 | 492.2900 | 1 | 66.67 | 2116 | 1 | 151 | 155 |  |
| total 2 peptides |  |  |  |  |  |  |  |  |  |  |  |  |
| --- | --- | --- | --- | --- | --- | --- | --- | --- | --- | --- | --- | --- |

tr|A0A068FFC7|A0A068FFC7\_9DIPT

back to list

  

| Protein Coverage
| Supporting Peptides
|

Protein Coverage:

Supporting Peptides:

| Peptide | Uniq | -10lgP | Mass | ppm | m/z | z | RT | Scan | #Spec | Start | End | PTM |
| --- | --- | --- | --- | --- | --- | --- | --- | --- | --- | --- | --- | --- |
| L.SMLIR.A | N | 16.79 | 618.3523 | 1003.3 | 619.9800 | 1 | 54.06 | 1684 | 1 | 12 | 16 |  |
| R.SNGIT.F | Y | 16.75 | 490.2387 | 2129.6 | 492.2900 | 1 | 66.67 | 2116 | 1 | 151 | 155 |  |
| total 2 peptides |  |  |  |  |  |  |  |  |  |  |  |  |
| --- | --- | --- | --- | --- | --- | --- | --- | --- | --- | --- | --- | --- |

tr|A0A068FGI6|A0A068FGI6\_9DIPT

back to list

  

| Protein Coverage
| Supporting Peptides
|

Protein Coverage:

Supporting Peptides:

| Peptide | Uniq | -10lgP | Mass | ppm | m/z | z | RT | Scan | #Spec | Start | End | PTM |
| --- | --- | --- | --- | --- | --- | --- | --- | --- | --- | --- | --- | --- |
| L.SMLIR.A | N | 16.79 | 618.3523 | 1003.3 | 619.9800 | 1 | 54.06 | 1684 | 1 | 12 | 16 |  |
| R.SNGIT.F | Y | 16.75 | 490.2387 | 2129.6 | 492.2900 | 1 | 66.67 | 2116 | 1 | 151 | 155 |  |
| total 2 peptides |  |  |  |  |  |  |  |  |  |  |  |  |
| --- | --- | --- | --- | --- | --- | --- | --- | --- | --- | --- | --- | --- |

tr|A0A068FE36|A0A068FE36\_9DIPT

back to list

  

| Protein Coverage
| Supporting Peptides
|

Protein Coverage:

Supporting Peptides:

| Peptide | Uniq | -10lgP | Mass | ppm | m/z | z | RT | Scan | #Spec | Start | End | PTM |
| --- | --- | --- | --- | --- | --- | --- | --- | --- | --- | --- | --- | --- |
| L.SMLIR.A | N | 16.79 | 618.3523 | 1003.3 | 619.9800 | 1 | 54.06 | 1684 | 1 | 12 | 16 |  |
| R.SNGIT.F | Y | 16.75 | 490.2387 | 2129.6 | 492.2900 | 1 | 66.67 | 2116 | 1 | 151 | 155 |  |
| total 2 peptides |  |  |  |  |  |  |  |  |  |  |  |  |
| --- | --- | --- | --- | --- | --- | --- | --- | --- | --- | --- | --- | --- |

tr|A0A068FD97|A0A068FD97\_9DIPT

back to list

  

| Protein Coverage
| Supporting Peptides
|

Protein Coverage:

Supporting Peptides:

| Peptide | Uniq | -10lgP | Mass | ppm | m/z | z | RT | Scan | #Spec | Start | End | PTM |
| --- | --- | --- | --- | --- | --- | --- | --- | --- | --- | --- | --- | --- |
| L.SMLIR.A | N | 16.79 | 618.3523 | 1003.3 | 619.9800 | 1 | 54.06 | 1684 | 1 | 12 | 16 |  |
| R.SNGIT.F | Y | 16.75 | 490.2387 | 2129.6 | 492.2900 | 1 | 66.67 | 2116 | 1 | 151 | 155 |  |
| total 2 peptides |  |  |  |  |  |  |  |  |  |  |  |  |
| --- | --- | --- | --- | --- | --- | --- | --- | --- | --- | --- | --- | --- |

tr|A0A068FDL8|A0A068FDL8\_9DIPT

back to list

  

| Protein Coverage
| Supporting Peptides
|

Protein Coverage:

Supporting Peptides:

| Peptide | Uniq | -10lgP | Mass | ppm | m/z | z | RT | Scan | #Spec | Start | End | PTM |
| --- | --- | --- | --- | --- | --- | --- | --- | --- | --- | --- | --- | --- |
| L.SMLIR.A | N | 16.79 | 618.3523 | 1003.3 | 619.9800 | 1 | 54.06 | 1684 | 1 | 12 | 16 |  |
| R.SNGIT.F | Y | 16.75 | 490.2387 | 2129.6 | 492.2900 | 1 | 66.67 | 2116 | 1 | 151 | 155 |  |
| total 2 peptides |  |  |  |  |  |  |  |  |  |  |  |  |
| --- | --- | --- | --- | --- | --- | --- | --- | --- | --- | --- | --- | --- |

tr|A0A068FE04|A0A068FE04\_9DIPT

back to list

  

| Protein Coverage
| Supporting Peptides
|

Protein Coverage:

Supporting Peptides:

| Peptide | Uniq | -10lgP | Mass | ppm | m/z | z | RT | Scan | #Spec | Start | End | PTM |
| --- | --- | --- | --- | --- | --- | --- | --- | --- | --- | --- | --- | --- |
| L.SMLIR.A | N | 16.79 | 618.3523 | 1003.3 | 619.9800 | 1 | 54.06 | 1684 | 1 | 12 | 16 |  |
| R.SNGIT.F | Y | 16.75 | 490.2387 | 2129.6 | 492.2900 | 1 | 66.67 | 2116 | 1 | 151 | 155 |  |
| total 2 peptides |  |  |  |  |  |  |  |  |  |  |  |  |
| --- | --- | --- | --- | --- | --- | --- | --- | --- | --- | --- | --- | --- |

tr|A0A068FDA5|A0A068FDA5\_9DIPT

back to list

  

| Protein Coverage
| Supporting Peptides
|

Protein Coverage:

Supporting Peptides:

| Peptide | Uniq | -10lgP | Mass | ppm | m/z | z | RT | Scan | #Spec | Start | End | PTM |
| --- | --- | --- | --- | --- | --- | --- | --- | --- | --- | --- | --- | --- |
| L.SMLIR.A | N | 16.79 | 618.3523 | 1003.3 | 619.9800 | 1 | 54.06 | 1684 | 1 | 12 | 16 |  |
| R.SNGIT.F | Y | 16.75 | 490.2387 | 2129.6 | 492.2900 | 1 | 66.67 | 2116 | 1 | 151 | 155 |  |
| total 2 peptides |  |  |  |  |  |  |  |  |  |  |  |  |
| --- | --- | --- | --- | --- | --- | --- | --- | --- | --- | --- | --- | --- |

tr|A0A068FG87|A0A068FG87\_9DIPT

back to list

  

| Protein Coverage
| Supporting Peptides
|

Protein Coverage:

Supporting Peptides:

| Peptide | Uniq | -10lgP | Mass | ppm | m/z | z | RT | Scan | #Spec | Start | End | PTM |
| --- | --- | --- | --- | --- | --- | --- | --- | --- | --- | --- | --- | --- |
| L.SMLIR.A | N | 16.79 | 618.3523 | 1003.3 | 619.9800 | 1 | 54.06 | 1684 | 1 | 12 | 16 |  |
| R.SNGIT.F | Y | 16.75 | 490.2387 | 2129.6 | 492.2900 | 1 | 66.67 | 2116 | 1 | 151 | 155 |  |
| total 2 peptides |  |  |  |  |  |  |  |  |  |  |  |  |
| --- | --- | --- | --- | --- | --- | --- | --- | --- | --- | --- | --- | --- |

tr|A0A068FGC1|A0A068FGC1\_9DIPT

back to list

  

| Protein Coverage
| Supporting Peptides
|

Protein Coverage:

Supporting Peptides:

| Peptide | Uniq | -10lgP | Mass | ppm | m/z | z | RT | Scan | #Spec | Start | End | PTM |
| --- | --- | --- | --- | --- | --- | --- | --- | --- | --- | --- | --- | --- |
| L.SMLIR.A | N | 16.79 | 618.3523 | 1003.3 | 619.9800 | 1 | 54.06 | 1684 | 1 | 12 | 16 |  |
| R.SNGIT.F | Y | 16.75 | 490.2387 | 2129.6 | 492.2900 | 1 | 66.67 | 2116 | 1 | 151 | 155 |  |
| total 2 peptides |  |  |  |  |  |  |  |  |  |  |  |  |
| --- | --- | --- | --- | --- | --- | --- | --- | --- | --- | --- | --- | --- |

tr|A0A068FDL0|A0A068FDL0\_9DIPT

back to list

  

| Protein Coverage
| Supporting Peptides
|

Protein Coverage:

Supporting Peptides:

| Peptide | Uniq | -10lgP | Mass | ppm | m/z | z | RT | Scan | #Spec | Start | End | PTM |
| --- | --- | --- | --- | --- | --- | --- | --- | --- | --- | --- | --- | --- |
| L.SMLIR.A | N | 16.79 | 618.3523 | 1003.3 | 619.9800 | 1 | 54.06 | 1684 | 1 | 12 | 16 |  |
| R.SNGIT.F | Y | 16.75 | 490.2387 | 2129.6 | 492.2900 | 1 | 66.67 | 2116 | 1 | 151 | 155 |  |
| total 2 peptides |  |  |  |  |  |  |  |  |  |  |  |  |
| --- | --- | --- | --- | --- | --- | --- | --- | --- | --- | --- | --- | --- |

tr|A0A068FDF4|A0A068FDF4\_9DIPT

back to list

  

| Protein Coverage
| Supporting Peptides
|

Protein Coverage:

Supporting Peptides:

| Peptide | Uniq | -10lgP | Mass | ppm | m/z | z | RT | Scan | #Spec | Start | End | PTM |
| --- | --- | --- | --- | --- | --- | --- | --- | --- | --- | --- | --- | --- |
| L.SMLIR.A | N | 16.79 | 618.3523 | 1003.3 | 619.9800 | 1 | 54.06 | 1684 | 1 | 12 | 16 |  |
| R.SNGIT.F | Y | 16.75 | 490.2387 | 2129.6 | 492.2900 | 1 | 66.67 | 2116 | 1 | 151 | 155 |  |
| total 2 peptides |  |  |  |  |  |  |  |  |  |  |  |  |
| --- | --- | --- | --- | --- | --- | --- | --- | --- | --- | --- | --- | --- |

tr|A0A068FD62|A0A068FD62\_9DIPT

back to list

  

| Protein Coverage
| Supporting Peptides
|

Protein Coverage:

Supporting Peptides:

| Peptide | Uniq | -10lgP | Mass | ppm | m/z | z | RT | Scan | #Spec | Start | End | PTM |
| --- | --- | --- | --- | --- | --- | --- | --- | --- | --- | --- | --- | --- |
| L.SMLIR.A | N | 16.79 | 618.3523 | 1003.3 | 619.9800 | 1 | 54.06 | 1684 | 1 | 12 | 16 |  |
| R.SNGIT.F | Y | 16.75 | 490.2387 | 2129.6 | 492.2900 | 1 | 66.67 | 2116 | 1 | 151 | 155 |  |
| total 2 peptides |  |  |  |  |  |  |  |  |  |  |  |  |
| --- | --- | --- | --- | --- | --- | --- | --- | --- | --- | --- | --- | --- |

tr|A0A068FDC0|A0A068FDC0\_9DIPT

back to list

  

| Protein Coverage
| Supporting Peptides
|

Protein Coverage:

Supporting Peptides:

| Peptide | Uniq | -10lgP | Mass | ppm | m/z | z | RT | Scan | #Spec | Start | End | PTM |
| --- | --- | --- | --- | --- | --- | --- | --- | --- | --- | --- | --- | --- |
| L.SMLIR.A | N | 16.79 | 618.3523 | 1003.3 | 619.9800 | 1 | 54.06 | 1684 | 1 | 12 | 16 |  |
| R.SNGIT.F | Y | 16.75 | 490.2387 | 2129.6 | 492.2900 | 1 | 66.67 | 2116 | 1 | 151 | 155 |  |
| total 2 peptides |  |  |  |  |  |  |  |  |  |  |  |  |
| --- | --- | --- | --- | --- | --- | --- | --- | --- | --- | --- | --- | --- |

tr|A0A068FKG1|A0A068FKG1\_9DIPT

back to list

  

| Protein Coverage
| Supporting Peptides
|

Protein Coverage:

Supporting Peptides:

| Peptide | Uniq | -10lgP | Mass | ppm | m/z | z | RT | Scan | #Spec | Start | End | PTM |
| --- | --- | --- | --- | --- | --- | --- | --- | --- | --- | --- | --- | --- |
| L.SMLIR.A | N | 16.79 | 618.3523 | 1003.3 | 619.9800 | 1 | 54.06 | 1684 | 1 | 12 | 16 |  |
| R.SNGIT.F | Y | 16.75 | 490.2387 | 2129.6 | 492.2900 | 1 | 66.67 | 2116 | 1 | 151 | 155 |  |
| total 2 peptides |  |  |  |  |  |  |  |  |  |  |  |  |
| --- | --- | --- | --- | --- | --- | --- | --- | --- | --- | --- | --- | --- |

tr|A0A068FEF2|A0A068FEF2\_9DIPT

back to list

  

| Protein Coverage
| Supporting Peptides
|

Protein Coverage:

Supporting Peptides:

| Peptide | Uniq | -10lgP | Mass | ppm | m/z | z | RT | Scan | #Spec | Start | End | PTM |
| --- | --- | --- | --- | --- | --- | --- | --- | --- | --- | --- | --- | --- |
| L.SMLIR.A | N | 16.79 | 618.3523 | 1003.3 | 619.9800 | 1 | 54.06 | 1684 | 1 | 12 | 16 |  |
| R.SNGIT.F | Y | 16.75 | 490.2387 | 2129.6 | 492.2900 | 1 | 66.67 | 2116 | 1 | 151 | 155 |  |
| total 2 peptides |  |  |  |  |  |  |  |  |  |  |  |  |
| --- | --- | --- | --- | --- | --- | --- | --- | --- | --- | --- | --- | --- |

tr|A0A068FEH2|A0A068FEH2\_9DIPT

back to list

  

| Protein Coverage
| Supporting Peptides
|

Protein Coverage:

Supporting Peptides:

| Peptide | Uniq | -10lgP | Mass | ppm | m/z | z | RT | Scan | #Spec | Start | End | PTM |
| --- | --- | --- | --- | --- | --- | --- | --- | --- | --- | --- | --- | --- |
| L.SMLIR.A | N | 16.79 | 618.3523 | 1003.3 | 619.9800 | 1 | 54.06 | 1684 | 1 | 12 | 16 |  |
| R.SNGIT.F | Y | 16.75 | 490.2387 | 2129.6 | 492.2900 | 1 | 66.67 | 2116 | 1 | 151 | 155 |  |
| total 2 peptides |  |  |  |  |  |  |  |  |  |  |  |  |
| --- | --- | --- | --- | --- | --- | --- | --- | --- | --- | --- | --- | --- |

tr|A0A068FG94|A0A068FG94\_9DIPT

back to list

  

| Protein Coverage
| Supporting Peptides
|

Protein Coverage:

Supporting Peptides:

| Peptide | Uniq | -10lgP | Mass | ppm | m/z | z | RT | Scan | #Spec | Start | End | PTM |
| --- | --- | --- | --- | --- | --- | --- | --- | --- | --- | --- | --- | --- |
| L.SMLIR.A | N | 16.79 | 618.3523 | 1003.3 | 619.9800 | 1 | 54.06 | 1684 | 1 | 12 | 16 |  |
| R.SNGIT.F | Y | 16.75 | 490.2387 | 2129.6 | 492.2900 | 1 | 66.67 | 2116 | 1 | 151 | 155 |  |
| total 2 peptides |  |  |  |  |  |  |  |  |  |  |  |  |
| --- | --- | --- | --- | --- | --- | --- | --- | --- | --- | --- | --- | --- |

tr|A0A068FEZ8|A0A068FEZ8\_9DIPT

back to list

  

| Protein Coverage
| Supporting Peptides
|

Protein Coverage:

Supporting Peptides:

| Peptide | Uniq | -10lgP | Mass | ppm | m/z | z | RT | Scan | #Spec | Start | End | PTM |
| --- | --- | --- | --- | --- | --- | --- | --- | --- | --- | --- | --- | --- |
| L.SMLIR.A | N | 16.79 | 618.3523 | 1003.3 | 619.9800 | 1 | 54.06 | 1684 | 1 | 12 | 16 |  |
| R.SNGIT.F | Y | 16.75 | 490.2387 | 2129.6 | 492.2900 | 1 | 66.67 | 2116 | 1 | 151 | 155 |  |
| total 2 peptides |  |  |  |  |  |  |  |  |  |  |  |  |
| --- | --- | --- | --- | --- | --- | --- | --- | --- | --- | --- | --- | --- |

tr|A0A068FK19|A0A068FK19\_9DIPT

back to list

  

| Protein Coverage
| Supporting Peptides
|

Protein Coverage:

Supporting Peptides:

| Peptide | Uniq | -10lgP | Mass | ppm | m/z | z | RT | Scan | #Spec | Start | End | PTM |
| --- | --- | --- | --- | --- | --- | --- | --- | --- | --- | --- | --- | --- |
| L.SMLIR.A | N | 16.79 | 618.3523 | 1003.3 | 619.9800 | 1 | 54.06 | 1684 | 1 | 12 | 16 |  |
| R.SNGIT.F | Y | 16.75 | 490.2387 | 2129.6 | 492.2900 | 1 | 66.67 | 2116 | 1 | 151 | 155 |  |
| total 2 peptides |  |  |  |  |  |  |  |  |  |  |  |  |
| --- | --- | --- | --- | --- | --- | --- | --- | --- | --- | --- | --- | --- |

tr|A0A068FEC3|A0A068FEC3\_9DIPT

back to list

  

| Protein Coverage
| Supporting Peptides
|

Protein Coverage:

Supporting Peptides:

| Peptide | Uniq | -10lgP | Mass | ppm | m/z | z | RT | Scan | #Spec | Start | End | PTM |
| --- | --- | --- | --- | --- | --- | --- | --- | --- | --- | --- | --- | --- |
| L.SMLIR.A | N | 16.79 | 618.3523 | 1003.3 | 619.9800 | 1 | 54.06 | 1684 | 1 | 12 | 16 |  |
| R.SNGIT.F | Y | 16.75 | 490.2387 | 2129.6 | 492.2900 | 1 | 66.67 | 2116 | 1 | 151 | 155 |  |
| total 2 peptides |  |  |  |  |  |  |  |  |  |  |  |  |
| --- | --- | --- | --- | --- | --- | --- | --- | --- | --- | --- | --- | --- |

tr|A0A068FF05|A0A068FF05\_9DIPT

back to list

  

| Protein Coverage
| Supporting Peptides
|

Protein Coverage:

Supporting Peptides:

| Peptide | Uniq | -10lgP | Mass | ppm | m/z | z | RT | Scan | #Spec | Start | End | PTM |
| --- | --- | --- | --- | --- | --- | --- | --- | --- | --- | --- | --- | --- |
| L.SMLIR.A | N | 16.79 | 618.3523 | 1003.3 | 619.9800 | 1 | 54.06 | 1684 | 1 | 12 | 16 |  |
| R.SNGIT.F | Y | 16.75 | 490.2387 | 2129.6 | 492.2900 | 1 | 66.67 | 2116 | 1 | 151 | 155 |  |
| total 2 peptides |  |  |  |  |  |  |  |  |  |  |  |  |
| --- | --- | --- | --- | --- | --- | --- | --- | --- | --- | --- | --- | --- |

tr|A0A068FF65|A0A068FF65\_9DIPT

back to list

  

| Protein Coverage
| Supporting Peptides
|

Protein Coverage:

Supporting Peptides:

| Peptide | Uniq | -10lgP | Mass | ppm | m/z | z | RT | Scan | #Spec | Start | End | PTM |
| --- | --- | --- | --- | --- | --- | --- | --- | --- | --- | --- | --- | --- |
| L.SMLIR.A | N | 16.79 | 618.3523 | 1003.3 | 619.9800 | 1 | 54.06 | 1684 | 1 | 12 | 16 |  |
| R.SNGIT.F | Y | 16.75 | 490.2387 | 2129.6 | 492.2900 | 1 | 66.67 | 2116 | 1 | 151 | 155 |  |
| total 2 peptides |  |  |  |  |  |  |  |  |  |  |  |  |
| --- | --- | --- | --- | --- | --- | --- | --- | --- | --- | --- | --- | --- |

tr|A0A0G2STG6|A0A0G2STG6\_9DIPT

back to list

  

| Protein Coverage
| Supporting Peptides
|

Protein Coverage:

Supporting Peptides:

| Peptide | Uniq | -10lgP | Mass | ppm | m/z | z | RT | Scan | #Spec | Start | End | PTM |
| --- | --- | --- | --- | --- | --- | --- | --- | --- | --- | --- | --- | --- |
| L.SMLIR.A | N | 16.79 | 618.3523 | 1003.3 | 619.9800 | 1 | 54.06 | 1684 | 1 | 20 | 24 |  |
| R.SNGIT.F | Y | 16.75 | 490.2387 | 2129.6 | 492.2900 | 1 | 66.67 | 2116 | 1 | 159 | 163 |  |
| total 2 peptides |  |  |  |  |  |  |  |  |  |  |  |  |
| --- | --- | --- | --- | --- | --- | --- | --- | --- | --- | --- | --- | --- |

tr|A0A0G2STG2|A0A0G2STG2\_9DIPT

back to list

  

| Protein Coverage
| Supporting Peptides
|

Protein Coverage:

Supporting Peptides:

| Peptide | Uniq | -10lgP | Mass | ppm | m/z | z | RT | Scan | #Spec | Start | End | PTM |
| --- | --- | --- | --- | --- | --- | --- | --- | --- | --- | --- | --- | --- |
| L.SMLIR.A | N | 16.79 | 618.3523 | 1003.3 | 619.9800 | 1 | 54.06 | 1684 | 1 | 20 | 24 |  |
| R.SNGIT.F | Y | 16.75 | 490.2387 | 2129.6 | 492.2900 | 1 | 66.67 | 2116 | 1 | 159 | 163 |  |
| total 2 peptides |  |  |  |  |  |  |  |  |  |  |  |  |
| --- | --- | --- | --- | --- | --- | --- | --- | --- | --- | --- | --- | --- |

tr|A0A0G2STA7|A0A0G2STA7\_9DIPT

back to list

  

| Protein Coverage
| Supporting Peptides
|

Protein Coverage:

Supporting Peptides:

| Peptide | Uniq | -10lgP | Mass | ppm | m/z | z | RT | Scan | #Spec | Start | End | PTM |
| --- | --- | --- | --- | --- | --- | --- | --- | --- | --- | --- | --- | --- |
| L.SMLIR.A | N | 16.79 | 618.3523 | 1003.3 | 619.9800 | 1 | 54.06 | 1684 | 1 | 20 | 24 |  |
| R.SNGIT.F | Y | 16.75 | 490.2387 | 2129.6 | 492.2900 | 1 | 66.67 | 2116 | 1 | 159 | 163 |  |
| total 2 peptides |  |  |  |  |  |  |  |  |  |  |  |  |
| --- | --- | --- | --- | --- | --- | --- | --- | --- | --- | --- | --- | --- |

tr|A0A0G2STC9|A0A0G2STC9\_9DIPT

back to list

  

| Protein Coverage
| Supporting Peptides
|

Protein Coverage:

Supporting Peptides:

| Peptide | Uniq | -10lgP | Mass | ppm | m/z | z | RT | Scan | #Spec | Start | End | PTM |
| --- | --- | --- | --- | --- | --- | --- | --- | --- | --- | --- | --- | --- |
| L.SMLIR.A | N | 16.79 | 618.3523 | 1003.3 | 619.9800 | 1 | 54.06 | 1684 | 1 | 20 | 24 |  |
| R.SNGIT.F | Y | 16.75 | 490.2387 | 2129.6 | 492.2900 | 1 | 66.67 | 2116 | 1 | 159 | 163 |  |
| total 2 peptides |  |  |  |  |  |  |  |  |  |  |  |  |
| --- | --- | --- | --- | --- | --- | --- | --- | --- | --- | --- | --- | --- |

tr|A0A0G2STC8|A0A0G2STC8\_9DIPT

back to list

  

| Protein Coverage
| Supporting Peptides
|

Protein Coverage:

Supporting Peptides:

| Peptide | Uniq | -10lgP | Mass | ppm | m/z | z | RT | Scan | #Spec | Start | End | PTM |
| --- | --- | --- | --- | --- | --- | --- | --- | --- | --- | --- | --- | --- |
| L.SMLIR.A | N | 16.79 | 618.3523 | 1003.3 | 619.9800 | 1 | 54.06 | 1684 | 1 | 20 | 24 |  |
| R.SNGIT.F | Y | 16.75 | 490.2387 | 2129.6 | 492.2900 | 1 | 66.67 | 2116 | 1 | 159 | 163 |  |
| total 2 peptides |  |  |  |  |  |  |  |  |  |  |  |  |
| --- | --- | --- | --- | --- | --- | --- | --- | --- | --- | --- | --- | --- |

tr|A0A0F6PRQ8|A0A0F6PRQ8\_9DIPT

back to list

  

| Protein Coverage
| Supporting Peptides
|

Protein Coverage:

Supporting Peptides:

| Peptide | Uniq | -10lgP | Mass | ppm | m/z | z | RT | Scan | #Spec | Start | End | PTM |
| --- | --- | --- | --- | --- | --- | --- | --- | --- | --- | --- | --- | --- |
| L.SMLIR.A | N | 16.79 | 618.3523 | 1003.3 | 619.9800 | 1 | 54.06 | 1684 | 1 | 21 | 25 |  |
| R.SNGIT.F | Y | 16.75 | 490.2387 | 2129.6 | 492.2900 | 1 | 66.67 | 2116 | 1 | 160 | 164 |  |
| total 2 peptides |  |  |  |  |  |  |  |  |  |  |  |  |
| --- | --- | --- | --- | --- | --- | --- | --- | --- | --- | --- | --- | --- |

tr|A0A0F6PPG4|A0A0F6PPG4\_9DIPT

back to list

  

| Protein Coverage
| Supporting Peptides
|

Protein Coverage:

Supporting Peptides:

| Peptide | Uniq | -10lgP | Mass | ppm | m/z | z | RT | Scan | #Spec | Start | End | PTM |
| --- | --- | --- | --- | --- | --- | --- | --- | --- | --- | --- | --- | --- |
| L.SMLIR.A | N | 16.79 | 618.3523 | 1003.3 | 619.9800 | 1 | 54.06 | 1684 | 1 | 21 | 25 |  |
| R.SNGIT.F | Y | 16.75 | 490.2387 | 2129.6 | 492.2900 | 1 | 66.67 | 2116 | 1 | 160 | 164 |  |
| total 2 peptides |  |  |  |  |  |  |  |  |  |  |  |  |
| --- | --- | --- | --- | --- | --- | --- | --- | --- | --- | --- | --- | --- |

tr|A0A0F6PRP5|A0A0F6PRP5\_9DIPT

back to list

  

| Protein Coverage
| Supporting Peptides
|

Protein Coverage:

Supporting Peptides:

| Peptide | Uniq | -10lgP | Mass | ppm | m/z | z | RT | Scan | #Spec | Start | End | PTM |
| --- | --- | --- | --- | --- | --- | --- | --- | --- | --- | --- | --- | --- |
| L.SMLIR.A | N | 16.79 | 618.3523 | 1003.3 | 619.9800 | 1 | 54.06 | 1684 | 1 | 21 | 25 |  |
| R.SNGIT.F | Y | 16.75 | 490.2387 | 2129.6 | 492.2900 | 1 | 66.67 | 2116 | 1 | 160 | 164 |  |
| total 2 peptides |  |  |  |  |  |  |  |  |  |  |  |  |
| --- | --- | --- | --- | --- | --- | --- | --- | --- | --- | --- | --- | --- |

tr|A0A0F6PQP9|A0A0F6PQP9\_9DIPT

back to list

  

| Protein Coverage
| Supporting Peptides
|

Protein Coverage:

Supporting Peptides:

| Peptide | Uniq | -10lgP | Mass | ppm | m/z | z | RT | Scan | #Spec | Start | End | PTM |
| --- | --- | --- | --- | --- | --- | --- | --- | --- | --- | --- | --- | --- |
| L.SMLIR.A | N | 16.79 | 618.3523 | 1003.3 | 619.9800 | 1 | 54.06 | 1684 | 1 | 21 | 25 |  |
| R.SNGIT.F | Y | 16.75 | 490.2387 | 2129.6 | 492.2900 | 1 | 66.67 | 2116 | 1 | 160 | 164 |  |
| total 2 peptides |  |  |  |  |  |  |  |  |  |  |  |  |
| --- | --- | --- | --- | --- | --- | --- | --- | --- | --- | --- | --- | --- |

tr|A0A0F6PRN0|A0A0F6PRN0\_9DIPT

back to list

  

| Protein Coverage
| Supporting Peptides
|

Protein Coverage:

Supporting Peptides:

| Peptide | Uniq | -10lgP | Mass | ppm | m/z | z | RT | Scan | #Spec | Start | End | PTM |
| --- | --- | --- | --- | --- | --- | --- | --- | --- | --- | --- | --- | --- |
| L.SMLIR.A | N | 16.79 | 618.3523 | 1003.3 | 619.9800 | 1 | 54.06 | 1684 | 1 | 21 | 25 |  |
| R.SNGIT.F | Y | 16.75 | 490.2387 | 2129.6 | 492.2900 | 1 | 66.67 | 2116 | 1 | 160 | 164 |  |
| total 2 peptides |  |  |  |  |  |  |  |  |  |  |  |  |
| --- | --- | --- | --- | --- | --- | --- | --- | --- | --- | --- | --- | --- |

tr|A0A0F6PRR4|A0A0F6PRR4\_9DIPT

back to list

  

| Protein Coverage
| Supporting Peptides
|

Protein Coverage:

Supporting Peptides:

| Peptide | Uniq | -10lgP | Mass | ppm | m/z | z | RT | Scan | #Spec | Start | End | PTM |
| --- | --- | --- | --- | --- | --- | --- | --- | --- | --- | --- | --- | --- |
| L.SMLIR.A | N | 16.79 | 618.3523 | 1003.3 | 619.9800 | 1 | 54.06 | 1684 | 1 | 21 | 25 |  |
| R.SNGIT.F | Y | 16.75 | 490.2387 | 2129.6 | 492.2900 | 1 | 66.67 | 2116 | 1 | 160 | 164 |  |
| total 2 peptides |  |  |  |  |  |  |  |  |  |  |  |  |
| --- | --- | --- | --- | --- | --- | --- | --- | --- | --- | --- | --- | --- |

tr|A0A0F6PQP5|A0A0F6PQP5\_9DIPT

back to list

  

| Protein Coverage
| Supporting Peptides
|

Protein Coverage:

Supporting Peptides:

| Peptide | Uniq | -10lgP | Mass | ppm | m/z | z | RT | Scan | #Spec | Start | End | PTM |
| --- | --- | --- | --- | --- | --- | --- | --- | --- | --- | --- | --- | --- |
| L.SMLIR.A | N | 16.79 | 618.3523 | 1003.3 | 619.9800 | 1 | 54.06 | 1684 | 1 | 21 | 25 |  |
| R.SNGIT.F | Y | 16.75 | 490.2387 | 2129.6 | 492.2900 | 1 | 66.67 | 2116 | 1 | 160 | 164 |  |
| total 2 peptides |  |  |  |  |  |  |  |  |  |  |  |  |
| --- | --- | --- | --- | --- | --- | --- | --- | --- | --- | --- | --- | --- |

tr|A0A0F6PPF2|A0A0F6PPF2\_9DIPT

back to list

  

| Protein Coverage
| Supporting Peptides
|

Protein Coverage:

Supporting Peptides:

| Peptide | Uniq | -10lgP | Mass | ppm | m/z | z | RT | Scan | #Spec | Start | End | PTM |
| --- | --- | --- | --- | --- | --- | --- | --- | --- | --- | --- | --- | --- |
| L.SMLIR.A | N | 16.79 | 618.3523 | 1003.3 | 619.9800 | 1 | 54.06 | 1684 | 1 | 21 | 25 |  |
| R.SNGIT.F | Y | 16.75 | 490.2387 | 2129.6 | 492.2900 | 1 | 66.67 | 2116 | 1 | 160 | 164 |  |
| total 2 peptides |  |  |  |  |  |  |  |  |  |  |  |  |
| --- | --- | --- | --- | --- | --- | --- | --- | --- | --- | --- | --- | --- |

tr|A0A0F6PQN3|A0A0F6PQN3\_9DIPT

back to list

  

| Protein Coverage
| Supporting Peptides
|

Protein Coverage:

Supporting Peptides:

| Peptide | Uniq | -10lgP | Mass | ppm | m/z | z | RT | Scan | #Spec | Start | End | PTM |
| --- | --- | --- | --- | --- | --- | --- | --- | --- | --- | --- | --- | --- |
| L.SMLIR.A | N | 16.79 | 618.3523 | 1003.3 | 619.9800 | 1 | 54.06 | 1684 | 1 | 21 | 25 |  |
| R.SNGIT.F | Y | 16.75 | 490.2387 | 2129.6 | 492.2900 | 1 | 66.67 | 2116 | 1 | 160 | 164 |  |
| total 2 peptides |  |  |  |  |  |  |  |  |  |  |  |  |
| --- | --- | --- | --- | --- | --- | --- | --- | --- | --- | --- | --- | --- |

tr|A0A0F6PQN7|A0A0F6PQN7\_9DIPT

back to list

  

| Protein Coverage
| Supporting Peptides
|

Protein Coverage:

Supporting Peptides:

| Peptide | Uniq | -10lgP | Mass | ppm | m/z | z | RT | Scan | #Spec | Start | End | PTM |
| --- | --- | --- | --- | --- | --- | --- | --- | --- | --- | --- | --- | --- |
| L.SMLIR.A | N | 16.79 | 618.3523 | 1003.3 | 619.9800 | 1 | 54.06 | 1684 | 1 | 21 | 25 |  |
| R.SNGIT.F | Y | 16.75 | 490.2387 | 2129.6 | 492.2900 | 1 | 66.67 | 2116 | 1 | 160 | 164 |  |
| total 2 peptides |  |  |  |  |  |  |  |  |  |  |  |  |
| --- | --- | --- | --- | --- | --- | --- | --- | --- | --- | --- | --- | --- |

tr|A0A0F6PRN6|A0A0F6PRN6\_9DIPT

back to list

  

| Protein Coverage
| Supporting Peptides
|

Protein Coverage:

Supporting Peptides:

| Peptide | Uniq | -10lgP | Mass | ppm | m/z | z | RT | Scan | #Spec | Start | End | PTM |
| --- | --- | --- | --- | --- | --- | --- | --- | --- | --- | --- | --- | --- |
| L.SMLIR.A | N | 16.79 | 618.3523 | 1003.3 | 619.9800 | 1 | 54.06 | 1684 | 1 | 21 | 25 |  |
| R.SNGIT.F | Y | 16.75 | 490.2387 | 2129.6 | 492.2900 | 1 | 66.67 | 2116 | 1 | 160 | 164 |  |
| total 2 peptides |  |  |  |  |  |  |  |  |  |  |  |  |
| --- | --- | --- | --- | --- | --- | --- | --- | --- | --- | --- | --- | --- |

tr|A0A077D6R9|A0A077D6R9\_9DIPT

back to list

  

| Protein Coverage
| Supporting Peptides
|

Protein Coverage:

Supporting Peptides:

| Peptide | Uniq | -10lgP | Mass | ppm | m/z | z | RT | Scan | #Spec | Start | End | PTM |
| --- | --- | --- | --- | --- | --- | --- | --- | --- | --- | --- | --- | --- |
| L.SMLIR.A | N | 16.79 | 618.3523 | 1003.3 | 619.9800 | 1 | 54.06 | 1684 | 1 | 26 | 30 |  |
| R.SNGIT.F | Y | 16.75 | 490.2387 | 2129.6 | 492.2900 | 1 | 66.67 | 2116 | 1 | 165 | 169 |  |
| total 2 peptides |  |  |  |  |  |  |  |  |  |  |  |  |
| --- | --- | --- | --- | --- | --- | --- | --- | --- | --- | --- | --- | --- |

tr|A0A077D482|A0A077D482\_9DIPT

back to list

  

| Protein Coverage
| Supporting Peptides
|

Protein Coverage:

Supporting Peptides:

| Peptide | Uniq | -10lgP | Mass | ppm | m/z | z | RT | Scan | #Spec | Start | End | PTM |
| --- | --- | --- | --- | --- | --- | --- | --- | --- | --- | --- | --- | --- |
| L.SMLIR.A | N | 16.79 | 618.3523 | 1003.3 | 619.9800 | 1 | 54.06 | 1684 | 1 | 26 | 30 |  |
| R.SNGIT.F | Y | 16.75 | 490.2387 | 2129.6 | 492.2900 | 1 | 66.67 | 2116 | 1 | 165 | 169 |  |
| total 2 peptides |  |  |  |  |  |  |  |  |  |  |  |  |
| --- | --- | --- | --- | --- | --- | --- | --- | --- | --- | --- | --- | --- |

tr|A0A077D9S9|A0A077D9S9\_9DIPT

back to list

  

| Protein Coverage
| Supporting Peptides
|

Protein Coverage:

Supporting Peptides:

| Peptide | Uniq | -10lgP | Mass | ppm | m/z | z | RT | Scan | #Spec | Start | End | PTM |
| --- | --- | --- | --- | --- | --- | --- | --- | --- | --- | --- | --- | --- |
| L.SMLIR.A | N | 16.79 | 618.3523 | 1003.3 | 619.9800 | 1 | 54.06 | 1684 | 1 | 26 | 30 |  |
| R.SNGIT.F | Y | 16.75 | 490.2387 | 2129.6 | 492.2900 | 1 | 66.67 | 2116 | 1 | 165 | 169 |  |
| total 2 peptides |  |  |  |  |  |  |  |  |  |  |  |  |
| --- | --- | --- | --- | --- | --- | --- | --- | --- | --- | --- | --- | --- |

tr|A0A077D8K9|A0A077D8K9\_9DIPT

back to list

  

| Protein Coverage
| Supporting Peptides
|

Protein Coverage:

Supporting Peptides:

| Peptide | Uniq | -10lgP | Mass | ppm | m/z | z | RT | Scan | #Spec | Start | End | PTM |
| --- | --- | --- | --- | --- | --- | --- | --- | --- | --- | --- | --- | --- |
| L.SMLIR.A | N | 16.79 | 618.3523 | 1003.3 | 619.9800 | 1 | 54.06 | 1684 | 1 | 26 | 30 |  |
| R.SNGIT.F | Y | 16.75 | 490.2387 | 2129.6 | 492.2900 | 1 | 66.67 | 2116 | 1 | 165 | 169 |  |
| total 2 peptides |  |  |  |  |  |  |  |  |  |  |  |  |
| --- | --- | --- | --- | --- | --- | --- | --- | --- | --- | --- | --- | --- |

tr|A0A0N9FUK8|A0A0N9FUK8\_9DIPT

back to list

  

| Protein Coverage
| Supporting Peptides
|

Protein Coverage:

Supporting Peptides:

| Peptide | Uniq | -10lgP | Mass | ppm | m/z | z | RT | Scan | #Spec | Start | End | PTM |
| --- | --- | --- | --- | --- | --- | --- | --- | --- | --- | --- | --- | --- |
| L.SMLIR.A | N | 16.79 | 618.3523 | 1003.3 | 619.9800 | 1 | 54.06 | 1684 | 1 | 13 | 17 |  |
| R.SNGIT.F | Y | 16.75 | 490.2387 | 2129.6 | 492.2900 | 1 | 66.67 | 2116 | 1 | 151 | 155 |  |
| total 2 peptides |  |  |  |  |  |  |  |  |  |  |  |  |
| --- | --- | --- | --- | --- | --- | --- | --- | --- | --- | --- | --- | --- |

tr|A0A0N7HMJ8|A0A0N7HMJ8\_9DIPT

back to list

  

| Protein Coverage
| Supporting Peptides
|

Protein Coverage:

Supporting Peptides:

| Peptide | Uniq | -10lgP | Mass | ppm | m/z | z | RT | Scan | #Spec | Start | End | PTM |
| --- | --- | --- | --- | --- | --- | --- | --- | --- | --- | --- | --- | --- |
| L.SMLIR.A | N | 16.79 | 618.3523 | 1003.3 | 619.9800 | 1 | 54.06 | 1684 | 1 | 6 | 10 |  |
| R.SNGIT.F | Y | 16.75 | 490.2387 | 2129.6 | 492.2900 | 1 | 66.67 | 2116 | 1 | 145 | 149 |  |
| total 2 peptides |  |  |  |  |  |  |  |  |  |  |  |  |
| --- | --- | --- | --- | --- | --- | --- | --- | --- | --- | --- | --- | --- |

tr|A0A0N9FT07|A0A0N9FT07\_SIMVI

back to list

  

| Protein Coverage
| Supporting Peptides
|

Protein Coverage:

Supporting Peptides:

| Peptide | Uniq | -10lgP | Mass | ppm | m/z | z | RT | Scan | #Spec | Start | End | PTM |
| --- | --- | --- | --- | --- | --- | --- | --- | --- | --- | --- | --- | --- |
| L.SMLIR.A | N | 16.79 | 618.3523 | 1003.3 | 619.9800 | 1 | 54.06 | 1684 | 1 | 2 | 6 |  |
| R.SNGIT.F | Y | 16.75 | 490.2387 | 2129.6 | 492.2900 | 1 | 66.67 | 2116 | 1 | 141 | 145 |  |
| total 2 peptides |  |  |  |  |  |  |  |  |  |  |  |  |
| --- | --- | --- | --- | --- | --- | --- | --- | --- | --- | --- | --- | --- |

tr|A0A0N9FVD5|A0A0N9FVD5\_SIMVI

back to list

  

| Protein Coverage
| Supporting Peptides
|

Protein Coverage:

Supporting Peptides:

| Peptide | Uniq | -10lgP | Mass | ppm | m/z | z | RT | Scan | #Spec | Start | End | PTM |
| --- | --- | --- | --- | --- | --- | --- | --- | --- | --- | --- | --- | --- |
| L.SMLIR.A | N | 16.79 | 618.3523 | 1003.3 | 619.9800 | 1 | 54.06 | 1684 | 1 | 9 | 13 |  |
| R.SNGIT.F | Y | 16.75 | 490.2387 | 2129.6 | 492.2900 | 1 | 66.67 | 2116 | 1 | 148 | 152 |  |
| total 2 peptides |  |  |  |  |  |  |  |  |  |  |  |  |
| --- | --- | --- | --- | --- | --- | --- | --- | --- | --- | --- | --- | --- |

tr|A0A0N9G0S6|A0A0N9G0S6\_SIMVI

back to list

  

| Protein Coverage
| Supporting Peptides
|

Protein Coverage:

Supporting Peptides:

| Peptide | Uniq | -10lgP | Mass | ppm | m/z | z | RT | Scan | #Spec | Start | End | PTM |
| --- | --- | --- | --- | --- | --- | --- | --- | --- | --- | --- | --- | --- |
| L.SMLIR.A | N | 16.79 | 618.3523 | 1003.3 | 619.9800 | 1 | 54.06 | 1684 | 1 | 10 | 14 |  |
| R.SNGIT.F | Y | 16.75 | 490.2387 | 2129.6 | 492.2900 | 1 | 66.67 | 2116 | 1 | 149 | 153 |  |
| total 2 peptides |  |  |  |  |  |  |  |  |  |  |  |  |
| --- | --- | --- | --- | --- | --- | --- | --- | --- | --- | --- | --- | --- |

tr|A0A0N9FRZ2|A0A0N9FRZ2\_SIMVI

back to list

  

| Protein Coverage
| Supporting Peptides
|

Protein Coverage:

Supporting Peptides:

| Peptide | Uniq | -10lgP | Mass | ppm | m/z | z | RT | Scan | #Spec | Start | End | PTM |
| --- | --- | --- | --- | --- | --- | --- | --- | --- | --- | --- | --- | --- |
| L.SMLIR.A | N | 16.79 | 618.3523 | 1003.3 | 619.9800 | 1 | 54.06 | 1684 | 1 | 9 | 13 |  |
| R.SNGIT.F | Y | 16.75 | 490.2387 | 2129.6 | 492.2900 | 1 | 66.67 | 2116 | 1 | 148 | 152 |  |
| total 2 peptides |  |  |  |  |  |  |  |  |  |  |  |  |
| --- | --- | --- | --- | --- | --- | --- | --- | --- | --- | --- | --- | --- |

tr|A0A0N9FVN2|A0A0N9FVN2\_SIMVI

back to list

  

| Protein Coverage
| Supporting Peptides
|

Protein Coverage:

Supporting Peptides:

| Peptide | Uniq | -10lgP | Mass | ppm | m/z | z | RT | Scan | #Spec | Start | End | PTM |
| --- | --- | --- | --- | --- | --- | --- | --- | --- | --- | --- | --- | --- |
| L.SMLIR.A | N | 16.79 | 618.3523 | 1003.3 | 619.9800 | 1 | 54.06 | 1684 | 1 | 6 | 10 |  |
| R.SNGIT.F | Y | 16.75 | 490.2387 | 2129.6 | 492.2900 | 1 | 66.67 | 2116 | 1 | 145 | 149 |  |
| total 2 peptides |  |  |  |  |  |  |  |  |  |  |  |  |
| --- | --- | --- | --- | --- | --- | --- | --- | --- | --- | --- | --- | --- |

tr|A0A0N9FRW7|A0A0N9FRW7\_SIMVI

back to list

  

| Protein Coverage
| Supporting Peptides
|

Protein Coverage:

Supporting Peptides:

| Peptide | Uniq | -10lgP | Mass | ppm | m/z | z | RT | Scan | #Spec | Start | End | PTM |
| --- | --- | --- | --- | --- | --- | --- | --- | --- | --- | --- | --- | --- |
| L.SMLIR.A | N | 16.79 | 618.3523 | 1003.3 | 619.9800 | 1 | 54.06 | 1684 | 1 | 6 | 10 |  |
| R.SNGIT.F | Y | 16.75 | 490.2387 | 2129.6 | 492.2900 | 1 | 66.67 | 2116 | 1 | 145 | 149 |  |
| total 2 peptides |  |  |  |  |  |  |  |  |  |  |  |  |
| --- | --- | --- | --- | --- | --- | --- | --- | --- | --- | --- | --- | --- |

tr|A0A0N9G226|A0A0N9G226\_SIMVI

back to list

  

| Protein Coverage
| Supporting Peptides
|

Protein Coverage:

Supporting Peptides:

| Peptide | Uniq | -10lgP | Mass | ppm | m/z | z | RT | Scan | #Spec | Start | End | PTM |
| --- | --- | --- | --- | --- | --- | --- | --- | --- | --- | --- | --- | --- |
| L.SMIIR.A | N | 16.79 | 618.3523 | 1003.3 | 619.9800 | 1 | 54.06 | 1684 | 1 | 10 | 14 |  |
| R.SNGIT.F | Y | 16.75 | 490.2387 | 2129.6 | 492.2900 | 1 | 66.67 | 2116 | 1 | 149 | 153 |  |
| total 2 peptides |  |  |  |  |  |  |  |  |  |  |  |  |
| --- | --- | --- | --- | --- | --- | --- | --- | --- | --- | --- | --- | --- |

tr|A0A0N7HQ75|A0A0N7HQ75\_9DIPT

back to list

  

| Protein Coverage
| Supporting Peptides
|

Protein Coverage:

Supporting Peptides:

| Peptide | Uniq | -10lgP | Mass | ppm | m/z | z | RT | Scan | #Spec | Start | End | PTM |
| --- | --- | --- | --- | --- | --- | --- | --- | --- | --- | --- | --- | --- |
| L.SMLIR.A | N | 16.79 | 618.3523 | 1003.3 | 619.9800 | 1 | 54.06 | 1684 | 1 | 8 | 12 |  |
| R.SNGIT.F | Y | 16.75 | 490.2387 | 2129.6 | 492.2900 | 1 | 66.67 | 2116 | 1 | 147 | 151 |  |
| total 2 peptides |  |  |  |  |  |  |  |  |  |  |  |  |
| --- | --- | --- | --- | --- | --- | --- | --- | --- | --- | --- | --- | --- |

tr|A0A0P0B2U5|A0A0P0B2U5\_9DIPT

back to list

  

| Protein Coverage
| Supporting Peptides
|

Protein Coverage:

Supporting Peptides:

| Peptide | Uniq | -10lgP | Mass | ppm | m/z | z | RT | Scan | #Spec | Start | End | PTM |
| --- | --- | --- | --- | --- | --- | --- | --- | --- | --- | --- | --- | --- |
| L.SMLIR.A | N | 16.79 | 618.3523 | 1003.3 | 619.9800 | 1 | 54.06 | 1684 | 1 | 8 | 12 |  |
| R.SNGIT.F | Y | 16.75 | 490.2387 | 2129.6 | 492.2900 | 1 | 66.67 | 2116 | 1 | 147 | 151 |  |
| total 2 peptides |  |  |  |  |  |  |  |  |  |  |  |  |
| --- | --- | --- | --- | --- | --- | --- | --- | --- | --- | --- | --- | --- |

tr|A0A0N9G394|A0A0N9G394\_SIMVI

back to list

  

| Protein Coverage
| Supporting Peptides
|

Protein Coverage:

Supporting Peptides:

| Peptide | Uniq | -10lgP | Mass | ppm | m/z | z | RT | Scan | #Spec | Start | End | PTM |
| --- | --- | --- | --- | --- | --- | --- | --- | --- | --- | --- | --- | --- |
| L.SMLIR.A | N | 16.79 | 618.3523 | 1003.3 | 619.9800 | 1 | 54.06 | 1684 | 1 | 9 | 13 |  |
| R.SNGIT.F | Y | 16.75 | 490.2387 | 2129.6 | 492.2900 | 1 | 66.67 | 2116 | 1 | 148 | 152 |  |
| total 2 peptides |  |  |  |  |  |  |  |  |  |  |  |  |
| --- | --- | --- | --- | --- | --- | --- | --- | --- | --- | --- | --- | --- |

tr|A0A0N9G330|A0A0N9G330\_SIMVI

back to list

  

| Protein Coverage
| Supporting Peptides
|

Protein Coverage:

Supporting Peptides:

| Peptide | Uniq | -10lgP | Mass | ppm | m/z | z | RT | Scan | #Spec | Start | End | PTM |
| --- | --- | --- | --- | --- | --- | --- | --- | --- | --- | --- | --- | --- |
| L.SMLIR.A | N | 16.79 | 618.3523 | 1003.3 | 619.9800 | 1 | 54.06 | 1684 | 1 | 9 | 13 |  |
| R.SNGIT.F | Y | 16.75 | 490.2387 | 2129.6 | 492.2900 | 1 | 66.67 | 2116 | 1 | 148 | 152 |  |
| total 2 peptides |  |  |  |  |  |  |  |  |  |  |  |  |
| --- | --- | --- | --- | --- | --- | --- | --- | --- | --- | --- | --- | --- |

tr|A0A0N9FZW7|A0A0N9FZW7\_SIMVI

back to list

  

| Protein Coverage
| Supporting Peptides
|

Protein Coverage:

Supporting Peptides:

| Peptide | Uniq | -10lgP | Mass | ppm | m/z | z | RT | Scan | #Spec | Start | End | PTM |
| --- | --- | --- | --- | --- | --- | --- | --- | --- | --- | --- | --- | --- |
| L.SMLIR.A | N | 16.79 | 618.3523 | 1003.3 | 619.9800 | 1 | 54.06 | 1684 | 1 | 10 | 14 |  |
| R.SNGIT.F | Y | 16.75 | 490.2387 | 2129.6 | 492.2900 | 1 | 66.67 | 2116 | 1 | 149 | 153 |  |
| total 2 peptides |  |  |  |  |  |  |  |  |  |  |  |  |
| --- | --- | --- | --- | --- | --- | --- | --- | --- | --- | --- | --- | --- |

tr|A0A0N9G108|A0A0N9G108\_SIMVI

back to list

  

| Protein Coverage
| Supporting Peptides
|

Protein Coverage:

Supporting Peptides:

| Peptide | Uniq | -10lgP | Mass | ppm | m/z | z | RT | Scan | #Spec | Start | End | PTM |
| --- | --- | --- | --- | --- | --- | --- | --- | --- | --- | --- | --- | --- |
| L.SMLIR.A | N | 16.79 | 618.3523 | 1003.3 | 619.9800 | 1 | 54.06 | 1684 | 1 | 10 | 14 |  |
| R.SNGIT.F | Y | 16.75 | 490.2387 | 2129.6 | 492.2900 | 1 | 66.67 | 2116 | 1 | 149 | 153 |  |
| total 2 peptides |  |  |  |  |  |  |  |  |  |  |  |  |
| --- | --- | --- | --- | --- | --- | --- | --- | --- | --- | --- | --- | --- |

tr|A0A0N9FZA3|A0A0N9FZA3\_SIMVI

back to list

  

| Protein Coverage
| Supporting Peptides
|

Protein Coverage:

Supporting Peptides:

| Peptide | Uniq | -10lgP | Mass | ppm | m/z | z | RT | Scan | #Spec | Start | End | PTM |
| --- | --- | --- | --- | --- | --- | --- | --- | --- | --- | --- | --- | --- |
| L.SMLIR.A | N | 16.79 | 618.3523 | 1003.3 | 619.9800 | 1 | 54.06 | 1684 | 1 | 8 | 12 |  |
| R.SNGIT.F | Y | 16.75 | 490.2387 | 2129.6 | 492.2900 | 1 | 66.67 | 2116 | 1 | 147 | 151 |  |
| total 2 peptides |  |  |  |  |  |  |  |  |  |  |  |  |
| --- | --- | --- | --- | --- | --- | --- | --- | --- | --- | --- | --- | --- |

tr|A0A0N9FXU3|A0A0N9FXU3\_SIMVI

back to list

  

| Protein Coverage
| Supporting Peptides
|

Protein Coverage:

Supporting Peptides:

| Peptide | Uniq | -10lgP | Mass | ppm | m/z | z | RT | Scan | #Spec | Start | End | PTM |
| --- | --- | --- | --- | --- | --- | --- | --- | --- | --- | --- | --- | --- |
| L.SMLIR.A | N | 16.79 | 618.3523 | 1003.3 | 619.9800 | 1 | 54.06 | 1684 | 1 | 9 | 13 |  |
| R.SNGIT.F | Y | 16.75 | 490.2387 | 2129.6 | 492.2900 | 1 | 66.67 | 2116 | 1 | 148 | 152 |  |
| total 2 peptides |  |  |  |  |  |  |  |  |  |  |  |  |
| --- | --- | --- | --- | --- | --- | --- | --- | --- | --- | --- | --- | --- |

tr|A0A0N9G1J3|A0A0N9G1J3\_SIMVI

back to list

  

| Protein Coverage
| Supporting Peptides
|

Protein Coverage:

Supporting Peptides:

| Peptide | Uniq | -10lgP | Mass | ppm | m/z | z | RT | Scan | #Spec | Start | End | PTM |
| --- | --- | --- | --- | --- | --- | --- | --- | --- | --- | --- | --- | --- |
| L.SMLIR.A | N | 16.79 | 618.3523 | 1003.3 | 619.9800 | 1 | 54.06 | 1684 | 1 | 10 | 14 |  |
| R.SNGIT.F | Y | 16.75 | 490.2387 | 2129.6 | 492.2900 | 1 | 66.67 | 2116 | 1 | 149 | 153 |  |
| total 2 peptides |  |  |  |  |  |  |  |  |  |  |  |  |
| --- | --- | --- | --- | --- | --- | --- | --- | --- | --- | --- | --- | --- |

tr|A0A0N9FYE2|A0A0N9FYE2\_SIMVI

back to list

  

| Protein Coverage
| Supporting Peptides
|

Protein Coverage:

Supporting Peptides:

| Peptide | Uniq | -10lgP | Mass | ppm | m/z | z | RT | Scan | #Spec | Start | End | PTM |
| --- | --- | --- | --- | --- | --- | --- | --- | --- | --- | --- | --- | --- |
| L.SMLIR.A | N | 16.79 | 618.3523 | 1003.3 | 619.9800 | 1 | 54.06 | 1684 | 1 | 14 | 18 |  |
| R.SNGIT.F | Y | 16.75 | 490.2387 | 2129.6 | 492.2900 | 1 | 66.67 | 2116 | 1 | 153 | 157 |  |
| total 2 peptides |  |  |  |  |  |  |  |  |  |  |  |  |
| --- | --- | --- | --- | --- | --- | --- | --- | --- | --- | --- | --- | --- |

tr|A0A0P0AVW9|A0A0P0AVW9\_9DIPT

back to list

  

| Protein Coverage
| Supporting Peptides
|

Protein Coverage:

Supporting Peptides:

| Peptide | Uniq | -10lgP | Mass | ppm | m/z | z | RT | Scan | #Spec | Start | End | PTM |
| --- | --- | --- | --- | --- | --- | --- | --- | --- | --- | --- | --- | --- |
| L.SMLIR.A | N | 16.79 | 618.3523 | 1003.3 | 619.9800 | 1 | 54.06 | 1684 | 1 | 14 | 18 |  |
| R.SNGIT.F | Y | 16.75 | 490.2387 | 2129.6 | 492.2900 | 1 | 66.67 | 2116 | 1 | 153 | 157 |  |
| total 2 peptides |  |  |  |  |  |  |  |  |  |  |  |  |
| --- | --- | --- | --- | --- | --- | --- | --- | --- | --- | --- | --- | --- |

tr|A0A0R6MKB5|A0A0R6MKB5\_9DIPT

back to list

  

| Protein Coverage
| Supporting Peptides
|

Protein Coverage:

Supporting Peptides:

| Peptide | Uniq | -10lgP | Mass | ppm | m/z | z | RT | Scan | #Spec | Start | End | PTM |
| --- | --- | --- | --- | --- | --- | --- | --- | --- | --- | --- | --- | --- |
| L.SMLIR.A | N | 16.79 | 618.3523 | 1003.3 | 619.9800 | 1 | 54.06 | 1684 | 1 | 5 | 9 |  |
| R.SNGIT.F | Y | 16.75 | 490.2387 | 2129.6 | 492.2900 | 1 | 66.67 | 2116 | 1 | 144 | 148 |  |
| total 2 peptides |  |  |  |  |  |  |  |  |  |  |  |  |
| --- | --- | --- | --- | --- | --- | --- | --- | --- | --- | --- | --- | --- |

tr|A0A0N9FUS4|A0A0N9FUS4\_SIMVI

back to list

  

| Protein Coverage
| Supporting Peptides
|

Protein Coverage:

Supporting Peptides:

| Peptide | Uniq | -10lgP | Mass | ppm | m/z | z | RT | Scan | #Spec | Start | End | PTM |
| --- | --- | --- | --- | --- | --- | --- | --- | --- | --- | --- | --- | --- |
| L.SMLIR.A | N | 16.79 | 618.3523 | 1003.3 | 619.9800 | 1 | 54.06 | 1684 | 1 | 18 | 22 |  |
| R.SNGIT.F | Y | 16.75 | 490.2387 | 2129.6 | 492.2900 | 1 | 66.67 | 2116 | 1 | 157 | 161 |  |
| total 2 peptides |  |  |  |  |  |  |  |  |  |  |  |  |
| --- | --- | --- | --- | --- | --- | --- | --- | --- | --- | --- | --- | --- |

tr|A0A0P0BTU2|A0A0P0BTU2\_9DIPT

back to list

  

| Protein Coverage
| Supporting Peptides
|

Protein Coverage:

Supporting Peptides:

| Peptide | Uniq | -10lgP | Mass | ppm | m/z | z | RT | Scan | #Spec | Start | End | PTM |
| --- | --- | --- | --- | --- | --- | --- | --- | --- | --- | --- | --- | --- |
| L.SMLIR.A | N | 16.79 | 618.3523 | 1003.3 | 619.9800 | 1 | 54.06 | 1684 | 1 | 14 | 18 |  |
| R.SNGIT.F | Y | 16.75 | 490.2387 | 2129.6 | 492.2900 | 1 | 66.67 | 2116 | 1 | 153 | 157 |  |
| total 2 peptides |  |  |  |  |  |  |  |  |  |  |  |  |
| --- | --- | --- | --- | --- | --- | --- | --- | --- | --- | --- | --- | --- |

tr|A0A0N7ESR4|A0A0N7ESR4\_9DIPT

back to list

  

| Protein Coverage
| Supporting Peptides
|

Protein Coverage:

Supporting Peptides:

| Peptide | Uniq | -10lgP | Mass | ppm | m/z | z | RT | Scan | #Spec | Start | End | PTM |
| --- | --- | --- | --- | --- | --- | --- | --- | --- | --- | --- | --- | --- |
| L.SMLIR.A | N | 16.79 | 618.3523 | 1003.3 | 619.9800 | 1 | 54.06 | 1684 | 1 | 14 | 18 |  |
| R.SNGIT.F | Y | 16.75 | 490.2387 | 2129.6 | 492.2900 | 1 | 66.67 | 2116 | 1 | 153 | 157 |  |
| total 2 peptides |  |  |  |  |  |  |  |  |  |  |  |  |
| --- | --- | --- | --- | --- | --- | --- | --- | --- | --- | --- | --- | --- |

tr|A0A0H3VZQ4|A0A0H3VZQ4\_9DIPT

back to list

  

| Protein Coverage
| Supporting Peptides
|

Protein Coverage:

Supporting Peptides:

| Peptide | Uniq | -10lgP | Mass | ppm | m/z | z | RT | Scan | #Spec | Start | End | PTM |
| --- | --- | --- | --- | --- | --- | --- | --- | --- | --- | --- | --- | --- |
| L.SMLIR.A | N | 16.79 | 618.3523 | 1003.3 | 619.9800 | 1 | 54.06 | 1684 | 1 | 6 | 10 |  |
| R.SNGIT.F | Y | 16.75 | 490.2387 | 2129.6 | 492.2900 | 1 | 66.67 | 2116 | 1 | 145 | 149 |  |
| total 2 peptides |  |  |  |  |  |  |  |  |  |  |  |  |
| --- | --- | --- | --- | --- | --- | --- | --- | --- | --- | --- | --- | --- |

tr|A0A0P0AR86|A0A0P0AR86\_9DIPT

back to list

  

| Protein Coverage
| Supporting Peptides
|

Protein Coverage:

Supporting Peptides:

| Peptide | Uniq | -10lgP | Mass | ppm | m/z | z | RT | Scan | #Spec | Start | End | PTM |
| --- | --- | --- | --- | --- | --- | --- | --- | --- | --- | --- | --- | --- |
| L.SMLIR.A | N | 16.79 | 618.3523 | 1003.3 | 619.9800 | 1 | 54.06 | 1684 | 1 | 18 | 22 |  |
| R.SNGIT.F | Y | 16.75 | 490.2387 | 2129.6 | 492.2900 | 1 | 66.67 | 2116 | 1 | 157 | 161 |  |
| total 2 peptides |  |  |  |  |  |  |  |  |  |  |  |  |
| --- | --- | --- | --- | --- | --- | --- | --- | --- | --- | --- | --- | --- |

tr|A0A0P0A1S1|A0A0P0A1S1\_9DIPT

back to list

  

| Protein Coverage
| Supporting Peptides
|

Protein Coverage:

Supporting Peptides:

| Peptide | Uniq | -10lgP | Mass | ppm | m/z | z | RT | Scan | #Spec | Start | End | PTM |
| --- | --- | --- | --- | --- | --- | --- | --- | --- | --- | --- | --- | --- |
| L.SMLIR.A | N | 16.79 | 618.3523 | 1003.3 | 619.9800 | 1 | 54.06 | 1684 | 1 | 18 | 22 |  |
| R.SNGIT.F | Y | 16.75 | 490.2387 | 2129.6 | 492.2900 | 1 | 66.67 | 2116 | 1 | 157 | 161 |  |
| total 2 peptides |  |  |  |  |  |  |  |  |  |  |  |  |
| --- | --- | --- | --- | --- | --- | --- | --- | --- | --- | --- | --- | --- |

tr|A0A0R6MN67|A0A0R6MN67\_9DIPT

back to list

  

| Protein Coverage
| Supporting Peptides
|

Protein Coverage:

Supporting Peptides:

| Peptide | Uniq | -10lgP | Mass | ppm | m/z | z | RT | Scan | #Spec | Start | End | PTM |
| --- | --- | --- | --- | --- | --- | --- | --- | --- | --- | --- | --- | --- |
| L.SMLIR.A | N | 16.79 | 618.3523 | 1003.3 | 619.9800 | 1 | 54.06 | 1684 | 1 | 2 | 6 |  |
| R.SNGIT.F | Y | 16.75 | 490.2387 | 2129.6 | 492.2900 | 1 | 66.67 | 2116 | 1 | 141 | 145 |  |
| total 2 peptides |  |  |  |  |  |  |  |  |  |  |  |  |
| --- | --- | --- | --- | --- | --- | --- | --- | --- | --- | --- | --- | --- |

tr|A0A0R6MJZ1|A0A0R6MJZ1\_9DIPT

back to list

  

| Protein Coverage
| Supporting Peptides
|

Protein Coverage:

Supporting Peptides:

| Peptide | Uniq | -10lgP | Mass | ppm | m/z | z | RT | Scan | #Spec | Start | End | PTM |
| --- | --- | --- | --- | --- | --- | --- | --- | --- | --- | --- | --- | --- |
| L.SMLIR.A | N | 16.79 | 618.3523 | 1003.3 | 619.9800 | 1 | 54.06 | 1684 | 1 | 3 | 7 |  |
| R.SNGIT.F | Y | 16.75 | 490.2387 | 2129.6 | 492.2900 | 1 | 66.67 | 2116 | 1 | 142 | 146 |  |
| total 2 peptides |  |  |  |  |  |  |  |  |  |  |  |  |
| --- | --- | --- | --- | --- | --- | --- | --- | --- | --- | --- | --- | --- |

tr|A0A0H3W048|A0A0H3W048\_9DIPT

back to list

  

| Protein Coverage
| Supporting Peptides
|

Protein Coverage:

Supporting Peptides:

| Peptide | Uniq | -10lgP | Mass | ppm | m/z | z | RT | Scan | #Spec | Start | End | PTM |
| --- | --- | --- | --- | --- | --- | --- | --- | --- | --- | --- | --- | --- |
| L.SMLIR.A | N | 16.79 | 618.3523 | 1003.3 | 619.9800 | 1 | 54.06 | 1684 | 1 | 12 | 16 |  |
| R.SNGIT.F | Y | 16.75 | 490.2387 | 2129.6 | 492.2900 | 1 | 66.67 | 2116 | 1 | 151 | 155 |  |
| total 2 peptides |  |  |  |  |  |  |  |  |  |  |  |  |
| --- | --- | --- | --- | --- | --- | --- | --- | --- | --- | --- | --- | --- |

tr|A0A0H3W0E2|A0A0H3W0E2\_9DIPT

back to list

  

| Protein Coverage
| Supporting Peptides
|

Protein Coverage:

Supporting Peptides:

| Peptide | Uniq | -10lgP | Mass | ppm | m/z | z | RT | Scan | #Spec | Start | End | PTM |
| --- | --- | --- | --- | --- | --- | --- | --- | --- | --- | --- | --- | --- |
| L.SMLIR.A | N | 16.79 | 618.3523 | 1003.3 | 619.9800 | 1 | 54.06 | 1684 | 1 | 12 | 16 |  |
| R.SNGIT.F | Y | 16.75 | 490.2387 | 2129.6 | 492.2900 | 1 | 66.67 | 2116 | 1 | 151 | 155 |  |
| total 2 peptides |  |  |  |  |  |  |  |  |  |  |  |  |
| --- | --- | --- | --- | --- | --- | --- | --- | --- | --- | --- | --- | --- |

tr|A0A0H3W044|A0A0H3W044\_9DIPT

back to list

  

| Protein Coverage
| Supporting Peptides
|

Protein Coverage:

Supporting Peptides:

| Peptide | Uniq | -10lgP | Mass | ppm | m/z | z | RT | Scan | #Spec | Start | End | PTM |
| --- | --- | --- | --- | --- | --- | --- | --- | --- | --- | --- | --- | --- |
| L.SMLIR.A | N | 16.79 | 618.3523 | 1003.3 | 619.9800 | 1 | 54.06 | 1684 | 1 | 12 | 16 |  |
| R.SNGIT.F | Y | 16.75 | 490.2387 | 2129.6 | 492.2900 | 1 | 66.67 | 2116 | 1 | 151 | 155 |  |
| total 2 peptides |  |  |  |  |  |  |  |  |  |  |  |  |
| --- | --- | --- | --- | --- | --- | --- | --- | --- | --- | --- | --- | --- |

tr|A0A0H3W046|A0A0H3W046\_9DIPT

back to list

  

| Protein Coverage
| Supporting Peptides
|

Protein Coverage:

Supporting Peptides:

| Peptide | Uniq | -10lgP | Mass | ppm | m/z | z | RT | Scan | #Spec | Start | End | PTM |
| --- | --- | --- | --- | --- | --- | --- | --- | --- | --- | --- | --- | --- |
| L.SMLIR.A | N | 16.79 | 618.3523 | 1003.3 | 619.9800 | 1 | 54.06 | 1684 | 1 | 12 | 16 |  |
| R.SNGIT.F | Y | 16.75 | 490.2387 | 2129.6 | 492.2900 | 1 | 66.67 | 2116 | 1 | 151 | 155 |  |
| total 2 peptides |  |  |  |  |  |  |  |  |  |  |  |  |
| --- | --- | --- | --- | --- | --- | --- | --- | --- | --- | --- | --- | --- |

tr|A0A0H3VZQ5|A0A0H3VZQ5\_9DIPT

back to list

  

| Protein Coverage
| Supporting Peptides
|

Protein Coverage:

Supporting Peptides:

| Peptide | Uniq | -10lgP | Mass | ppm | m/z | z | RT | Scan | #Spec | Start | End | PTM |
| --- | --- | --- | --- | --- | --- | --- | --- | --- | --- | --- | --- | --- |
| L.SMLIR.A | N | 16.79 | 618.3523 | 1003.3 | 619.9800 | 1 | 54.06 | 1684 | 1 | 12 | 16 |  |
| R.SNGIT.F | Y | 16.75 | 490.2387 | 2129.6 | 492.2900 | 1 | 66.67 | 2116 | 1 | 151 | 155 |  |
| total 2 peptides |  |  |  |  |  |  |  |  |  |  |  |  |
| --- | --- | --- | --- | --- | --- | --- | --- | --- | --- | --- | --- | --- |

tr|A0A0H3W049|A0A0H3W049\_9DIPT

back to list

  

| Protein Coverage
| Supporting Peptides
|

Protein Coverage:

Supporting Peptides:

| Peptide | Uniq | -10lgP | Mass | ppm | m/z | z | RT | Scan | #Spec | Start | End | PTM |
| --- | --- | --- | --- | --- | --- | --- | --- | --- | --- | --- | --- | --- |
| L.SMLIR.A | N | 16.79 | 618.3523 | 1003.3 | 619.9800 | 1 | 54.06 | 1684 | 1 | 12 | 16 |  |
| R.SNGIT.F | Y | 16.75 | 490.2387 | 2129.6 | 492.2900 | 1 | 66.67 | 2116 | 1 | 151 | 155 |  |
| total 2 peptides |  |  |  |  |  |  |  |  |  |  |  |  |
| --- | --- | --- | --- | --- | --- | --- | --- | --- | --- | --- | --- | --- |

tr|A0A0H3W0E7|A0A0H3W0E7\_9DIPT

back to list

  

| Protein Coverage
| Supporting Peptides
|

Protein Coverage:

Supporting Peptides:

| Peptide | Uniq | -10lgP | Mass | ppm | m/z | z | RT | Scan | #Spec | Start | End | PTM |
| --- | --- | --- | --- | --- | --- | --- | --- | --- | --- | --- | --- | --- |
| L.SMLIR.A | N | 16.79 | 618.3523 | 1003.3 | 619.9800 | 1 | 54.06 | 1684 | 1 | 12 | 16 |  |
| R.SNGIT.F | Y | 16.75 | 490.2387 | 2129.6 | 492.2900 | 1 | 66.67 | 2116 | 1 | 151 | 155 |  |
| total 2 peptides |  |  |  |  |  |  |  |  |  |  |  |  |
| --- | --- | --- | --- | --- | --- | --- | --- | --- | --- | --- | --- | --- |

tr|A0A0H3VZQ1|A0A0H3VZQ1\_9DIPT

back to list

  

| Protein Coverage
| Supporting Peptides
|

Protein Coverage:

Supporting Peptides:

| Peptide | Uniq | -10lgP | Mass | ppm | m/z | z | RT | Scan | #Spec | Start | End | PTM |
| --- | --- | --- | --- | --- | --- | --- | --- | --- | --- | --- | --- | --- |
| L.SMLIR.A | N | 16.79 | 618.3523 | 1003.3 | 619.9800 | 1 | 54.06 | 1684 | 1 | 12 | 16 |  |
| R.SNGIT.F | Y | 16.75 | 490.2387 | 2129.6 | 492.2900 | 1 | 66.67 | 2116 | 1 | 151 | 155 |  |
| total 2 peptides |  |  |  |  |  |  |  |  |  |  |  |  |
| --- | --- | --- | --- | --- | --- | --- | --- | --- | --- | --- | --- | --- |

tr|A0A0H3W051|A0A0H3W051\_9DIPT

back to list

  

| Protein Coverage
| Supporting Peptides
|

Protein Coverage:

Supporting Peptides:

| Peptide | Uniq | -10lgP | Mass | ppm | m/z | z | RT | Scan | #Spec | Start | End | PTM |
| --- | --- | --- | --- | --- | --- | --- | --- | --- | --- | --- | --- | --- |
| L.SMLIR.A | N | 16.79 | 618.3523 | 1003.3 | 619.9800 | 1 | 54.06 | 1684 | 1 | 12 | 16 |  |
| R.SNGIT.F | Y | 16.75 | 490.2387 | 2129.6 | 492.2900 | 1 | 66.67 | 2116 | 1 | 151 | 155 |  |
| total 2 peptides |  |  |  |  |  |  |  |  |  |  |  |  |
| --- | --- | --- | --- | --- | --- | --- | --- | --- | --- | --- | --- | --- |

tr|A0A0H3VZQ6|A0A0H3VZQ6\_9DIPT

back to list

  

| Protein Coverage
| Supporting Peptides
|

Protein Coverage:

Supporting Peptides:

| Peptide | Uniq | -10lgP | Mass | ppm | m/z | z | RT | Scan | #Spec | Start | End | PTM |
| --- | --- | --- | --- | --- | --- | --- | --- | --- | --- | --- | --- | --- |
| L.SMLIR.A | N | 16.79 | 618.3523 | 1003.3 | 619.9800 | 1 | 54.06 | 1684 | 1 | 12 | 16 |  |
| R.SNGIT.F | Y | 16.75 | 490.2387 | 2129.6 | 492.2900 | 1 | 66.67 | 2116 | 1 | 151 | 155 |  |
| total 2 peptides |  |  |  |  |  |  |  |  |  |  |  |  |
| --- | --- | --- | --- | --- | --- | --- | --- | --- | --- | --- | --- | --- |

tr|A0A0H3W0E4|A0A0H3W0E4\_9DIPT

back to list

  

| Protein Coverage
| Supporting Peptides
|

Protein Coverage:

Supporting Peptides:

| Peptide | Uniq | -10lgP | Mass | ppm | m/z | z | RT | Scan | #Spec | Start | End | PTM |
| --- | --- | --- | --- | --- | --- | --- | --- | --- | --- | --- | --- | --- |
| L.SMLIR.A | N | 16.79 | 618.3523 | 1003.3 | 619.9800 | 1 | 54.06 | 1684 | 1 | 12 | 16 |  |
| R.SNGIT.F | Y | 16.75 | 490.2387 | 2129.6 | 492.2900 | 1 | 66.67 | 2116 | 1 | 151 | 155 |  |
| total 2 peptides |  |  |  |  |  |  |  |  |  |  |  |  |
| --- | --- | --- | --- | --- | --- | --- | --- | --- | --- | --- | --- | --- |

tr|A0A0H3W0R9|A0A0H3W0R9\_9DIPT

back to list

  

| Protein Coverage
| Supporting Peptides
|

Protein Coverage:

Supporting Peptides:

| Peptide | Uniq | -10lgP | Mass | ppm | m/z | z | RT | Scan | #Spec | Start | End | PTM |
| --- | --- | --- | --- | --- | --- | --- | --- | --- | --- | --- | --- | --- |
| L.SMLIR.A | N | 16.79 | 618.3523 | 1003.3 | 619.9800 | 1 | 54.06 | 1684 | 1 | 12 | 16 |  |
| R.SNGIT.F | Y | 16.75 | 490.2387 | 2129.6 | 492.2900 | 1 | 66.67 | 2116 | 1 | 151 | 155 |  |
| total 2 peptides |  |  |  |  |  |  |  |  |  |  |  |  |
| --- | --- | --- | --- | --- | --- | --- | --- | --- | --- | --- | --- | --- |

tr|A0A0H3W0E8|A0A0H3W0E8\_9DIPT

back to list

  

| Protein Coverage
| Supporting Peptides
|

Protein Coverage:

Supporting Peptides:

| Peptide | Uniq | -10lgP | Mass | ppm | m/z | z | RT | Scan | #Spec | Start | End | PTM |
| --- | --- | --- | --- | --- | --- | --- | --- | --- | --- | --- | --- | --- |
| L.SMLIR.A | N | 16.79 | 618.3523 | 1003.3 | 619.9800 | 1 | 54.06 | 1684 | 1 | 12 | 16 |  |
| R.SNGIT.F | Y | 16.75 | 490.2387 | 2129.6 | 492.2900 | 1 | 66.67 | 2116 | 1 | 151 | 155 |  |
| total 2 peptides |  |  |  |  |  |  |  |  |  |  |  |  |
| --- | --- | --- | --- | --- | --- | --- | --- | --- | --- | --- | --- | --- |

tr|A0A0H3W037|A0A0H3W037\_9DIPT

back to list

  

| Protein Coverage
| Supporting Peptides
|

Protein Coverage:

Supporting Peptides:

| Peptide | Uniq | -10lgP | Mass | ppm | m/z | z | RT | Scan | #Spec | Start | End | PTM |
| --- | --- | --- | --- | --- | --- | --- | --- | --- | --- | --- | --- | --- |
| L.SMLIR.A | N | 16.79 | 618.3523 | 1003.3 | 619.9800 | 1 | 54.06 | 1684 | 1 | 12 | 16 |  |
| R.SNGIT.F | Y | 16.75 | 490.2387 | 2129.6 | 492.2900 | 1 | 66.67 | 2116 | 1 | 151 | 155 |  |
| total 2 peptides |  |  |  |  |  |  |  |  |  |  |  |  |
| --- | --- | --- | --- | --- | --- | --- | --- | --- | --- | --- | --- | --- |

tr|A0A0R6MM04|A0A0R6MM04\_9DIPT

back to list

  

| Protein Coverage
| Supporting Peptides
|

Protein Coverage:

Supporting Peptides:

| Peptide | Uniq | -10lgP | Mass | ppm | m/z | z | RT | Scan | #Spec | Start | End | PTM |
| --- | --- | --- | --- | --- | --- | --- | --- | --- | --- | --- | --- | --- |
| L.SMLIR.A | N | 16.79 | 618.3523 | 1003.3 | 619.9800 | 1 | 54.06 | 1684 | 1 | 6 | 10 |  |
| R.SNGIT.F | Y | 16.75 | 490.2387 | 2129.6 | 492.2900 | 1 | 66.67 | 2116 | 1 | 145 | 149 |  |
| total 2 peptides |  |  |  |  |  |  |  |  |  |  |  |  |
| --- | --- | --- | --- | --- | --- | --- | --- | --- | --- | --- | --- | --- |

tr|A0A0H3W0E6|A0A0H3W0E6\_9DIPT

back to list

  

| Protein Coverage
| Supporting Peptides
|

Protein Coverage:

Supporting Peptides:

| Peptide | Uniq | -10lgP | Mass | ppm | m/z | z | RT | Scan | #Spec | Start | End | PTM |
| --- | --- | --- | --- | --- | --- | --- | --- | --- | --- | --- | --- | --- |
| L.SMLIR.A | N | 16.79 | 618.3523 | 1003.3 | 619.9800 | 1 | 54.06 | 1684 | 1 | 12 | 16 |  |
| R.SNGIT.F | Y | 16.75 | 490.2387 | 2129.6 | 492.2900 | 1 | 66.67 | 2116 | 1 | 151 | 155 |  |
| total 2 peptides |  |  |  |  |  |  |  |  |  |  |  |  |
| --- | --- | --- | --- | --- | --- | --- | --- | --- | --- | --- | --- | --- |

tr|A0A0P0AJX7|A0A0P0AJX7\_9DIPT

back to list

  

| Protein Coverage
| Supporting Peptides
|

Protein Coverage:

Supporting Peptides:

| Peptide | Uniq | -10lgP | Mass | ppm | m/z | z | RT | Scan | #Spec | Start | End | PTM |
| --- | --- | --- | --- | --- | --- | --- | --- | --- | --- | --- | --- | --- |
| L.SMLIR.A | N | 16.79 | 618.3523 | 1003.3 | 619.9800 | 1 | 54.06 | 1684 | 1 | 18 | 22 |  |
| R.SNGIT.F | Y | 16.75 | 490.2387 | 2129.6 | 492.2900 | 1 | 66.67 | 2116 | 1 | 157 | 161 |  |
| total 2 peptides |  |  |  |  |  |  |  |  |  |  |  |  |
| --- | --- | --- | --- | --- | --- | --- | --- | --- | --- | --- | --- | --- |

tr|A0A0R6MM60|A0A0R6MM60\_9DIPT

back to list

  

| Protein Coverage
| Supporting Peptides
|

Protein Coverage:

Supporting Peptides:

| Peptide | Uniq | -10lgP | Mass | ppm | m/z | z | RT | Scan | #Spec | Start | End | PTM |
| --- | --- | --- | --- | --- | --- | --- | --- | --- | --- | --- | --- | --- |
| L.SMLIR.A | N | 16.79 | 618.3523 | 1003.3 | 619.9800 | 1 | 54.06 | 1684 | 1 | 11 | 15 |  |
| R.SNGIT.F | Y | 16.75 | 490.2387 | 2129.6 | 492.2900 | 1 | 66.67 | 2116 | 1 | 150 | 154 |  |
| total 2 peptides |  |  |  |  |  |  |  |  |  |  |  |  |
| --- | --- | --- | --- | --- | --- | --- | --- | --- | --- | --- | --- | --- |

tr|A0A0K0PVD6|A0A0K0PVD6\_9DIPT

back to list

  

| Protein Coverage
| Supporting Peptides
|

Protein Coverage:

Supporting Peptides:

| Peptide | Uniq | -10lgP | Mass | ppm | m/z | z | RT | Scan | #Spec | Start | End | PTM |
| --- | --- | --- | --- | --- | --- | --- | --- | --- | --- | --- | --- | --- |
| L.SMLIR.A | N | 16.79 | 618.3523 | 1003.3 | 619.9800 | 1 | 54.06 | 1684 | 1 | 12 | 16 |  |
| R.SNGIT.F | Y | 16.75 | 490.2387 | 2129.6 | 492.2900 | 1 | 66.67 | 2116 | 1 | 151 | 155 |  |
| total 2 peptides |  |  |  |  |  |  |  |  |  |  |  |  |
| --- | --- | --- | --- | --- | --- | --- | --- | --- | --- | --- | --- | --- |

tr|A0A068FGJ4|A0A068FGJ4\_9DIPT

back to list

  

| Protein Coverage
| Supporting Peptides
|

Protein Coverage:

Supporting Peptides:

| Peptide | Uniq | -10lgP | Mass | ppm | m/z | z | RT | Scan | #Spec | Start | End | PTM |
| --- | --- | --- | --- | --- | --- | --- | --- | --- | --- | --- | --- | --- |
| L.SMLIR.A | N | 16.79 | 618.3523 | 1003.3 | 619.9800 | 1 | 54.06 | 1684 | 1 | 12 | 16 |  |
| R.SNGIT.F | Y | 16.75 | 490.2387 | 2129.6 | 492.2900 | 1 | 66.67 | 2116 | 1 | 151 | 155 |  |
| total 2 peptides |  |  |  |  |  |  |  |  |  |  |  |  |
| --- | --- | --- | --- | --- | --- | --- | --- | --- | --- | --- | --- | --- |

tr|A0A0H3W042|A0A0H3W042\_9DIPT

back to list

  

| Protein Coverage
| Supporting Peptides
|

Protein Coverage:

Supporting Peptides:

| Peptide | Uniq | -10lgP | Mass | ppm | m/z | z | RT | Scan | #Spec | Start | End | PTM |
| --- | --- | --- | --- | --- | --- | --- | --- | --- | --- | --- | --- | --- |
| L.SMLIR.A | N | 16.79 | 618.3523 | 1003.3 | 619.9800 | 1 | 54.06 | 1684 | 1 | 12 | 16 |  |
| R.SNGIT.F | Y | 16.75 | 490.2387 | 2129.6 | 492.2900 | 1 | 66.67 | 2116 | 1 | 151 | 155 |  |
| total 2 peptides |  |  |  |  |  |  |  |  |  |  |  |  |
| --- | --- | --- | --- | --- | --- | --- | --- | --- | --- | --- | --- | --- |

tr|A0A0H3W0R7|A0A0H3W0R7\_9DIPT

back to list

  

| Protein Coverage
| Supporting Peptides
|

Protein Coverage:

Supporting Peptides:

| Peptide | Uniq | -10lgP | Mass | ppm | m/z | z | RT | Scan | #Spec | Start | End | PTM |
| --- | --- | --- | --- | --- | --- | --- | --- | --- | --- | --- | --- | --- |
| L.SMLIR.A | N | 16.79 | 618.3523 | 1003.3 | 619.9800 | 1 | 54.06 | 1684 | 1 | 12 | 16 |  |
| R.SNGIT.F | Y | 16.75 | 490.2387 | 2129.6 | 492.2900 | 1 | 66.67 | 2116 | 1 | 151 | 155 |  |
| total 2 peptides |  |  |  |  |  |  |  |  |  |  |  |  |
| --- | --- | --- | --- | --- | --- | --- | --- | --- | --- | --- | --- | --- |

tr|A0A0H4SZ59|A0A0H4SZ59\_9DIPT

back to list

  

| Protein Coverage
| Supporting Peptides
|

Protein Coverage:

Supporting Peptides:

| Peptide | Uniq | -10lgP | Mass | ppm | m/z | z | RT | Scan | #Spec | Start | End | PTM |
| --- | --- | --- | --- | --- | --- | --- | --- | --- | --- | --- | --- | --- |
| L.SMLIR.A | N | 16.79 | 618.3523 | 1003.3 | 619.9800 | 1 | 54.06 | 1684 | 1 | 29 | 33 |  |
| R.SNGIT.F | Y | 16.75 | 490.2387 | 2129.6 | 492.2900 | 1 | 66.67 | 2116 | 1 | 168 | 172 |  |
| total 2 peptides |  |  |  |  |  |  |  |  |  |  |  |  |
| --- | --- | --- | --- | --- | --- | --- | --- | --- | --- | --- | --- | --- |

tr|A0A023J419|A0A023J419\_9DIPT

back to list

  

| Protein Coverage
| Supporting Peptides
|

Protein Coverage:

Supporting Peptides:

| Peptide | Uniq | -10lgP | Mass | ppm | m/z | z | RT | Scan | #Spec | Start | End | PTM |
| --- | --- | --- | --- | --- | --- | --- | --- | --- | --- | --- | --- | --- |
| R.SSSFS.T | Y | 17.04 | 513.2071 | -1918.1 | 513.2300 | 1 | 65.26 | 2068 | 1 | 636 | 640 |  |
| A.MAAAT.X | Y | 16.17 | 463.2101 | 2207.7 | 465.2400 | 1 | 84.48 | 2722 | 1 | 95 | 99 |  |
| total 2 peptides |  |  |  |  |  |  |  |  |  |  |  |  |
| --- | --- | --- | --- | --- | --- | --- | --- | --- | --- | --- | --- | --- |

tr|A0A023J489|A0A023J489\_9DIPT

back to list

  

| Protein Coverage
| Supporting Peptides
|

Protein Coverage:

Supporting Peptides:

| Peptide | Uniq | -10lgP | Mass | ppm | m/z | z | RT | Scan | #Spec | Start | End | PTM |
| --- | --- | --- | --- | --- | --- | --- | --- | --- | --- | --- | --- | --- |
| R.SSSFS.T | Y | 17.04 | 513.2071 | -1918.1 | 513.2300 | 1 | 65.26 | 2068 | 1 | 637 | 641 |  |
| A.MAAAT.X | Y | 16.17 | 463.2101 | 2207.7 | 465.2400 | 1 | 84.48 | 2722 | 1 | 95 | 99 |  |
| total 2 peptides |  |  |  |  |  |  |  |  |  |  |  |  |
| --- | --- | --- | --- | --- | --- | --- | --- | --- | --- | --- | --- | --- |

tr|A0A023J3Z8|A0A023J3Z8\_9DIPT

back to list

  

| Protein Coverage
| Supporting Peptides
|

Protein Coverage:

Supporting Peptides:

| Peptide | Uniq | -10lgP | Mass | ppm | m/z | z | RT | Scan | #Spec | Start | End | PTM |
| --- | --- | --- | --- | --- | --- | --- | --- | --- | --- | --- | --- | --- |
| R.SSSFS.T | Y | 17.04 | 513.2071 | -1918.1 | 513.2300 | 1 | 65.26 | 2068 | 1 | 636 | 640 |  |
| A.MAAAT.X | Y | 16.17 | 463.2101 | 2207.7 | 465.2400 | 1 | 84.48 | 2722 | 1 | 95 | 99 |  |
| total 2 peptides |  |  |  |  |  |  |  |  |  |  |  |  |
| --- | --- | --- | --- | --- | --- | --- | --- | --- | --- | --- | --- | --- |

tr|A0A023J469|A0A023J469\_9DIPT

back to list

  

| Protein Coverage
| Supporting Peptides
|

Protein Coverage:

Supporting Peptides:

| Peptide | Uniq | -10lgP | Mass | ppm | m/z | z | RT | Scan | #Spec | Start | End | PTM |
| --- | --- | --- | --- | --- | --- | --- | --- | --- | --- | --- | --- | --- |
| R.SSSFS.T | Y | 17.04 | 513.2071 | -1918.1 | 513.2300 | 1 | 65.26 | 2068 | 1 | 638 | 642 |  |
| A.MAAAT.X | Y | 16.17 | 463.2101 | 2207.7 | 465.2400 | 1 | 84.48 | 2722 | 1 | 97 | 101 |  |
| total 2 peptides |  |  |  |  |  |  |  |  |  |  |  |  |
| --- | --- | --- | --- | --- | --- | --- | --- | --- | --- | --- | --- | --- |

tr|B5M0P8|B5M0P8\_SIMVI

back to list

  

| Protein Coverage
| Supporting Peptides
|

Protein Coverage:

Supporting Peptides:

| Peptide | Uniq | -10lgP | Mass | ppm | m/z | z | RT | Scan | #Spec | Start | End | PTM |
| --- | --- | --- | --- | --- | --- | --- | --- | --- | --- | --- | --- | --- |
| V.GLASK.N | Y | 16.30 | 474.2802 | 47.5 | 475.3100 | 1 | 91.86 | 2970 | 1 | 49 | 53 |  |
| K.NSATN.V | Y | 15.58 | 505.2133 | 137.5 | 506.2900 | 1 | 75.50 | 2418 | 1 | 43 | 47 |  |
| total 2 peptides |  |  |  |  |  |  |  |  |  |  |  |  |
| --- | --- | --- | --- | --- | --- | --- | --- | --- | --- | --- | --- | --- |

tr|A0A023GYN2|A0A023GYN2\_9DIPT

back to list

  

| Protein Coverage
| Supporting Peptides
|

Protein Coverage:

Supporting Peptides:

| Peptide | Uniq | -10lgP | Mass | ppm | m/z | z | RT | Scan | #Spec | Start | End | PTM |
| --- | --- | --- | --- | --- | --- | --- | --- | --- | --- | --- | --- | --- |
| K.TTPSA.T | Y | 22.47 | 475.2278 | 115.6 | 476.2900 | 1 | 42.45 | 1286 | 2 | 637 | 641 |  |
| total 1 peptides |  |  |  |  |  |  |  |  |  |  |  |  |
| --- | --- | --- | --- | --- | --- | --- | --- | --- | --- | --- | --- | --- |

tr|A0A023GYR7|A0A023GYR7\_9DIPT

back to list

  

| Protein Coverage
| Supporting Peptides
|

Protein Coverage:

Supporting Peptides:

| Peptide | Uniq | -10lgP | Mass | ppm | m/z | z | RT | Scan | #Spec | Start | End | PTM |
| --- | --- | --- | --- | --- | --- | --- | --- | --- | --- | --- | --- | --- |
| K.TTPSA.T | Y | 22.47 | 475.2278 | 115.6 | 476.2900 | 1 | 42.45 | 1286 | 2 | 637 | 641 |  |
| total 1 peptides |  |  |  |  |  |  |  |  |  |  |  |  |
| --- | --- | --- | --- | --- | --- | --- | --- | --- | --- | --- | --- | --- |

tr|A0A023GYQ2|A0A023GYQ2\_9DIPT

back to list

  

| Protein Coverage
| Supporting Peptides
|

Protein Coverage:

Supporting Peptides:

| Peptide | Uniq | -10lgP | Mass | ppm | m/z | z | RT | Scan | #Spec | Start | End | PTM |
| --- | --- | --- | --- | --- | --- | --- | --- | --- | --- | --- | --- | --- |
| K.TTPSA.T | Y | 22.47 | 475.2278 | 115.6 | 476.2900 | 1 | 42.45 | 1286 | 2 | 637 | 641 |  |
| total 1 peptides |  |  |  |  |  |  |  |  |  |  |  |  |
| --- | --- | --- | --- | --- | --- | --- | --- | --- | --- | --- | --- | --- |

tr|A0A023GYL6|A0A023GYL6\_9DIPT

back to list

  

| Protein Coverage
| Supporting Peptides
|

Protein Coverage:

Supporting Peptides:

| Peptide | Uniq | -10lgP | Mass | ppm | m/z | z | RT | Scan | #Spec | Start | End | PTM |
| --- | --- | --- | --- | --- | --- | --- | --- | --- | --- | --- | --- | --- |
| K.TTPSA.T | Y | 22.47 | 475.2278 | 115.6 | 476.2900 | 1 | 42.45 | 1286 | 2 | 638 | 642 |  |
| total 1 peptides |  |  |  |  |  |  |  |  |  |  |  |  |
| --- | --- | --- | --- | --- | --- | --- | --- | --- | --- | --- | --- | --- |

tr|A0A023GYN6|A0A023GYN6\_9DIPT

back to list

  

| Protein Coverage
| Supporting Peptides
|

Protein Coverage:

Supporting Peptides:

| Peptide | Uniq | -10lgP | Mass | ppm | m/z | z | RT | Scan | #Spec | Start | End | PTM |
| --- | --- | --- | --- | --- | --- | --- | --- | --- | --- | --- | --- | --- |
| K.TTPSA.T | Y | 22.47 | 475.2278 | 115.6 | 476.2900 | 1 | 42.45 | 1286 | 2 | 638 | 642 |  |
| total 1 peptides |  |  |  |  |  |  |  |  |  |  |  |  |
| --- | --- | --- | --- | --- | --- | --- | --- | --- | --- | --- | --- | --- |

tr|A0A023GYR1|A0A023GYR1\_9DIPT

back to list

  

| Protein Coverage
| Supporting Peptides
|

Protein Coverage:

Supporting Peptides:

| Peptide | Uniq | -10lgP | Mass | ppm | m/z | z | RT | Scan | #Spec | Start | End | PTM |
| --- | --- | --- | --- | --- | --- | --- | --- | --- | --- | --- | --- | --- |
| K.TTPSA.X | Y | 22.47 | 475.2278 | 115.6 | 476.2900 | 1 | 42.45 | 1286 | 2 | 638 | 642 |  |
| total 1 peptides |  |  |  |  |  |  |  |  |  |  |  |  |
| --- | --- | --- | --- | --- | --- | --- | --- | --- | --- | --- | --- | --- |

tr|A0A023GYR8|A0A023GYR8\_9DIPT

back to list

  

| Protein Coverage
| Supporting Peptides
|

Protein Coverage:

Supporting Peptides:

| Peptide | Uniq | -10lgP | Mass | ppm | m/z | z | RT | Scan | #Spec | Start | End | PTM |
| --- | --- | --- | --- | --- | --- | --- | --- | --- | --- | --- | --- | --- |
| K.TTPSA.T | Y | 22.47 | 475.2278 | 115.6 | 476.2900 | 1 | 42.45 | 1286 | 2 | 638 | 642 |  |
| total 1 peptides |  |  |  |  |  |  |  |  |  |  |  |  |
| --- | --- | --- | --- | --- | --- | --- | --- | --- | --- | --- | --- | --- |

tr|A0A023GYL2|A0A023GYL2\_9DIPT

back to list

  

| Protein Coverage
| Supporting Peptides
|

Protein Coverage:

Supporting Peptides:

| Peptide | Uniq | -10lgP | Mass | ppm | m/z | z | RT | Scan | #Spec | Start | End | PTM |
| --- | --- | --- | --- | --- | --- | --- | --- | --- | --- | --- | --- | --- |
| K.TTPSA.T | Y | 22.47 | 475.2278 | 115.6 | 476.2900 | 1 | 42.45 | 1286 | 2 | 638 | 642 |  |
| total 1 peptides |  |  |  |  |  |  |  |  |  |  |  |  |
| --- | --- | --- | --- | --- | --- | --- | --- | --- | --- | --- | --- | --- |

tr|A0A023GYM4|A0A023GYM4\_9DIPT

back to list

  

| Protein Coverage
| Supporting Peptides
|

Protein Coverage:

Supporting Peptides:

| Peptide | Uniq | -10lgP | Mass | ppm | m/z | z | RT | Scan | #Spec | Start | End | PTM |
| --- | --- | --- | --- | --- | --- | --- | --- | --- | --- | --- | --- | --- |
| K.TTPSA.T | Y | 22.47 | 475.2278 | 115.6 | 476.2900 | 1 | 42.45 | 1286 | 2 | 638 | 642 |  |
| total 1 peptides |  |  |  |  |  |  |  |  |  |  |  |  |
| --- | --- | --- | --- | --- | --- | --- | --- | --- | --- | --- | --- | --- |

tr|A0A023GYN1|A0A023GYN1\_9DIPT

back to list

  

| Protein Coverage
| Supporting Peptides
|

Protein Coverage:

Supporting Peptides:

| Peptide | Uniq | -10lgP | Mass | ppm | m/z | z | RT | Scan | #Spec | Start | End | PTM |
| --- | --- | --- | --- | --- | --- | --- | --- | --- | --- | --- | --- | --- |
| K.TTPSA.T | Y | 22.47 | 475.2278 | 115.6 | 476.2900 | 1 | 42.45 | 1286 | 2 | 638 | 642 |  |
| total 1 peptides |  |  |  |  |  |  |  |  |  |  |  |  |
| --- | --- | --- | --- | --- | --- | --- | --- | --- | --- | --- | --- | --- |

tr|A0A023GYQ5|A0A023GYQ5\_9DIPT

back to list

  

| Protein Coverage
| Supporting Peptides
|

Protein Coverage:

Supporting Peptides:

| Peptide | Uniq | -10lgP | Mass | ppm | m/z | z | RT | Scan | #Spec | Start | End | PTM |
| --- | --- | --- | --- | --- | --- | --- | --- | --- | --- | --- | --- | --- |
| K.TTPSA.T | Y | 22.47 | 475.2278 | 115.6 | 476.2900 | 1 | 42.45 | 1286 | 2 | 638 | 642 |  |
| total 1 peptides |  |  |  |  |  |  |  |  |  |  |  |  |
| --- | --- | --- | --- | --- | --- | --- | --- | --- | --- | --- | --- | --- |

tr|A0A023GYP6|A0A023GYP6\_9DIPT

back to list

  

| Protein Coverage
| Supporting Peptides
|

Protein Coverage:

Supporting Peptides:

| Peptide | Uniq | -10lgP | Mass | ppm | m/z | z | RT | Scan | #Spec | Start | End | PTM |
| --- | --- | --- | --- | --- | --- | --- | --- | --- | --- | --- | --- | --- |
| K.TTPSA.T | Y | 22.47 | 475.2278 | 115.6 | 476.2900 | 1 | 42.45 | 1286 | 2 | 638 | 642 |  |
| total 1 peptides |  |  |  |  |  |  |  |  |  |  |  |  |
| --- | --- | --- | --- | --- | --- | --- | --- | --- | --- | --- | --- | --- |

tr|A0A023GYM1|A0A023GYM1\_9DIPT

back to list

  

| Protein Coverage
| Supporting Peptides
|

Protein Coverage:

Supporting Peptides:

| Peptide | Uniq | -10lgP | Mass | ppm | m/z | z | RT | Scan | #Spec | Start | End | PTM |
| --- | --- | --- | --- | --- | --- | --- | --- | --- | --- | --- | --- | --- |
| K.TTPSA.T | Y | 22.47 | 475.2278 | 115.6 | 476.2900 | 1 | 42.45 | 1286 | 2 | 638 | 642 |  |
| total 1 peptides |  |  |  |  |  |  |  |  |  |  |  |  |
| --- | --- | --- | --- | --- | --- | --- | --- | --- | --- | --- | --- | --- |

tr|A0A023GYT1|A0A023GYT1\_9DIPT

back to list

  

| Protein Coverage
| Supporting Peptides
|

Protein Coverage:

Supporting Peptides:

| Peptide | Uniq | -10lgP | Mass | ppm | m/z | z | RT | Scan | #Spec | Start | End | PTM |
| --- | --- | --- | --- | --- | --- | --- | --- | --- | --- | --- | --- | --- |
| K.TTPSA.T | Y | 22.47 | 475.2278 | 115.6 | 476.2900 | 1 | 42.45 | 1286 | 2 | 638 | 642 |  |
| total 1 peptides |  |  |  |  |  |  |  |  |  |  |  |  |
| --- | --- | --- | --- | --- | --- | --- | --- | --- | --- | --- | --- | --- |

tr|A0A023GYQ7|A0A023GYQ7\_9DIPT

back to list

  

| Protein Coverage
| Supporting Peptides
|

Protein Coverage:

Supporting Peptides:

| Peptide | Uniq | -10lgP | Mass | ppm | m/z | z | RT | Scan | #Spec | Start | End | PTM |
| --- | --- | --- | --- | --- | --- | --- | --- | --- | --- | --- | --- | --- |
| K.TTPSA.T | Y | 22.47 | 475.2278 | 115.6 | 476.2900 | 1 | 42.45 | 1286 | 2 | 638 | 642 |  |
| total 1 peptides |  |  |  |  |  |  |  |  |  |  |  |  |
| --- | --- | --- | --- | --- | --- | --- | --- | --- | --- | --- | --- | --- |

tr|A0A023GYN7|A0A023GYN7\_9DIPT

back to list

  

| Protein Coverage
| Supporting Peptides
|

Protein Coverage:

Supporting Peptides:

| Peptide | Uniq | -10lgP | Mass | ppm | m/z | z | RT | Scan | #Spec | Start | End | PTM |
| --- | --- | --- | --- | --- | --- | --- | --- | --- | --- | --- | --- | --- |
| K.TTPSA.T | Y | 22.47 | 475.2278 | 115.6 | 476.2900 | 1 | 42.45 | 1286 | 2 | 638 | 642 |  |
| total 1 peptides |  |  |  |  |  |  |  |  |  |  |  |  |
| --- | --- | --- | --- | --- | --- | --- | --- | --- | --- | --- | --- | --- |

tr|A0A023GYS0|A0A023GYS0\_9DIPT

back to list

  

| Protein Coverage
| Supporting Peptides
|

Protein Coverage:

Supporting Peptides:

| Peptide | Uniq | -10lgP | Mass | ppm | m/z | z | RT | Scan | #Spec | Start | End | PTM |
| --- | --- | --- | --- | --- | --- | --- | --- | --- | --- | --- | --- | --- |
| K.TTPSA.T | Y | 22.47 | 475.2278 | 115.6 | 476.2900 | 1 | 42.45 | 1286 | 2 | 638 | 642 |  |
| total 1 peptides |  |  |  |  |  |  |  |  |  |  |  |  |
| --- | --- | --- | --- | --- | --- | --- | --- | --- | --- | --- | --- | --- |

tr|A0A023GYT3|A0A023GYT3\_9DIPT

back to list

  

| Protein Coverage
| Supporting Peptides
|

Protein Coverage:

Supporting Peptides:

| Peptide | Uniq | -10lgP | Mass | ppm | m/z | z | RT | Scan | #Spec | Start | End | PTM |
| --- | --- | --- | --- | --- | --- | --- | --- | --- | --- | --- | --- | --- |
| K.TTPSA.T | Y | 22.47 | 475.2278 | 115.6 | 476.2900 | 1 | 42.45 | 1286 | 2 | 638 | 642 |  |
| total 1 peptides |  |  |  |  |  |  |  |  |  |  |  |  |
| --- | --- | --- | --- | --- | --- | --- | --- | --- | --- | --- | --- | --- |

tr|A0A023GYM5|A0A023GYM5\_9DIPT

back to list

  

| Protein Coverage
| Supporting Peptides
|

Protein Coverage:

Supporting Peptides:

| Peptide | Uniq | -10lgP | Mass | ppm | m/z | z | RT | Scan | #Spec | Start | End | PTM |
| --- | --- | --- | --- | --- | --- | --- | --- | --- | --- | --- | --- | --- |
| K.TTPSA.T | Y | 22.47 | 475.2278 | 115.6 | 476.2900 | 1 | 42.45 | 1286 | 2 | 638 | 642 |  |
| total 1 peptides |  |  |  |  |  |  |  |  |  |  |  |  |
| --- | --- | --- | --- | --- | --- | --- | --- | --- | --- | --- | --- | --- |

tr|A0A023GYL7|A0A023GYL7\_9DIPT

back to list

  

| Protein Coverage
| Supporting Peptides
|

Protein Coverage:

Supporting Peptides:

| Peptide | Uniq | -10lgP | Mass | ppm | m/z | z | RT | Scan | #Spec | Start | End | PTM |
| --- | --- | --- | --- | --- | --- | --- | --- | --- | --- | --- | --- | --- |
| K.TTPSA.T | Y | 22.47 | 475.2278 | 115.6 | 476.2900 | 1 | 42.45 | 1286 | 2 | 638 | 642 |  |
| total 1 peptides |  |  |  |  |  |  |  |  |  |  |  |  |
| --- | --- | --- | --- | --- | --- | --- | --- | --- | --- | --- | --- | --- |

tr|A0A023GYN8|A0A023GYN8\_9DIPT

back to list

  

| Protein Coverage
| Supporting Peptides
|

Protein Coverage:

Supporting Peptides:

| Peptide | Uniq | -10lgP | Mass | ppm | m/z | z | RT | Scan | #Spec | Start | End | PTM |
| --- | --- | --- | --- | --- | --- | --- | --- | --- | --- | --- | --- | --- |
| K.TTPSA.T | Y | 22.47 | 475.2278 | 115.6 | 476.2900 | 1 | 42.45 | 1286 | 2 | 638 | 642 |  |
| total 1 peptides |  |  |  |  |  |  |  |  |  |  |  |  |
| --- | --- | --- | --- | --- | --- | --- | --- | --- | --- | --- | --- | --- |

tr|A0A023GYM3|A0A023GYM3\_9DIPT

back to list

  

| Protein Coverage
| Supporting Peptides
|

Protein Coverage:

Supporting Peptides:

| Peptide | Uniq | -10lgP | Mass | ppm | m/z | z | RT | Scan | #Spec | Start | End | PTM |
| --- | --- | --- | --- | --- | --- | --- | --- | --- | --- | --- | --- | --- |
| K.TTPSA.T | Y | 22.47 | 475.2278 | 115.6 | 476.2900 | 1 | 42.45 | 1286 | 2 | 638 | 642 |  |
| total 1 peptides |  |  |  |  |  |  |  |  |  |  |  |  |
| --- | --- | --- | --- | --- | --- | --- | --- | --- | --- | --- | --- | --- |

tr|A0A023GYT2|A0A023GYT2\_9DIPT

back to list

  

| Protein Coverage
| Supporting Peptides
|

Protein Coverage:

Supporting Peptides:

| Peptide | Uniq | -10lgP | Mass | ppm | m/z | z | RT | Scan | #Spec | Start | End | PTM |
| --- | --- | --- | --- | --- | --- | --- | --- | --- | --- | --- | --- | --- |
| K.TTPSA.T | Y | 22.47 | 475.2278 | 115.6 | 476.2900 | 1 | 42.45 | 1286 | 2 | 638 | 642 |  |
| total 1 peptides |  |  |  |  |  |  |  |  |  |  |  |  |
| --- | --- | --- | --- | --- | --- | --- | --- | --- | --- | --- | --- | --- |

tr|A0A023GYP8|A0A023GYP8\_9DIPT

back to list

  

| Protein Coverage
| Supporting Peptides
|

Protein Coverage:

Supporting Peptides:

| Peptide | Uniq | -10lgP | Mass | ppm | m/z | z | RT | Scan | #Spec | Start | End | PTM |
| --- | --- | --- | --- | --- | --- | --- | --- | --- | --- | --- | --- | --- |
| K.TTPSA.T | Y | 22.47 | 475.2278 | 115.6 | 476.2900 | 1 | 42.45 | 1286 | 2 | 639 | 643 |  |
| total 1 peptides |  |  |  |  |  |  |  |  |  |  |  |  |
| --- | --- | --- | --- | --- | --- | --- | --- | --- | --- | --- | --- | --- |

tr|A0A023GYR3|A0A023GYR3\_9DIPT

back to list

  

| Protein Coverage
| Supporting Peptides
|

Protein Coverage:

Supporting Peptides:

| Peptide | Uniq | -10lgP | Mass | ppm | m/z | z | RT | Scan | #Spec | Start | End | PTM |
| --- | --- | --- | --- | --- | --- | --- | --- | --- | --- | --- | --- | --- |
| K.TTPSA.T | Y | 22.47 | 475.2278 | 115.6 | 476.2900 | 1 | 42.45 | 1286 | 2 | 639 | 643 |  |
| total 1 peptides |  |  |  |  |  |  |  |  |  |  |  |  |
| --- | --- | --- | --- | --- | --- | --- | --- | --- | --- | --- | --- | --- |

tr|A0A023GYQ3|A0A023GYQ3\_9DIPT

back to list

  

| Protein Coverage
| Supporting Peptides
|

Protein Coverage:

Supporting Peptides:

| Peptide | Uniq | -10lgP | Mass | ppm | m/z | z | RT | Scan | #Spec | Start | End | PTM |
| --- | --- | --- | --- | --- | --- | --- | --- | --- | --- | --- | --- | --- |
| K.TTPSA.T | Y | 22.47 | 475.2278 | 115.6 | 476.2900 | 1 | 42.45 | 1286 | 2 | 639 | 643 |  |
| total 1 peptides |  |  |  |  |  |  |  |  |  |  |  |  |
| --- | --- | --- | --- | --- | --- | --- | --- | --- | --- | --- | --- | --- |

tr|A0A023GYL8|A0A023GYL8\_9DIPT

back to list

  

| Protein Coverage
| Supporting Peptides
|

Protein Coverage:

Supporting Peptides:

| Peptide | Uniq | -10lgP | Mass | ppm | m/z | z | RT | Scan | #Spec | Start | End | PTM |
| --- | --- | --- | --- | --- | --- | --- | --- | --- | --- | --- | --- | --- |
| K.TTPSA.T | Y | 22.47 | 475.2278 | 115.6 | 476.2900 | 1 | 42.45 | 1286 | 2 | 639 | 643 |  |
| total 1 peptides |  |  |  |  |  |  |  |  |  |  |  |  |
| --- | --- | --- | --- | --- | --- | --- | --- | --- | --- | --- | --- | --- |

tr|A0A023GYS5|A0A023GYS5\_9DIPT

back to list

  

| Protein Coverage
| Supporting Peptides
|

Protein Coverage:

Supporting Peptides:

| Peptide | Uniq | -10lgP | Mass | ppm | m/z | z | RT | Scan | #Spec | Start | End | PTM |
| --- | --- | --- | --- | --- | --- | --- | --- | --- | --- | --- | --- | --- |
| K.TTPSA.T | Y | 22.47 | 475.2278 | 115.6 | 476.2900 | 1 | 42.45 | 1286 | 2 | 638 | 642 |  |
| total 1 peptides |  |  |  |  |  |  |  |  |  |  |  |  |
| --- | --- | --- | --- | --- | --- | --- | --- | --- | --- | --- | --- | --- |

tr|A0A023GYP0|A0A023GYP0\_9DIPT

back to list

  

| Protein Coverage
| Supporting Peptides
|

Protein Coverage:

Supporting Peptides:

| Peptide | Uniq | -10lgP | Mass | ppm | m/z | z | RT | Scan | #Spec | Start | End | PTM |
| --- | --- | --- | --- | --- | --- | --- | --- | --- | --- | --- | --- | --- |
| K.TTPSA.T | Y | 22.47 | 475.2278 | 115.6 | 476.2900 | 1 | 42.45 | 1286 | 2 | 638 | 642 |  |
| total 1 peptides |  |  |  |  |  |  |  |  |  |  |  |  |
| --- | --- | --- | --- | --- | --- | --- | --- | --- | --- | --- | --- | --- |

tr|A0A023GYM2|A0A023GYM2\_9DIPT

back to list

  

| Protein Coverage
| Supporting Peptides
|

Protein Coverage:

Supporting Peptides:

| Peptide | Uniq | -10lgP | Mass | ppm | m/z | z | RT | Scan | #Spec | Start | End | PTM |
| --- | --- | --- | --- | --- | --- | --- | --- | --- | --- | --- | --- | --- |
| K.TTPSA.T | Y | 22.47 | 475.2278 | 115.6 | 476.2900 | 1 | 42.45 | 1286 | 2 | 638 | 642 |  |
| total 1 peptides |  |  |  |  |  |  |  |  |  |  |  |  |
| --- | --- | --- | --- | --- | --- | --- | --- | --- | --- | --- | --- | --- |

tr|A0A023GYS9|A0A023GYS9\_9DIPT

back to list

  

| Protein Coverage
| Supporting Peptides
|

Protein Coverage:

Supporting Peptides:

| Peptide | Uniq | -10lgP | Mass | ppm | m/z | z | RT | Scan | #Spec | Start | End | PTM |
| --- | --- | --- | --- | --- | --- | --- | --- | --- | --- | --- | --- | --- |
| K.TTPSA.T | Y | 22.47 | 475.2278 | 115.6 | 476.2900 | 1 | 42.45 | 1286 | 2 | 637 | 641 |  |
| total 1 peptides |  |  |  |  |  |  |  |  |  |  |  |  |
| --- | --- | --- | --- | --- | --- | --- | --- | --- | --- | --- | --- | --- |

---

Prepared with PEAKS ™ (bioinfor.com)
